# Topological zero-dimensional defect and flux states in three-dimensional insulators

**DOI:** 10.1038/s41467-022-33471-x

**Published:** 2022-10-02

**Authors:** Frank Schindler, Stepan S. Tsirkin, Titus Neupert, B. Andrei Bernevig, Benjamin J. Wieder

**Affiliations:** 1 Princeton Center for Theoretical Science, Princeton University, Princeton, NJ 08544 USA; 2 Department of Physics, University of Zurich, Winterthurerstrasse 190, 8057 Zurich, Switzerland; 3 Donostia International Physics Center, P. Manuel de Lardizabal 4, 20018 Donostia-San Sebastian, Spain; 4 IKERBASQUE, Basque Foundation for Science, Bilbao, Spain; 5 Department of Physics, Princeton University, Princeton, NJ 08544 USA; 6 Department of Physics, Northeastern University, Boston, MA 02115 USA; 7 Present Address: Department of Physics, Princeton University, Princeton, NJ 08544 USA; 8 Present Address: Department of Physics, Massachusetts Institute of Technology, Cambridge, MA 02139 USA

**Keywords:** Topological defects, Topological insulators, Electronic properties and materials

## Abstract

In insulating crystals, it was previously shown that defects with two fewer dimensions than the bulk can bind topological electronic states. We here further extend the classification of topological defect states by demonstrating that the corners of crystalline defects with integer Burgers vectors can bind 0D higher-order end (HEND) states with anomalous charge and spin. We demonstrate that HEND states are intrinsic topological consequences of the bulk electronic structure and introduce new bulk topological invariants that are predictive of HEND dislocation states in solid-state materials. We demonstrate the presence of first-order 0D defect states in PbTe monolayers and HEND states in 3D SnTe crystals. We relate our analysis to magnetic flux insertion in insulating crystals. We find that *π*-flux tubes in inversion- and time-reversal-symmetric (helical) higher-order topological insulators bind Kramers pairs of spin-charge-separated HEND states, which represent observable signatures of anomalous surface half quantum spin Hall states.

## Abstract (web summary)

Many topological crystalline phases have unknown physical responses. Here, the authors systematically extend the theory of defect and flux responses to predict zero-dimensional (0D) states in topological crystalline materials, including 2D PbTe monolayers and 3D SnTe.

## Introduction

In crystalline solids, there are numerous sources of disorder and defects. One type of crystal defect—integer dislocations—can manifest as edge dislocations, in which planes of atoms are missing within a region of the sample. Integer dislocations can also manifest as screw dislocations, in which planes of atoms in a portion of the crystal are successively shifted by an integer linear combination of lattice vectors^1^. Screw and edge dislocations—which locally represent 1D line defects in 3D crystals—are each characterized by a gauge-invariant Burgers vector ***B***.

In pristine crystals—defined by the absence of disorder and defects—the electronic states form bands, which may be classified by their topological properties^2–17^. When a crystal exhibits unitary symmetries beyond translation—such as spatial inversion (${{{{{{{\mathcal{I}}}}}}}}$), then the band topology may conveniently be diagnosed by symmetry eigenvalues through elementary band representations, which give rise to symmetry-based indicators^18,19^. Well-established symmetry-based indicators of insulating band topology include the Fu–Kane parity criterion^4^, and the strong 3D ${{\mathbb{Z}}}_{4}$ and weak 2D ${{\mathbb{Z}}}_{2}$ invariants of ${{{{{{{\mathcal{I}}}}}}}}$- and time-reversal- (${{{{{{{\mathcal{T}}}}}}}}$-) symmetric 3D insulators^20–23^.

Over the past decade, numerous proposals have been introduced to link the seemingly disparate limits of pristine crystalline solids with nontrivial electronic band topology and the more realistic setting of crystals hosting defects^24–30^. This has led to the identification of electronic defect states in both topological insulators (TIs)^2–6^ and topological crystalline insulators (TCIs)^7–14^. In particular, it has been extensively demonstrated^24–29^ that screw and edge dislocations in ${{{{{{{\mathcal{T}}}}}}}}$-symmetric 3D insulators can bind helical pairs of 1D states if the defect Burgers vector aligns with the weak-index vector ***M***_*ν*_ = (*ν*_*x*_, *ν*_*y*_, *ν*_*z*_):

1 $${{{{{{{\boldsymbol{B}}}}}}}}\cdot {{{{{{{{\boldsymbol{M}}}}}}}}}_{\nu }\,{{{{{{{\rm{mod}}}}}}}}\ 2\pi=\pi,$$

where *ν*_*i*_ is the ${{\mathbb{Z}}}_{2}$-valued weak index in the *k*_*i*_ = *π* plane^4^. For a ${{{{{{{\mathcal{T}}}}}}}}$-symmetric 3D insulator with vanishing strong indices^4,10,15,20–23^, ***M***_*ν*_ ≠ ***0*** further indicates that the insulator can be adiabatically deformed without breaking a symmetry or closing a gap into a decoupled stack of 2D TIs—known as a weak TI^4^. In weak TIs hosting defects with ***B*** directed along the stacking direction, (***B*** ⋅ ***M***_*ν*_)/*π* indicates the number of decoupled 2D layers connecting the crystal defects. Hence intuitively, if (***B*** ⋅ ***M***_*ν*_)/*π* is odd [i.e. Eq. (1) is satisfied], then the defects carry robust helical modes. In terms of momentum-space band topology, Eq. (1) and its ${{{{{{{\mathcal{T}}}}}}}}$-broken variant^26^ predict defect bound states. They respectively diagnose which of the Brillouin-zone- (BZ-) boundary planes have Hamiltonians that are topologically equivalent to 2D TIs and magnetic Chern insulators. In the above discussion of Eq. (1) and throughout the remainder of this work, we have defined the BZ boundary as the set of momentum-space surfaces for which ***k*** ⋅ ***b***_*i*_ = *π*, where ***b***_*i*_ is a primitive reciprocal lattice vector.

In addition to crystal defects, static magnetic flux has also been proposed as a probe of bulk topology^6,27,31–33^. For example, static *π*-flux cores in Chern insulators (2D TIs) have been shown to bind 0D solitons with *e*/2 charge (spin-charge separation), where we have defined all charges with respect to the point of charge neutrality. In 3D TIs and magnetic axion insulators (AXIs)^4–6,15–17,19,23,34–38^, *π*-flux tubes provide a means of probing the topologically-quantized bulk magnetoelectric polarizability. Specifically, in 3D TIs, a pair of *π*-flux tubes will bind a pair of "wormhole-like” helical modes (subdivided into one pair of helical modes per tube)^33^. If ${{{{{{{\mathcal{T}}}}}}}}$ is relaxed in a manner that preserves the quantized bulk axion angle *θ* = *π*, the 3D TI is converted into a magnetic AXI, and the flux-tube helical modes will become gapped and leave behind anomalous ± *e*/2 end charges, one at one end of each flux tube, in a manifestation of the axionic magnetoelectric effect. Specifically, the topological axion angle *θ* = *π* is the coefficient of the magnetoelectric response ***E***_e_ ⋅ ***B***_e_, where ***E***_e_ and ***B***_e_ are the electric and magnetic fields, respectively. Hence, the *e*/2 end charges bound to *π*-flux tubes in an AXI represent signatures of the quantized bulk magnetoelectric polarizability (nontrivial axion angle), because the external magnetic field has induced a quantized electric polarization aligned with the magnetic field.

In recent years, the set of topologically nontrivial 2D and 3D insulating phases has been greatly extended beyond TIs, Chern insulators, and AXIs by incorporating the constraints imposed by crystalline symmetry on electronic band structures^18,20^. Recently introduced symmetry-protected 2D topological insulating phases include 2D TCIs with mirror-protected edge states^8,39–41^, as well as fragile TIs (FTIs)^17,23,39,42–45^ and 2D obstructed atomic limits (OALs)^15,17,18,23,39,46,47^ with 0D fractionally charged or spin-charge-separated corner states. In 3D, TCI phases with gapped 2D surfaces and gapless 1D hinges have recently been discovered, and have become known as higher-order TIs (HOTIs)^15–17,20–23,35,37,46^. After the discovery of higher-order topology, earlier examples of magnetic AXIs were recognized to in fact be magnetic chiral HOTIs^17,37^. In an AXI, each surface exhibits an odd number of massive or massless twofold Dirac cones corresponding to an anomalous half-integer surface Hall conductivity, and domain walls between gapped surfaces with differing half-integer Hall conductivities bind chiral hinge modes^17,19,35–37^.

${{{{{{{\mathcal{T}}}}}}}}$-symmetric HOTI phases with helical hinge modes have also been predicted in rhombohedral bismuth crystals^48^, the transition metal dichalcogenides MoTe_2_ and WTe_2_^23,49^, and BiBr^12,13,49^. Through scanning tunneling microscopy (STM) and quantum oscillation experiments, incipient support for the existence of helical hinge states was subsequently reported in the aforementioned candidate HOTIs bismuth^48^, MoTe_2_^50,51^, WTe_2_^52^, and BiBr^53,54^. However, the experimental data attributed to helical higher-order topology has also attracted alternative explanations^30,55,56^. Unlike AXIs, ${{{{{{{\mathcal{T}}}}}}}}$-symmetric helical HOTIs exhibit trivial axion angles $\theta \,{{{{{{{\rm{mod}}}}}}}}\ 2\pi=0$ and are therefore non-axionic. To date, there does not yet exist a *θ*-like bulk topological field theory for non-axionic HOTIs to provide clarity for the experimental data^17,19,57^.

In this work, we present novel defect and static flux response effects in 3D insulators, which provide experimentally observable signatures of fragile and non-axionic higher-order topology in solid-state materials (see Table 1). We begin below by reviewing spin-charge separation in non-interacting electronic materials. We then introduce a more general formulation of Eq. (1) that captures the dislocation bound states of all possible topologically nontrivial insulating phases, including FTIs and OALs; this formulation is based on a mapping from (*d*−1)-dimensional [(*d*−1)-D] subspaces of the BZ to (*d*−1)-D real-space surfaces in *d*-D crystals with (*d*−2)-D defects. Next, we show that our extended formulation of topological defect response captures all previously identified topological electronic crystal dislocation states and reveals the existence of higher-order end (HEND) states bound to the surface and corner terminations of screw and edge dislocations in FTIs, OALs, and HOTIs [see Supplementary Note (SN) 4 for numerical defect-state calculation details]. We analytically and numerically demonstrate that 0D HEND states are equivalent to the fractionally charged or spin-charge-separated corner states of 2D FTIs and OALs, and are anomalous, intrinsic consequences of the bulk electronic structure. Using tight-binding calculations (detailed in SN 4), we specifically demonstrate the presence of topological HEND states in 3D HOTIs and weak FTIs driven by double band inversion^23,43,48^ on the BZ boundary. Lastly, we use density functional theory (DFT) to demonstrate the presence of intrinsic HEND corner states on edge dislocation networks in the 3D TCI^8^ and HOTI^16^ SnTe (SN 6B). Following our crystal-defect calculations, we next extend the TI and TCI magnetic flux-threading analyses in refs. 6, 27, 31–33 to ${{{{{{{\mathcal{T}}}}}}}}$-symmetric helical HOTIs. Below and in SN 2A3, 2B2, and 5, we first reproduce the earlier results of refs. 6, 27, 31–33 by analytically and numerically demonstrating the static *π*-flux response of 2D TIs and Chern insulators, as well as 3D AXIs. We then demonstrate the existence of a novel quantized *π*-flux response in ${{{{{{{\mathcal{I}}}}}}}}$- and ${{{{{{{\mathcal{T}}}}}}}}$-symmetric HOTIs. Specifically, we show that a pair of static *π*-flux tubes in an ${{{{{{{\mathcal{I}}}}}}}}$- and ${{{{{{{\mathcal{T}}}}}}}}$-symmetric HOTI together binds an odd (anomalous) number of chargeless spinons per surface at a half system filling, suggesting that the bulk exhibits a novel form of quantized "magneto-spinon polarizability” (MSP). Because a half-filled pair of fluxes in an isolated 2D TI binds an even number of chargeless spinons, then our results further imply that each gapped surface of an ${{{{{{{\mathcal{I}}}}}}}}$- and ${{{{{{{\mathcal{T}}}}}}}}$-symmetric HOTI is topologically equivalent to "half” of a 2D TI. We conclude by discussing experimental venues for observing the HEND states and response effects introduced in this work.

**Table 1:** Summary of dislocation- and flux-state responses derived in this work

Summary of higher-order dislocation- and flux-state responses in inversion-symmetric 3D insulators			
	Fragile topological insulators and obstructed atomic limits	Magnetic axion insulators (AXIs)	Helical higher-order topological insulators (HOTIs)
Integer dislocation	Nontrivial if and only if: ${{{{{{{\boldsymbol{B}}}}}}}}\cdot {{{{{{{{\boldsymbol{M}}}}}}}}}_{\nu }^{{{{{{{{\rm{F}}}}}}}}}\,{{{{{{{\rm{mod}}}}}}}}\ 2\pi=\pi$	Nontrivial if and only if: ${{{{{{{\boldsymbol{B}}}}}}}}\cdot {{{{{{{{\boldsymbol{M}}}}}}}}}_{\nu }^{{{{{{{{\rm{F}}}}}}}}}\,{{{{{{{\rm{mod}}}}}}}}\ 2\pi=\pi$	Nontrivial if and only if: ${{{{{{{\boldsymbol{B}}}}}}}}\cdot {{{{{{{{\boldsymbol{M}}}}}}}}}_{\nu }^{{{{{{{{\rm{F}}}}}}}}}\,{{{{{{{\rm{mod}}}}}}}}\ 2\pi=\pi$
*π*-Flux tube	Trivial	Nontrivial, signature of surface half quantum Hall state, bulk magnetoelectric polarizability	Nontrivial, signature of surface half quantum spin Hall state, bulk magneto-spinon polarizability (MSP)

We have uncovered a new bulk weak topological index ${{{{{{{{\boldsymbol{M}}}}}}}}}_{\nu }^{{{{{{{{\rm{F}}}}}}}}}$ [detailed in the Methods section and in Supplementary Notes (SN) 2A1, 2A2, 2B1, 2B3, and 3B] that indicates whether integer dislocations with Burgers vector ***B*** in an inversion-symmetric 3D insulator bind anomalous 0D states on their ends and corners, which we term higher-order end (HEND) states. While ${{{{{{{{\boldsymbol{M}}}}}}}}}_{\nu }^{{{{{{{{\rm{F}}}}}}}}}$ can be nontrivial in stable topological crystalline insulators (TCIs) with 1D hinge modes [e.g. magnetic axion insulators (AXIs)^4–6,17,19,34–38^ and helical higher-order topological insulators (HOTIs)^15,16,20–23,46^], ${{{{{{{{\boldsymbol{M}}}}}}}}}_{\nu }^{{{{{{{{\rm{F}}}}}}}}}$ can also be nontrivial in insulators with less robust forms of topology, such as fragile topological insulators^42–45^ and obstructed atomic limits^18,39,47^. Integer dislocations therefore do not provide an unambiguous probe of bulk higher-order topology. Through first principles and tight-binding calculations, we further demonstrate a nontrivial HEND-state dislocation response in the 3D TCI^8^ and HOTI SnTe^16^ (see the Methods section and SN 6B for calculation details). We have also studied the related problem of magnetic *π*-flux insertion in 3D insulators. For AXIs, *π*-flux tubes are known to provide probes of the anomalous half quantum Hall states on gapped 2D surfaces, as well as the bulk topological magnetoelectric response^6,33^. For helical HOTIs, we find that *π*-flux tubes reveal previously unrecognized bulk and surface topological features similar to those of AXIs, including surface halves of time-reversal-symmetric quantum spin Hall states, and a spin-charge-separated variant of the bulk axionic magnetoelectric effect.

## Results

### Review of spin-charge separation without interactions

Throughout this work, we will demonstrate the existence of 0D defect and flux bound states with spin-charge separation in non-interacting insulating crystals. Hence, before discussing defect and flux states in 2D and 3D insulators, we will briefly review spin-charge separation in non-interacting ${{{{{{{\mathcal{T}}}}}}}}$- and spin-rotation- [SU(2)-] invariant systems as a generalization of the familiar charge (fermion number) fractionalization previously discussed by Jackiw, Rebbi, Goldstone, and Wilczek^17,34,39,58,59^.

**Fig. 1:**
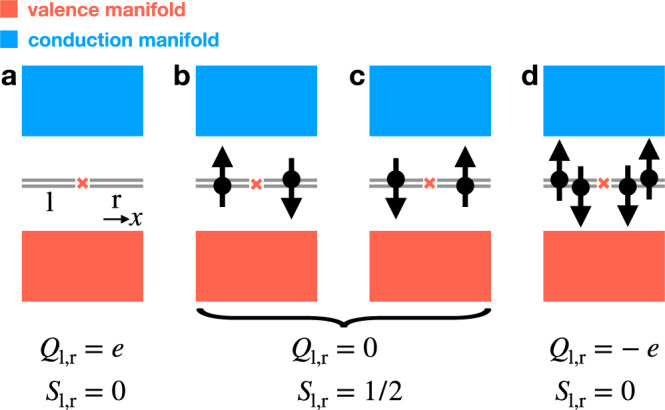
Spin-charge-separated Kramers pairs of defect or flux states. **a–d** An inversion- (${{{{{{{\mathcal{I}}}}}}}}$-) related pair of Kramers pairs of 0D defect or flux states in a spinful, time-reversal- (${{{{{{{\mathcal{T}}}}}}}}$-) symmetric insulator (where the ${{{{{{{\mathcal{I}}}}}}}}$ center is represented with a red × symbol in **a–d**). **b**, **c** When the Fermi level lies at charge neutrality, each Kramers pair is filled by only a single electron and therefore carries an excess chargeless spin-1/2 moment (*Q* = 0, *S* = 1/2). Hence at half filling, and taking the spins of the electrons occupying each pair of states to point in opposite directions, ${{{{{{{\mathcal{I}}}}}}}}$ (which relates the positions of the Kramers pairs) and ${{{{{{{\mathcal{T}}}}}}}}$ symmetries are "softly'' broken^17,34,39,59^, and each half-filled Kramers pair of states forms an effective spinon quasiparticle with a free-angle spin-1/2 moment (depicted in **b**, **c** in configurations that preserve ${{{{{{{\mathcal{I}}}}}}}}\times {{{{{{{\mathcal{T}}}}}}}}$ symmetry). By **a** removing or **d** adding two electrons to the system (one electron per Kramers pair), we may realize a system configuration in which each Kramers pair respectively carries a net charge of ± *e* (taking electrons to carry a charge −*e*), but carries a net-zero spin (*Q* = ± *e*, *S* = 0). Hence, each Kramers pair of states either carries chargeless spin or spinless charge and therefore exhibits the same reversed spin-charge relations as the solitons in polyacetylene^60^.

We begin by considering two ${{{{{{{\mathcal{I}}}}}}}}$-related pairs of topological defects or flux tubes in a 2D or 3D insulator that each bind a pair of 0D states (four degenerate single-particle states in total), taking each pair of states to be half-filled at charge neutrality (Fig. 1b, c). The arguments below do not depend on whether the twofold degeneracy of each pair of states is enforced by spinful ${{{{{{{\mathcal{T}}}}}}}}$ or SU(2) symmetry, and therefore for simplicity, we will focus on the case in which the two states within each pair are time-reversal (Kramers) pairs. Enforcing ${{{{{{{\mathcal{I}}}}}}}}\times {{{{{{{\mathcal{T}}}}}}}}$ symmetry (where we have denoted a global ${{{{{{{\mathcal{I}}}}}}}}$ center with a red × symbol in Fig. 1), there is one filled state per Kramers pair. Hence, each Kramers pair carries a balanced (net-zero) charge with respect to charge neutrality, but necessarily "softly” breaks ${{{{{{{\mathcal{T}}}}}}}}$ symmetry, because each pair of states is filled with an unpaired spin-1/2 degree of freedom. We emphasize that without a spin conservation symmetry such as *s*^*z*^, however, each unpaired electron is not required to exhibit a quantized spin projection along a particular high-symmetry axis.

Next, if the system is doped away from charge neutrality by adding two more electrons, ${{{{{{{\mathcal{T}}}}}}}}$ and global ${{{{{{{\mathcal{I}}}}}}}}$ symmetries can conversely be satisfied individually (Fig. 1d). In the system configuration with two extra electrons, each fully filled Kramers pair of states carries a charge −*e* (taking electrons to have charge −*e*). Unlike in the previous system configuration with chargeless spin-1/2 0D states at zero doping depicted in Fig. 1b, c, at a system doping of −2*e*, each Kramers pair of states is charged, but exhibits a net-zero spin, because ${{{{{{{\mathcal{T}}}}}}}}$ [or SU(2)] symmetry pairs electrons with reversed spins. Similarly, if we remove one electron from each Kramers pair of states in Fig. 1b, c, then we realize a system configuration in which there is a total charge of +2*e*, implying that each fully empty pair of states carries a charge +*e* and does not carry an electron spin (Fig. 1a). Hence, the 0D Kramers pairs of states exhibit the same well-established spin-charge separation and reversed spin-charge relations as the solitons in polyacetylene^60^.

### Defect response of inversion-symmetric 2D insulators

In this work, we rigorously establish a prescription for identifying insulators that bind anomalous 0D defect states as a consequence of the bulk topology. We will first here numerically demonstrate that ${{{{{{{\mathcal{I}}}}}}}}$-symmetric 2D insulators with band inversion at high-symmetry points on the 2D BZ boundary exhibit a nontrivial dislocation response. We will then bolster the numerical results through first-principles and tight-binding calculations demonstrating a nontrivial first-order defect response in PbTe monolayers (see the Methods section and SN 6A for calculation details).

**Fig. 2:**
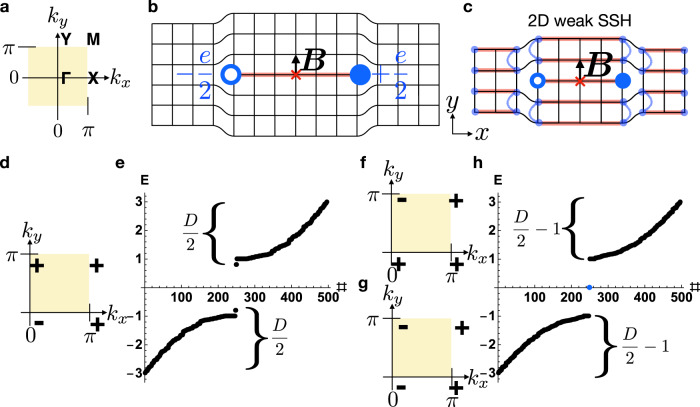
First-order 0D dislocation states in 2D crystals from 1D polarization topology. **a** The bulk Brillouin zone (BZ) of a 2D rectangular magnetic crystal with only ${{{{{{{\mathcal{I}}}}}}}}$ symmetry. **b** An ${{{{{{{\mathcal{I}}}}}}}}$-related pair of 0D dislocations with Burgers vector ${{{{{{{\boldsymbol{B}}}}}}}}=\hat{y}$ in an ${{{{{{{\mathcal{I}}}}}}}}$-symmetric crystal, where the global ${{{{{{{\mathcal{I}}}}}}}}$ center is represented with a red × symbol. **d–h** Bulk parity (${{{{{{{\mathcal{I}}}}}}}}$) eigenvalues and periodic-boundary-condition (PBC) energy spectra for the defect in **b** when the bulk is equivalent to **d** a ∣*C*∣ = 1 Chern insulator with band inversion at Γ, **f** a ∣*C*∣ = 1 Chern insulator with band inversion at *Y*, **g** a weak *y*-directed array **c** of *x*-directed Su–Schrieffer–Heeger (SSH) chains^60^. Anomalous 0D defect states ***h*** with charge ± *e*/2 are present in cases **f**, **g**, but not **d**, which instead exhibits the trivial PBC spectrum in **e** [Eq. (2)]. Specifically, the spectrum in **e** may be deformed to that of a trivial insulator (i.e. a finite-sized insulator without midgap 0D states or without an imbalance in the number of states above or below the gap) without breaking ${{{{{{{\mathcal{I}}}}}}}}$ symmetry or closing the bulk gap, whereas the spectrum in **h** cannot. Hence, as defined in refs. 17, 39, 47, the midgap dislocation states in **h** are filling-anomalous. Next, by considering the limit in **c** in which the bulk is equivalent to a decoupled array of SSH chains, we find that the two dislocations correspond to the ends of a "leftover'' SSH chain that is decoupled from the bulk. This implies that the ± *e*/2-charged defect states are equivalent to the end states of an ${{{{{{{\mathcal{I}}}}}}}}$-symmetric SSH chain^60^ (red line in **b**), and thus persist under the relaxation of particle-hole symmetry^17,34,39,59^. The explicit details of the numerical calculations shown in this figure are provided in SN 4A1.

We begin by considering a simple magnetic 2D insulator with only rectangular lattice translations *T*_*x*,*y*_ and ${{{{{{{\mathcal{I}}}}}}}}$ symmetry, such that the system respects the symmetries of magnetic layer group $p\bar{1}$^19,39^ (Fig. 2, numerical details provided in SN 4A1). We consider the case in which the pristine crystal is initially furnished with a single occupied, uncoupled, spinful *s* orbital and a single unoccupied, uncoupled, spinful *p* orbital – both at the origin of each unit cell. This implies that initially, the electronic structure at each ${{{{{{{\mathcal{I}}}}}}}}$-invariant crystal momentum (TRIM point) consists of one occupied state with a positive parity (${{{{{{{\mathcal{I}}}}}}}}$) eigenvalue and one unoccupied state with a negative parity eigenvalue^18,19^.

Next, by tuning model parameters to invert the bands at different TRIM points (Fig. 2a), we may realize several different insulating phases. When only one of the parity (${{{{{{{\mathcal{I}}}}}}}}$) eigenvalues of the occupied band is negative, the bulk is a symmetry-indicated Chern insulator with Chern number $C\,{{{{{{{\rm{mod}}}}}}}}\ 2=1$^19^. In Fig. 2d (Fig. 2f), we show the occupied parity eigenvalues of a ∣*C*∣ = 1 Chern insulator driven by band inversion at Γ (*Y*). Inserting a pair of dislocations with Burgers vector ${{{{{{{\boldsymbol{B}}}}}}}}=\hat{y}$ that preserves global ${{{{{{{\mathcal{I}}}}}}}}$ symmetry (Fig. 2b) and calculating the energy spectrum of the corresponding tight-binding model with periodic boundary conditions (PBC), we observe a pair of anomalous midgap states with charges ± *e*/2^17,34,39,59,60^ for the parity eigenvalue in Fig. 2f, but not for the parity eigenvalues in Fig. 2d, reproducing the conclusions of refs. 26, 27. Specifically, the spectrum in Fig. 2e is the same as that of a trivial (uninverted) insulator with two ${{{{{{{\mathcal{I}}}}}}}}$-related point dislocations. On the other hand, the spectrum in Fig. 2h cannot be symmetrically deformed into the spectrum of an ${{{{{{{\mathcal{I}}}}}}}}$-symmetric trivial insulator with two point dislocations. Hence, as defined in refs. 17, 39, 47, the midgap dislocation states in Fig. 2h are filling-anomalous. Throughout this work, we will use PBC and filling anomalies to numerically identify topologically nontrivial 0D defect- and flux-state responses in insulating crystals with ${{{{{{{\mathcal{I}}}}}}}}$ or ${{{{{{{\mathcal{I}}}}}}}}$ and ${{{{{{{\mathcal{T}}}}}}}}$ symmetries.

To understand the pattern of dislocation responses for the Chern insulators in Fig. 2d, f, we next form a new insulator that is equivalent to a weak, *y*-directed array of *x*-directed, ${{{{{{{\mathcal{I}}}}}}}}$-symmetric Su–Schrieffer–Heeger (SSH) chains^60^ (Fig. 2c, g); we observe that ${{{{{{{\boldsymbol{B}}}}}}}}=\hat{y}$ dislocations in this Wannierizable^18,19^ (*C* = 0) insulator also bind ± *e*/2 charges. By analogy to the weak TI discussion in ref. 24, the center red line in the weak SSH array in Fig. 2c represents a "leftover” SSH chain that may be adiabatically decoupled from the bulk crystal and binds ± *e*/2 charges on its ends, the dislocations. The results of Fig. 2d–h can be summarized by defining a weak polarization invariant ${{{{{{{{\boldsymbol{M}}}}}}}}}_{\nu }^{{{{{{{{\rm{SSH}}}}}}}}}=\pi ({n}_{XM},{n}_{YM})$, where *n*_*ab*_ is the ${{\mathbb{Z}}}_{2}$ SSH polarization invariant of the occupied bands along the BZ-edge line *ab*, such that for example, the index *n*_*XM*_ is nontrivial for *y*-directed SSH chains (see SN 3A). Analogously to the weak-index vectors of ${{{{{{{\mathcal{T}}}}}}}}$-symmetric 3D insulators^4^, ${{{{{{{{\boldsymbol{M}}}}}}}}}_{\nu }^{{{{{{{{\rm{SSH}}}}}}}}}$ can only realize values equal to half-integer linear combinations of 2D reciprocal lattice vectors. For the insulators in Fig. 2d, f, g, ${{{{{{{{\boldsymbol{M}}}}}}}}}_{\nu }^{{{{{{{{\rm{SSH}}}}}}}}}=(0,0),$ (0, *π*), and (0, *π*), respectively. Hence, for magnetic 2D insulators with ${{{{{{{\mathcal{I}}}}}}}}$ symmetry and integer Burgers vectors ***B***, we conclude that dislocations bind anomalous ±*e*/2 charges if and only if:

2 $${{{{{{{\boldsymbol{B}}}}}}}}\cdot {{{{{{{{\boldsymbol{M}}}}}}}}}_{\nu }^{{{{{{{{\rm{SSH}}}}}}}}}\,{{{{{{{\rm{mod}}}}}}}}\ 2\pi=\pi,$$

in direct analogy to Eq. (1).

In SN 3A, we additionally extend Eq. (2) to ${{{{{{{\mathcal{I}}}}}}}}$- and ${{{{{{{\mathcal{T}}}}}}}}$-symmetric 2D insulators by instead computing the BZ-boundary weak time-reversal (partial) polarization indices, which reduce to the polarization per spin sector in the limit of *s*^*z*^-spin conservation symmetry^61^. For ${{{{{{{\mathcal{I}}}}}}}}$- and ${{{{{{{\mathcal{T}}}}}}}}$-symmetric 2D insulators with nontrivial ${{{{{{{{\boldsymbol{M}}}}}}}}}_{\nu }^{{{{{{{{\rm{SSH}}}}}}}}}$ vectors, we show in SN 3A and 4B1 that dislocations satisfying Eq. (2) bind spin-charge-separated 0D solitons, rather than ±*e*/2 charges.

**Fig. 3:**
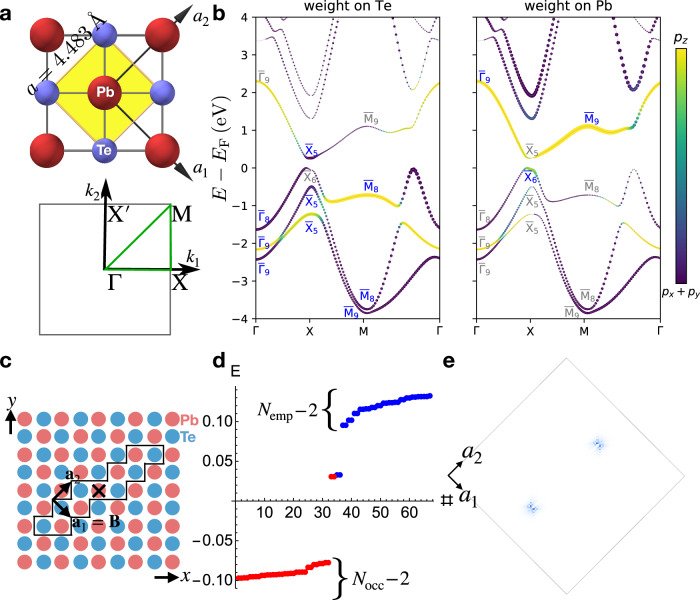
First-order dislocation states in 2D PbTe monolayers. **a** The crystal structure of monolayer PbTe and the bulk BZ. The yellow diamond in **a** indicate the primitive cell. A PbTe monolayer^40,41^ has fourfold rotation, ${{{{{{{\mathcal{I}}}}}}}}$, and mirror symmetries (layer group $p4/mmm1^{\prime}$^39^). **b** Band structure of a PbTe monolayer along the high-symmetry lines of the 2D BZ in **a**. The size of the circle at each plotted point in **b** indicates the spectral weight on the Te (left panel) and Pb (right panel) atoms, and the color bars indicate the orbital character of each Bloch state on a scale of *p*_*z*_ or *p*_*x*,*y*_ orbitals. We have additionally labeled the irreducible small corepresentations at the symmetry-independent TRIM points in the BZ in **a** (see SN 6A for details). The bands in **b** are inverted at *X* and $X^{\prime}$, driving the bulk into a 2D mirror TCI phase^40,41^ with mirror Chern number ${C}_{{M}_{z}}=2$ and nontrivial weak (partial) SSH indices ${{{{{{{{\boldsymbol{M}}}}}}}}}_{\nu }^{{{{{{{{\rm{SSH}}}}}}}}}=({{{{{{{{\boldsymbol{b}}}}}}}}}_{1}+{{{{{{{{\boldsymbol{b}}}}}}}}}_{2})/2$ (see SN 3A and 6A). **c** Schematic of our real-space implementation of an ${{{{{{{\mathcal{I}}}}}}}}$-related pair of ***B*** = ***a***_1_ point dislocations in a Wannier-based tight-binding model of a PbTe monolayer obtained from first-principles calculations (details provided in SN 6A), where the ${{{{{{{\mathcal{I}}}}}}}}$ center is marked with a black × symbol. In **c**, the sites enclosed within the black line have been removed to implement the pair of point dislocations. **d** PBC energy spectrum of a tight-binding model of PbTe with the ${{{{{{{\mathcal{I}}}}}}}}$-related pair of ***B*** = ***a***_1_ point dislocations shown in **c**; there are four midgap, filling-anomalous^17,23,39,47^ dislocation states, consistent with Eq. (2) [${{{{{{{\boldsymbol{B}}}}}}}}\cdot {{{{{{{{\boldsymbol{M}}}}}}}}}_{\nu }^{{{{{{{{\rm{SSH}}}}}}}}}\,{{{{{{{\rm{mod}}}}}}}}\ 2\pi=\pi$]. **e** The real-space localization of the four midgap states in **d**, which subdivide into two ${{{{{{{\mathcal{I}}}}}}}}$-related Kramers pairs. One Kramers pair of states is localized on each dislocation core and corresponds when half-filled to a chargeless, spin-1/2 quasiparticle (i.e. a spinon) that is equivalent to the end state of a spinful SSH chain.

To further confirm Eq. (2) and its ${{{{{{{\mathcal{T}}}}}}}}$-invariant extension, we have performed first-principles calculations of the electronic structure of a PbTe monolayer^40,41^ (layer group $p4/mmm1^{\prime}$) [Fig. 3a]. The lattice vectors of a PbTe monolayer are given by

3 $${{{{{{{{\boldsymbol{a}}}}}}}}}_{1}=(1/2,-1/2),\,{{{{{{{{\boldsymbol{a}}}}}}}}}_{2}=(1/2,1/2),$$

and the reciprocal lattice vectors are given by:

4 $${{{{{{{{\boldsymbol{b}}}}}}}}}_{1}=2\pi (1,-1),\,{{{{{{{{\boldsymbol{b}}}}}}}}}_{2}=2\pi (1,\, 1).$$

Previous works^40,41^ have demonstrated that PbTe monolayers are mirror-Chern ${C}_{{M}_{z}}=2$ TCIs driven by band inversions at the *X* [***k***_*X*_ = ***b***_1_/2] and $X^{\prime}$ [${{{{{{{{\boldsymbol{k}}}}}}}}}_{X^{\prime} }={{{{{{{{\boldsymbol{b}}}}}}}}}_{2}/2$] TRIM points [Fig. 3b]. Computing the weak partial polarization indices along *XM* and $X^{\prime} M$, we determine that PbTe monolayers carry a nontrivial dislocation response vector:

5 $${{{{{{{{\boldsymbol{M}}}}}}}}}_{\nu }^{{{{{{{{\rm{SSH}}}}}}}}}=({{{{{{{{\boldsymbol{b}}}}}}}}}_{1}+{{{{{{{{\boldsymbol{b}}}}}}}}}_{2})/2,$$

where the details of our calculation are provided in SN 6A.

To probe the dislocation response, we next construct a Wannier-based tight-binding model of a PbTe monolayer and insert an ${{{{{{{\mathcal{I}}}}}}}}$-related pair of ***B*** = ***a***_1_ point dislocations, as shown in Fig. 3c. In the dislocation geometry with PBC, the energy spectrum is filling-anomalous (Fig. 3d), with each dislocation binding a Kramers pair of states (Fig. 3e) where, at half filling, each pair carries a net-zero charge and a free-angle ∣***S***∣ = 1/2 spin moment (i.e. a spinon). Hence, the Kramers pairs of dislocation bound states in PbTe monolayers are equivalent to the spin-charge-separated end states of a spinful SSH chain^60,61^. In summary, the appearance of filling-anomalous dislocation bound states in an ${{{{{{{\mathcal{I}}}}}}}}$-symmetric defect geometry in a PbTe monolayer provides further evidence for a first-order dislocation response in 2D insulators whose pristine electronic structure and dislocation Burgers vectors satisfy Eq. (2).

### Defect response from momentum-space band topology

We will next describe proofs—summarized in the Methods section and provided in complete detail in SN 2A1, 2A2, 2B1, and 2B3—explicitly linking the topology of pristine, insulating crystals to the electronic states bound to dislocations. In this work, we specifically show that dislocations with integer Burgers vectors^1^ bind edge and corner modes deriving from the momentum-space topology of lower-dimensional surfaces of the BZs of pristine crystals. In the 3D case—which is most relevant to solid-state materials—we use this mapping to analytically demonstrate that the corners and ends of 1D edge and screw dislocations in 3D insulators can bind anomalous 0D HEND states as an intrinsic consequence of nontrivial bulk topology.

A central result of this work is the recognition that Eqs. (1) and (2) represent specific cases of a more general statement, which we will summarize below. First, for a *d*-D crystal hosting (*d*−2)-D dislocations with integer-valued Burgers vectors^25^, the exact location of the real-space (*d*−1)-D surface spanning the dislocations is a gauge-dependent quantity^1^ (it can be moved at zero energy cost and changed by redefinition), while the locations of the (*d*−2)-D dislocations are gauge-invariant, as they carry quantized and measurable Burgers vectors. Specifically, ***B*** is defined by measuring the total displacement along a loop around a dislocation; though the amount of displacement assigned to a given (*d*−1)-D surface between a pair of dislocations represents a numerical choice of gauge, the location of each dislocation and the value of the total displacement ***B*** are conversely gauge-independent. In the momentum-space *d*-D Hamiltonians of pristine insulators with the same bulk topology as the crystal with dislocations, we next consider the topology in the (*d*−1)-D BZ-boundary surface defined by the normal momentum vector ***M*** [e.g., in the *k*_*x*_ = *π* plane of a 3D insulator, ***M*** = (*π*, 0, 0)]. In this work, we find that the (*d*−1)-D position-space surface spanning a pair or closed loop of dislocations – regardless of its gauge-dependent shape—hosts the same topological boundary states as a (*d* − 1)-D crystal whose bulk topology is equivalent to that of the (*d*−1)-D BZ-boundary surface defined by ***M***, provided that two conditions are satisfied:

1. $${{{{{{{\boldsymbol{B}}}}}}}}\cdot {{{{{{{\boldsymbol{M}}}}}}}}\,{{{{{{{\rm{mod}}}}}}}}\ 2\pi=\pi$$.
2. The position-space system with dislocations preserves the same symmetries that enforce the momentum-space bulk (*d*−1)-D topology in the (*d*−1)-D BZ surface defined by ***M***.

In a weak TI^4,24^, the necessary symmetry is ${{{{{{{\mathcal{T}}}}}}}}$; however as shown in this work, the required symmetry may also be spatial (e.g. ${{{{{{{\mathcal{I}}}}}}}}$).

To reconcile our results with previous works, we have formulated two alternative and equivalent sets of proofs demonstrating the aforementioned dislocation topological mapping from momentum space to position space. Our proofs reproduce the results of all previous studies of crystal dislocation bound states with integer ***B***^24–29^. First, building upon the "cutting” and "gluing” construction of topological defect states developed in ref. 24 to predict helical dislocation modes in weak TIs^4^, we have employed *k* ⋅ *p* theory to predict HEND states in 3D crystals (see SN 2A1 and 2A2). Next, we use more general arguments to demonstrate that (*d*−2)-D dislocations in *d*-D crystals can map (*d*−1)-D BZ surfaces to (*d*−1)-D real-space surfaces, leading in 3D crystals to the presence of 1D and 0D topological defect states (see SN 2B1 and 2B3).

Through both sets of proofs, we deduce that given an ${{{{{{{\mathcal{I}}}}}}}}$-symmetric, ${{{{{{{\mathcal{T}}}}}}}}$-broken 3D insulator with vanishing weak Chern numbers^19,25,26,57^, ${{{{{{{\mathcal{I}}}}}}}}$-symmetric dislocations with Burgers vector ***B*** will bind anomalous 0D states at ${{{{{{{\mathcal{I}}}}}}}}$-related locations along the set of dislocations if and only if:

6 $${{{{{{{\boldsymbol{B}}}}}}}}\cdot {{{{{{{{\boldsymbol{M}}}}}}}}}_{\nu }^{{{{{{{{\rm{F}}}}}}}}}\,{{{{{{{\rm{mod}}}}}}}}\, 2\pi=\pi,$$

where ${{{{{{{{\boldsymbol{M}}}}}}}}}_{\nu }^{{{{{{{{\rm{F}}}}}}}}}=\pi ({\nu }_{x}^{{{{{{{{\rm{F}}}}}}}}},{\nu }_{y}^{{{{{{{{\rm{F}}}}}}}}},{\nu }_{z}^{{{{{{{{\rm{F}}}}}}}}})$ is a new weak index vector characterizing which of the BZ-boundary planes host Hamiltonians that are topologically equivalent to the ${{{{{{{\mathcal{I}}}}}}}}$-symmetric 2D FTI introduced in refs. 17, 23, or the OAL that results from adding trivial bands without anomalous corner charges to the ${{{{{{{\mathcal{I}}}}}}}}$-symmetric 2D FTI. Like the weak-index vectors of ${{{{{{{\mathcal{T}}}}}}}}$-symmetric 3D insulators^4^, ${{{{{{{{\boldsymbol{M}}}}}}}}}_{\nu }^{{{{{{{{\rm{F}}}}}}}}}$ can only realize values equal to half-integer linear combinations of 3D reciprocal lattice vectors. In SN 3B, we rigorously define ${{{{{{{{\boldsymbol{M}}}}}}}}}_{\nu }^{{{{{{{{\rm{F}}}}}}}}}$ using elementary band representations^18,19^. Heuristically, ${\nu }_{i}^{{{{{{{{\rm{F}}}}}}}}}$ is nontrivial when the Hamiltonian in the *k*_*i*_ = *π* BZ boundary plane differs by 2 + 4*n* band inversions from an ${{{{{{{\mathcal{I}}}}}}}}$-symmetric 2D trivial atomic limit (counting each state individually, as opposed to Kramers pairs of states).

Analogously to ${{{{{{{{\boldsymbol{M}}}}}}}}}_{\nu }^{{{{{{{{\rm{SSH}}}}}}}}}$ [defined in the text preceding Eq. (2)], ${{{{{{{{\boldsymbol{M}}}}}}}}}_{\nu }^{{{{{{{{\rm{F}}}}}}}}}$ can also be adapted to ${{{{{{{\mathcal{I}}}}}}}}$- and ${{{{{{{\mathcal{T}}}}}}}}$-symmetric 3D systems by analyzing nonmagnetic insulators with four band inversions (two Kramers pairs) in a BZ boundary plane. In SN 3B2 and 3B3, we respectively define the ${{{{{{{\mathcal{T}}}}}}}}$-symmetric invariant ${{{{{{{{\boldsymbol{M}}}}}}}}}_{\nu }^{{{{{{{{\rm{F}}}}}}}}}$, using elementary band representations and by introducing a nested Wilson loop formulation^15–17,23,39,46^ of partial nested Berry phase (which reduces to the nested Berry phase per spin sector in the limit of *s*^*z*^-spin conservation symmetry). As with the ${{{{{{{\mathcal{T}}}}}}}}$-symmetric generalization of ${{{{{{{{\boldsymbol{M}}}}}}}}}_{\nu }^{{{{{{{{\rm{SSH}}}}}}}}}$ discussed earlier in the context of PbTe monolayers [see Eq. (5) and the surrounding text], for ${{{{{{{\mathcal{I}}}}}}}}$- and ${{{{{{{\mathcal{T}}}}}}}}$-symmetric 3D insulators with nontrivial ${{{{{{{{\boldsymbol{M}}}}}}}}}_{\nu }^{{{{{{{{\rm{F}}}}}}}}}$ vectors, the corners of edge dislocations and the ends of screw dislocations satisfying Eq. (6) bind spin-charge-separated 0D solitons, rather than ± *e*/2 charges.

### Topological 0D defect states in 3D insulators

**Fig. 4:**
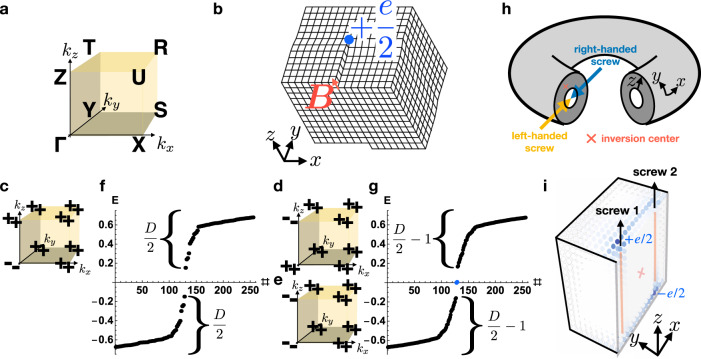
Higher-order end dislocation states in 3D crystals from 2D fragile topology. **a** The bulk BZ of a 3D orthorhombic magnetic crystal with only ${{{{{{{\mathcal{I}}}}}}}}$ symmetry. **b** A screw dislocation in an ${{{{{{{\mathcal{I}}}}}}}}$-symmetric crystal with Burgers vectors ${{{{{{{\boldsymbol{B}}}}}}}}=\hat{z}$. **c**–**g** Bulk parity (${{{{{{{\mathcal{I}}}}}}}}$) eigenvalues and hollow-doughnut-boundary-condition (HDBC) energy spectra for the defects in **b**, **h** when the bulk is topologically equivalent to ***c*** an ${{{{{{{\mathcal{I}}}}}}}}$-symmetric axion insulator (AXI)^15 -- 17,19,23,35,37^ with double band inversion at Γ, **d** an AXI with double band inversion at *Z*, and **e** a weak stack of ${{{{{{{\mathcal{I}}}}}}}}$-symmetric 2D fragile TIs (FTIs) with ± *e*/2 corner charges^17,23^. **h**, **i** The HDBC geometry is defined by imposing periodic boundary conditions in two directions (here *x* and *y*), and open boundary conditions in the remaining direction (here *z*). The screw dislocations in **h**, **i** are related by global ${{{{{{{\mathcal{I}}}}}}}}$ symmetry (red × symbol in **h**, **i**). Filling-anomalous higher-order end (HEND) states with charge ± *e*/2 (the midgap states in **g**) are present at two of the four ${{{{{{{\mathcal{I}}}}}}}}$-related ends of the two screw dislocations in **d** and **e** (the top end of screw 1 and the bottom end of screw 2 in **i**), but are absent in **c** [Eq. (6)], which instead displays the trivial HDBC spectrum in **f** (see SN 4A2 for calculation details). The ±*e*/2-charged HEND states of the insulators in **d**, **e** are equivalent to the corner modes of the 2D FTI stacked to form **e**, and thus persist under the relaxation of particle-hole symmetry^17,23,39^ (see SN 2A2, 2B3, and 4A2c). Each gapped dislocation, therefore, carries an anomalous half of the *e*/2 polarization of an isolated SSH chain in the case in which global ${{{{{{{\mathcal{I}}}}}}}}$ symmetry is enforced, as it is in our numerics for the purpose of detecting filling anomalies.

Having analytically established the existence of a new weak index for 2D fragile (and OAL) topology in 3D crystals with ${{{{{{{\mathcal{I}}}}}}}}$ symmetry—${{{{{{{{\boldsymbol{M}}}}}}}}}_{\nu }^{{{{{{{{\rm{F}}}}}}}}}$—we will now numerically confirm the presence of anomalous HEND dislocation states in 3D insulators with ***B*** and ${{{{{{{{\boldsymbol{M}}}}}}}}}_{\nu }^{{{{{{{{\rm{F}}}}}}}}}$ vectors that satisfy Eq. (6). We begin by considering a magnetic 3D insulator with only orthorhombic lattice translations *T*_*x*,*y*,*z*_ and ${{{{{{{\mathcal{I}}}}}}}}$ symmetry, such that the system respects the symmetries of magnetic space group (SG) 2.4 $P\bar{1}$^19^ (Fig. 4, numerical details provided in SN 4A2). We take the pristine crystal to initially be furnished with two occupied, uncoupled, spinful *s* orbitals and two unoccupied, uncoupled, spinful *p* orbitals—all at the origin of each unit cell. This implies that initially, the electronic structure at each TRIM point consists of two occupied states with positive parity eigenvalues and two unoccupied states with negative parity eigenvalues^18,19^.

Next, by tuning model parameters to drive double band inversions at different TRIM points^23,43,48^, we may realize several different 3D insulating phases, including chiral HOTIs (AXIs) and weak stacks of 2D FTIs. Specifically, if there is an odd total number of double band inversions (recalling that single band inversions give rise to Weyl semimetal phases^19^), and if the bulk is gapped and all weak Chern numbers vanish, then the system is an ${{{{{{{\mathcal{I}}}}}}}}$-symmetry-indicated AXI^15–17,19,23,35,37,57^. In an AXI phase, the bulk topology can generically be expressed as a pumping cycle of a 2D FTI or OAL with ±*e*/2-charged 0D corner modes, where the 3D spectral flow of each 0D corner mode manifests as a 1D chiral hinge state^17,23^. Hence, in an AXI, the weak fragile index ${{{{{{{{\boldsymbol{M}}}}}}}}}_{\nu }^{{{{{{{{\rm{F}}}}}}}}}$ indicates whether the 2D BZ planes in which the Hamiltonians characterize 2D FTIs and OALs with anomalous corner modes lie in the BZ boundary.

We next insert two ${{{{{{{\boldsymbol{B}}}}}}}}=\hat{z}$ screw dislocations of opposite chiralities (SN 2A2) at ${{{{{{{\mathcal{I}}}}}}}}$-related positions into the four-band model taken with hollow-doughnut boundary conditions (HDBC, see Fig. 4h, i) for each of the occupied parity eigenvalue configurations in Fig. 4c–e. The HDBC geometry is closely related to the "Corbino doughnut” employed in ref. 4 to characterize 3D TIs; however, in this work, we will introduce screw dislocations (and later flux tubes) in a different arrangement than in ref. 4. In Fig. 4f, g, we plot the HDBC spectra of the three insulators with the parity eigenvalues listed in Fig. 4c–e, which respectively are an AXI with ${{{{{{{{\boldsymbol{M}}}}}}}}}_{\nu }^{{{{{{{{\rm{F}}}}}}}}}={{{{{{{\boldsymbol{0}}}}}}}}$, an AXI with ${{{{{{{{\boldsymbol{M}}}}}}}}}_{\nu }^{{{{{{{{\rm{F}}}}}}}}}=\pi \hat{z}$, and a weak *z*-directed stack^45^ of an ${{{{{{{\mathcal{I}}}}}}}}$-symmetric 2D FTI, where the weak FTI stack also exhibits ${{{{{{{{\boldsymbol{M}}}}}}}}}_{\nu }^{{{{{{{{\rm{F}}}}}}}}}=\pi \hat{z}$. To draw connection with previous works, we note that the ${{{{{{{\mathcal{I}}}}}}}}$-symmetric weak FTI in Fig. 4e, when cut into a rod geometry, exhibits the same flat-band-like floating hinge states (per spin) as a spinless (spin-doubled) ${{{{{{{\mathcal{I}}}}}}}}\times {{{{{{{\mathcal{T}}}}}}}}$-symmetric 3D Stiefel-Whitney insulator^43^. In Fig. 4d, e, but not Fig. 4c, alternating ends of the screw dislocations bind filling-anomalous, ±*e*/2-charged 0D HEND states (Fig. 4i).

This result can be understood by focusing on the weak FTI stack whose occupied parity eigenvalues are shown in Fig. 4e. In the limit in which the weak FTI is adiabatically deformed into decoupled layers of 2D FTIs and the screw dislocations replaced with edge dislocations (see SN 2A1, 2B1, and 4A2c), the plane between the dislocations represents a "leftover” FTI that may be adiabatically decoupled from the bulk crystal (Fig. 4h, i), analogous to the previous "leftover” SSH chain in Fig. 2***c***. Hence, the HEND states in Fig. 4 are equivalent to the corner charges of the 2D FTI that comprises each layer of the weak stack. Furthermore, because the gapped 1D edges of 2D ${{{{{{{\mathcal{I}}}}}}}}$-symmetric FTIs carry anomalous halves of the *e*/2 polarization of an isolated SSH chain when global ${{{{{{{\mathcal{I}}}}}}}}$ symmetry is enforced^17,23^, then each of the screw dislocations in Fig. 4i carries only half of the fractionally charged end states of an isolated SSH chain.

**Fig. 5:**
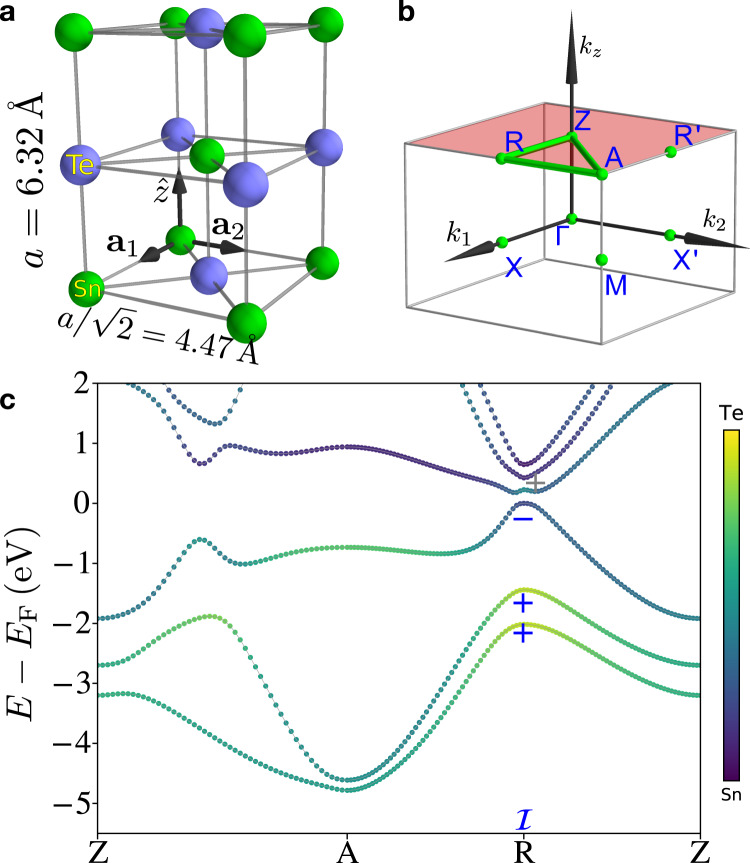
Nontrivial weak partial fragile indices in 3D SnTe. **a** Crystal structure of 3D SnTe^8,16^ in a tetragonal supercell that contains four atoms and respects the symmetries of space group 123 $P4/mmm1^{\prime}$. **b** The BZ of the tetragonal supercell in the left panel of (**a**). **c** The first-principles electronic structure of SnTe plotted along the path indicated in **b** with a green line (see SN 6B1 for calculation details). The bands in **c** exhibit a fourfold degeneracy at all ***k*** points due to the combined effects of spinful ${{{{{{{\mathcal{I}}}}}}}}\times {{{{{{{\mathcal{T}}}}}}}}$ symmetry and supercell BZ folding. We have specifically employed a supercell geometry that preserves the primitive lattice translation symmetries of SnTe in order to simplify the system geometry when dislocations are inserted. Bands in the *k*_*z*_ = *π* plane are hence fourfold degenerate due to band backfolding and ${{{{{{{\mathcal{I}}}}}}}}\times {{{{{{{\mathcal{T}}}}}}}}$ symmetry. However, the tetragonal supercell only represents a choice of convention and does not affect the generalization of our results to real SnTe crystals, which are face-centered cubic^8,16^. The ± signs in **c** denote the parity eigenvalues per Kramers pair of the Bloch states at the TRIM point *R* [***k***_*R*_ = ***b***_1_/2, see Eq. (8) for the definitions of ***b***_1,2,3_]. The band structure in **c** indicates that SnTe differs from an unobstructed atomic limit [that is topologically equivalent to 3D PbTe, see ref. 8 and SN 6A] by double band inversions at the *R* and $R^{\prime}$ points [${{{{{{{{\boldsymbol{k}}}}}}}}}_{R^{\prime} }={{{{{{{{\boldsymbol{b}}}}}}}}}_{2}/2$] in the tetragonal supercell between two pairs of Kramers pairs of states with opposite parity eigenvalues [four valence bands and four conduction bands become inverted at both *R* and $R^{\prime}$]. The four band inversions drive the bulk into a fourfold "rotation-anomaly'' TCI phase^11,16,21^ with a nontrivial weak (partial) fragile index vector ${{{{{{{{\boldsymbol{M}}}}}}}}}_{\nu }^{{{{{{{{\rm{F}}}}}}}}}=({{{{{{{{\boldsymbol{b}}}}}}}}}_{1}+{{{{{{{{\boldsymbol{b}}}}}}}}}_{2})/2$ [see SN 3B and 6B1 and the text surrounding Eq. (6)].

To provide further support for the HEND-state response introduced in this work [Eq. (6)], we will next demonstrate the presence of anomalous HEND states on the corners of edge dislocations with the shortest possible integer Burgers vectors in 3D SnTe crystals. Through first-principles calculations detailed in the Methods section and SN 6B, we find in this work that 3D SnTe—a well-established fourfold rotation-anomaly TCI with helical hinge states^8,11,16,21^—exhibits a nontrivial HEND-state response vector. SnTe crystals respect the symmetries of the face-centered-cubic space group (SG) 225 $Fm\bar{3}m1^{\prime}$. We begin by, for geometric simplicity, artificially enlarging the unit cell of SnTe into a tetragonal supercell in SG 123 $P4/mmm1^{\prime}$ [Fig. 5a] with lattice vectors given by

7 $${{{{{{{{\boldsymbol{a}}}}}}}}}_{1}=(1/2,-1/2,\, 0),\,{{{{{{{{\boldsymbol{a}}}}}}}}}_{2}=(1/2,\, 1/2,\, 0),\,{{{{{{{{\boldsymbol{a}}}}}}}}}_{3}=(0,\, 0,\, 1),$$

in units in which the lattice spacing *a* = 1, and reciprocal lattice vectors given by

8 $${{{{{{{{\boldsymbol{b}}}}}}}}}_{1}=2\pi (1,-1,\, 0),\,{{{{{{{{\boldsymbol{b}}}}}}}}}_{2}=2\pi (1,\, 1,0),\,{{{{{{{{\boldsymbol{b}}}}}}}}}_{3}=2\pi (0,\, 0,\, 1).$$

In SN 6B1, we show that 3D SnTe differs from an unobstructed atomic limit without corner or hinge states [i.e. 3D PbTe, see ref. 8 and SN 6A] by double band inversions at the *R* point [***k***_*R*_ = ***b***_1_/2] and at the symmetry-related point $R^{\prime}$ [${{{{{{{{\boldsymbol{k}}}}}}}}}_{R^{\prime} }={{{{{{{{\boldsymbol{b}}}}}}}}}_{2}/2$] between two pairs of Kramers pairs of states with opposite parity eigenvalues [four valence bands and four conduction bands become inverted at *R* and at $R^{\prime}$, see Fig. 5b, c]. The four Kramers pairs of band inversions drive SnTe into a fourfold rotation-anomaly TCI phase with a nontrivial weak (partial) fragile index vector [see SN 3B and 6B1 and the text surrounding Eq. (6)]:

9 $${{{{{{{{\boldsymbol{M}}}}}}}}}_{\nu }^{{{{{{{{\rm{F}}}}}}}}}=({{{{{{{{\boldsymbol{b}}}}}}}}}_{1}+{{{{{{{{\boldsymbol{b}}}}}}}}}_{2})/2,$$

given in terms of the tetragonal supercell reciprocal lattice vectors in Eq. (8).

**Fig. 6:**
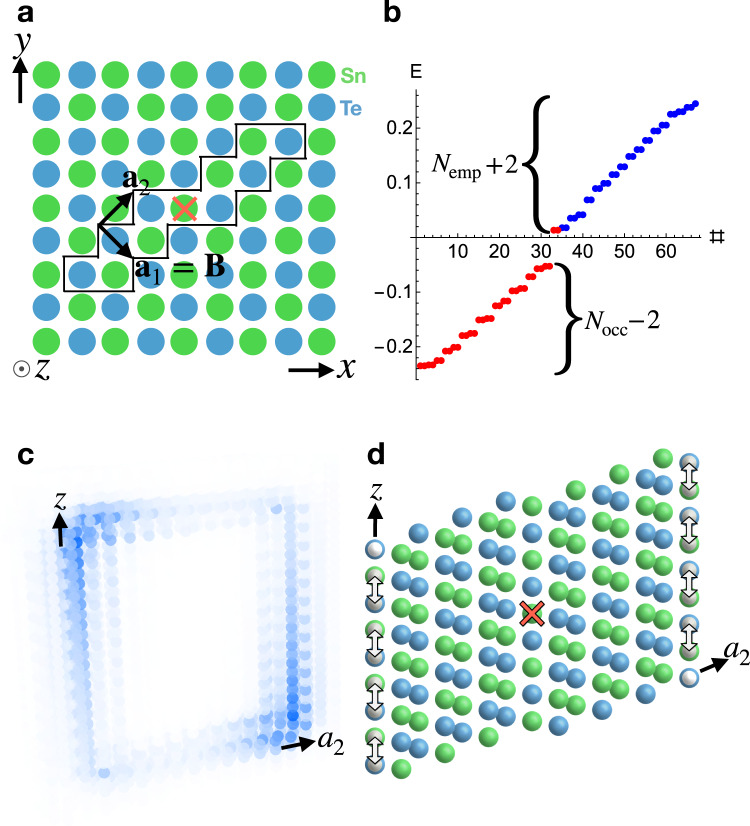
0D dislocation states in 3D SnTe crystals. **a** Defect geometry for an ${{{{{{{\mathcal{I}}}}}}}}$-related pair of internal edge dislocations with ***B*** = ***a***_1_ in 3D SnTe, where the ${{{{{{{\mathcal{I}}}}}}}}$ center is marked with a red × symbol. In **a**, the sites enclosed within the black line have been removed in a finite number of layers in the tight-binding calculation to implement the pair of edge dislocations. **b** The PBC dislocation spectrum of SnTe using the edge dislocation geometry in **a** exhibits four filling-anomalous states (two Kramers pairs), consistent with Eq. (6) [see SN 6B2 for calculation details]. **c** The real-space profile of the four anomalous states in **b**. In **c**, two total Kramers pairs of states are localized on ${{{{{{{\mathcal{I}}}}}}}}$-related dislocation corners (one Kramers pair of states is bound to every other corner). When the HEND states in **c** are half-filled, each Kramers pair corresponds to a chargeless, spin-1/2 quasiparticle (i.e. a spinon) that is equivalent to the corner state of an ${{{{{{{\mathcal{I}}}}}}}}$- and ${{{{{{{\mathcal{T}}}}}}}}$-symmetric 2D FTI (see SN 4B2 and refs. 17, 23). **d** The SnTe defect plane, for which a cross-sectional cut is enclosed by the black lines in ***a***, schematically depicted as a stack of PbTe monolayer defect lines (Fig. 3**c**, **e**). In **d**, each defect line has two 0D dislocations on its end, which each bind first-order 0D topological dislocation states. We choose PbTe for the monolayers—rather than SnTe— because a decoupled stack of PbTe monolayers has the same *x*, *y* components of the ${{{{{{{{\boldsymbol{M}}}}}}}}}_{\nu }^{{{{{{{{\rm{F}}}}}}}}}$ vector as a tetragonal supercell of 3D SnTe, whereas the interlayer coupling in realistic 3D PbTe drives additional band inversions [Eqs. (5) and (9), see Fig. 3 and SN 6 for further details]. Hence, HEND dislocation states can be considered the result of stacking and symmetrically coupling (gray arrows in **d**) an odd number of 2D monolayers that each contain first-order dislocation bound states.

To probe the HEND-state dislocation response of SnTe, we begin with the tight-binding model introduced in ref. 8, and then insert an ${{{{{{{\mathcal{I}}}}}}}}$-related pair of ***B*** = ***a***_1_ internal edge dislocations, as shown in Fig. 6a. Notably, ***a***_1_ is also a primitive lattice vector in the face-centered-cubic cell of 3D SnTe in SG 225 $Fm\bar{3}m1^{\prime}$ (see Fig. 5a). Because the Frank energy criterion^62^ for dislocation formation indicates that dislocations with larger values of ∣***B***∣ are energetically unfavorable, then dislocations with the smallest possible integer Burgers vectors—such as the ***B*** = ***a***_1_ dislocations in our calculations – may be energetically favorable and present in SnTe samples. In the dislocation geometry with PBC, the energy spectrum is filling-anomalous (Fig. 6b), with alternating dislocation corners binding Kramers pairs of spin-charge-separated HEND states (Fig. 6c, see SN 6B2 for calculation details). As discussed earlier and in SN 2A, the Kramers pairs of dislocation bound states in Fig. 6c are equivalent to the corner states of an ${{{{{{{\mathcal{I}}}}}}}}$- and ${{{{{{{\mathcal{T}}}}}}}}$-symmetric 2D FTI^17,23^, which are themselves equivalent to the end states of an ${{{{{{{\mathcal{I}}}}}}}}$- and ${{{{{{{\mathcal{T}}}}}}}}$-symmetric spinful SSH chain^60^. The appearance of filling-anomalous dislocation bound states in an ${{{{{{{\mathcal{I}}}}}}}}$-symmetric defect geometry in 3D SnTe provides further evidence for a HEND-state dislocation response in 3D insulators whose pristine electronic structure and dislocation Burgers vectors satisfy Eq. (6).

Lastly, as shown in Fig. 6d, the HEND states in SnTe can be understood as the result of stacking and pairwise coupling monolayers of 2D PbTe (Fig. 3), where each layer is shifted by (***a***_1_ + ***a***_2_)/2 with respect to the layer underneath and contains 0D dislocations with first-order dislocation bound states at the same in-plane position. In an ${{{{{{{\mathcal{I}}}}}}}}$-symmetric stack, the 0D dislocations evolve into 1D dislocations, and neighboring 0D states pairwise annihilate in an ${{{{{{{\mathcal{I}}}}}}}}$-symmetric fashion, leaving two filling-anomalous HEND states. We choose 2D PbTe for the monolayers—rather than SnTe—because the interlayer coupling in realistic 3D PbTe drives additional band inversions, whereas a tetragonal supercell of 3D SnTe has the same *x*, *y* components of the ${{{{{{{{\boldsymbol{M}}}}}}}}}_{\nu }^{{{{{{{{\rm{F}}}}}}}}}$ vector as a decoupled stack of PbTe monolayers [Eqs. (5) and (9), see SN 6 for further details]. Hence, in the same sense that a helical HOTI is equivalent to an ${{{{{{{\mathcal{I}}}}}}}}$-symmetric stack of 2D TIs (with an odd total number of layers)^16,19,21,23,48^, HEND dislocation states can be considered the result of stacking and symmetrically coupling an odd number of 2D monolayers that each contain first-order dislocation bound states. Furthermore, if an additional layer were added to the top of Fig. 6d, global ${{{{{{{\mathcal{I}}}}}}}}$ symmetry would be relaxed, but each surface would still carry only one HEND state. Hence in more realistic material geometries without global ${{{{{{{\mathcal{I}}}}}}}}$ symmetry, we more generally expect a 3D insulator with ${{{{{{{{\boldsymbol{M}}}}}}}}}_{\nu }^{{{{{{{{\rm{F}}}}}}}}}\, \ne \, {{{{{{{\boldsymbol{0}}}}}}}}$ to exhibit a random configuration of HEND states in which, on the average, every other end or corner of a dislocation satisfying Eq. (6) carries a spin-charge-separated HEND state. This is analogous to the helical hinge modes in the HOTI bismuth, which appear in STM probes on every other surface step edge, despite the absence of perfect global point group symmetries^48^.

### 0D Flux states in 3D insulators

We now shift focus to the closely related problem of static *π*-flux bound states in crystals with nontrivial band topology. As shown in several previous works^6,27,31–33^, *π*-flux cores can bind anomalous 0D solitons with the same fractional charge or spin-charge separation as the 0D HEND dislocation states introduced earlier in this work. Specifically, *π*-fluxes in Chern insulators (2D TIs) bind solitons with ± *e*/2 charge (spin-charge separation). We have numerically confirmed the static *π*-flux responses of 2D Chern insulators and TIs in SN 5A1 and 5B1, respectively.

As previously for dislocation bound states, in this work, we recognize that the anomalous 0D *π*-flux bound states in Chern insulators and 2D TIs represent specific cases of a more general phenomenon. Rather than probing the BZ-boundary topology, as is done by dislocations (see the text above, as well as SN 2A1, 2A2, 2B1, and 2B3), we find that fluxes in 2D [3D] insulators bind anomalous states deriving from the summed topologies of all BZ lines [planes]. The topological boundary states of the summed topological phase correspondingly appear at the boundary of the real-space line [plane] connecting two flux tubes. More succinctly, whereas crystal defects are sensitive to weak indices, we find that the *π*-flux response of an insulator is only sensitive to strong topological indices, in agreement with the results of previous works^25–27,31,32,63^. Crucially, although the location of the position-space line [plane] between the flux cores [tubes] is sensitive to the gauge of the electromagnetic vector potential, the locations of the anomalous states on its boundaries—the flux cores [tubes]—are gauge-independent, as the flux cores [tubes] contribute a measurable Aharonov–Bohm phase shift. Our recognition that magnetic fluxes probe bulk stable topology is supported by extensive numerical calculations (SN 5), as well as rigorous analytic proofs, which are summarized in the Methods section, and provided in complete detail in SN 2A3 and 2B2.

Our analytic calculations suggest that in 3D AXIs and HOTIs, which can respectively be represented as pumping cycles of ${{{{{{{\mathcal{T}}}}}}}}$-broken and ${{{{{{{\mathcal{T}}}}}}}}$-symmetric 2D FTIs with anomalous 0D corner states^17,23^, *π*-flux tubes will bind anomalous 0D HEND states. To confirm this result, we have respectively in SN 5A2 and 5B2 numerically computed the *π*-flux-tube responses of ${{{{{{{\mathcal{I}}}}}}}}$-symmetric AXIs and ${{{{{{{\mathcal{I}}}}}}}}$- and ${{{{{{{\mathcal{T}}}}}}}}$-symmetric helical HOTIs.

In the case of an AXI, our numerical calculations reproduce the established result that a pair of parallel *π*-flux tubes in an AXI carries a total bulk *e*/2 polarization density along the direction of the tubes^33^. This represents a signature that the bulk is a TCI with a nontrivial axion angle (magnetoelectric polarizability) *θ* = *π*, where *θ* is the coefficient of the magnetoelectric response ***E***_e_ ⋅ ***B***_e_. Specifically, the nontrivial axion angle *θ* = *π* indicates that as a flux quantum *ϕ* is adiabatically threaded from *ϕ* = 0 to 2*π* into an AXI cut into a cylindrical geometry (where the flux tube is aligned with the cylinder axis and open boundary conditions are taken in all directions), a charge ∣*e*∣ is pumped from the flux tube (*r* = 0 in cylinder coordinates) to the boundary (*r* = *R*) of the top and bottom surfaces in a manifestation of the bulk topological magnetoelectric effect^6^. This observation is consistent with the appearance in our analytic and numerical calculations of an ∣*e*∣/2-charged, anomalous midgap state bound to the end of the flux tube at the midpoint of the pumping cycle *ϕ* = *π*^6,36^. Specifically, on both the top and bottom surfaces of the cylinder (which are related by ${{{{{{{\mathcal{I}}}}}}}}$ symmetry), a charge ∣*e*∣/2 is pumped from the flux tube to the boundary, consistent with the anomalous *σ*_*xy*_ = *e*^2^/(2*h*) Hall conductivity of gapped AXI surfaces.

**Fig. 7:**
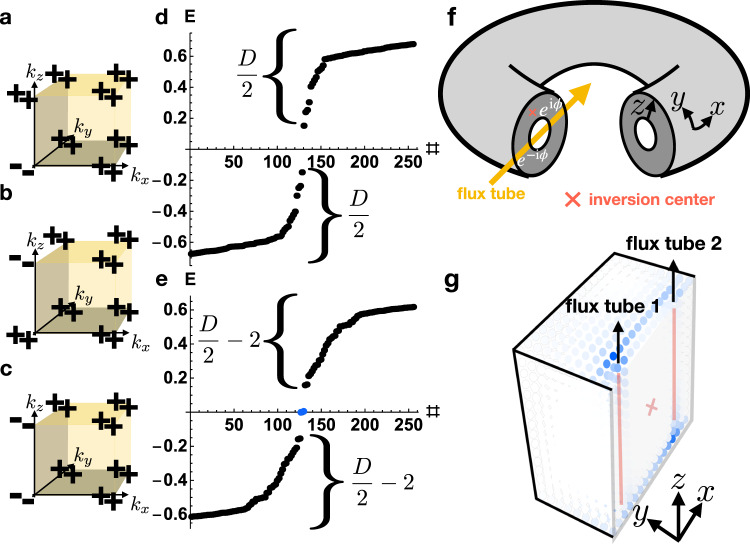
*π*-flux signatures of helical higher-order topological insulators. **a**–**c** Bulk parity eigenvalues per Kramers pair of ${{{{{{{\mathcal{I}}}}}}}}$- and ${{{{{{{\mathcal{T}}}}}}}}$-symmetric 3D insulators with four occupied bands. **a**, **b** The occupied parity eigenvalues of helical HOTIs formed from double band inversion^23,43^ about Γ and *Z*, respectively. **c** A weak stack of the ${{{{{{{\mathcal{I}}}}}}}}$- and ${{{{{{{\mathcal{T}}}}}}}}$-symmetric 2D FTI from ref. 23, which is equivalent to two superposed, ${{{{{{{\mathcal{T}}}}}}}}$-reversed copies of the magnetic 2D FTI introduced in refs. 17, 23. **f** We place the insulators in **a**–**c** in the hingeless HDBC geometry detailed in Fig. 4 and the surrounding text, and then pierce the doughnut with magnetic flux *ϕ*, creating **g** a pair of flux tubes related by global ${{{{{{{\mathcal{I}}}}}}}}$ symmetry (red × symbol in **f**, **g**). Plotting the HDBC spectra of the insulators in **a**–**c** for *ϕ* = *π* flux tubes, we observe a filling anomaly^17,23,39,47^ **e** for the helical HOTIs in cases **a**, **b** and a trivial spectrum **d** for the 3D weak FTI in case **c**. For the helical HOTIs in **a**, **b** with threaded *π*-flux tubes, **g** only one end of each *π*-flux tube binds a Kramers pair of spin-charge separated HEND states, such that each surface carries only a single Kramers pair. Per surface, this represents half of the *π*-flux response of an isolated 2D TI (see refs.^25^–^27^, ^31^, ^32^, ^63^ and SN 2A3), implying that gapped helical HOTI surfaces carry anomalous "half'' quantum spin Hall states. Furthermore, because each flux tube is equivalent to the gapped 1D edge of an ${{{{{{{\mathcal{I}}}}}}}}$- and ${{{{{{{\mathcal{T}}}}}}}}$-symmetric 2D FTI^23^, then the flux tubes each carry an anomalous half of the nontrivial partial (time-reversal) polarization of a spinful SSH chain^61^ in the case in which global ${{{{{{{\mathcal{I}}}}}}}}$ symmetry is enforced, as it is in our numerics for the purpose of detecting filling anomalies. This suggests that helical HOTIs carry a novel bulk response that represents the 3D generalization of 1D time-reversal polarization.

Returning to the HDBC geometry employed in this work, in which there are (untwisted) PBC in the directions perpendicular to the threaded magnetic flux (Fig. 7f), we note that a lattice model cannot be constructed with a *ϕ*-flux tube unless a second tube with a flux −*ϕ* is inserted elsewhere into the system. Hence, in the case numerically investigated in this work of an AXI with two threaded flux tubes and HDBC, a charge ∣*e*∣ is instead pumped from one flux tube to the other as *ϕ* is advanced from 0 to 2*π*. Lastly, we note that because there are two flux tubes with opposite fluxes ± *ϕ*, then, even if the locations of the flux tubes are related by a global ${{{{{{{\mathcal{I}}}}}}}}$ center, neither flux tube lies exactly on the global ${{{{{{{\mathcal{I}}}}}}}}$ center, as this would require the flux tubes to lie at the same position. Hence, the HDBC flux-tube geometry itself generically violates ${{{{{{{\mathcal{I}}}}}}}}$ symmetry, except at the ${{{{{{{\mathcal{I}}}}}}}}$- and ${{{{{{{\mathcal{T}}}}}}}}$-invariant flux values *ϕ* = 0, *π*.

Unlike AXIs^5,6,34,36–38,64^, ${{{{{{{\mathcal{I}}}}}}}}$- and ${{{{{{{\mathcal{T}}}}}}}}$-symmetric helical HOTIs exhibit trivial axion angles $\theta \,{{{{{{{\rm{mod}}}}}}}}\ 2\pi=0$, and it is currently unknown—and of great theoretical and experimental interest—whether there exist 3D bulk or 2D surface quantized response effects that distinguish trivial insulators from ${{{{{{{\mathcal{T}}}}}}}}$-symmetric HOTIs. In this work, we discover for the first time that *π*-flux tubes in ${{{{{{{\mathcal{I}}}}}}}}$- and ${{{{{{{\mathcal{T}}}}}}}}$-symmetric 3D HOTIs bind Kramers pairs of spin-charge-separated HEND states on only one end (Fig. 7g). Specifically, on a lattice terminated in the hingeless, ${{{{{{{\mathcal{I}}}}}}}}$-symmetric HDBC geometry in Fig. 7f, both helical HOTIs and trivial insulators (FTIs) exhibit fully gapped spectra. However, when we pierce a hollow doughnut of the topologically distinct insulators with *π*-flux tubes that preserves an ${{{{{{{\mathcal{I}}}}}}}}$ center (red × symbol in Fig. 7f, g), the HOTI exhibits a filling-anomalous^17,23,39,47^ HDBC spectrum (Fig. 7e), whereas the trivial insulator (FTI) does not (Fig. 7d). Crucially, because two *π*-flux cores threaded into an isolated 2D TI each bind a Kramers pair of states corresponding to a spin-charge-separated soliton^25–27,31,32,63^, then relaxing global ${{{{{{{\mathcal{I}}}}}}}}$ symmetry by "gluing” additional 2D TIs onto the surface does not change the number of free-angle surface spinons modulo 2 (in the case in which the system remains half-filled). Hence on each 2D surface, pairs of *π*-flux tubes bind only a single spin-charge-separated soliton between them, indicating that each gapped surface carries an anomalous "half” of the static *π*-flux response of a 2D TI. This implies that even without global ${{{{{{{\mathcal{I}}}}}}}}$ symmetry, each surface of an ${{{{{{{\mathcal{I}}}}}}}}$- and ${{{{{{{\mathcal{T}}}}}}}}$-symmetric 3D HOTI is topologically equivalent to "half” of a quantum spin Hall insulator—i.e. two ${{{{{{{\mathcal{T}}}}}}}}$-reversed copies of the anomalous half-integer quantum Hall state of a gapped AXI surface^4–6,10,17,35–37^.

To understand this result, we first recognize that the surfaces of HOTIs derive from unpaired fourfold Dirac fermions^23^, which cannot be stabilized in isolated ${{{{{{{\mathcal{T}}}}}}}}$-symmetric 2D semimetals, as discussed in SN 2A3 and ref. 10. Because each fourfold Dirac fermion in 2D, when gapped without breaking ${{{{{{{\mathcal{T}}}}}}}}$ symmetry, provides half of the contribution towards the bulk being a 2D TI or trivial insulator (i.e. a half unit of spin Hall conductivity in the limit of *s*^*z*^-spin symmetry)^2,3,65^, then the gapped 2D surface states of ${{{{{{{\mathcal{I}}}}}}}}$- and ${{{{{{{\mathcal{T}}}}}}}}$-symmetric HOTIs cannot be either 2D TIs or trivial insulators, and must instead be anomalous "halves” of a quantum spin Hall insulator. We refer to the anomalous 2D surface phase as a half-integer quantum spin Hall insulator, as opposed to half of a 2D TI (which is a more precise designation, because *s*^*z*^ spin is not generically a conserved quantity in solid-state materials with spin-orbit coupling [SOC]^2,3,61^), to draw connection with the more familiar half-integer quantum Hall insulators present on gapped AXI surfaces^4–6,17,35–37^, as well as with earlier works^66^. Specifically, the half-integer quantum spin Hall state was previously predicted to appear on the top surfaces of weak TIs^66^; however, in this work, we recognize the anomalous half-integer quantum spin Hall state to more generally manifest on all gapped surfaces of ${{{{{{{\mathcal{I}}}}}}}}$- and ${{{{{{{\mathcal{T}}}}}}}}$-symmetric HOTIs.

Unlike the surfaces of AXIs—which are physically distinguishable by their anomalous Hall conductivities^36,37^ ± *e*^2^/2*h* – it is currently unknown whether HOTI surfaces with anomalous halves of a quantum spin Hall state can similarly be distinguished in a gauge-invariant manner in the absence of *s*^*z*^-spin-conservation symmetry, both from each other and from 2D trivial insulators. However, in the artificial limit of *s*^*z*^-spin conservation symmetry, half-integer quantum spin Hall phases may straightforwardly be differentiated by the signs of their spin Hall conductivities^65,67^. Additionally, because the surface states of weak TIs and non-axionic TCIs with 2 + 4*n* twofold surface Dirac cones are equivalent to 1 + 2*n* (massive or massless) anomalous fourfold Dirac fermions upon BZ folding^10,11,21–23^, then our observation of a surface half quantum spin Hall state suggests that previous studies of Anderson localization and topological order on interacting weak TI and TCI surfaces^63,68^ should be revisited in the context of higher-order topology and crystal-symmetry-enhanced fermion doubling. Specifically, our observation of an anomalous *π*-flux response on helical HOTI surfaces implies that when the surface Dirac fermions of a TCI phase are gapped by breaking a crystal symmetry while preserving ${{{{{{{\mathcal{T}}}}}}}}$, the resulting gapped surface, despite its vanishing Hall conductivity, is not necessarily featureless, as assumed in some of the earlier literature. Lastly, because previous constructions of strongly-interacting topological phases have exploited the half-quantized surface quantum Hall effect of 3D TIs^69^, then our identification of a half-quantized surface quantum spin Hall effect in HOTIs may also provide further insight into the theoretical construction of ${{{{{{{\mathcal{T}}}}}}}}$-symmetric fractional TIs and other phases with anomalous topological order^70^.

The presence of HEND states bound to *π*-flux tubes in a helical HOTI—but not in a trivial insulator (see Fig. 7 and SN 5B2)—additionally provides the first example of a bulk response effect that distinguishes helical HOTIs from trivial insulators. Specifically, because each flux tube in Fig. 7f, g is equivalent to the gapped 1D edge of an ${{{{{{{\mathcal{I}}}}}}}}$- and ${{{{{{{\mathcal{T}}}}}}}}$-symmetric 2D FTI^23^, then, in the presence of global ${{{{{{{\mathcal{I}}}}}}}}$ symmetry, the flux tubes each carry an anomalous half of the time-reversal polarization of an isolated spinful SSH chain (SN 3B3 and ref. 61), in that each flux tube binds a spin-charge-separated Kramers pair on only one end. This implies that the bulk exhibits a novel form of quantized nontrivial MSP—a spin-charge-separated generalization of the magnetoelectric polarizability of AXIs^5,6,34,36–38,64^.

We may further understand the MSP by recognizing that an ${{{{{{{\mathcal{I}}}}}}}}$-symmetric, finite-sized sample of an ${{{{{{{\mathcal{I}}}}}}}}$- and ${{{{{{{\mathcal{T}}}}}}}}$-symmetric helical HOTI is equivalent to a stack (layer construction) of 2D TIs in which the edge states have been pairwise gapped, leaving behind sample-encircling helical hinge modes^19–22^. In the limit of *s*^*z*^-spin conservation, it has previously been established that 2D TIs in a Corbino disc geometry with adiabatically threaded magnetic flux pass a quantized spin current from the inner region to the outer region in a manifestation of the quantum spin Hall effect^2,65,67^. Hence, we can conclude that in the *s*^*z*^-conserving limit, adiabatically threading a single magnetic flux from *ϕ* = 0 to 2*π* through an ${{{{{{{\mathcal{I}}}}}}}}$- and ${{{{{{{\mathcal{T}}}}}}}}$-symmetric helical HOTI in a finite cylindrical geometry can transport a quantized amount of spin from the flux tube (*r* = 0 in cylinder coordinates) to the boundary (*r* = *R*) of the top and bottom surfaces, representing a higher-order generalization of the quantum spin Hall effect. This is consistent with the appearance in our numerical calculations of spin-charge-separated HEND states bound to one end of each flux tube at the midpoint of the pumping cycle *ϕ* = *π* (see Fig. 7f, g). It is important to note that in the absence of *s*^*z*^-spin conservation symmetry, there is no guarantee that the MSP implies a magnetic field-dependent quantized spin accumulation. We leave the exciting questions of a Berry-connection formulation of the MSP, the *θ*-like topological field theory for the MSP, and whether the MSP can be computed ab initio for future works.

### Identical *π*-flux states in topologically distinct insulators

**Fig. 8:**
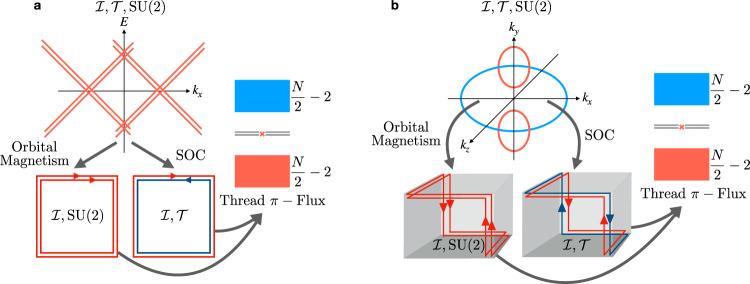
Patterns of identical static *π*-flux response in topologically distinct insulators. **a** (Top) A 2D graphene-like topological semimetal with two fourfold Dirac cones protected by ${{{{{{{\mathcal{I}}}}}}}}$, ${{{{{{{\mathcal{T}}}}}}}}$, and SU(2)-spin-rotation symmetries^71^ can gap into two topologically distinct insulators. (Bottom, left) Applying ${{{{{{{\mathcal{I}}}}}}}}$-symmetric orbital (Haldane) magnetism gaps the Dirac semimetal in **a** into a ∣*C*∣ = 2 spin-degenerate Chern insulator with ${{{{{{{\mathcal{I}}}}}}}}$ and SU(2) symmetries^72^. (Bottom, right) Conversely, ${{{{{{{\mathcal{I}}}}}}}}$-symmetric spin-orbit coupling (SOC) gaps the Dirac semimetal in **a** into a 2D TI with ${{{{{{{\mathcal{I}}}}}}}}$ and ${{{{{{{\mathcal{T}}}}}}}}$ symmetries^2,3^. (Right) However, ∣*C*∣ = 2 spin-degenerate Chern insulators and 2D TIs exhibit the same *π*-flux response. In both 2D insulators, *π*-flux cores each bind a twofold-degenerate, spin-charge-separated 0D soliton, where the twofold flux-state degeneracy in the Chern insulator [2D TI] is protected by SU(2) [${{{{{{{\mathcal{T}}}}}}}}$] symmetry (see SN 5A1 and 5B1). Nevertheless, ∣*C*∣ = 2 spin-degenerate Chern insulators and 2D TIs are still physically distinguishable by their ${\mathbb{Z}}$-valued Hall conductivities, where the Hall conductivity of the Chern insulator [2D TI] is given by *σ*^*H*^ = 2*e*^2^/*h* [*σ*^*H*^ = 0]^2,3,6^. **b** (Top) A 3D monopole-charged nodal-line semimetal (MNLSM) with a ${{{{{{{\mathcal{T}}}}}}}}$-reversed pair of nodal lines (red ellipses) that are locally protected by ${{{{{{{\mathcal{I}}}}}}}}$, ${{{{{{{\mathcal{T}}}}}}}}$, and SU(2) symmetries^71^ and carry nontrivial ${{\mathbb{Z}}}_{2}$ monopole charges^23,43^. (Bottom, left) Applying ${{{{{{{\mathcal{I}}}}}}}}$-symmetric orbital magnetism gaps the MNLSM in b into a $\theta \,{{{{{{{\rm{mod}}}}}}}}\ 2\pi=0$ spin-doubled AXI with ${{{{{{{\mathcal{I}}}}}}}}$ and SU(2) symmetries^23,43^. (Bottom, right) Conversely, ${{{{{{{\mathcal{I}}}}}}}}$-symmetric SOC gaps the MNLSM in **b** into a $\theta \,{{{{{{{\rm{mod}}}}}}}}\ 2\pi=0$ helical HOTI with ${{{{{{{\mathcal{I}}}}}}}}$ and ${{{{{{{\mathcal{T}}}}}}}}$ symmetries^23^. (Right) However, like the ∣*C*∣ = 2 spin-doubled Chern insulator and 2D TI in **a**, the spin-doubled AXI and helical HOTI in **b** exhibit the same *π*-flux response. In both 3D non-axionic HOTIs, *π*-flux tubes each bind a twofold-degenerate, spin-charge-separated 0D soliton on only one end, where the twofold surface flux-state degeneracy in the spin-doubled AXI [helical HOTI] is protected by SU(2) [${{{{{{{\mathcal{T}}}}}}}}$] symmetry (see SN 5A2 and 5B2).

Lastly, we will briefly discuss the limitations of static *π*-flux insertion as a complete diagnostic of bulk topology, suggesting interesting directions for future study. We begin by considering a 2D graphene-like topological semimetal with two fourfold Dirac cones protected locally by ${{{{{{{\mathcal{I}}}}}}}}$, ${{{{{{{\mathcal{T}}}}}}}}$, and SU(2) spin-rotation symmetry^71^ (Fig. 8a, top). The bulk may either be gapped by ${{{{{{{\mathcal{I}}}}}}}}$-symmetric orbital (Haldane) magnetism into a ∣*C*∣ = 2 spin-degenerate Chern insulator with ${{{{{{{\mathcal{I}}}}}}}}$ and SU(2) symmetries^72^ (Fig. 8a, bottom left), or by ${{{{{{{\mathcal{I}}}}}}}}$-symmetric SOC into a 2D TI with ${{{{{{{\mathcal{I}}}}}}}}$ and ${{{{{{{\mathcal{T}}}}}}}}$ symmetries^2,3^ (Fig. 8a, bottom right). However, from our earlier discussions and the numerical calculations performed in SN 5A1 and 5B1, we deduce that ∣*C*∣ = 2 spin-degenerate Chern insulators and 2D TIs exhibit the same *π*-flux response, despite being topologically distinct phases of matter. Specifically, when *π*-flux is threaded into ∣*C*∣ = 2 spin-doubled Chern insulators and 2D TIs, each flux core binds a twofold-degenerate, spin-charge-separated 0D soliton, where the twofold flux-state degeneracy in the Chern insulator [2D TI] is protected by SU(2) [${{{{{{{\mathcal{T}}}}}}}}$] symmetry (Fig. 8a, center right). Nevertheless, ∣*C*∣ = 2 spin-degenerate Chern insulators and 2D TIs are still physically distinguishable by their ${\mathbb{Z}}$-valued Hall conductivities, where the Hall conductivity of the Chern insulator [2D TI] is given by *σ*^H^ = 2*e*^2^/*h* [*σ*^H^ = 0]^2,3,6^.

In this work, we discover a similar pattern of identical static *π*-flux responses in two topologically distinct non-axionic 3D HOTIs that originate from the same semimetallic quantum critical point. We begin our analysis of 3D HOTIs by considering a 3D topological semimetal with a time-reversed pair of nodal lines at the Fermi level, where each nodal line is locally protected by ${{{{{{{\mathcal{I}}}}}}}}$, ${{{{{{{\mathcal{T}}}}}}}}$, and SU(2) symmetries^71^, and carries a nontrivial ${{\mathbb{Z}}}_{2}$ monopole charge^23,43^ (Fig. 8b, top). Monopole nodal-line semimetals (MNLSMs) represent the 3D, higher-order-topological^39^ generalizations of graphene, and MNLSM phases have been demonstrated to occur in 3D graphdiyne^43,73^ and *β*-MoTe_2_^23^ when the effects of SOC are neglected. Like graphene, 3D MNLSMs represent the quantum critical points between topologically distinct insulating phases^23^. A 3D MNLSM may be gapped by ${{{{{{{\mathcal{I}}}}}}}}$-symmetric orbital magnetism into an ${{{{{{{\mathcal{I}}}}}}}}$- and SU(2)-symmetric spin-doubled (spinless) AXI with two co-propagating chiral hinge modes and gapped 2D surfaces with anomalous SU(2)-symmetric ∣*C*∣ = 1 Chern insulating phases [where each spin sector contributes an anomalous half-integer surface Hall conductivity of *σ*^H^ = *e*^2^/(2*h*)]^19,23,43^ (Fig. 8b, bottom left). Alternatively, a 3D MNLSM may be gapped by ${{{{{{{\mathcal{I}}}}}}}}$-symmetric SOC into an ${{{{{{{\mathcal{I}}}}}}}}$- and ${{{{{{{\mathcal{T}}}}}}}}$-symmetric helical HOTI^23^ with helical hinge modes and gapped 2D surfaces with anomalous ${{{{{{{\mathcal{T}}}}}}}}$-invariant halves of 2D TI phases (Fig. 8b, bottom left), as demonstrated in this work (see Fig. 7e,g). However, from our discussions above and the numerical calculations performed in SN 5A2 and 5B2, we deduce that like the ∣*C*∣ = 2 spin-doubled Chern insulator and 2D TI in Fig. 8a, ${{{{{{{\mathcal{I}}}}}}}}$- and SU(2)-symmetric spin-doubled AXIs and ${{{{{{{\mathcal{I}}}}}}}}$- and ${{{{{{{\mathcal{T}}}}}}}}$-symmetric helical HOTIs exhibit the same *π*-flux response, despite being topologically distinct phases of matter. Specifically, when *π*-flux tubes are threaded into spin-doubled AXIs and helical HOTIs, each flux tube binds a twofold-degenerate, spin-charge-separated 0D soliton on only one end, where the twofold surface flux-state degeneracy in the spin-doubled AXI [helical HOTI] is protected by SU(2) [${{{{{{{\mathcal{T}}}}}}}}$] symmetry (Fig. 8b, center right). Distinctly unlike the ∣*C*∣ = 2 spin-doubled Chern insulator and 2D TI in Fig. 8a, spin-doubled AXIs and helical HOTIs both exhibit trivial ${{\mathbb{Z}}}_{2}$-valued axion angles $\theta \,{{{{{{{\rm{mod}}}}}}}}\ 2\pi=0$, and are therefore non-axionic.

It remains an open and urgent theoretical question whether there exists a quantized response effect beyond the axionic magnetoelectric effect and static *π*-flux insertion that can distinguish between spin-doubled AXIs and helical HOTIs. While it is clear that adiabatically threading a flux quantum can pump a charge ∣2*e*∣ [quantized spin] from the bulk of a flux tube to the boundary of a spin-doubled AXI [helical HOTI in the *s*^*z*^-conserving limit], neither effect is characterized by a well-established quantized response theory in noninteracting spinful topological (crystalline) insulators, such as the magnetoelectric effect. Specifically, the ${{\mathbb{Z}}}_{2}$-valued, axionic magnetoelectric response can only distinguish between pumping cycles that pass even and odd numbers of electron charges ∣*e*∣ per threaded flux quantum^6,36^, and therefore cannot distinguish between spin-doubled AXIs, helical HOTIs, and trivial insulators.

## Discussion

The HEND states proposed in this work may be observable through STM probes of the corners of edge dislocations and the surface terminations of screw dislocations and flux tubes (solenoids) in 3D insulators that, respectively, satisfy Eq. (6) or exhibit stable higher-order topology. For the case of flux-induced HEND states, it is important to note that for most solid-state topological materials^74^, an unrealistically strong magnetic field would be required to generate one *π*-flux per unit cell. However, because a 3D helical HOTI phase can be constructed by layering 2D TI states^21^, then by layering and twisting 2D TI layers to generate a Moiré potential, one could construct a HOTI with a much larger unit cell in which a proportionately smaller magnetic field is required to produce a *π*-flux. Twisted transition metal dichalcogenide few-layers have been theoretically predicted to host quantum spin Hall states^75^, and may hence represent a promising platform for probing the flux-induced HEND states and MSP HOTI response identified in this work. Additionally, in AXIs, the bulk magnetoelectric and anomalous surface Hall responses can be probed in optical experiments performed under applied magnetic fields significantly weaker than one *π*-flux per unit cell^6,64^. There may also exist analogous optical signatures of the anomalous surface half quantum spin Hall states in helical HOTIs predicted in this work, which we leave as an exciting direction for future investigations.

The recent theoretical and experimental identification of HOTI phases in materials including bismuth^48^, the transition metal dichalcogenides MoTe_2_ and WTe_2_^23,50–52^, BiBr^12,13,53,54^, the Ba_3_Cd_2_As_4_ family^14^, the Sr_3_PbO family of perovskites^76^, as well as in recently established vast databases of topological materials^49,74,77^ indicates particular promise for future experimental investigations of flux and defect HEND states. Spin-charge-separated HEND dislocation states may also be observable in weak FTI phases, for which several material candidates^74^ were recently discovered through the symmetry-based indicators of fragile topology introduced in refs. 44, 45. 3D OAL phases have recently been identified in electrides^78^ and other stoichiometric insulators^79^, and may also exhibit nontrivial HEND-state dislocation responses. For HEND states that carry chargeless spin, the spinon excitations may be detectable through nonlinear spectroscopy^80,81^. Additionally, recent investigations have revealed that ${{{{{{{\mathcal{T}}}}}}}}$-symmetric topological semimetals gapped with charge-density waves exhibit the same low-energy theory as helical HOTIs^57,82^, suggesting an intriguing future venue for investigating the spin-charge-separated defect and flux response effects introduced in this work. Furthermore, though we have focused on solid-state materials, metamaterials can also exhibit nontrivial defect and flux responses^83,84^, and may therefore provide an additional platform for realizing HEND states. Lastly, it was recently demonstrated that dislocations in *d*-D crystals can also map interacting (*d*−1)-D topological phases to real space^85^, suggesting that the interplay of crystal defects and topological order is a promising direction for future study.

## Methods

We will here summarize our analytic proofs of the criteria for generating 0D dislocation and flux HEND states in 3D insulators (see Table 1). Our proofs are supported by extensive numerical calculations of 0D dislocation and flux bound states, which we respectively detail in SN 4 and 5. We will then detail our first principles and tight-binding calculations demonstrating a nontrivial first-order dislocation response in 2D PbTe monolayers and a nontrivial HEND-state dislocation response in 3D SnTe.

### Summary of analytic HEND dislocation state proofs

In this work, we have formulated two alternative and equivalent sets of proofs demonstrating that integer dislocations map lower-dimensional topology from momentum space to position space. We have crucially demonstrated that dislocations can map not just stable topological phases with 1D edge modes, but also FTIs and OALs with anomalous 0D corner states. Our proofs further reproduce the results of all previous studies of crystal dislocation bound states with integer ***B***^24–29^. First, building upon the "cutting” and "gluing” construction of topological defect states developed in ref. 24 to predict helical dislocation modes in weak TIs^4^, we have employed *k* ⋅ *p* theory to predict 0D HEND states in 3D crystals (see SN 2A1 and 2A2). Next, in SN 2B1 and 2B3, we use more general arguments based on second-quantized expressions for noninteracting (topological) ground states to demonstrate that (*d*−2)-D dislocations in *d*-D crystals can map (*d*−1)-D BZ surfaces to (*d*−1)-D real-space surfaces, leading in 3D crystals to the presence of 1D and 0D topological defect states. Below, we will outline the *k* ⋅ *p*-level proof, leaving the more general case for SN 2B1 and 2B3.

For simplicity and without loss of generality, we will focus here on ${{{{{{{\mathcal{I}}}}}}}}$-symmetric, ${{{{{{{\mathcal{T}}}}}}}}$-broken insulators with edge dislocations. Because an ${{{{{{{\mathcal{I}}}}}}}}$- and ${{{{{{{\mathcal{T}}}}}}}}$-symmetric HOTI can be formed by superposing a time-reversed pair of ${{{{{{{\mathcal{I}}}}}}}}$-symmetric AXIs, the results derived here for magnetic AXIs (and ${{{{{{{\mathcal{I}}}}}}}}$-symmetric, ${{{{{{{\mathcal{T}}}}}}}}$-broken FTIs) can also be straightforwardly extended to helical HOTIs (and ${{{{{{{\mathcal{I}}}}}}}}$- and ${{{{{{{\mathcal{T}}}}}}}}$-symmetric FTIs), as shown in SN 2A1 and 2A2. To begin the summary of our *k* ⋅ *p* derivation of anomalous HEND-state dislocation response, the low-energy *k* ⋅ *p* Bloch Hamiltonian of an ${{{{{{{\mathcal{I}}}}}}}}$-symmetric insulator can be expressed as:

10 $${{{{{{{\mathcal{H}}}}}}}}({{{{{{{\boldsymbol{q}}}}}}}})=\mathop{\bigoplus}\limits_{a}{{{{{{{{\mathcal{H}}}}}}}}}_{a}({{{{{{{\boldsymbol{q}}}}}}}}),$$

where *a* runs over the TRIM points ***k***_D,*a*_ whose bands are inverted relative to those of the atomic insulator formed from the occupied atomic orbitals when all hoppings are taken to vanish^18^, and where ***q*** = ***k***−***k***_D,*a*_. We next take the simplifying assumption that the *k* ⋅ *p* Hamiltonian at each TRIM point ***k***_D,*a*_ has the form of the low-energy theory of the Bernevig–Hughes–Zhang model of a 3D TI^2,5,6^:

11 $${{{{{{{{\mathcal{H}}}}}}}}}_{a}({{{{{{{\boldsymbol{q}}}}}}}})={m}_{a}{\tau }^{z}+\mathop{\sum}\limits_{i=x,y,z}{v}_{i}{q}_{i}{\tau }^{x}{\sigma }^{i},$$

where *τ*^*x*,*y*,*z*^ and *σ*^*x*,*y*,*z*^ are Pauli matrices, and where we have employed the shorthand notation *τ*^*i*^ ⊗ *σ*^ *j*^ ≡ *τ* ^*i*^*σ* ^*j*^. We emphasize that in a four-band model with singly degenerate bands (such as a model with only ${{{{{{{\mathcal{I}}}}}}}}$ symmetry), we must invert two bands in order to ensure a band gap throughout the BZ: a single band inversion about a TRIM point instead gives rise to a Weyl semimetal phase^19^. As we are in this work focusing on gapped topological phases, the minimal realization of Eq. (11) relevant to the dislocation responses analyzed in this work hence involves a 4 × 4 *k* ⋅ *p* Hamiltonian.

Next, we construct a long-wavelength description of a pair of edge dislocations whose Burgers vectors lie along a crystallographic axis. As prescribed in ref. 24, we model an internal loop of edge dislocations by cutting the insulator described by ${{{{{{{\mathcal{H}}}}}}}}({{{{{{{\boldsymbol{q}}}}}}}})$ [Eq. (10)] into two pieces with $\pm \hat{z}$-normal (top and bottom) surfaces, and then "gluing” the two pieces back together with ∣***B***∣/*c* extra rows of unit cells in the region between the edge dislocations, where *c* is the lattice spacing in the *z*-direction. We initially implemented the gluing with ${{{{{{{\mathcal{I}}}}}}}}$- and ${{{{{{{\mathcal{T}}}}}}}}$-symmetric coupling between the top and bottom surfaces, and then later relax ${{{{{{{\mathcal{T}}}}}}}}$ symmetry. At each TRIM point in the bulk at which bands are inverted, the top and bottom surfaces each contribute a twofold Dirac-cone surface state to the interface. This implies that the combined top and bottom surfaces carry one effective fourfold Dirac fermion in 2D for each band inversion in the bulk, where each fourfold Dirac fermion admits a single, ${{{{{{{\mathcal{T}}}}}}}}$-symmetric mass term^10^. To account for the presence or absence of nonzero ***B***, we derive in SN 2A1 a consistent, intrinsic phase for the coupling mass at each band-inverted TRIM point, finding in particular that the relative sign across the dislocation of the mass of the fourfold Dirac cone induced from the TRIM *a* is proportional to $\cos ({{{{{{{{\boldsymbol{k}}}}}}}}}_{{\mathrm {D}},a}\cdot {{{{{{{\boldsymbol{B}}}}}}}})$. Hence, the edge dislocation loop effectively realizes an interface between two gapped fourfold Dirac cones, where the relative sign of the gap is given by $\cos ({{{{{{{{\boldsymbol{k}}}}}}}}}_{{\mathrm {D}},a}\cdot {{{{{{{\boldsymbol{B}}}}}}}})$. If the relative sign is negative, then the Dirac mass switches sign, and the resulting domain wall binds a helical pair of defect-localized states^39,58^. This implies that for each band-inverted bulk TRIM point ***k***_D,*a*_ will only contribute helical modes to an edge dislocation if ***k***_D,*a*_ ⋅ ***B*** is an odd multiple of *π*. Lastly, we relax ${{{{{{{\mathcal{T}}}}}}}}$ symmetry while preserving ${{{{{{{\mathcal{I}}}}}}}}$ symmetry. From the analysis of ${{{{{{{\mathcal{I}}}}}}}}$-symmetric 2D insulators with anomalous corner modes in refs. 17, 23, we can immediately deduce that each of the bulk TRIM points that previously contributed a pair of helical modes at the edge dislocation will necessarily now contribute an anomalous number of 0D ± *e*/2-charged (anti)solitons under the introduction of ${{{{{{{\mathcal{I}}}}}}}}$-symmetric magnetism. As discussed in refs. 17, 23, 39, 47, this conclusion is crucially not reliant on particle–hole symmetry, which is not present in real materials^74^.

### Summary of analytic HEND flux state proofs

In this work, we have also formulated two alternative and equivalent sets of proofs demonstrating that *π*-flux tubes in 3D insulators bind anomalous 1D and 0D states, including HEND states, if and only if the bulk is a stable TI or TCI. Our proofs reproduce the results of refs. 6, 27, 31–33, as well as suggest the presence of a novel quantized *π*-flux response in ${{{{{{{\mathcal{I}}}}}}}}$- and ${{{{{{{\mathcal{T}}}}}}}}$-symmetric helical (non-axionic) HOTIs. As previously for integer dislocation bound states, our flux-state proofs were performed both within the *k* ⋅ *p* approximation for 3D insulators (SN 2A3) and using more general arguments based on second-quantized expressions for the noninteracting (topological) ground states of *d*-D insulating crystals (SN 2B2). Below, we will detail the *k* ⋅ *p*-level proof, leaving the more general case for SN 2B2.

We will again here focus on the response of ${{{{{{{\mathcal{I}}}}}}}}$-symmetric, ${{{{{{{\mathcal{T}}}}}}}}$-broken 3D insulators. Because an ${{{{{{{\mathcal{I}}}}}}}}$- and ${{{{{{{\mathcal{T}}}}}}}}$-symmetric helical HOTI can be formed by superposing a time-reversed pair of ${{{{{{{\mathcal{I}}}}}}}}$-symmetric AXIs^23^, then the *π*-flux-tube response derived here for magnetic AXIs can straightforwardly be extended to helical HOTIs, as detailed and performed in SN 2A3. We begin the summary of our *k* ⋅ *p* derivation of anomalous HEND-state *π*-flux response by again considering a 3D insulator with (initially) ${{{{{{{\mathcal{I}}}}}}}}$ and ${{{{{{{\mathcal{T}}}}}}}}$ symmetries. We take the 3D insulator to differ from a trivial atomic insulator by a series of band inversions at a set of TRIM points {***k***_D,*a*_} between Kramers pairs of states with opposite parity eigenvalues. The low-energy Hamiltonian of the insulator in the absence of threaded magnetic flux is hence again given by Eqs. (10) and (11).

Next, we construct a long-wavelength description of magnetic flux threaded into the 3D insulator through two parallel 1D tubes with opposite field strengths ±*ϕ* located at ${{{{{{{\mathcal{I}}}}}}}}$-related positions. To implement the pair of flux tubes, we cut the insulator described by ${{{{{{{\mathcal{H}}}}}}}}({{{{{{{\boldsymbol{q}}}}}}}})$ [Eq. (10)] into two pieces with $\pm {\hat{x}}_{\perp }$-normal surfaces, and again glue the pieces back together. In the region between the flux tubes, we multiply all couplings between the top and bottom surface states by *e*^i*ϕ*^. We emphasize that the effective ±*ϕ*/2 phase rotation per surface only represents a local gauge transformation on each surface in the limit in which the surfaces are considered separately—however when the two surfaces are coupled, the *ϕ* phase difference between the surfaces corresponds to the gauge-invariant insertion of ±*ϕ*-fluxes along the boundaries of the region between the flux tubes.

As previously for integer dislocations, the interface between the top and bottom surfaces contains an effective fourfold Dirac cone from the two twofold surface Dirac cones contributed by each bulk band inversion at ***k***_D,*a*_ (one twofold Dirac cone from each of the top and bottom surfaces). However, unlike for integer dislocations, the ${{{{{{{\mathcal{T}}}}}}}}$-symmetric mass of the fourfold Dirac cone carries a relative phase of *e*^i*ϕ*^ between the regions inside and outside of the pair of flux tubes. Hence crucially, and unlike in the previous case of edge and screw dislocations, the relative sign of the fourfold Dirac mass for *π*-flux tubes is independent of ***k***_D,*a*_. This implies that when *ϕ* = *π*, the two flux tubes bind an odd (anomalous) number of helical pairs of modes if the bulk contains an odd total number of band inversions between Kramers pairs of states at TRIM points such that—through the Fu–Kane parity criterion—the bulk is a 3D TI^4^. Alternatively, this result may be summarized through the statement that *π*-flux tubes bind anomalous helical modes in an ${{{{{{{\mathcal{I}}}}}}}}$- and ${{{{{{{\mathcal{T}}}}}}}}$-symmetric 3D insulator if the 2D momentum-space Hamiltonian in only one of the ${k}_{{x}_{\perp }}=0,\, \pi$ BZ planes is equivalent to a 2D TI, because a 3D TI can be expressed as a helical pump of a 2D TI^4,6^. As shown in SN 2A3 and 2B2, we find more generally that a parallel pair of *x*_∥2_-directed *π*-flux tubes separated by a distance along *x*_∥1_ sums the 2D topology of all of the momentum-space Hamiltonians in the ${k}_{{x}_{\perp }}$-indexed BZ planes of the pristine insulating crystal [see Eq. (10), and note that *x*_∥1,2_ span the plane perpendicular to *x*_⊥_]. The summed 2D momentum-space topology is then projected onto the real-space surface spanning the flux tubes.

From this result, it is straightforward to derive the *π*-flux response of ${{{{{{{\mathcal{I}}}}}}}}$-symmetric AXIs. Numerous previous works^4–6,17,35–37^ have shown that an ${{{{{{{\mathcal{I}}}}}}}}$-symmetric 3D strong TI gaps into an AXI under the introduction of ${{{{{{{\mathcal{I}}}}}}}}$-symmetric magnetism. Furthermore, it was shown in recent works^17,23^ that, because an ${{{{{{{\mathcal{I}}}}}}}}$-symmetric 2D TI gaps into a 2D FTI with anomalous ±*e*/2-charged corner modes, then an AXI is equivalent to an odd, chiral pumping cycle of an ${{{{{{{\mathcal{I}}}}}}}}$-symmetric 2D FTI. Hence, when ${{{{{{{\mathcal{T}}}}}}}}$ symmetry is relaxed in an ${{{{{{{\mathcal{I}}}}}}}}$-symmetric 3D TI with two *π*-flux tubes, the Dirac-cone surface states, and helical flux states become gapped, but there remain an anomalous number of ±*e*/2-charged 0D states bound to the loop formed from the two flux tubes and the crystal surfaces. Hence, *π*-flux tubes in an AXI necessarily bind anomalous ±*e*/2-charged 0D HEND states, which appear in our numerical calculations on ${{{{{{{\mathcal{I}}}}}}}}$-related flux tube ends (see SN 5A2 and 5B2).

Because an ${{{{{{{\mathcal{I}}}}}}}}$- and ${{{{{{{\mathcal{T}}}}}}}}$-symmetric helical HOTI is equivalent to the superposition of a time-reversed pair of ${{{{{{{\mathcal{I}}}}}}}}$-symmetric AXIs^23^, then the previous derivation of flux-tube HEND states in AXIs also implies the *π*-flux response of helical HOTIs. Specifically, as detailed in SN 2A3, we discover in this work that *π*-flux tubes threaded into helical HOTIs bind Kramers pairs of spin-charge-separated 0D HEND states, rather than ±*e*/2 end charges.

### First principles and tight-binding calculation details for PbTe monolayers

We will here detail our first principles and tight-binding calculations for 2D PbTe monolayers (see SN 6A for complete calculation details). To obtain the crystal structure of a single, pristine monolayer of PbTe, we start with a 3D crystal of rock-salt-structure PbTe [SG 225 $Fm\bar{3}m1^{\prime}$, Inorganic Crystal Structure Database (ICSD)^86^ No. 194220, further details available at https://topologicalquantumchemistry.com/#/detail/194220^18,74,87–89^], increase the lattice spacing in the *z* (*c*-axis) direction to isolate a single plane of Pb and Te atoms, and then restrict the system symmetry to layer group (LG)^10,39,90–94^$p4/mmm1^{\prime}$. We next perform fully relativistic DFT calculations of the electronic structure using the Vienna Ab initio Simulation Package (VASP)^95,96^ employing the projector-augmented wave (PAW) method^97,98^ and the Perdew, Burke, and Ernzerhof generalized-gradient approximation (GGA-PBE)^99^ for the exchange-correlation functional. In our first-principles calculations, we have used the primitive unit cell shown in Fig. 3a, which contains one Pb atom at (*x*, *y*) = (0, 0) and one Te atom at (1/2, 0). The lattice vectors of the primitive cell (see Fig. 3a) are given by

12 $${{{{{{{{\boldsymbol{a}}}}}}}}}_{1}=(1/2,-1/2),\,{{{{{{{{\boldsymbol{a}}}}}}}}}_{2}=(1/2,\, 1/2),$$

and the reciprocal lattice vectors are given by

13 $${{{{{{{{\boldsymbol{b}}}}}}}}}_{1}=2\pi (1,-1),\,{{{{{{{{\boldsymbol{b}}}}}}}}}_{2}=2\pi (1,\, 1).$$

Lastly, we have allowed the in-plane lattice spacing *a*_1_ = *a*_2_ = *a* to relax from its experimental value to an equilibrium length of *a* = 4.483 Å.

To determine the topological indices of the PbTe monolayer, we use the IrRep program^100^ to first deduce the small corepresentations (coreps) of the six highest valence and the two lowest conduction bands, which are shown in Fig. 3c, d and labeled employing the convention of the REPRESENTATIONS DSG tool on the BCS^18,101^ for the *k*_*z*_ = 0 plane of SG 123 $P4/mmm1^{\prime}$, the index-2 supergroup of LG $p4/mmm1^{\prime}$ generated by adding lattice translations in the *z*-direction.

Next, to determine the dislocation response of PbTe monolayers, we calculate the weak (partial) SSH invariant vector ${{{{{{{{\boldsymbol{M}}}}}}}}}_{\nu }^{{{{{{{{\rm{SSH}}}}}}}}}$, which is defined in the text surrounding Eq. (2). ${{{{{{{{\boldsymbol{M}}}}}}}}}_{\nu }^{{{{{{{{\rm{SSH}}}}}}}}}$ can be obtained by counting the number of parity-eigenvalue-exchanging band inversions by which a set of bands differs from an unobstructed (trivial) atomic limit with a trivial dislocation response. As shown in Fig. 3c, d, PbTe monolayers differ from an unobstructed atomic limit through band inversion at the *X* point [***k***_*X*_ = ***b***_1_/2 = (*π*, − *π*)] between bands labeled by the small coreps ${\bar{X}}_{5,6}$ of the little group at *X*. The small coreps ${\bar{X}}_{5,6}$ correspond to doubly degenerate pairs of states with the same parity (${{{{{{{\mathcal{I}}}}}}}}$) eigenvalues within each pair, such that:

14 $${\chi }_{{\bar{X}}_{5}}({{{{{{{\mathcal{I}}}}}}}})=2,\,{\chi }_{{\bar{X}}_{6}}({{{{{{{\mathcal{I}}}}}}}})=-2,$$

where *χ*_*ρ*_(*h*) is the character of the unitary symmetry *h* in the corep *ρ*, and is equal to the sum of the eigenvalues of *h* in *ρ*. Because the *X* and symmetry-equivalent $X^{\prime}$ [${{{{{{{{\boldsymbol{k}}}}}}}}}_{X^{\prime} }={C}_{4z}{{{{{{{{\boldsymbol{k}}}}}}}}}_{X}\,{{{{{{{\rm{mod}}}}}}}}\,{{{{{{{{\boldsymbol{b}}}}}}}}}_{1}\,{{{{{{{\rm{mod}}}}}}}}\,{{{{{{{{\boldsymbol{b}}}}}}}}}_{2}={{{{{{{{\boldsymbol{b}}}}}}}}}_{2}/2=(\pi,\, \pi )$] points lie along the BZ-edge *XM* and $X^{\prime} M$ lines, then we conclude that PbTe monolayers exhibit a nontrivial weak partial (time-reversal) SSH invariant vector:

15 $${{{{{{{{\boldsymbol{M}}}}}}}}}_{\nu }^{{{{{{{{\rm{SSH}}}}}}}}}=\frac{1}{2}({{{{{{{{\boldsymbol{b}}}}}}}}}_{1}+{{{{{{{{\boldsymbol{b}}}}}}}}}_{2})=(2\pi,\, 0).$$

We emphasize that, despite ${\nu }_{x}^{{{{{{{{\rm{SSH}}}}}}}}}\,{{{{{{{\rm{mod}}}}}}}}\ 2\pi={\nu }_{y}^{{{{{{{{\rm{SSH}}}}}}}}}\,{{{{{{{\rm{mod}}}}}}}}\ 2\pi=0$ in Eq. (15), ${{{{{{{{\boldsymbol{M}}}}}}}}}_{\nu }^{{{{{{{{\rm{SSH}}}}}}}}}$ is still nontrivial, because (2*π*, 0) and (0, 2*π*) are not reciprocal lattice vectors [Eq. (13)] in the rotated coordinates employed in our calculations.

To confirm the nontrivial dislocation response of a PbTe monolayer, we next insert a pair of 0D dislocations into an eight-band tight-binding model obtained from maximally-localized, symmetric Wannier functions through WANNIER90^102,103^. In practice, when mapping a DFT calculation to a tight-binding model, one must choose a cutoff distance for hopping interactions. Surprisingly, even though the band inversion in PbTe monolayers is relatively strong (the negative band gap at the *X* and $X^{\prime}$ points is roughly ~ 260 meV)^40,41,104^, we find that the strong and weak partial-polarization topology of a PbTe monolayer is only reproduced in a tight-binding model that is truncated to a minimum range of sixth-nearest-neighbor hopping. As detailed in SN 6A and shown in Fig. 3d, e, computing the PBC spectrum of our Wannier-based tight-binding model with a pair of ***B*** = ***a***_1_ dislocations, we observe four filling-anomalous dislocations bound states, confirming the nontrivial first-order dislocation response of PbTe monolayers.

### First-principles and tight-binding calculation details for 3D SnTe

We will next detail our first principles and tight-binding calculations demonstrating a nontrivial HEND-state dislocation response in 3D SnTe crystals (see SN 6B for complete calculation details). To draw a comparison with SnTe, we have also performed analogous calculations on the isostructural compound PbTe, which we find to exhibit a trivial dislocation response. We begin by performing fully-relativistic DFT calculations of the electronic structure of 3D SnTe and PbTe using VASP^95,96^ employing the PAW method^97,98^ and GGA-PBE^99^ for the exchange-correlation functional. The lattice parameters of the rock-salt structure [SG 225 $Fm\bar{3}m1^{\prime}$] were fixed to their experimental values^105^*a* = 6.32 Å for SnTe and *a* = 6.46 Å for PbTe.

Below, we will specifically compute the dislocation response for the shortest possible dislocation Burgers vectors —i.e. dislocations for which the Burgers vector ***B*** is equal to one of the primitive, face-centered-cubic lattice vectors of SnTe or PbTe. For geometric simplicity and because 3D SnTe and PbTe are cubic, we without loss of generality form a tetragonal supercell in which the ***a***_1_ and ***a***_2_ primitive lattice vectors are also lattice vectors in the face-centered cubic cell, but in which ***a***_3_ is $\sqrt{2}$ times the length of a face-centered-cubic primitive lattice vector (see Fig. 5a). The tetragonal cell specifically contains two Sn/Pb atoms at (*x*, *y*, *z*) = (0, 0, 0) and (1/2, 1/2, 1/2) and two Te atoms at (0, 0, 1/2) and (1/2, 1/2, 0), and respects the symmetries of SG 123 $P4/mmm1^{\prime}$. The lattice and reciprocal lattice vectors of the tetragonal supercell are shown in Fig. 5a and detailed in Eq. (8) and the surrounding text. In our first-principles calculations, we only incorporate valence-shell states – hence, our calculations only include the 5*p* orbitals of Te and 5*p* (6*p*) orbitals of Sn (Pb), as well as twelve total empty conduction bands from higher-shell (empty) valence orbitals. Therefore, at each TRIM point in Fig. 5c, the lower twelve (upper twelve) bands are occupied (unoccupied) [the bands in Fig. 5c are fourfold degenerate due to the combined effects of spinful ${{{{{{{\mathcal{I}}}}}}}}\times {{{{{{{\mathcal{T}}}}}}}}$ symmetry and supercell BZ folding].

${{{{{{{{\boldsymbol{M}}}}}}}}}_{\nu }^{{{{{{{{\rm{F}}}}}}}}}$ can be obtained by counting the number of parity-eigenvalue-exchanging band inversions by which a set of bands differs from an unobstructed atomic limit with a trivial dislocation response. We first establish, in agreement with previous works^8^, that 3D PbTe realizes an unobstructed atomic limit in which three Kramers pairs of states are located on each of the four Te atoms in the tetragonal supercell. Our calculations indicate that 3D SnTe differs from 3D PbTe by double band inversions at the *R* point [***k***_*R*_ = ***b***_1_] and at the symmetry-related point $R^{\prime}$ [${{{{{{{{\boldsymbol{k}}}}}}}}}_{R^{\prime} }={C}_{4z}{{{{{{{{\boldsymbol{k}}}}}}}}}_{R}\,{{{{{{{\rm{mod}}}}}}}}\ {{{{{{{{\boldsymbol{b}}}}}}}}}_{1}\,{{{{{{{\rm{mod}}}}}}}}\ {{{{{{{{\boldsymbol{b}}}}}}}}}_{2}={{{{{{{{\boldsymbol{b}}}}}}}}}_{2}/2$] between two pairs of Kramers pairs of states with opposite parity eigenvalues [four valence states become inverted with four conduction states at *R* and at $R^{\prime}$].

To determine the dislocation response of SnTe, we first establish that ${{{{{{{{\boldsymbol{M}}}}}}}}}_{\nu }^{{{{{{{{\rm{F}}}}}}}}}={{{{{{{\boldsymbol{0}}}}}}}}$ in PbTe, because PbTe is an unobstructed atomic limit. Hence, because SnTe differs from PbTe by double band inversions at the *R* and $R^{\prime}$ points in the tetragonal supercell (see Fig. 5), the HEND-state response of SnTe is nontrivial:

16 $${{{{{{{{\boldsymbol{M}}}}}}}}}_{\nu }^{{{{{{{{\rm{F}}}}}}}}}=({{{{{{{{\boldsymbol{b}}}}}}}}}_{1}+{{{{{{{{\boldsymbol{b}}}}}}}}}_{2})/2=(2\pi,\, 0,\, 0).$$

We emphasize that, despite ${\nu }_{x}^{{{{{{{{\rm{F}}}}}}}}}\,{{{{{{{\rm{mod}}}}}}}}\ 2\pi={\nu }_{y}^{{{{{{{{\rm{F}}}}}}}}}\,{{{{{{{\rm{mod}}}}}}}}\ 2\pi=0$ in Eq. (16), ${{{{{{{{\boldsymbol{M}}}}}}}}}_{\nu }^{{{{{{{{\rm{F}}}}}}}}}$ is still nontrivial, because (2*π*, 0, 0) and (0, 2*π*, 0) are not reciprocal lattice vectors in the tetragonal supercell of SnTe [Eq. (8)].

We next explicitly confirm the nontrivial defect response of 3D SnTe. To model an edge dislocation in SnTe, we use the tight-binding model from ref. 8, with the parameters listed in ref. 106. We first enlarge the model unit cell to obtain the tetragonal supercell shown in Fig. 5a. We then determine the locations of the ${{{{{{{\mathcal{I}}}}}}}}$ centers in the supercell from the mirror symmetry representations given in ref. 106— in real space, the Sn and Te atoms in the model in ref. 8 occupy inversion centers that coincide with lines of *C*_4*z*_ (fourfold rotation) symmetry in the tetragonal supercell (Fig. 5a). Next, we implement an internal edge dislocation with ***B*** = ***a***_1_, as shown in Fig. 6a and detailed in SN 6B2. Importantly, in order to use filling anomalies to diagnose the nontrivial HEND-state dislocation response, we must implement the defect plane in an ${{{{{{{\mathcal{I}}}}}}}}$-symmetric manner, which we accomplish with the alternating pattern of site removal depicted in Fig. 6a.

To provide a reference for our numerical analysis of the defect response in 3D SnTe, we have also implemented a ***B*** = ***a***_1_ pair of edge dislocations in a tight-binding model of 3D PbTe. To construct the tight-binding model, we have increased the on-site energy difference between the two inequivalent atoms in the primitive unit cell [specifically, in the notation of ref. 106, we have changed the parameter *m* from 1.65 to 3 in Eq. (16) in ref. 106]. Increasing the on-site energies reverses the pair of double band inversions at *R* and $R^{\prime}$, and reproduces the first-principles-derived parity eigenvalues and electronic structure of PbTe. The on-site potential can also be understood as a chemical potential that localizes all of the electrons on the Te atoms of PbTe. Because PbTe is isostructural to SnTe, then the real-space defect geometry for our tight-binding model of PbTe is identical to the defect geometry previously employed in SnTe (depicted in Fig. 6a).

In Fig. 6b, we plot the PBC defect spectrum for SnTe, and in SN 6B2, we plot the analogous defect spectrum for PbTe. The dislocation spectrum of PbTe exhibits a large gap and is trivial, whereas the defect spectrum of SnTe is conversely filling-anomalous, specifically exhibiting four midgap states (two Kramers pairs corresponding to the circled states in Fig. 6c). This result validates our first-principles bulk identification of a nontrivial HEND-state dislocation response vector in 3D SnTe, and a trivial HEND-state response vector in 3D PbTe.

## Supplementary information

Supplementary Information

## Data Availability

The data supporting the findings of this study are available from the corresponding authors upon reasonable request.
