# Supplementary material for: Topological zero-dimensional defect and flux states in three-dimensional insulators
Source: Nat Commun. 2022 Oct 2;13:5791. doi: 10.1038/s41467-022-33471-x (PMC9527258; doi:10.1038/s41467-022-33471-x)
Supplement: Supplementary file 1 — Supplementary Information [file 41467_2022_33471_MOESM1_ESM.pdf]

Supplementary Information for "Topological Zero-Dimensional Defect and Flux States in Three-Dimensional Insulators"

Frank Schindler,[1,*] Stepan S. Tsirkin,[2] Titus Neupert,[2] B. Andrei Bernevig[‡],[3,4,5] and Benjamin J. Wieder[◇3,6,7,†]

[1]*Princeton Center for Theoretical Science, Princeton University, Princeton, NJ 08544, USA*
[2]*Department of Physics, University of Zurich, Winterthurerstrasse 190, 8057 Zurich, Switzerland*
[3]*Department of Physics, Princeton University, Princeton, NJ 08544, USA*
[4]*Donostia International Physics Center, P. Manuel de Lardizabal 4, 20018 Donostia-San Sebastian, Spain*
[5]*IKERBASQUE, Basque Foundation for Science, Bilbao, Spain*
[6]*Department of Physics, Massachusetts Institute of Technology, Cambridge, MA 02139, USA*
[7]*Department of Physics, Northeastern University, Boston, MA 02115, USA*

(Dated: September 15, 2022)

[‡]Primary address for B. A. B.: Department of Physics, Princeton University, Princeton, NJ 08544, USA.

[◇]Primary address for B. J. W.: Department of Physics, Massachusetts Institute of Technology, Cambridge, MA 02139, USA.

[*] Corresponding author: schindler@princeton.edu

[†] Corresponding author: bwieder@mit.edu

CONTENTS

Supplementary Notes 3

1. Review of Recent Related Literature 3

2. Mapping Topology from Momentum Space to Position Space with Defects and π-Flux 4
 A. Defect and Flux Bound States in 3D $\mathcal{I}$-Symmetric Insulators from $k \cdot p$ Theory 4
 1. $k \cdot p$ Derivation of HEND States on Edge Dislocation Corners in AXIs and Weak Fragile TIs 5
 2. $k \cdot p$ Derivation of HEND States on Screw Dislocation Ends in AXIs and Weak Fragile TIs 12
 3. $k \cdot p$ Derivation of HEND States on Flux Tube Ends in AXIs and HOTIs 15
 B. Ground State Mapping Proofs for Defect and π-Flux States 20
 1. Edge Dislocations in d-D Crystals 20
 2. π-Flux States in d-D Crystals 22
 3. Screw Dislocations in 3D Crystals 25

3. Weak Fragile and Obstructed-Atomic-Limit Indices for 2D Dislocation and 3D HEND States 28
 A. The 2D Weak SSH Invariant $\boldsymbol{M}_{\nu}^{\mathrm{SSH}}$ 28
 B. The 3D Weak Fragile Invariant $\boldsymbol{M}_{\nu}^{\mathrm{F}}$ 29
 1. $\mathcal{T}$-Broken EBR Formulation of $\boldsymbol{M}_{\nu}^{\mathrm{F}}$ 30
 2. $\mathcal{T}$-Symmetric EBR Formulation of $\boldsymbol{M}_{\nu}^{\mathrm{F}}$ 34
 3. $\mathcal{T}$-Symmetric Nested Wilson Loop Formulation of Partial Nested Berry Phase 36

4. Numerical Calculation Details: Edge and Screw Dislocations 40
 A. Dislocation Response without Time-Reversal Symmetry 40
 1. 2D Point Dislocations in the Absence of $\mathcal{T}$ Symmetry 40
 2. 3D Screw Dislocations in the Absence of $\mathcal{T}$ Symmetry 44
 B. Dislocation Response with Time-Reversal Symmetry 47
 1. 2D Point Dislocations in the Presence of $\mathcal{T}$ Symmetry 47
 2. 3D Screw Dislocations in the Presence of $\mathcal{T}$ Symmetry 50

5. Numerical Calculation Details: Fluxes and Flux Tubes 52
 A. Flux Response without Time-Reversal Symmetry 52
 1. 2D Fluxes in the Absence of $\mathcal{T}$ Symmetry 52
 2. 3D Flux Tubes in the Absence of $\mathcal{T}$ Symmetry 54
 B. Flux Response with Time-Reversal Symmetry 55
 1. 2D Fluxes in the Presence of $\mathcal{T}$ Symmetry 55
 2. 3D Flux Tubes in the Presence of $\mathcal{T}$ Symmetry 56

6. First-Principles Calculation Details 58
 A. 2D PbTe Monolayers 58
 B. 3D SnTe 61
 1. First-Principles Calculation of the Nontrivial Dislocation Response Vector in SnTe 61
 2. Tight-Binding Calculation of Topological HEND States in 3D SnTe 63

Supplementary References 65

SUPPLEMENTARY NOTES

1. REVIEW OF RECENT RELATED LITERATURE

There has been tremendous progress in recent years linking the band topology of pristine crystals [1–70] to the anomalous electronic states that can be bound by crystal defects [71–94]. For the topological crystalline insulating phases [14, 15, 18–20, 23–29] that have become known as higher-order TIs (HOTIs) [30–36, 40, 41, 95–110], these efforts have had only recent, incipient success, motivating the present study. Furthermore, though HOTI material candidates are readily accessible, experimental studies of candidate HOTIs have yielded results that have attracted an array of – at times contradictory – explanations [79, 102, 111–126]. In this work, we have performed extensive theoretical and numerical calculations demonstrating that dislocations with integer Burgers vectors [127] in 3D insulators can bind 0D higher-order end (HEND) states with anomalous charge and spin as a consequence of the bulk topology [see Supplementary Notes (SN) 2 A 1, 2 A 2, 2 B 1, 2 B 3, 3, and 4]. Using first-principles calculations, we have shown that PbTe monolayers [128–130] have a nontrivial first-order defect-state response (SN 6 A), and that the 3D TCI and HOTI SnTe (but not PbTe) [26, 31, 32, 131–133] carries a nontrivial HEND-state dislocation response (SN 6 B). HEND dislocation states may also be observable in "fragile" topological insulators (TIs) [33, 70, 100, 107–110, 134–151] and obstructed atomic limits [152–156]. Numerous candidate fragile TIs have recently been predicted through high-throughput material searches [132, 151], and 3D OAL phases have recently been identified in electrides [157–159] and other stoichiometric insulators [160–163].

In this work, we have additionally analytically and numerically compared the electronic states bound to crystal defects to the related problem of magnetic flux insertion (see SN 2 A 3, 2 B 2, and 5), which has previously been shown to provide a probe of bulk stable topology [76, 164–171]. Building from the previous recognition that the gapped 2D surfaces of 3D magnetic axion insulators (chiral HOTIs) [30–33, 96, 97, 100, 101, 106–110, 155, 172–175] carry anomalous half-integer quantum Hall states that can be detected by magnetic flux insertion [167, 168], we applied the techniques of magnetic flux insertion and filling anomalies [33, 70, 100, 176] to $\mathcal{I}$- and $\mathcal{T}$-symmetric helical HOTIs, for which the bulk and surface theories are unknown. Our numerical calculations reveal in particular that the gapped surfaces of helical HOTIs are neither trivial nor integer topological states, but rather carry anomalous halves of $\mathcal{T}$-invariant quantum *spin* Hall (QSH) states (SN 2 A 3 and 5 B 2). Similar half-integer QSH states were previously predicted to appear on the top surfaces of weak TIs [177], and in this work, we recognize the anomalous half-integer QSH state to more generally manifest on *all* gapped surfaces of $\mathcal{I}$- and $\mathcal{T}$-symmetric HOTIs. This finding raises several interesting questions for future works, including whether halves of QSH states can be distinguished in the absence of s^z-spin symmetry [3, 5, 6, 178–180], and whether studies of Anderson localization on weak TI and TCI surfaces [181–185] should be revisited in light of the existence of anomalous gapped surface states with trivial (charge) Hall conductivities (*i.e.* half QSH states). Additionally, previous constructions of strongly-interacting topological phases have exploited the half-quantized surface quantum Hall effect of 3D TIs [186–189], and our identification of a half-quantized surface quantum spin Hall effect in HOTIs may hence also provide further insight towards the theoretical construction of $\mathcal{T}$-symmetric fractional TIs and other phases with anomalous topological order [190–192]. Lastly, we also explored the limitations of static π-flux insertion as a complete diagnostic of bulk topology by theoretically demonstrating the presence of identical π-flux-tube spectra in two physically distinct phases of matter: an $\mathcal{I}$- and $\mathcal{T}$-protected helical HOTI [31, 100, 102, 103, 105, 173] with anomalous surface half quantum spin Hall states, and an $\mathcal{I}$- and SU(2)-symmetric spin-doubled (spinless) axion insulator with anomalous surface integer quantum Hall states [100, 144, 155, 193, 194]. Though we have largely focused in this work on helical HOTIs, for which numerous material candidates are known [29, 100, 102, 115–117, 131, 132, 195–198], an $\mathcal{I}$- and SU(2)-symmetric spin-doubled (spinless) axion insulator phase can be realized by inducing $\mathcal{T}$-symmetric orbital (Haldane) magnetism in a monopole-charged nodal-line semimetal state [64, 100, 144] like that predicted in 3D graphdiyne [199, 200].

During the long preparation of this work, several other works also studied defect and flux responses in topological systems and closely related theoretical methods. First, concurrent with the preparation of this work and consistent with our findings, a bulk spin-magnetoelectric response and anomalous surface half QSH states were numerically identified in helical HOTIs in Supplementary Reference (SRef.) 201. Next, an analysis of helical modes bound to partial defects with fractional Burgers vectors in $\mathcal{T}$-symmetric HOTIs was performed in SRef. 79; the analysis in SRef. 79 is complementary to, but does not overlap with, the results of the present work. Disclination states in TCIs and higher-order topological superconductors were recently explored in SRef. 202, and the presence of gapped helical modes in screw dislocations in $\mathcal{T}$-invariant stable HOTIs was also recently demonstrated in SRef. 203, but was not attributed to the (fragile or OAL) topological indices introduced in this work. Spinon excitations in corner-mode phases were also recently demonstrated in SRef. 204. Additionally, the possibility of topological 0D defect states in 3D insulators was briefly suggested in SRef. 205 as a means of completing the search for experimentally accessible defect states as classified by Teo and Kane [72]; the HEND states introduced in this work are consistent with this suggestion. A Wilson-loop formulation of partial nested Berry phase was also introduced during the preparation of this work in

SRef. 206; the authors of SRef. 206 use C_2 and $\mathcal{T}$ symmetries to obtain a formulation of partial nested Berry phase that is equivalent to the definition that we introduce in SN 3 B 3, but do not relate the new bulk topological quantity to the corner (defect) spectrum, as we do in this work. Closely related concentric Wilson loop invariants for $\mathcal{T}$- and rotation-symmetric insulators were also recently introduced in SRef. 207. During the preparation of this work, the authors of SRef. 208 analyzed the flux response of interacting $C_4 \times \mathcal{T}$-symmetric AXIs, which are distinct from the $\mathcal{I}$-symmetric AXIs and helical HOTIs considered in this work. Threaded magnetic flux was also recently theoretically proposed as a means of probing the sample-geometry dependence of the anomalous surface Dirac fermions of the TCI and HOTI SnTe [209]. Lastly, after the submission of this work, a semiclassical treatment of a spinor-axion response was explored in relation to HOTIs in SRef. 210.

2. MAPPING TOPOLOGY FROM MOMENTUM SPACE TO POSITION SPACE WITH DEFECTS AND π-FLUX

In this section, we will provide proofs demonstrating the relationship between the topology of pristine crystals and the electronic states bound to line-like defects and π-flux tubes. We will specifically show that dislocations with integer Burgers vectors [127] and threaded magnetic π-flux can map the momentum-space topology of lower-dimensional surfaces of the BZs of pristine crystals to surfaces in position space. We will analytically demonstrate that, under the preservation of specific symmetries, this mapping reveals the presence of anomalous 0D states bound to the ends of line-like dislocations and flux tubes in 3D insulators.

To reconcile our results with previous works, we will provide two alternative and equivalent sets of proofs demonstrating the aforementioned dislocation and π-flux topological mapping from momentum space to position space. First, in SN 2 A, building upon the "cutting" and "gluing" construction of topological defect states developed in SRef. 71 to predict dislocation helical modes in weak topological insulators [7–9, 211], we will employ $k \cdot p$ theory to predict HEND states in 3D crystals. Then, in SN 2 B, we will use more general arguments to demonstrate that edge dislocations and flux tubes in d-dimensional crystals can map $(d-1)$-dimensional [$(d-1)$-D] BZ surfaces to $(d-1)$-D real-space surfaces, leading in 3D crystals to the presence of 1D and 0D topological defect states. Specifically, in SN 2 B 1, we will prove that generalized edge dislocations in d-D, $\mathcal{I}$-symmetric crystals map the $(d-1)$-D BZ-surface topology of pristine crystals to the topology of $(d-1)$-D real-space surfaces between crystal defects, and in SN 2 B 2, we will employ similar arguments to analyze the topology of $\mathcal{I}$-symmetric arrangements of flux tubes in d-D crystals. Finally, in SN 2 B 3, we will extend the arguments in SN 2 B 1 and 2 B 2 to the experimentally relevant case of (1D) screw dislocations in 3D crystals.

A. Defect and Flux Bound States in 3D $\mathcal{I}$-Symmetric Insulators from $k \cdot p$ Theory

In this section, we will employ $k \cdot p$ theory to demonstrate the presence of HEND bound states on the corners and ends of crystal defects and flux tubes in two classes of HOTIs: inversion- ($\mathcal{I}$-) symmetric axion insulators (AXIs) [30–33, 96, 97, 100, 101, 106–110, 155, 172–175], which are 3D magnetic insulators with anomalous, gapped 2D surfaces and gapless hinges with 1D chiral modes [7, 8, 10, 13, 25, 33, 96, 106, 219–222], and time-reversal- ($\mathcal{T}$-) and $\mathcal{I}$-symmetric HOTIs [30, 100, 103–105] with anomalous, gapped ($\mathcal{T}$-symmetric) surfaces [25] and gapless hinges with 1D *helical* modes (see SN 2 A 3 for more details). We will also show that crystal defects, but crucially *not* π-flux tubes, bind anomalous HEND states in weak stacks of $\mathcal{I}$-symmetric *fragile* topological insulators (FTIs) [33, 70, 100, 107–110, 134–151] and obstructed atomic limits (OALs) [152–155].

Because AXIs and $\mathcal{I}$- and $\mathcal{T}$-symmetric HOTIs can be diagnosed by their bulk parity ($\mathcal{I}$) eigenvalues (through $\mathbb{Z}_2$- [7, 8, 10, 13, 33, 107–110, 155, 173–175, 219] and $\mathbb{Z}_4$-valued [100, 103–105] indices, respectively), then we can capture the bulk topology of AXIs and $\mathcal{I}$- and $\mathcal{T}$-symmetric HOTIs that differ from trivial insulators by (double) band-inversion at a time-reversal-invariant crystal momentum (TRIM point) $k_{\alpha,\beta,\gamma} = 0, \pi$ through $k \cdot p$ Hamiltonians. For simplicity, in this section, we will only consider crystals with orthorhombic lattice vectors [223], for which $\alpha, \beta, \gamma = x, y, z$, though our results can be extended to body- and face-centered geometries through generalizations of the BZ folding arguments in SRef. 70. First, in SN 2 A 1, we will extend the $k \cdot p$ construction from SRef. 71 to predict $\pm e/2$-charged HEND states bound to the corners of edge dislocations in AXIs and 3D weak FTIs. In SN 2 A 2, we will then adapt the arguments from SN 2 A 1 to also predict HEND states bound to the ends of screw dislocations in AXIs and 3D weak FTIs. Finally, in SN 2 A 3, we will further utilize the $k \cdot p$ construction in SN 2 A 1 and 2 A 2 to analyze flux threading in 3D insulators. In particular, in SN 2 A 3, we will show that the "wormhole" helical modes of strong TIs [164–170] with threaded π-flux evolve into anomalous HEND states in AXIs under the introduction of $\mathcal{I}$-symmetric magnetism.

An $\mathcal{I}$- and $\mathcal{T}$- symmetric HOTI can be formed by superposing a time-reversed pair of AXIs. Hence, the results derived in SN 2 A 1, 2 A 2, and 2 A 3 for AXIs (and $\mathcal{I}$-symmetric, $\mathcal{T}$-broken FTIs) can also be straightforwardly

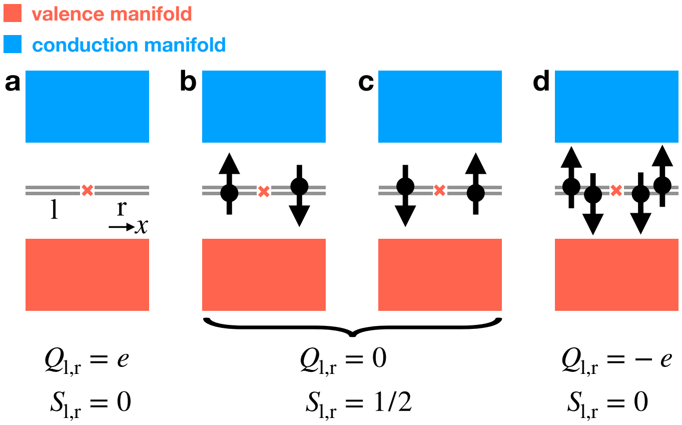

Supplementary Figure 1. **Spin-charge-separated Kramers pairs of defect or flux states.** $\boldsymbol{a-d}$ An inversion- ($\mathcal{I}$-) related pair of Kramers pairs of defect or flux states in a spinful, time-reversal- ($\mathcal{T}$-) symmetric insulator (where the $\mathcal{I}$ center is represented with a red $\times$ symbol in $\boldsymbol{a-d}$). $\boldsymbol{b, c}$ When the entire system lies at charge neutrality, each Kramers pair is filled by only a single electron, and therefore carries an excess chargeless spin-1/2 moment. Hence, at half filling and taking the spins of the electrons occupying each pair of states to point in opposite directions, $\mathcal{I}$ (which relates the positions of the Kramers pairs) and $\mathcal{T}$ symmetries are "softly" broken [33, 70, 212–214], and each half-filled Kramers pair of states forms an effective spinon quasiparticle with a free-angle spin-1/2 moment (depicted in $\boldsymbol{b, c}$ in configurations that preserve $\mathcal{I}\times\mathcal{T}$ symmetry). By $\boldsymbol{a}$ removing or $\boldsymbol{d}$ adding two electrons to the system (one electron per Kramers pair), we may realize a system configuration in which each Kramers pair respectively carries a net charge of $\pm e$ (taking electrons to carry a charge $-e$), but carries a net-zero spin. Hence, each Kramers pair of defect states either carries chargeless spin or spinless charge, and therefore exhibits the same reversed spin-charge relations as the solitons in polyacetylene [215–218].

extended to helical HOTIs (and $\mathcal{I}$- and $\mathcal{T}$-symmetric FTIs). Specifically, we will show that $\mathcal{I}$- and $\mathcal{T}$-symmetric insulators with crystal defects or threaded π-flux bind *Kramers pairs* of HEND states that are equivalent to higher-order generalizations [33, 70, 95, 100, 149] of the spin-charge separated solitons (*i.e.* spinons or *fluxons*) discussed in SRefs. 164–166, 169, 170, 215, 217, and 218.

To understand the spin-charge separation, we consider two $\mathcal{I}$-related pairs of topological defects or flux tubes in a 2D or 3D insulator that each binds a Kramers pair of 0D states (four degenerate single-particle states in total), taking each Kramers pair of states to be half-filled at charge neutrality [Supplementary Figure (SFig.) 1 $\boldsymbol{b, c}$], so that the ground state is degenerate. Enforcing $\mathcal{I}$ symmetry (where we have denoted a global $\mathcal{I}$ center with a red $\times$ symbol in SFig. 1), there is one filled state per Kramers pair. Hence, each Kramers pair carries a balanced (net-zero) charge, but necessarily "softly" breaks $\mathcal{T}$ symmetry, because each pair of states is filled with an unpaired spin-1/2 degree of freedom (without spin conservation symmetry such as s^z, however, the unpaired electron is not required to exhibit a quantized spin projection along a particular high-symmetry axis). When the system is doped away from charge neutrality by adding two more electrons in an $\mathcal{I}$-symmetric fashion, $\mathcal{I}$ and $\mathcal{T}$ symmetries can be satisfied (SFig. 1 $\boldsymbol{d}$). In the system configuration with two extra electrons, each fully-filled Kramers pair of states carries a charge $-e$ (taking electrons to have charge $-e$ measured from charge neutrality). Unlike in the previous system configuration with chargeless spin-1/2 0D states at zero doping in SFig. 1 $\boldsymbol{b, c}$, at a system doping of $-2e$, each Kramers pair of states is charged, but exhibits a net-zero spin, because $\mathcal{T}$ symmetry pairs electrons with reversed spins. In this $\mathcal{I}$- and $\mathcal{T}$-symmetric system configuration, the ground state is unique and has no degeneracy. Similarly, if we remove one electron from each Kramers pair of states in SFig. 1 $\boldsymbol{b, c}$, then we realize a system configuration in which there is a total charge of $+2e$, implying that each fully empty pair of states carries a charge $+e$ and does not carry an electron spin (SFig. 1 $\boldsymbol{a}$). Hence, the 0D Kramers pairs of states exhibit the same well-established spin-charge separation and reversed spin-charge relations as the solitons in polyacetylene [215–218].

1. *$k \cdot p$ Derivation of HEND States on Edge Dislocation Corners in AXIs and Weak Fragile TIs*

To derive the edge dislocation response of $\mathcal{I}$-symmetric 3D magnetic insulators, we will begin by formulating the $k \cdot p$ Hamiltonian of an $\mathcal{I}$- and $\mathcal{T}$-symmetric 3D insulator. After we complete this derivation, we will then relax $\mathcal{T}$ symmetry and observe the emergence of HEND states on edge dislocation corners. We consider the topology of the 3D $\mathcal{I}$- and $\mathcal{T}$-symmetric insulator to be fully captured by a set of band inversions at TRIM points between the two highest valence bands and the two lowest conduction bands [7, 8, 10], and therefore, for now, we exclude HOTIs and

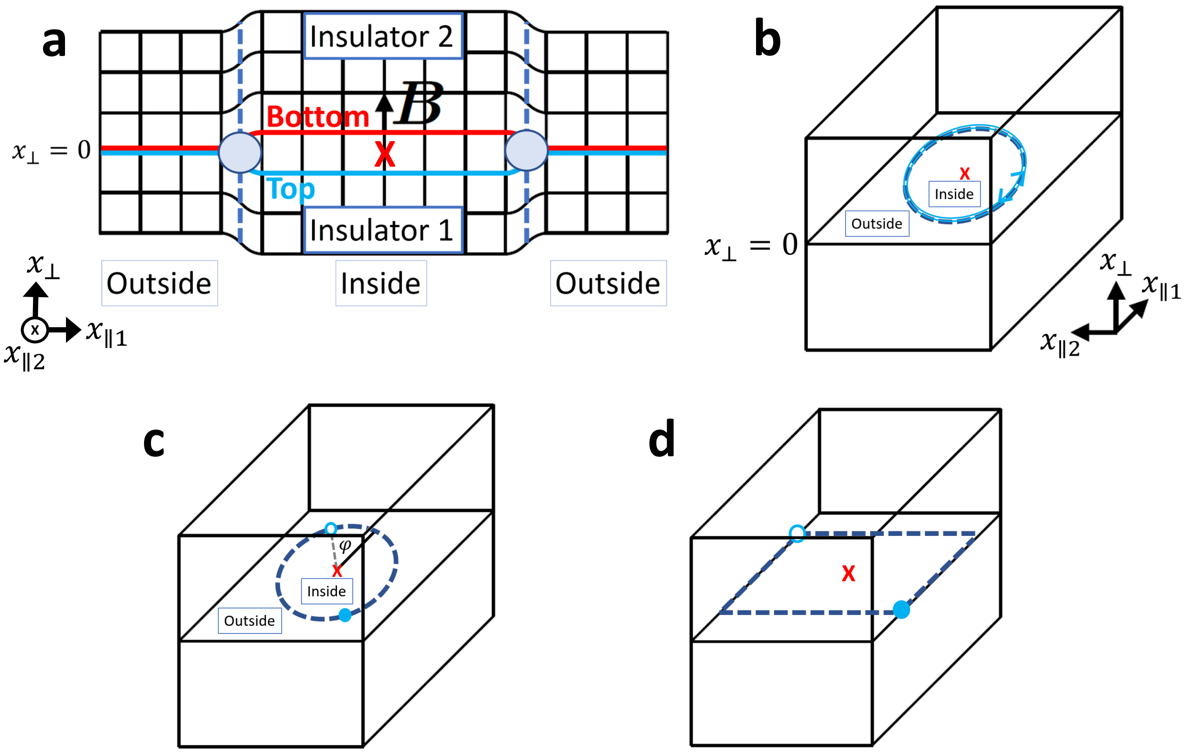

Supplementary Figure 2. **0D edge dislocation states in a 3D crystal.** ***a*** The Burgers vector $\boldsymbol{B}$ of the two dislocations (blue circles) has a length $|\boldsymbol{B}| = a_\perp$, the lattice spacing in the $x_\perp$ direction. Following the procedure in SRef. 71, we divide the system by cutting along the red, blue, and dashed lines. The red and blue lines separate two insulators with the same bulk topology, whose top and bottom surfaces are glued together with (inside) and without (outside) an additional row of unit cells. ***b*** A 3D crystal with an internal edge dislocation formed by taking the two dislocations in ***a*** and closing them into a circle in the $x_\perp = 0$ plane. As shown in SRefs. 71, 72, 75–77, and 80 and rederived in SEq. (35), if insulators 1,2 are inversion- ($\mathcal{I}$-) and time-reversal- ($\mathcal{T}$-) symmetric and feature nontrivial weak indices $\boldsymbol{M}_\nu$, then, if $\boldsymbol{B}\cdot\boldsymbol{M}_\nu \bmod 2\pi = \pi$, a helical pair of modes will be bound to the circular edge dislocation (dashed line) in ***b***. However, we can further extend the set of possible dislocation responses by requiring that an inversion center (red $\times$) is preserved in the plane surrounded by the dislocation. ***c*** In this case, under the relaxation of $\mathcal{T}$ symmetry in an $\mathcal{I}$-symmetric manner, the helical modes gap into $2+4n$ (filling) anomalous [33, 70, 100, 176] 0D states with charges $\pm e/2$ [SEq. (36)]. Specifically, the 0D states are equivalent to the corner modes of the 2D FTIs detailed in SRefs. 33 and 100. ***d*** We next smoothly grow the internal dislocation until it meets with the crystal boundary, at which point the edge dislocations coincide with exterior crystal surfaces, realizing an effective "stacking" defect within the region bounded by the edge dislocations (dashed lines). In ***d***, two of the four corners between edge dislocations bind $\pm e/2$-charged higher-order 0D (*i.e.* HEND) states. Because the process of breaking $\mathcal{T}$ symmetry while preserving $\mathcal{I}$ converts $\boldsymbol{M}_\nu$ to the weak fragile (and OAL) index $\boldsymbol{M}_\nu^{\mathrm{F}}$ [defined in SEq. (117) and the surrounding text in SN 3 B], then we conclude that anomalous HEND states are present in $\mathcal{T}$-broken, $\mathcal{I}$-symmetric 3D insulators with edge dislocations for which $\boldsymbol{B}\cdot\boldsymbol{M}_\nu^{\mathrm{F}} \bmod 2\pi = \pi$ [SEq. (40)].

weak FTIs formed from *double* band inversion at the same TRIM point [30, 31, 33, 100, 102, 104, 144]. Later, in SN 2 A 3, we will reintroduce more general symmetry and counting arguments that capture HOTIs formed through double band inversion.

The low-energy Hamiltonian of an $\mathcal{I}$-symmetric insulator can be expressed as:

$$\mathcal{H}(\boldsymbol{q}) = \bigoplus_a \mathcal{H}_a(\boldsymbol{q}), \tag{1}$$

where a runs over the TRIM points $\boldsymbol{k}_{D,a}$ whose bands are inverted relative to those of the atomic insulator formed from the occupied atomic orbitals when all hoppings are taken to vanish [152], and where $\boldsymbol{q} = \boldsymbol{k} - \boldsymbol{k}_{D,a}$. In anticipation of later breaking translation symmetry through the insertion of a crystal defect, we note that in Supplementary Equation (SEq.) (1), the summation is equivalent to folding the Hamiltonians of the band-inverted TRIM points onto a single k point and expanding in a $k\cdot p$ momentum range $\boldsymbol{q}$ around that point. We take the $k\cdot p$ Hamiltonian of each TRIM point $\boldsymbol{k}_{D,a}$ to have the form of the low-energy theory of the Bernevig-Hughes-Zhang (BHZ) model [3, 10, 13] of a 3D

TI (where we have neglected a $\boldsymbol{q}^2$ contribution to the bulk mass, because it does not affect the topology):

$$\mathcal{H}_a(\boldsymbol{q}) = m_a\tau^z + \sum_{i=x,y,z} v_i q_i \tau^x\sigma^i. \tag{2}$$

SEq. (2) is invariant under $\mathcal{I}$ and $\mathcal{T}$ symmetries, whose action we represent as:

$$\mathcal{I}:\ \mathcal{H}_a(\boldsymbol{q}) \to \tau^z\mathcal{H}_a(-\boldsymbol{q})\tau^z,\ \mathcal{T}:\ \mathcal{H}_a(\boldsymbol{q}) \to \sigma^y\mathcal{H}^*_a(-\boldsymbol{q})\sigma^y. \tag{3}$$

Next, we construct a long-wavelength description of a loop of edge dislocations whose Burgers vectors $\boldsymbol{B} = (B_x, B_y, B_z)$ lie along one of the lattice vectors (SFig. 2 ***a***), where $B_{x,y,z}$ are integer multiples of the lattice spacing. For the purposes of the following proof, we take $\boldsymbol{B} \parallel \hat{z}$, such that:

$$x_\perp = z,\ x_{\parallel 1} = x,\ x_{\parallel 2} = y, \tag{4}$$

in the notation of SFig. 2. As prescribed in SRef. 71, we model an internal loop of edge dislocations by cutting the insulator described by $\mathcal{H}(\boldsymbol{q})$ [SEq. (1)] into two pieces with $\pm\hat{z}$-normal surfaces, and then "gluing" the two pieces back together with and without extra rows of unit cells in the region (labeled "inside" in SFig. 2) between the edge dislocations.

Using the labeling in SFig. 2 ***a***, we begin by solving for the Hamiltonian of the top surface of insulator 1. We first form a Jackiw-Rebbi domain wall [70, 224] by taking the mass at each band-inverted bulk TRIM point to spatially vary $m_a \to m^T(z)$ [SEq. (2)]. Specifically, we take $m^T(z)$ to be large and negative for $z < z^T_\mu$ and large and positive for $z > z^T_\mu$, where z^T_μ is the z-coordinate of the top surface of insulator 1 in either the "inside" or "outside" region in SFig. 2, indexed by μ. This distribution of $m^T(z)$ can be summarized as:

$$\text{sgn}[m^T(z)] = \text{sgn}[z - z^T_\mu]. \tag{5}$$

Next, taking $q_x = q_y = 0$, we Fourier transform:

$$q_z \to -(\boldsymbol{k}_{D,a}\cdot\hat{z}) - i\partial_z, \tag{6}$$

and search for zero-energy, normalizable bound states $|\psi^T_{a,\mu}(z)\rangle$ that satisfy:

$$[m^T(z)\tau^z - v_z\tau^x\sigma^z(\boldsymbol{k}_{D,a}\cdot\hat{z} + i\partial_z)]|\psi^T_{a,\mu}(z)\rangle = 0. \tag{7}$$

SEq. (7) can be solved by left-multiplying by $\tau^x\sigma^z$ and integrating:

$$|\psi^T_{a,\mu}(z)\rangle_i = \frac{1}{\sqrt{N}}e^{i(\boldsymbol{k}_{D,a}\cdot\hat{z})(z-z^T_\mu)}e^{-\frac{1}{v_z}\int_{z^T_\mu}^{z} m^T(z')dz'}|+\rangle_i \equiv e^{i(\boldsymbol{k}_{D,a}\cdot\hat{z})(z-z^T_\mu)}\mathcal{Z}^T(z-z^T_\mu)|+\rangle_i, \tag{8}$$

where N is a normalization constant and $|+\rangle_i$ is an eigenstate of $\tau^y\sigma^z$ with eigenvalue +1(SFig. 2 ***a***):

$$|+\rangle_1 = \frac{1}{\sqrt{2}}\begin{pmatrix}0\\ i\\ 0\\ 1\end{pmatrix},\ |+\rangle_2 = \frac{1}{\sqrt{2}}\begin{pmatrix}-i\\ 0\\ 1\\ 0\end{pmatrix}. \tag{9}$$

To restore dispersion in the $q_{x,y}$ directions, we follow the procedure employed in SRefs. 70 and 102 for higher-order TIs and semimetals and project the linear terms in SEq. (2) into the basis of $|\psi^T_{a,\mu}(z)\rangle_i$:

$$[\mathcal{H}^T_{a,\mu}(q_x,q_y)]_{ij} = {}_i\langle\psi^T_{a,\mu}(z)|v_xq_x\tau^x\sigma^x + v_yq_y\tau^x\sigma^y|\psi^T_{a,\mu}(z)\rangle_j = \int_{-\infty}^{\infty} dz|\mathcal{Z}^T(z-z^T_\mu)|^2\,{}_i\langle+|\mathcal{H}_a(q_x,q_y,0)|+\rangle_j,$$
$$\mathcal{H}^T_{a,\mu}(q_x,q_y) = v_xq_xs^y + v_yq_ys^x, \tag{10}$$

where s is a matrix in the 2×2 basis of $|+\rangle_{1,2}$, and where we have exploited that $|\mathcal{Z}^T(z-z^T_\mu)|^2 \to \delta(z-z^T_\mu)$ if $m(z)$ rapidly saturates at a large value relative to [70, 224] v_z. Though SEq. (10) is actually exact, because $\mathcal{Z}^T(z-z^T_\mu)$ is already normalized in SEq. (8), we will find the approximation $\mathcal{Z}^T(z-z^T_\mu) \sim \sqrt{\delta(z-z^T_\mu)}$ useful for subsequent calculations. $\mathcal{H}^T_{a,\mu}(q_x,q_y)$ has the expected form of the $k\cdot p$ Hamiltonian of the linear (twofold Dirac) surface fermion of a 3D TI [225], and correspondingly, respects $\mathcal{T}$ symmetry, here represented by the action:

$$\mathcal{T}:\ \mathcal{H}^T_{a,\mu}(q_x,q_y) \to s^y[\mathcal{H}^T_{a,\mu}(-q_x,-q_y)]^*s^y. \tag{11}$$

Next, we solve for the bottom surface Hamiltonian of the insulator described by SEq. (1). The derivation is equivalent to the previous derivation for the top surface up to the replacement $m^T(z) \to m^B(z) = m^T(-z)$ (where we have chosen the origin of the z coordinate such that $z^B_\mu = -z^T_\mu$). Then,

$$\text{sgn}[m^B(z)] = \text{sgn}[-z + z^B_\mu]. \tag{12}$$

Due to the change in sign of the z coordinate, the normalizable solutions for the bottom surface are instead proportional to eigenstates of $\tau^y\sigma^z$ with eigenvalue -1, as opposed to the eigenstates with eigenvalue $+1$ previously obtained for the top surface Hamiltonian [SEq. (9)]. For the bottom surface states, we obtain:

$$|\psi^B_{a,\mu}(z)\rangle_i = \frac{1}{\sqrt{N}} e^{i(\boldsymbol{k}_{D,a}\cdot\hat{z})(z-z^B_\mu)} e^{+\frac{1}{v_z}\int_{z^B_\mu}^{z} m^B(z')dz'} |-\rangle_i = e^{i(\boldsymbol{k}_{D,a}\cdot\hat{z})(z-z^B_\mu)} \mathcal{Z}^B(z - z^B_\mu)|-\rangle_i, \tag{13}$$

where N is a normalization constant and $|-\rangle_i$ is an eigenstate of $\tau^y\sigma^z$ with eigenvalue -1 (SFig. 2 ***a***):

$$|-\rangle_1 = \frac{1}{\sqrt{2}}\begin{pmatrix} 0 \\ i \\ 0 \\ -1 \end{pmatrix},\ |-\rangle_2 = \frac{1}{\sqrt{2}}\begin{pmatrix} -i \\ 0 \\ -1 \\ 0 \end{pmatrix}. \tag{14}$$

To restore dispersion in the $q_{x,y}$ directions, we follow the procedure in SEq. (10) and project the linear terms in SEq. (2) into the basis of the bottom surfaces states $|\psi^B_{a,\mu}(z)\rangle_i$:

$$\mathcal{H}^B_{a,\mu}(q_x, q_y) = -v_x q_x s^y - v_y q_y s^x = -\mathcal{H}^T_{a,\mu}(q_x, q_y), \tag{15}$$

where s is a matrix in the 2×2 basis of $|-\rangle_{1,2}$. Like $\mathcal{H}^T_{a,\mu}(q_x, q_y)$, $\mathcal{H}^B_{a,\mu}(q_x, q_y)$ respects $\mathcal{T}$ symmetry, which is represented by the same action as in SEq. (11).

Now that we have established the forms of the top- and bottom-surface Hamiltonians [SEqs. (10) and (15)], we next explicitly form an $\mathcal{I}$-symmetric interface between the top and bottom of two insulators that each exhibit the same bulk band ordering and topology as SEq. (1) (SFig. 2 ***a***). This is equivalent to cutting a single copy of this insulator into two halves [71] related by $\mathcal{I}$ symmetry (red $\times$ in SFig. 2). Redefining the z coordinates, we take the inversion center of the cut region to lie at $z = 0$, and consider two regions: an "outside" ($\mu = out$) region in which the top and bottom surfaces are flush with each other, and an "inside" ($\mu = in$) region in which there are $|\boldsymbol{B}|$ extra unit cells in the z direction between the top and bottom surfaces of the two insulators (SFig. 2 ***a***). The 1D interface between the "inside," "outside," "top," and "bottom" regions in SFig. 2 ***a*** is therefore spanned by edge dislocations with Burgers vector $\boldsymbol{B}$. Before allowing coupling between the top and bottom surfaces, we combine the two Kramers pairs of linear surface fermions in SEqs. (10) and (15) in both the "inside" and "outside" regions to form the Hamiltonian of a fourfold-degenerate, 2D Dirac fermion [25, 226–228]:

$$\mathcal{H}^D_{a,\mu}(q_x, q_y) = v_x q_x \xi^z s^y + v_y q_y \xi^z s^x, \tag{16}$$

where ξ is a matrix that acts on the "top" and "bottom" degrees of freedom and s continues to act on the indices $i = 1, 2$ in $|\pm\rangle_i$ within each of the two surfaces of the insulator described by SEq. (1). In this geometry (SFig. 2 ***a***), $\mathcal{I}$ is represented through the action:

$$\mathcal{I}:\ \mathcal{H}^D_{a,\mu}(q_x, q_y) \to \xi^x \mathcal{H}^D_{a,\mu}(-q_x, -q_y)\xi^x. \tag{17}$$

SEq. (16) also remains invariant under $\mathcal{T}$ symmetry, which is represented in the 4×4 basis of $\xi \otimes s$ by:

$$\mathcal{T}:\ \mathcal{H}^D_{a,\mu}(q_x, q_y) \to s^y[\mathcal{H}^D_{a,\mu}(-q_x, -q_y)]^* s^y. \tag{18}$$

Finally, we allow for $\mathcal{I}$- and $\mathcal{T}$-symmetric coupling between the top and bottom surfaces. Because $\mathcal{H}^D_{a,\mu}(q_x, q_y)$ in SEq. (16) is equivalent to the Hamiltonian of a fourfold Dirac fermion in 2D [25, 226–228], then $\mathcal{H}^D_{a,\mu}(q_x, q_y)$ admits a single, $\mathcal{T}$-symmetric, anticommuting mass term, which here is proportional to ξ^x. To account for the presence or absence of nonzero $\boldsymbol{B}$ in the regions indexed by μ, we derive a consistent phase for the coupling mass by fixing the defect displacements to be centered about the interface at $z = 0$ (SFig. 2 ***a***). We then define a spinless interface coupling potential:

$$[V^{\text{C}}_{a,\mu}]^{TB}_{ij} = m_{\text{C}}\delta_{ij}\int_{-\infty}^{\infty} dz\ \left[|\psi^T_{a,\mu}(z)\rangle_i\langle\psi^B_{a,\mu}(z)|_j + \text{h.c.}\right],$$
$$[V^{\text{C}}_{a,\mu}]^{TT}_{ij} = [V^{\text{C}}_{a,\mu}]^{BB}_{ij} = 0, \tag{19}$$

where $m_{\rm C}$ is the strength of the coupling; $i,j=1,2$; and δ_{ij} enforces $\mathcal{T}$ symmetry [SEq. (18)] by excluding magnetic coupling interactions (for now). Substituting SEqs. (8) and (13) into SEq. (19), we arrive at the more specific expression:

$$[V^{\rm C}_{a,\mu}]^{TB}_{ij} = m_{\rm C}\delta_{ij}e^{i(\boldsymbol{k}_{D,a}\cdot\hat{z})(z^B_\mu - z^T_\mu)}\left[\int_{-\infty}^{\infty} dz\ \mathcal{Z}^T(z-z^T_\mu)\mathcal{Z}^B(z-z^B_\mu)\right]|+\rangle_{ij}\langle-| + \text{h.c.}. \tag{20}$$

In the $\mu = out$ region, for which:

$$z^T_{out} = z^B_{out} = 0, \tag{21}$$

SEq. (20) greatly simplifies:

$$[V^{\rm C}_{a,out}]^{TB}_{ij} = m_{\rm C}\delta_{ij}\int_{-\infty}^{\infty} dz\ \delta(z) = m_{\rm C}\delta_{ij},$$
$$V^{\rm C}_{a,out} = m_{\rm C}\xi^x, \tag{22}$$

for which we have exploited that both $\mathcal{Z}^{T,B}(z)$ can be taken to go to zero sufficiently rapidly away from $z=0$ [see the text after SEq. (10)] to approximate that $[\mathcal{Z}^{T,B}(z)]^2 \to \delta(z)$. For the $\mu = in$ region, however, for which:

$$z^T_{in} = -|\boldsymbol{B}|/2,\ z^B_{in} = |\boldsymbol{B}|/2, \tag{23}$$

SEq. (20) acquires an additional phase:

$$[V^{\rm C}_{a,in}]^{TB}_{ij} = m_{\rm C}\delta_{ij}e^{i(\boldsymbol{k}_{D,a}\cdot\hat{z})|\boldsymbol{B}|}\int_{-\infty}^{\infty} dz\ C\delta(z), \tag{24}$$

where we have approximated $\mathcal{Z}^T(z+|\boldsymbol{B}|/2)\mathcal{Z}^B(z-|\boldsymbol{B}|/2)$ as a single delta function localized at $z=0$ weighted by the real constant $C\geq 0$, which captures the overlap between the two offset wavefunctions $\mathcal{Z}^{T,B}(z)$. This approximation is valid if $|\boldsymbol{B}|$ is small compared to the decay lengths of $\mathcal{Z}^{T,B}(z)$. While the limit of SEq. (24) necessitates that $C<1$ (and possibly $C\ll 1$), because $|\mathcal{Z}^{T,B}(z)|^2$ was earlier approximated as a delta function $\delta(z)$, we can still take $m_{\rm C}C$ to be sufficiently large compared to the dispersions $v_{x,y}$ in SEq. (16) to ensure that higher-order, nested Jackiw-Rebbi domain-wall constructions [70, 100] remain valid predictors of 1D (and 0D) bound states. Next, as we are specializing to integer Burgers vectors, $\mathcal{I}$ and $\mathcal{T}$ symmetry as represented in SEqs. (17) and (18), respectively, lead to the restriction that SEq. (24) only admits real phases:

$$V^{\rm C}_{a,in} = m_{\rm C}C\cos\left(\boldsymbol{k}_{D,a}\cdot\boldsymbol{B}\right)\xi^x, \tag{25}$$

where we have used the previous simplification that $\boldsymbol{B} = |\boldsymbol{B}|\hat{z}$ to reexpress the argument of the cosine in its more general form. During the long preparation of this manuscript, a discussion of topological response effects in HOTIs with partial defects characterized by fractional Burgers vectors appeared in SRef. 79; we will not further address the case of fractional $|\boldsymbol{B}|$ in the present work.

We will now briefly discuss the bulk origin of $[V^{\rm C}_{a,\mu}]^{TB}_{ij}$. First, the top and bottom surface states are given by SEqs. (8) and (13), respectively. Projecting the bulk Hamiltonian into the interface basis spanned by $|\psi_{a,\mu}(z)\rangle_\alpha = \{|\psi^T_{a,\mu}(z)\rangle_1, |\psi^T_{a,\mu}(z)\rangle_2, |\psi^B_{a,\mu}(z)\rangle_1, |\psi^B_{a,\mu}(z)\rangle_2\}_\alpha$, $\alpha = 1\ldots 4$, we obtain:

$$\begin{aligned}\left[\mathcal{H}^{\rm C}_{a,\mu}(q_x,q_y)\right]_{\alpha\beta} &= \int_{-\infty}^{\infty} {}_\alpha\langle\psi_{a,\mu}(z)|\mathcal{H}_a(q_x,q_y,0)|\psi_{a,\mu}(z)\rangle_\beta\, dz \\ &= \left[\xi^z\left(v_xq_xs^y + v_yq_ys^x\right) + m_a\xi^x\left(\delta_{\mu,out} + C\delta_{\mu,in}e^{i(\boldsymbol{k}_{D,a}\cdot\hat{z})(z^T_\mu - z^B_\mu)}\right)\right]_{\alpha\beta},\end{aligned} \tag{26}$$

where we have taken the 4×4 matrices $\xi^i s^j$, $i,j = 0,x,y,z$, to act on the basis indexed by $\alpha = 1\ldots 4$. This recovers our earlier result: $\mathcal{H}^{\rm C}_{a,\mu}(q_x,q_y) = \mathcal{H}^{\rm D}_{a,\mu}(q_x,q_y) + V^{\rm C}_{a,\mu}$. From SEq. (26), we identify the bulk origin of the coupling mass as $m_{\rm C} = m_a$ (*i.e.*, the BHZ mass at $\boldsymbol{k}_{D,a}$).

With SEqs. (22) and (25) established, we will now determine the conditions under which defect bound states are present. We consider an internal edge dislocation at $z=0$ with a circular geometry and radius R (SFig. 2 ***b***). The geometry of the circular dislocation is more naturally addressed in cylindrical coordinates, and so we first Fourier transform $\mathcal{H}^D_a(q_x,q_y)$ [SEq. (16)] by taking $q_{x,y}\to -i\partial_{x,y}$, where we have suppressed (for the in-plane coordinates

x, y) the factors of $\boldsymbol{k}_{D,a}$ that previously appeared in SEq. (6), because they will ultimately only contribute to gauge-dependent phases that cancel when physical observables (*e.g.* bound state locations and charges) are calculated. We then convert to cylindrical coordinates:

$$\partial_x = \cos(\varphi)\partial_r - \frac{1}{r}\sin(\varphi)\partial_\varphi,$$
$$\partial_y = \sin(\varphi)\partial_r + \frac{1}{r}\cos(\varphi)\partial_\varphi, \tag{27}$$

such that SEq. (16), taking the isotropic limit that $v_x = v_y = v$, now takes the form:

$$\mathcal{H}^D_a(r,\varphi) = -iv\xi^z\left(s^1(\varphi)\partial_r + \frac{1}{r}s^2(\varphi)\partial_\varphi\right), \tag{28}$$

where:

$$s^1(\varphi) = \sin(\varphi)s^x + \cos(\varphi)s^y = \begin{pmatrix} 0 & -ie^{i\varphi} \\ ie^{-i\varphi} & 0 \end{pmatrix},$$
$$s^2(\varphi) = \cos(\varphi)s^x - \sin(\varphi)s^y = \begin{pmatrix} 0 & e^{i\varphi} \\ e^{-i\varphi} & 0 \end{pmatrix},$$
$$\{s^1(\varphi), s^2(\varphi)\} = 0,\ \ s^1(\varphi)s^2(\varphi) = -is^z. \tag{29}$$

The "inside" and "outside" index μ has been suppressed in SEq. (28); we instead capture μ through the introduction of an r-dependent coupling mass between the top and bottom surfaces inside and outside of the defect:

$$V^{\mathrm{C}}_a(r,\varphi) = m_{\mathrm{C}}(r)\xi^x, \tag{30}$$

where $m_{\mathrm{C}}(r)$ is large and positive for $r > R$ and large with a sign given by $\cos(\boldsymbol{k}_{D,a}\cdot\boldsymbol{B})$ for $r < R$ compared to the other parameters in the Hamiltonian [SEq. (25)]. We then form the combined Hamiltonian:

$$\mathcal{H}^C_a(r,\varphi) = \mathcal{H}^D_a(r,\varphi) + V^{\mathrm{C}}_a(r,\varphi), \tag{31}$$

which is invariant under the cylindrical-coordinate representations of $\mathcal{I}$ and $\mathcal{T}$ symmetry:

$$\mathcal{I}:\ \mathcal{H}^C_a(r,\varphi) \to \xi^x\mathcal{H}^C_a(r,\varphi+\pi)\xi^x,\ \ \mathcal{T}:\ \mathcal{H}^C_a(r,\varphi) \to s^y(\mathcal{H}^C_a(r,\varphi))^* s^y. \tag{32}$$

Because SEq. (31) is equivalent to the low-energy theory of a 2D TI [3, 5, 6] (or a gapped anomalous fourfold Dirac surface fermion [25]), domain walls between regions with opposite signs of the $\mathcal{T}$-symmetric mass ξ^x will bind 1D helical modes. In the geometry in SFig. 2 ***b***, such a domain wall lies at $r = R$ if $\cos(\boldsymbol{k}_{D,a}\cdot\boldsymbol{B}) = -1$. This implies that each band-inverted bulk TRIM point $\boldsymbol{k}_{D,a}$ will only contribute helical modes to R if $\boldsymbol{k}_{D,a}\cdot\boldsymbol{B}$ is an odd multiple of π. For example, for the Burgers vector in this section $\boldsymbol{B} = |\boldsymbol{B}|\hat{z}$, only TRIM points in the $k_z = \pi$ plane can possibly contribute helical modes. Recalling that SEq. (1), the full position-space Hamiltonian at $z = 0$ is given by:

$$\mathcal{H}^C(r,\varphi) = \bigoplus_a \mathcal{H}^C_a(r,\varphi), \tag{33}$$

we determine that the total number of pairs of helical modes bound at $r = R$ is given by the total number of band-inverted bulk TRIM points for which:

$$\boldsymbol{k}_{D,a}\cdot\boldsymbol{B} = \pi \bmod 2\pi. \tag{34}$$

SEq. (34) implies that only edge dislocations with Burgers vectors equal to odd-integer lattice translations and band inversions at TRIM points away from Γ ($k_x = k_y = k_z = 0$) can contribute to dislocation helical modes. Furthermore, because an even number of pairs of 1D helical modes on the edge of a 2D insulator can be pairwise pushed into the valence and conduction manifolds, absent additional crystal symmetries (*i.e.* mirror reflections) [6, 8, 15], then we further require that an odd number of TRIM points satisfy SEq. (34) in order for anomalous helical modes to be bound at $r = R$. Taken together, this implies that if $\boldsymbol{B}$ contains an odd number of primitive lattice vectors in the k_i direction, then the Hamiltonian of the $k_i = \pi$ plane must differ from that of an atomic insulator by an odd number of band inversions between Kramers pairs of bands with opposite parity eigenvalues, implying that the weak index M_i is nontrivial [7, 8] [where M_i counts the number of band inversions per Kramers pair in the BZ plane satisfying

SEq. (34)]. From this, we recover the main result of SRefs. 71, 72, 75–77, and 80: an $\mathcal{I}$- and $\mathcal{T}$-symmetric insulator with an edge dislocation with an integer Burgers vector $\boldsymbol{B}$ will bind anomalous helical modes along the dislocation if:

$$\boldsymbol{B}\cdot\boldsymbol{M}_{\nu} \bmod 2\pi = \pi. \tag{35}$$

We note that $\mathcal{I}$ symmetry is not required for this result to hold; $\mathcal{T}$ symmetry alone is sufficient. However, exploiting recent advances in higher-order (polarization) [30, 32, 33, 70, 95, 98–100, 141–143, 145–149, 156, 176, 229–234] and fragile [33, 70, 100, 107–110, 134–151] topology, we will show below that *filling anomalies* (SRefs. 33, 70, 100, and 176 and SN 4) can be exploited to further extend SEq. (35).

Beginning with $\mathcal{H}^{C}_{a}(r,\varphi)$ [SEq. (31)], we now relax $\mathcal{T}$ symmetry while preserving $\mathcal{I}$ symmetry [SEq. (32)]. Without $\mathcal{T}$ symmetry, SEq. (31) admits a set of $\mathcal{I}$-symmetric, r-independent masses that gap all edge states [70]:

$$V^{A}_{a}(r,\varphi) = \sum_{n,F}\sum_{\bar{\gamma}_n} m_{n,F,\bar{\gamma}_n}\Gamma_{\mathrm{F}}\cos(L^{\mathrm{FTI}}_{z,n}\varphi+\bar{\gamma}_n), \tag{36}$$

where:

$$L^{\mathrm{FTI}}_{z,n} = 1+2n \text{ where } n\in\{\mathbb{Z}^{+},0\},\ \Gamma_{\mathrm{F}} = \xi^{y},\ \xi^{z}s^{z}, \tag{37}$$

$\bar{\gamma}_n$ is a free angle, and the sum in SEq. (36) runs over all possible-symmetry allowed values of angular momenta ($L^{\mathrm{FTI}}_{z,n}$), angles ($\bar{\gamma}_n$) per angular momentum, and Dirac matrices (Γ_{F}). As rigorously shown in SRefs. 33, 70, and 100, a Hamiltonian of the form:

$$\mathcal{H}^{A}_{a}(r,\varphi) = \mathcal{H}^{C}_{a}(r,\varphi) + V^{A}_{a}(r,\varphi), \tag{38}$$

binds singly-degenerate, 0D (anti)solitons at the zeroes of $\cos(L^{\mathrm{FTI}}_{z,n}\varphi+\bar{\gamma}_n)$, which respectively acquire charges $\pm e/2$ under the "soft" relaxation of $\mathcal{I}$ symmetry [33, 70, 212–214] (SFig. 2 ***c***). Because $\cos(L^{\mathrm{FTI}}_{z,n}\varphi+\bar{\gamma}_n)$ has $2L^{\mathrm{FTI}}_{z,n}$ zeroes on a circle, and $2L^{\mathrm{FTI}}_{z,n} = 4n+2$ (anti)solitons are necessarily filling-anomalous under the presence of $\mathcal{I}$ symmetry (SRefs. 33, 70, 100, and 176 and SN 4), then SEq. (38) necessarily implies that each of the bulk TRIM points that previously contributed a pair of helical modes at $r=R$ through SEq. (34) will necessarily *now* contribute an anomalous number of 0D (anti)solitons at $r=R$ under the introduction of $\mathcal{I}$-symmetric magnetism. As shown in SRefs. 33, 70, 100, and 176, this conclusion is crucially *not* reliant on particle-hole symmetry, which is not present in real materials [131, 132]. The presence of $\bar{\gamma}_n$ in SEq. (38) reflects that, until sharp "corners" are externally imposed, the anomalous 0D boundary modes are free to lie at any $\mathcal{I}$-related pair of angles φ and $\varphi+\pi$ [SEq. (32)] along $r=R$, because the 2D (layer [25, 227, 228]) point group with only $\mathcal{I}$ symmetry has no fixed points (angles) on the boundary of a circle [235–239]. The effects of curvature on 0D boundary solitons in a cylindrical geometry are explored in detail in SRef. 70. As previously with SEqs. (1) and (33), the full, $\mathcal{T}$-broken position-space Hamiltonian at $z=0$ is:

$$\mathcal{H}^{A}(r,\varphi) = \bigoplus_{a}\mathcal{H}^{A}_{a}(r,\varphi), \tag{39}$$

implying that the total number of 0D boundary states is given by the summed contributions of each of the TRIM points $\boldsymbol{k}_{D,a}$ that previously contributed helical modes at $r=R$ before $\mathcal{T}$ was relaxed [SEq. (34)]. This result can be understood by recognizing that SEq. (31) is equivalent to the $k\cdot p$ Hamiltonian of an $\mathcal{I}$- and $\mathcal{T}$-symmetric 2D TI [3, 5, 6], and that $\mathcal{I}$- and $\mathcal{T}$-symmetric 2D TIs transition into 2D magnetic FTIs with anomalous corner modes under the application of $\mathcal{I}$-symmetric magnetism [33, 100].

Next, absent additional rotation [30, 70, 95, 176] or magnetic antiunitary [33, 100, 107–110, 149] symmetries, two superposed copies of the $k\cdot p$ Hamiltonian in SEq. (38) combine to contribute a non-anomalous number of 0D boundary states [33, 100, 176]. Hence, we conclude that an $\mathcal{I}$-symmetric, $\mathcal{T}$-broken 3D insulator with a closed set of $\mathcal{I}$-symmetric edge dislocations with Burgers vector $\boldsymbol{B}$ will bind anomalous 0D states at $\mathcal{I}$-related locations along the set of dislocations if:

$$\boldsymbol{B}\cdot\boldsymbol{M}^{\mathrm{F}}_{\nu} \bmod 2\pi = \pi, \tag{40}$$

where $\boldsymbol{M}^{\mathrm{F}}_{\nu}$ is a new weak index vector characterizing which of the BZ-boundary planes host Hamiltonians that are topologically equivalent to the $\mathcal{I}$-symmetric FTI characterized in SRefs. 33 and 100, or the obstructed atomic limit (OAL) that results from adding trivial bands without anomalous corner charges. Specifically, in the case in which the FTI is trivialized to an OAL with corner charges, the valence manifold on the BZ boundary is instead composed of fully trivial bands (unobstructed atomic limits) and fragile bands that as a whole combine to form a (Wannierizable) 2D OAL [70, 100, 134–137, 141–143, 145–152, 240]; nevertheless, in this case, $\boldsymbol{M}^{\mathrm{F}}_{\nu}$ remains a valid

indicator of the higher-order dislocation (HEND state) response derived in this section (see SN 3 B for an explicit numerical demonstration).

In SN 3, we formally define $\boldsymbol{M}_\nu^{\mathrm{F}}$ in terms of elementary band representations (EBRs) [135, 137, 152–155, 241–243] and nested Wilson loops [23–25, 30, 33, 70, 95, 100, 137, 149, 244–246]. In SN 3 B 2, we show that, because the anomalous solitons of SEq. (38) can remain anomalous if doubled under $\mathcal{T}$ symmetry, then there also exists a $\mathcal{T}$-symmetric generalization of SEq. (40) that predicts whether or not edge dislocations in $\mathcal{I}$- and $\mathcal{T}$-symmetric crystals bind Kramers pairs of 0D states. We emphasize that $\boldsymbol{M}_\nu^{\mathrm{F}}$ does not distinguish between 3D insulators for which both the $k_i = 0, \pi$ planes have topologically equivalent 2D Hamiltonians and insulators for which k_i indexes a pumping cycle between a 2D trivial insulator at $k_i = 0$ and a 2D FTI at $k_i = \pi$ [33, 107–110]. Furthermore, FTIs can be trivialized by the introduction of appropriately chosen trivial bands [33, 70, 100, 107–110, 134–151]. Therefore, we conclude that the dislocation response of 3D insulators does not distinguish between strong and weak (fragile) topology. This implies that 3D insulators may still exhibit an anomalous crystal defect response even if they are Wannierizable (*i.e.*, a 3D OAL) [135, 137, 152–155, 243, 244, 247–251]. This is in stark contrast to the flux response, which we will show in SN 2 A 3 and 2 B 2 to only be anomalous in strong topological (crystalline) insulators. Finally, to connect the circular internal edge dislocation used throughout this section (SFig. 2 ***c***) to surface-terminating edge dislocations in real materials, we imagine smoothly growing the internal dislocation until it meets the sample boundary, and only then creating sharp corners between the dislocations and the surface (SFig. 2 ***d***). In this geometry, the ends (corners) of the surface-facing edge dislocations (which now appear as surface point dislocations, see SFig. 2 ***d***), will carry higher-order 0D (HEND) states, consistent with our calculations of the dislocation bound states in tight-binding models (SN 4) and in 3D SnTe crystals (SN 6 B).

2. $k \cdot p$ Derivation of HEND States on Screw Dislocation Ends in AXIs and Weak Fragile TIs

In this section, we will adapt the arguments derived in the previous section for HEND states bound to edge dislocations to analyze the electronic structure of screw dislocations in AXIs and 3D weak FTIs. To begin, we again consider, as previously in SN 2 A 1, a 3D insulator with $\mathcal{I}$ and $\mathcal{T}$ symmetries that differs from an atomic insulator by a series of band inversions at the Fermi energy between only the two highest valence bands and the two lowest conduction bands. We take these band inversions to occur at a set of TRIM points $\boldsymbol{k}_{D,a}$ between Kramers pairs of states with opposite parity eigenvalues. The Hamiltonian of the bulk insulator, when pristine, is given by SEqs. (1) and (2). Next, we insert a pair of screw dislocations with Burgers vector $\boldsymbol{B}$ (SFig. 3 ***a***), where $|\boldsymbol{B}|$ is an integer multiple of the lattice spacing in the $\hat{B}$ direction. For this section, unlike previously for the edge dislocations in SN 2 A 1, we take $\boldsymbol{B} \parallel \hat{y}$, such that:

$$x_\perp = z,\ x_{\parallel 1} = x,\ x_{\parallel 2} = y, \tag{41}$$

in the notation of SFig. 3.

We will find it useful to briefly introduce the notion of screw chirality. For a screw dislocation, the direction of net displacement is indicated by a sense vector [127] $\boldsymbol{s}$ that is parallel to $\boldsymbol{B}$. The chirality C of a particular screw dislocation is then indicated by:

$$\mathsf{C} = \mathrm{sgn}[\boldsymbol{B} \cdot \boldsymbol{s}]. \tag{42}$$

We take the screw dislocation on the left (right) in SFig. 3 ***a*** to have a sense vector $\boldsymbol{s}$ that points in the $\hat{y}$ $(-\hat{y})$ direction, such that the screw on the left (right) is right- (left-) handed. If the insertion of the screw dislocations preserves an inversion center (red $\times$ in SFig. 3), then the action of $\mathcal{I}$ exchanges the locations and chiralities of the two screws, in agreement with well-established notion that $\boldsymbol{s}$ transforms as a vector ($\mathcal{I}$-odd) and $\boldsymbol{B}$ transforms as a pseudovector ($\mathcal{I}$-even), such that C transforms as a pseudoscalar [127].

As was previously done in SN 2 A 1, we model the pair of screw dislocations by cutting the insulator described by $\mathcal{H}(\boldsymbol{q})$ [SEq. (1)] into two pieces with $\pm\hat{z}$-normal surfaces, and then "gluing" the pieces back together. However, instead of inserting an extra row of unit cells in the $\hat{z}$ direction to create edge dislocations, as we did previously in SFig. 2 ***a***, we implement a pair of screw dislocations by gluing the upper portion of the cut insulator (labeled insulator 2 in SFig. 3 ***a***) to the lower portion (labeled insulator 1) with an extra relative displacement of $\boldsymbol{B}$ along the y direction in the region between the two insulators (labeled "inside" in SFig. 3). To preserve $\mathcal{I}$ symmetry, we implement this displacement evenly between the two insulators, such that insulator 1 (2) is displaced by $|\boldsymbol{B}/2|$ in the $-y$ $(+y)$ direction (a 3D rendering of this geometry, viewed from the exterior of the crystal with screw dislocations, is provided in SFig. 3 ***b***).

We next solve for the top surface states of insulator 1. Here, because the crystal with the two screw dislocations still preserves y direction lattice translation at this stage of the calculation (SFig. 3 ***a***), then the analysis from the

previous section [SEqs. (5) through (10)] can still be used with only minor modifications. To determine the effect of the screw dislocations on the electronic structure, we begin with the original bound states in SEq. (8), and then, setting $z_\mu^T = 0$, act with $\hat{T}_{y_\mu^T}$, the y direction lattice translation operator. The operation of translation $\hat{T}_{y_\mu^T}$ results in a phase shift of the original states in SEq. (8):

$$|\psi^T_{a,\mu}(z)\rangle_i = e^{i(\boldsymbol{k}_{D,a}\cdot\hat{y})y_\mu^T} e^{i(\boldsymbol{k}_{D,a}\cdot\hat{z})z}\mathcal{Z}^T(z)|+\rangle_i, \tag{43}$$

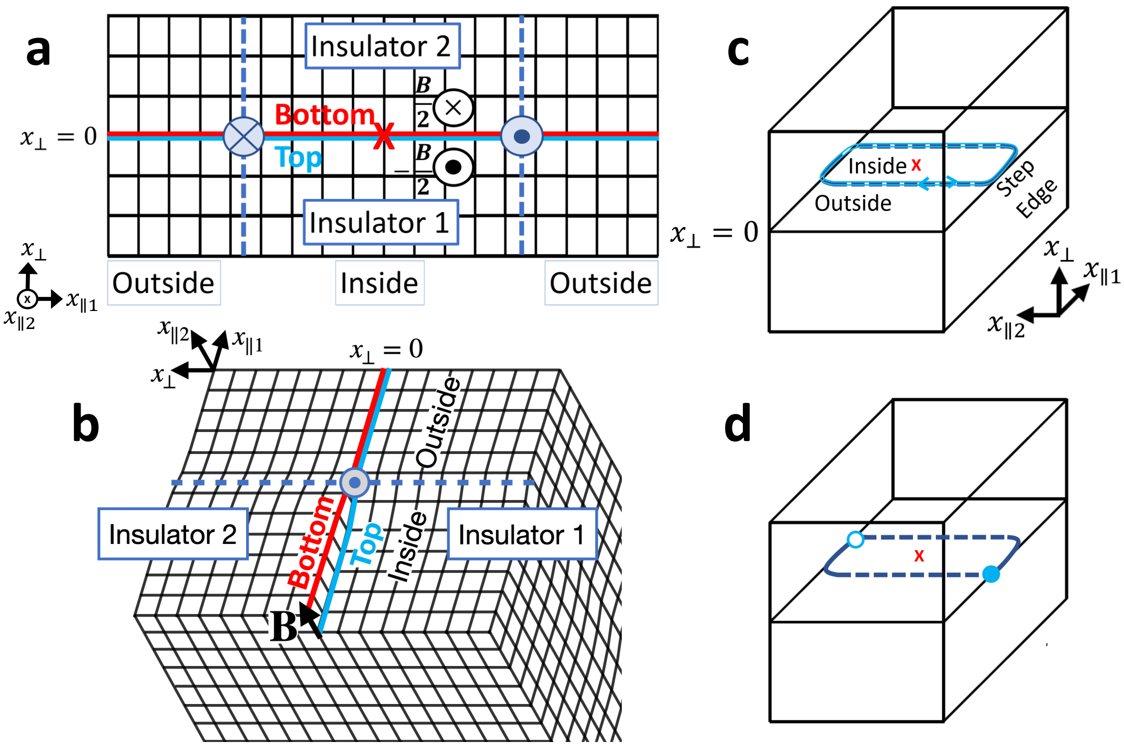

Supplementary Figure 3. **0D screw dislocation states in a 3D crystal.** ***a*** A cross-sectional cut of an $\mathcal{I}$- and $\mathcal{T}$-symmetric 3D crystal with a pair of screw dislocations with opposite sense vectors $\boldsymbol{s}$ [127], indicated by the blue vectors into and out of the page on the left and right, respectively. The Burgers vectors $\boldsymbol{B}$ of both dislocations point into the page, which we take to be the positive $x_{\parallel 2}$ direction. Therefore, as indicated by sgn$[\boldsymbol{B}\cdot\boldsymbol{s}]$ [SEq. (42)], the screw on the left (right) in ***a*** is right-(left-) handed. Following the procedure in SRef. 71 and SN 2 A 1, we divide the system in ***a*** by cutting along the red, blue, and dashed lines. As previously with the edge dislocation in SFig. 2 ***a***, the red and blue lines separate two insulators with the same bulk topology. However, unlike for an edge dislocation, we instead glue the top and bottom surfaces of the two insulators together with (inside) and without (outside) an extra lattice displacement in the $x_{\parallel 2}$ direction. To preserve an inversion center (red $\times$), we split the displacement evenly over insulators 1 and 2, such that insulator 1 (2) is displaced by $|\boldsymbol{B}/2|$ in the $-x_{\parallel 2}$ $(+x_{\parallel 2})$ direction. ***b*** A 3D view of the $\hat{x}_{\parallel 2}$-normal surface of the crystal with screw dislocations in panel ***a***. ***c*** A 3D crystal with the pair of screw dislocations from ***a*** (dashed lines). Unlike previously in SFig. 2 ***b, c***, we are unable to consider a fully internal screw dislocation (*i.e.*, a screw dislocation that does not terminate on a surface), because, as the Burgers vectors of screw dislocations are parallel to the dislocation line (sense vector) [127], such a dislocation would have a position-dependent Burgers vector. Because we expect the electronic structure of screw dislocations with spatially varying Burgers vectors to be considerably more complicated than that of screw dislocations with spatially constant Burgers vectors, we do not further examine screw dislocations with spatially-varying Burgers vectors in this work. Instead, we simply consider two parallel screw dislocations with the same Burgers vector $\boldsymbol{B}$, and avoid the curvature effects of sharp corners [70] by lightly "sanding" away some of the crystal near the screw dislocations so that the dislocations curve (relatively) smoothly into surface step edges (solid blue lines). In this geometry (panel ***c***), the screw dislocations and step edges form a closed loop that is topologically equivalent to the rounded internal edge dislocation in SFig. 2 ***b***. If the Burgers vectors of the screw dislocations and the bulk electronic structure satisfy SEq. (55), then the screw and step-edge loop in ***c*** will bind an odd (anomalous) number of helical modes, as previously shown in SRefs. 71, 72, 75–77, and 80. ***d*** If an inversion center is also preserved throughout the formation of the screw dislocations, then, upon the introduction of $\mathcal{I}$-symmetric magnetism, $\boldsymbol{M}_\nu$ will again be converted to the weak fragile (and OAL) index $\boldsymbol{M}_\nu^{\mathrm{F}}$ [see SEq. (56) and SN 3 B], and the helical modes will gap into an anomalous number of HEND states. In ***d***, we depict the two filling-anomalous HEND states appearing on alternating, $\mathcal{I}$-related ends of the screw dislocations in ***a***, as they do in our numerical calculations in SN 4. The four ends of the two screw dislocations in ***d*** are thus equivalent to the corners of the $\mathcal{I}$-symmetric 2D FTI characterized in SRefs. 33 and 100.

where y^T_μ is the displacement of insulator 1 in the region μ, and $\mathcal{Z}^T(z)$ and $|+\rangle_i$ are defined in SEq. (8) and the surrounding text. We next derive the dispersion (velocity) terms for the top-surface Hamiltonian by projecting the $q_{x,y}$ dispersion terms from SEq. (2) into the basis of $|\psi^T_{a,\mu}(z)\rangle_i$, as was previously done in SEq. (10):

$$\mathcal{H}^T_{a,\mu}(q_x,q_y) = v_x q_x s^y + v_y q_y s^x, \tag{44}$$

where s^i is a 2×2 matrix that indexes the states $|+\rangle_i$. SEq. (44) is invariant under $\mathcal{T}$ symmetry, which remains represented by SEq. (11). Next, following SEq. (13), we obtain the bottom-surface bound states of insulator 2:

$$|\psi^B_{a,\mu}(z)\rangle_i = e^{i(\boldsymbol{k}_{D,a}\cdot\hat{y})y^B_\mu} e^{i(\boldsymbol{k}_{D,a}\cdot\hat{z})z}\mathcal{Z}^B(z)|-\rangle_i. \tag{45}$$

We then project the bulk $q_{x,y}$ dispersion terms of SEq. (2) into the basis of $|\psi^B_{a,\mu}(z)\rangle_i$ to realize the bottom-surface Hamiltonian:

$$\mathcal{H}^B_{a,\mu}(q_x,q_y) = -v_x q_x s^y - v_y q_y s^x = -\mathcal{H}^T_{a,\mu}(q_x,q_y), \tag{46}$$

where $s^{x,y,z}$ are Pauli matrices that act on the states $|-\rangle_i$, $i=1,2$. SEq. (46) is invariant under $\mathcal{T}$ symmetry, which is also represented by SEq. (11).

With SEqs. (44) and (46) established, we can again form the Hamiltonian in the plane between the defects ($x_\perp = 0$ in SFig. 3). We begin by forming the uncoupled Hamiltonian of both the top and bottom surfaces, which is, as previously in the case of edge dislocations [see the text surrounding SEq. (16)], the Hamiltonian of a fourfold-degenerate, 2D Dirac fermion [25, 226–228]:

$$\mathcal{H}^D_{a,\mu}(q_x,q_y) = v_x q_x \xi^z s^y + v_y q_y \xi^z s^x. \tag{47}$$

In SEq. (47), ξ^i is a Pauli matrix that indexes the "top" and "bottom" degrees of freedom and s continues to index $i=1,2$ in $|\pm\rangle_i$ within each of the two surfaces of the insulator described by SEq. (1). In this geometry, $\mathcal{I}$ and $\mathcal{T}$ are respectively represented through the actions in SEqs. (17) and (18).

Finally, we allow for $\mathcal{I}$- and $\mathcal{T}$-symmetric coupling between the top and bottom surfaces of the two insulators. Once again, SEqs. (17), (18), and (47) imply that there will only be a single coupling mass, and that it will be proportional to ξ^x. We account for the presence of the screw dislocations by defining a consistent coupling phase about $y=z=0$ (red $\times$ in SFig. 3 ***a***) in the spinless coupling interaction:

$$[V^{\mathrm{C}}_{a,\mu}]^{TB}_{ij} = m_{\mathrm{C}}\delta_{ij}\int_{-\infty}^{\infty} dz\ \left[|\psi^T_{a,\mu}(z)\rangle_i\langle\psi^B_{a,\mu}(z)|_j + \text{h.c.}\right],$$
$$[V^{\mathrm{C}}_{a,\mu}]^{TT}_{ij} = [V^{\mathrm{C}}_{a,\mu}]^{BB}_{ij} = 0, \tag{48}$$

where m_{C} is the strength of the coupling; $i,j=1,2$; and δ_{ij} enforces $\mathcal{T}$ symmetry [SEq. (18)] by again excluding spinful (magnetic) interactions. Substituting SEqs. (43) and (45) into SEq. (48), we arrive at the expression:

$$\begin{aligned}[V^{\mathrm{C}}_{a,\mu}]^{TB}_{ij} &= m_{\mathrm{C}}\delta_{ij}\int_{-\infty}^{\infty} dz\ \left[e^{i(\boldsymbol{k}_{D,a}\cdot\hat{y})(y^T_\mu - y^B_\mu)}\mathcal{Z}^T(z)\mathcal{Z}^B(z)|+\rangle_{ij}\langle -| + \text{h.c.}\right],\\ &= m_{\mathrm{C}}\delta_{ij}e^{i(\boldsymbol{k}_{D,a}\cdot\hat{y})(y^T_\mu - y^B_\mu)}|+\rangle_{ij}\langle -| + \text{h.c.},\end{aligned} \tag{49}$$

where, in the second line, we have again employed the approximation that $\mathcal{Z}^T(z)\mathcal{Z}^B(z)\to\delta(z)$ [see the text preceding SEq. (23)].

In the $\mu = out$ region:

$$y^T_{out} = y^B_{out} = 0, \tag{50}$$

such that:

$$V^{\mathrm{C}}_{a,out} = m_{\mathrm{C}}\xi^x. \tag{51}$$

For the $\mu = in$ region, however, for which:

$$y^T_{in} = -|\boldsymbol{B}|/2,\ y^B_{in} = |\boldsymbol{B}|/2, \tag{52}$$

an additional nonzero phase factor persists from SEq. (49):

$$[V^{\mathrm{C}}_{a,in}]^{TB}_{ij} = m_{\mathrm{C}}\delta_{ij}e^{-i(\boldsymbol{k}_{D,a}\cdot\hat{y})|\boldsymbol{B}|}|+\rangle_{ij}\langle -| + \text{h.c.}, \tag{53}$$

just as it previously did for the edge dislocation in SEq. (24). As discussed in the text surrounding SEq. (25), we next restrict consideration to integer Burgers vectors, and require $\mathcal{I}$ and $\mathcal{T}$ symmetry, which allows us to discard the imaginary phases in SEq. (53). Imposing this restriction on SEq. (53) leads to the simplified expression:

$$V^{\mathrm{C}}_{a,in} = m_{\mathrm{C}} \cos\left(\boldsymbol{k}_{D,a} \cdot \boldsymbol{B}\right) \xi^{x}, \tag{54}$$

where we have used the previous simplification that $\boldsymbol{B} = |\boldsymbol{B}|\hat{y}$ to reexpress the argument of the cosine in a more general form that is independent of our choice of the screw-dislocation Burgers vector direction. For each band-inverted bulk TRIM point $\boldsymbol{k}_{D,a}$, the screw dislocations will therefore form domain walls in the sign of V^{C}_{a} if and only if $\cos\left(\boldsymbol{k}_{D,a} \cdot \boldsymbol{B}\right) = -1$, which is only satisfied by the previous conditions in SEq. (34). Because SEq. (47) is equivalent to the Hamiltonian of a 2D, $\mathcal{T}$-symmetric, fourfold Dirac fermion [25, 226–228], the domain walls necessarily bind an anomalous pair of helical modes (SFig. 3 ***c***), which are equivalent to the edge states of a 2D TI [3, 5, 6] located in the plane between the two dislocations (the "inside" region in SFig. 3). The total number of helical modes is thus determined by summing over all of the bulk TRIM points [SEq. (33)], again resulting in the statement that a closed loop formed from two screw dislocations and two step edges in an $\mathcal{I}$- and $\mathcal{T}$-symmetric crystal will bind anomalous helical modes if the dislocation Burgers vector $\boldsymbol{B}$ and the weak index vector $\boldsymbol{M}_{\nu}$ of the pristine crystal satisfy:

$$\boldsymbol{B} \cdot \boldsymbol{M}_{\nu} \text{ mod } 2\pi = \pi, \tag{55}$$

reproducing the central result of SRefs. 71, 72, 75–77, and 80.

As previously in SN 2 A 1, we can immediately extend SEq. (55) to the $\mathcal{T}$-broken regime through the recent recognition that the edge states of an $\mathcal{I}$-symmetric 2D TI necessarily gap into the anomalous corner modes of a 2D FTI under the introduction of $\mathcal{I}$-symmetric magnetism [33, 100]. Unlike previously for edge dislocations, we are unable to form a fully internal loop of screw dislocations (*i.e.*, screw dislocations that do not terminate on a surface), because the Burgers vector of a pair of screw dislocations is parallel to the dislocation, and we wish to avoid the confounding complications of position-dependent Burgers vectors. However, we can consider the closed loop of two screw dislocations and two step edges in SFig. 3 to be sufficiently smooth for the previous analysis in SEqs. (36) through (39) to remain applicable if the ends of the screw dislocations are taken to continuously evolve into the surface step edges in SFig. 3 ***b, c, d*** in an $\mathcal{I}$-symmetric manner with nonsingular local curvature [70]. In this case, the closed loop of the dislocations and step edges becomes equivalent to the smooth boundary of an $\mathcal{I}$-symmetric FTI [33, 100], and binds an anomalous number of 0D HEND states if:

$$\boldsymbol{B} \cdot \boldsymbol{M}^{\mathrm{F}}_{\nu} \text{ mod } 2\pi = \pi, \tag{56}$$

where $\boldsymbol{M}^{\mathrm{F}}_{\nu}$ is the new weak index previously introduced in SEq. (40) to characterize which of the BZ-boundary planes of the pristine bulk insulator host Hamiltonians equivalent to (possibly trivialized copies of) 2D $\mathcal{I}$-symmetric FTIs. In SN 3 B 1 and 3 B 2, we will rigorously define $\boldsymbol{M}^{\mathrm{F}}_{\nu}$ and its $\mathcal{T}$-symmetric generalization using EBRs and nested Wilson loops.

3. $k \cdot p$ Derivation of HEND States on Flux Tube Ends in AXIs and HOTIs

In this section, we will exploit the previous derivation in SN 2 A 2 of HEND states bound to screw dislocations to determine the flux response of $\mathcal{I}$-symmetric axion insulators (AXIs). As in the previous two sections (SN 2 A 1 and 2 A 2), we begin by considering a 3D insulator with $\mathcal{I}$ and $\mathcal{T}$ symmetries that differs from an atomic insulator by a series of band inversions at the Fermi energy between only the two highest valence bands and the two lowest conduction bands. We take these band inversions to occur at a set of TRIM points $\boldsymbol{k}_{D,a}$ between Kramers pairs of states with opposite parity eigenvalues. The Hamiltonian of the insulator in the absence of an external magnetic field is given by SEqs. (1) and (2). Next, we thread magnetic flux into the insulator through two small parallel tubes with opposite field strengths $\pm\phi$ located at $\mathcal{I}$-related positions (SFig. 4 ***a***). We take the magnetic field in the right (left) tube to be directed in the $\hat{y}$ $(-\hat{y})$ direction, such that:

$$x_{\perp} = z,\ x_{\parallel 1} = x,\ x_{\parallel 2} = y, \tag{57}$$

in the notation of SFig. 4. The tubes are therefore localized in the $z = 0$ plane at $\mathcal{I}$-related x coordinates. We note, however, that this flux configuration is not invariant under $\mathcal{I}$ and $\mathcal{T}$ symmetries for generic values of ϕ, because $\mathcal{T}\phi = -\phi$, and because the action of $\mathcal{I}$ exchanges the locations of the flux tubes, which generically carry opposite magnetic fluxes $\pm\phi$. Later in this section, we will specialize to the cases of $\phi \text{ mod } 2\pi = 0, \pi$, at which $\mathcal{I}$ and $\mathcal{T}$ symmetries are restored.

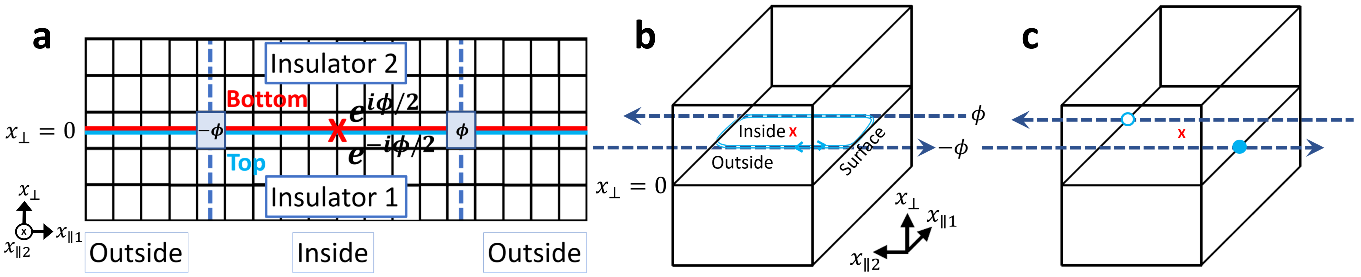

Supplementary Figure 4. **0D π-flux states in a 3D crystal.** ***a*** A cross-sectional cut of an $\mathcal{I}$- and $\mathcal{T}$-symmetric 3D crystal with a pair of threaded flux tubes with opposite magnetic flux $\pm\phi$ directed in the $x_{\parallel 2}$ direction, indicated by the blue plaquettes. Following the procedure used for screw dislocations in SN 2 A 2, we divide the system in ***a*** by cutting the lattice along the red, blue, and dashed lines. As previously with the screw dislocations in SFig. 3 ***a***, the red and blue lines separate two insulators with the same bulk topology. To implement the effects of the $\pm\phi$-flux tubes, we multiply all couplings between the top surface states of insulator 1 and the bottom surface states of insulator 2 by $e^{i\phi}$ in the "inside" region between the two flux tubes. The tubes are inserted at locations related by a bulk inversion center (red $\times$), and importantly, $\mathcal{I}$ is only a system symmetry when ϕ mod $2\pi = 0, \pi$. ***b*** An $\mathcal{I}$-symmetric strong TI with the pair of flux tubes from ***a*** (dashed lines). When $\phi = \pi$ per unit cell (depicted as plaquettes in panel ***a***), each tube binds an anomalous pair of helical modes, forming a "wormhole" between the $\pm\hat{x}_{\parallel 2}$-normal surfaces [164–170]. Though the wormhole states merge with the surface states, we can consider the closed loop of helical modes across the flux tubes and parallel surfaces to be equivalent to the anomalous edge of a 2D TI. ***c*** Relaxing $\mathcal{T}$ symmetry while preserving $\mathcal{I}$, the bulk of both insulators 1 and 2 necessarily transitions into an $\mathcal{I}$-symmetric axion insulator (AXI) [7, 8, 10, 13, 25, 33, 96, 106, 219–222]. Relying on the previous recognition from SRefs. 33 and 100 that the edge states of an $\mathcal{I}$-symmetric 2D TI gap into the anomalous corner modes of a 2D FTI in the presence of $\mathcal{I}$-symmetric magnetism, we conclude that π-flux tubes threaded through an AXI bind 0D HEND states that, like the HEND states bound to the edge and screw dislocations previously discussed in SN 2 A 1 and 2 A 2 respectively, are equivalent to the $\pm e/2$-charged corner modes of an $\mathcal{I}$-symmetric 2D FTI. In ***c***, we depict two filling-anomalous HEND flux states appearing on alternating, $\mathcal{I}$-related ends of the flux tubes in ***a***, as they do in our numerical calculations in SN 5.

To implement the pair of flux tubes, we cut the insulator described by $\mathcal{H}(\boldsymbol{q})$ [SEq. (1)] into two pieces with $\pm\hat{z}$-normal surfaces, and again glue the pieces back together. Instead of gluing together the region between the flux tubes (labeled "inside" in SFig. 4) with a relative translation between the top and bottom surfaces of the two insulators, we instead rotate the phase of the top (bottom) surface states of the $\mu = in$ region of insulator 1 (2) by $-\phi/2$ ($\phi/2$). Formally, this is accomplished through the substitution:

$$(\boldsymbol{k}_{D,a}\cdot\hat{y})y_\mu^{T,B} \rightarrow \phi_\mu^{T,B}, \quad \phi_\mu^{T,B} = \mp\frac{\phi}{2}, \tag{58}$$

in SEqs. (43) and (45), respectively, resulting in:

$$|\psi^T_{a,\mu}(z)\rangle_i = e^{i\phi^T_\mu} e^{i(\boldsymbol{k}_{D,a}\cdot\hat{z})z}\mathcal{Z}^T(z)|+\rangle_i,$$
$$|\psi^B_{a,\mu}(z)\rangle_i = e^{i\phi^B_\mu} e^{i(\boldsymbol{k}_{D,a}\cdot\hat{z})z}\mathcal{Z}^B(z)|-\rangle_i. \tag{59}$$

Projecting the $q_{x,y}$ dispersion terms in SEq. (2) into the basis of the top and bottom surface states in SEq. (59), and, for now, ignoring coupling between the top and bottom surfaces, we again realize the Hamiltonian of a 2D fourfold Dirac fermion [25, 226–228]:

$$\mathcal{H}^D_{a,\mu}(q_x, q_y) = v_x q_x \xi^z s^y + v_y q_y \xi^z s^x, \tag{60}$$

as we did previously in the cases of edge and screw dislocations [SEqs. (16) and (47), respectively]. In SEq. (60), ξ is a matrix that acts on the "top" and "bottom" degrees of freedom, and s continues to act on the indices $i = 1, 2$ in $|\pm\rangle_i$ within each of the two surfaces of the insulator described by SEq. (1). Because SEq. (60) is independent of ϕ, then SEq. (60) remains invariant under $\mathcal{I}$ and $\mathcal{T}$ for all values of ϕ.

However and importantly, because magnetic flux is odd under $\mathcal{T}$, and the locations of the flux tubes in SFig. 4 are exchanged by the action of $\mathcal{I}$ (red $\times$), then the full Hamiltonian of the "glued" interface at $z = 0$ ($x_\perp = 0$), which includes symmetry-allowed couplings, will *not* be invariant under $\mathcal{I}$ and $\mathcal{T}$ symmetries for generic values of ϕ, but will rather only be invariant under the product $\mathcal{I}\times\mathcal{T}$. We capture $\mathcal{I}$- and $\mathcal{T}$-symmetry-breaking at generic flux strengths by introducing a coupling interaction $V^{\mathrm{C}}_{a,\mu}(\phi)$ between the top and bottom surfaces of the two insulators in SFig. 4:

$$\mathcal{H}^C_{a,\mu}(q_x, q_y, \phi) = \mathcal{H}^D_{a,\mu}(q_x, q_y) + V^{\mathrm{C}}_{a,\mu}(\phi), \tag{61}$$

where $\mathcal{H}^C_{a,\mu}(q_x,q_y,\phi)$ is now a three-parameter *family* of Hamiltonians indexed by $q_{x,y}$ and ϕ. In the specific flux-tube geometry in SFig. 4:

$$\mathcal{I}\phi = -\phi,\ \mathcal{T}\phi = -\phi, \tag{62}$$

such that the action of $\mathcal{I}$ symmetry from SEq. (17) becomes:

$$\mathcal{I}:\ \mathcal{H}^C_{a,\mu}(q_x,q_y,\phi) \rightarrow \xi^x \mathcal{H}^C_{a,\mu}(-q_x,-q_y,-\phi)\xi^x, \tag{63}$$

and the action of $\mathcal{T}$ symmetry from SEq. (18) becomes:

$$\mathcal{T}:\ \mathcal{H}^C_{a,\mu}(q_x,q_y,\phi) \rightarrow s^y[\mathcal{H}^C_{a,\mu}(-q_x,-q_y,-\phi)]^* s^y. \tag{64}$$

SEqs. (62), (63), and (64), along with the recognition that ϕ is only implemented as the argument of the exponential $e^{i\phi}$ [see the text surrounding SEq. (59)], imply that the system can only be $\mathcal{I}$ and $\mathcal{T}$ invariant at $\phi \bmod 2\pi = 0,\pi$.

From SEq. (60), we recognize that $V^{\mathrm{C}}_{a,\mu}(\phi)$ can only be guaranteed to open a gap if it contains terms proportional to ξ^x, ξ^y, $\xi^z s^z$, or s^z. Furthermore, of these possibilities, only ξ^x is invariant under $\mathcal{I}$ and $\mathcal{T}$ symmetries as represented in SEqs. (63) and (64), respectively. Hence, as in this work we are considering the restrictions on flux bound states from $\mathcal{I}$ and $\mathcal{T}$ symmetries, then we may focus without loss of generality on spinless coupling terms containing ξ^x, which are captured by:

$$\begin{aligned}
[V^{\mathrm{C}}_{a,\mu}(\phi)]^{TB}_{ij} &= m_{\mathrm{C}}\delta_{ij}\int_{-\infty}^{\infty} dz\ \left[|\psi^T_{a,\mu}(z)\rangle_i\langle\psi^B_{a,\mu}(z)|_j + \mathrm{h.c.}\right], \\
[V^{\mathrm{C}}_{a,\mu}(\phi)]^{TT}_{ij} &= [V^{\mathrm{C}}_{a,\mu}(\phi)]^{BB}_{ij} = 0,
\end{aligned} \tag{65}$$

where m_{C} is the strength of the coupling. Substituting SEq. (59) into SEq. (65), we arrive at the expression:

$$\begin{aligned}
[V^{\mathrm{C}}_{a,\mu}(\phi)]^{TB}_{ij} &= m_{\mathrm{C}}\delta_{ij}\int_{-\infty}^{\infty} dz\ \left[e^{i(\phi^T_\mu-\phi^B_\mu)}\mathcal{Z}^T(z)\mathcal{Z}^B(z)|+\rangle_{ij}\langle -| + \mathrm{h.c.}\right], \\
&= m_{\mathrm{C}}\delta_{ij}e^{i(\phi^T_\mu-\phi^B_\mu)}|+\rangle_{ij}\langle -| + \mathrm{h.c.}.
\end{aligned} \tag{66}$$

where, in the second line, we have again employed the approximation that $\mathcal{Z}^T(z)\mathcal{Z}^B(z)\rightarrow\delta(z)$ described in the text before SEq. (23)

In the $\mu = out$ region:

$$\phi^T_{out} = \phi^B_{out} = 0, \tag{67}$$

such that:

$$V^{\mathrm{C}}_{a,out}(\phi) = m_{\mathrm{C}}\xi^x. \tag{68}$$

For the $\mu = in$ region, however, for which:

$$\phi^T_{in} = -\phi/2,\ \phi^B_{in} = \phi/2, \tag{69}$$

an additional nonzero phase factor persists from SEq. (66):

$$\begin{aligned}
[V^{\mathrm{C}}_{a,in}(\phi)]^{TB}_{ij} &= m_{\mathrm{C}}\delta_{ij}e^{-i\phi}|+\rangle_{ij}\langle -| + \mathrm{h.c.} \\
V^{\mathrm{C}}_{a,in}(\phi) &= m_{\mathrm{C}}\left[\cos(\phi)\xi^x + \sin(\phi)\xi^y\right].
\end{aligned} \tag{70}$$

While the family of coupling terms characterized by SEq. (70) is as a set invariant under $\mathcal{I}$ and $\mathcal{T}$ symmetries as represented by SEqs. (63) and (64), ϕ as a parameter is itself only invariant under $\mathcal{I}$ and $\mathcal{T}$ symmetries at $\phi \bmod 2\pi = 0,\pi$ [see the text surrounding SEq. (62)]. Hence, at generic values of ϕ away from 0 and π, SEq. (70) is only invariant under the magnetic symmetry $\mathcal{I}\times\mathcal{T}$, as expected for the flux tube geometry shown in SFig. 4 ***a***.

If we restrict consideration to flux tubes with $\phi=\pi$, then SEq. (70) simplifies to an $\mathcal{I}$- and $\mathcal{T}$-symmetric form:

$$V^{\mathrm{C}}_{a,out}(\pi) = -m_{\mathrm{C}}\xi^x. \tag{71}$$

Thus, the π-flux tubes represent domain walls in the mass of a $\mathcal{T}$-symmetric 2D fourfold Dirac fermion [25, 226–228] for *every* bulk band-inverted TRIM point $\boldsymbol{k}_{D,a}$, *distinctly unlike* the previous k-dependent masses for the edge and

screw dislocations in SN 2 A 1 and 2 A 2, respectively [SEqs. (25) and (54), respectively]. Hence, each $\boldsymbol{k}_{D,a}$ contributes an anomalous pair of helical modes to each flux tube (SFig. 4 ***b***). As the full coupled Hamiltonian is given by:

$$\mathcal{H}^{C}_{\mu}(q_x, q_y, \phi) = \bigoplus_{a} \mathcal{H}^{C}_{a,\mu}(q_x, q_y, \phi), \tag{72}$$

then the total number of pairs of helical modes is simply equal to the number of bulk band-inverted TRIM points, and specifically, is only anomalous if this number is odd. Therefore, π-flux tubes will only bind anomalous helical modes (*i.e.* "wormholes" between surfaces [167, 168]) in a 3D $\mathcal{I}$- and $\mathcal{T}$-symmetric insulator that differs from an atomic insulator by an odd number of band inversions between bands with opposite parity eigenvalues. Through the Fu-Kane parity criterion [7, 8], we recognize that such an insulator is necessarily a strong 3D TI. We have thus reproduced the result of SRefs. 164–170 that π-flux tubes in 3D strong TIs bind anomalous helical modes.

More generally, we can rearrange the sum in SEq. (72) to gain further insight:

$$\mathcal{H}^{C}_{\mu}(q_x, q_y, \phi) = \bigoplus_{k_z} \bigoplus_{\boldsymbol{k}_{D,a}\cdot\hat{z}=k_z} \mathcal{H}^{C}_{a,\mu}(q_x, q_y, \phi) \equiv \bigoplus_{k_z=0,\pi} \mathcal{H}^{C}_{k_z,\mu}(q_x, q_y, \phi), \tag{73}$$

where we have separated the low-energy contributions of the TRIM points by k_z. The sum in SEq. (73) implies that π-flux tubes bind anomalous helical modes in a 3D $\mathcal{I}$- and $\mathcal{T}$-symmetric insulator that contains an odd number of k_z-indexed BZ planes with Hamiltonians that differ from 2D atomic insulators by an odd number of band inversions that exchange parity eigenvalues (*i.e.* are topologically equivalent to 2D TIs [3, 5, 6]). Therefore, we can consider the π-flux tubes to be summing the 2D topologies of *all* of the Hamiltonians of the k_z-indexed planes of the pristine crystal [SEq. (1)], and projecting them to the real-space surface spanning the flux tubes (labeled "inside" in SFig. 4 ***a, b***). As previously with the screw dislocations in SFig. 3 ***c, d***, we can imagine lightly bending the flux tubes as they approach the crystal surface to avoid any curvature effects associated with sharp corners [70]. In this construction, and for now ignoring the complications of merging surface and flux-tube states, an $\mathcal{I}$- and $\mathcal{T}$-symmetric crystal with two flux tubes will therefore host a real-space surface between the tubes that is equivalent to a 2D TI if and only if the bulk contains an odd number of (here k_z-indexed) 2D surfaces with the topology of a 2D TI. Specifically, because the superposition of two 2D TIs does not exhibit anomalous helical modes (though it does exhibit anomalous corner states if $\mathcal{I}$ symmetry is additionally present [70, 100]), then helical modes only appear on the tubes if an odd number of 2D TIs contribute to the summation in SEq. (73).

Furthermore, because a 3D TI can be reexpressed as a pumping cycle of a 2D TI with odd, helical winding [7, 8, 13, 244], the edges of the real-space surface between the π-flux tubes (*i.e.*, the tubes themselves, as well as two lines on the crystal surface [SFig. 4 ***b, c***] bind anomalous helical modes if and only if the bulk pristine crystal is a 3D strong TI. Crucially, while the location of the 2D real-space surface is electromagnetic gauge-dependent (here, we take it to simply lie at $z = 0$), the locations of its edges, the π-flux tubes, are gauge-independent because we have fixed their positions in real space, and because they give rise to a gauge-invariant Aharonov-Bohm phase shift. This pumping interpretation of π-flux response is explored for more general Hamiltonians in SN 2 B 2.

From this result, it becomes straightforward to derive the π-flux response of $\mathcal{I}$-symmetric AXIs. Numerous previous works [7, 8, 10, 13, 25, 33, 96, 106, 219–222] have shown that an $\mathcal{I}$-symmetric 3D strong TI gaps into an AXI under the introduction of $\mathcal{I}$-symmetric magnetism. Furthermore, it was shown in recent works [33, 100, 107–110] that, because an $\mathcal{I}$-symmetric 2D TI gaps into an FTI with anomalous corner modes under breaking $\mathcal{T}$ symmetry with $\mathcal{I}$-symmetric magnetism, then an AXI is equivalent to an odd, chiral pumping cycle of an $\mathcal{I}$-symmetric FTI. For example, in the AXI models in SRef. 33, the Hamiltonian in the $k_z = 0$ BZ plane is equivalent to a $\mathcal{I}$-symmetric 2D FTI with (filling-) anomalous corner modes, whereas the Hamiltonian in the $k_z = \pi$ plane is equivalent to 2D trivial atomic insulator. Thus, when $\mathcal{T}$ symmetry is relaxed in an $\mathcal{I}$-symmetric 3D TI with two π-flux tubes, the helical surface and flux states gap, resulting in an anomalous number of fractionally charged 0D states bound to the loop formed from the two flux tubes and the crystal surfaces (SFig. 4 ***c***). Therefore, π-flux tubes in an AXI necessarily bind anomalous HEND states, which appear in our numerical calculations on $\mathcal{I}$-related flux tube ends (SN 5).

This can also be understood through the pumping interpretation of the flux response described in the text surrounding SEq. (73). Because y-directed π-flux tubes separated by an x-directed displacement project the superposed topologies of the momentum-space Hamiltonians of k_z-indexed planes of a pristine AXI, and, because there are necessarily an odd number of k_z-indexed BZ planes with corner modes in an AXI pumping cycle [33], then the π-flux tubes necessarily bind HEND states. Furthermore, because the flux-tube and surface loop (SFig. 4 ***c***) exhibits the largest curvature [70] where the tubes meet the surfaces, then at least two HEND states will necessarily be localized on $\mathcal{I}$-related tube ends on opposing surfaces. This is in agreement with the results of previous works [8, 13, 75, 167, 168] that showed that the surfaces of an AXI accumulate fractional charge under threaded π flux in a manifestation of the nontrivial bulk magnetoelectric polarizability [10, 13, 33, 106, 212, 219, 252–254]. Furthermore, because π fluxes in $|C| = 1$ Chern insulators bind 0D states with $\pm e/2$ fractional charge (SRef. 75 and SN 5), then the presence of

isolated HEND states on $\mathcal{I}$-related surfaces represents a signature that AXI surfaces are topologically equivalent to anomalous "halves" of the integer quantum Hall effect [7, 8, 10, 13, 25, 33, 96, 106, 219–222]. Specifically, each surface of an AXI exhibits only *half* of the π-flux response of a $|C| = 1$ Chern insulator, because only one of the two flux cores per surface binds a HEND state (with the $\mathcal{I}$-related HEND state being localized on the opposite surface).

Exploiting the recent recognition that $\mathcal{I}$- and $\mathcal{T}$-symmetric higher-order TIs (HOTIs) are equivalent to two superposed, $\mathcal{T}$-reversed copies of AXIs [33, 100], we can use the above conclusions to determine the π-flux response of HOTIs. Because an $\mathcal{I}$- and $\mathcal{T}$-symmetric FTI exhibits corner modes that are equivalent to the spin-charge separated solitons of the spinful Su-Schrieffer-Heeger (SSH) chain [33, 100, 215, 217, 218], and are themselves formed from Kramers pairs of the corner (anti)solitons of $\mathcal{T}$-broken FTIs [33, 100], then we immediately conclude that π-flux tubes in HOTIs bind Kramers pairs of anomalous spin-charge separated HEND states (*i.e.* fluxons [164–166, 169, 170]). Like the π-flux HEND states in AXIs, the 0D π-flux states in HOTIs appear on alternating, $\mathcal{I}$-related ends of pairs of tubes (SFig. 4 ***c***). Crucially, because π fluxes in 2D TIs bind 0D fluxons (SRefs. 76, 164–166, 169, and 170 and SN 5), then the presence of unpaired HEND states on $\mathcal{I}$-related surfaces represents a signature that gapped HOTI surfaces are not topologically equivalent to 2D trivial or topological insulators, but are rather equivalent to anomalous "halves" of the quantum *spin* Hall effect, a relatively unexplored phase of matter. Specifically, each surface of a HOTI only exhibits half the π-flux response of a 2D TI, because the two flux cores on each surface only bind one total fluxon (which manifests in our numerical calculations as a Kramers pair of HEND states on the end of only one of the two flux tubes). While the half quantum spin Hall (QSH) effect was previously predicted to occur on the top surfaces of weak TIs [177], in this work, we recognize the half QSH effect to more generally manifest on *all* (gapped) surfaces of $\mathcal{I}$- and $\mathcal{T}$-symmetric HOTIs.

To understand why the surfaces of $\mathcal{I}$- and $\mathcal{T}$-symmetric HOTIs host half-integer QSH phases, instead of full-integer 2D trivial or topological insulators, we draw connection to recent discussions of symmetry-enhanced fermion doubling in TCIs. Specifically, the 2D surfaces of 3D HOTIs are generically formed from fourfold, $\mathcal{T}$-symmetric Dirac cones [100] that become gapped by symmetry-allowed perturbations in the absence of specific surface symmetries, such as perpendicular glide reflections [25]. When located at a surface TRIM point, each fourfold Dirac cone allows a single $\mathcal{T}$-symmetric anticommuting mass, whose sign has been shown in previous works [31–33, 103–105] to be an intrinsic property of the bulk topology. As we will detail below, if one were to try to describe the topology of any individual gapped 2D HOTI surface, one would be unable to regularize the continuum theory derived from the single gapped fourfold Dirac cone, due to an obstruction from symmetry-enhanced fermion doubling [25]; the theory could only be regularized if another (gapped) surface fourfold Dirac cone were also present [226]. This is analogous to the gapped – yet not regularizable – surface theory of the massive twofold Dirac cone appearing on AXI surfaces, which exhibits an anomalous $\sigma_{xy} = e^2/(2h)$ Hall conductivity. Indeed, $\mathcal{I}$- and $\mathcal{T}$-symmetric HOTIs have been shown to admit an s^z-preserving limit [100], and in this limit, it is clear that the obstruction to lattice-regularizing the surface theory originates from the parity anomaly (anomalous half-integer surface Hall conductivity) per spin sector.

More generally, in the presence of spin-orbit coupling (SOC) that removes the s^z sector labels, the obstruction to regularizing the gapped surface theory of $\mathcal{I}$- and $\mathcal{T}$-symmetric HOTIs can be understood by considering the role of crystal symmetries. First, it has been shown that an $\mathcal{I}$- and $\mathcal{T}$-symmetric HOTI can be expressed as the real-space superposition (formally a "stack" or Kronecker sum) of $2+4n$ ($n \in \{\mathbb{Z}^+, 0\}$) $\mathcal{I}$-symmetric 3D TIs [103, 105, 173]. We emphasize that the term superposition in real space does *not* imply that the HOTI ground state wavefunction is a superposition of the ground state wavefunctions of $2+4n$ 3D TIs – rather, in the decoupled limit, the HOTI ground state wavefunction is a product of the individual ground state wavefunctions of the $2+4n$ superposed (summed) 3D TIs. Next, considering the case of two superposed, initially decoupled 3D TIs with $\mathcal{I}$ symmetry that host band inversions at the same bulk TRIM point, each surface carries an unpaired, gapless fourfold Dirac cone that is stabilized by $\mathcal{T}$ symmetry and an artificial ("sublattice") symmetry that indexes the two decoupled, superposed TIs [25, 31, 100, 102, 103, 105]. When $\mathcal{I}$- and $\mathcal{T}$-symmetric coupling between the two superposed TIs is introduced to break the artificial symmetry, the fourfold surface Dirac cones develop a gap. Crucially, SRefs. 103, 105, and 173 demonstrated that the sign of the $\mathcal{T}$-invariant Dirac mass is opposite on surfaces with $\mathcal{I}$-related Miller indices. Hence, in $\mathcal{I}$- and $\mathcal{T}$-symmetric HOTIs, there exists a crystal symmetry ($\mathcal{I}$ in the simplest HOTIs [31, 100, 102, 103, 105]) that relates the gapped surface phases resulting from either sign of the $\mathcal{T}$-symmetric Dirac mass [25]. Additionally, as discussed in SRefs. 3, 25, and 226, two $\mathcal{T}$-symmetric 2D insulators that differ only by the sign of the mass of a fourfold Dirac fermion at a TRIM point must differ topologically by a relative Kane-Mele $\mathbb{Z}_2$ index. However, it has previously been established that 2D TIs and trivial insulators are *not* related by crystal symmetry operations (*c.f.* SM 1B of SRef. 25). Hence, absent a second fourfold surface Dirac cone that is symmetry-stabilized in the decoupled limit and pinned to a TRIM point, the continuum theory of an unpaired, gapped, surface, fourfold Dirac cone that was previously symmetry-stabilized cannot describe *any* isolated $\mathcal{T}$-invariant 2D insulator (*i.e.* a 2D insulator that is not connected to a 3D bulk) [25], because the 2D insulator and its $\mathcal{I}$-symmetry-related partner on the opposing surface must *both* differ by a relative $\mathbb{Z}_2$ index *and* be related by a crystal symmetry. Therefore, $\mathcal{I}$-related HOTI surfaces, whose topologies are determined by the sign of only a *single* (fourfold) Dirac mass, are neither completely trivial

nor topological 2D insulators, but must instead represent anomalous "halves" of a 2D TI. Similar to the half-integer surface quantum Hall effect in AXIs [7, 8, 10, 13, 25, 33, 96, 106, 219–222], domain walls between halves of the QSH effect still bind topological 1D modes [25, 177] (though the modes are helical, instead of chiral). The 1D helical modes are, in fact, the characteristic hinge states of HOTIs [100, 102, 103, 105]. From this perspective, the anomalous halves of the QSH effect can be considered superposed, $\mathcal{T}$-reversed copies of the half-integer quantum Hall effect that remain anomalous under the preservation of global $\mathcal{T}$ and bulk $\mathcal{I}$ symmetries.

The presence of spin-charge-separated Kramers pairs of states (fluxons) [164–166, 169, 170] on HOTI surfaces with threaded π-flux (specifically spinons at half filling [215]) implies that, even though the magnetoelectric polarizability θ of HOTIs is trivial ($\theta \bmod 2\pi = 0$) [31–33, 103–105], there is still a quantized bulk response that may be observable in experiment. We leave further analysis of this response for future works [201].

B. Ground State Mapping Proofs for Defect and π-Flux States

In this section, we will use the many-body (Slater-determinant) ground states of pristine crystals to determine defect and flux responses beyond the $k \cdot p$ approximation previously employed in SN 2 A. For edge dislocations (SN 2 B 1) and π-flux tubes (SN 2 B 2) in particular, our arguments can be straightforwardly generalized to crystals with arbitrarily large dimensionality d.

We will here model each dislocation or flux tube as an interface between two thermodynamically large, d-D bulk systems, whose occupied electronic states in momentum space differ by the Brillouin zones of $(d-1)$-D phases. We will then subsequently deduce the presence of $(d-2)$-D or $(d-3)$-D bound states via the bulk-boundary correspondences of the $(d-1)$-D phases. Throughout this section, we will assume that any symmetries that enforce the topology of the $(d-1)$-D momentum-space subsystems are also preserved by the position-space dislocation or flux-tube geometry itself. Because we are assuming that the crystal without defects is insulating, any bound states are necessarily localized on the defects and flux tubes (or on their corners and ends), and decay exponentially away from them.

1. *Edge Dislocations in d-D Crystals*

Here, we derive the topological relations governing the appearance of bound states arising from the insertion of a set of $\mathcal{I}$-symmetric edge dislocations into an insulating system with arbitrary dimensionality (d-D). We first focus on an edge dislocation with Burgers vector $\boldsymbol{B} = \hat{y}$ for clarity [see SFig. 2 ***b*** in the main text], before generalizing to arbitrary Burgers vectors.

Let S be a d-dimensional insulating system defined on a lattice Λ of size $V_S = L_y V_S^{\perp}$, where L_y is the linear extent in y direction (we take the lattice spacing to be $a = 1$ along each of the lattice vectors) and $V_S^{\perp}$ denotes the $(d-1)$-dimensional volume spanning the remaining directions. We take S to be governed by a gapped single-particle Hamiltonian $\mathcal{H}$, and first consider the pristine system without dislocations. Making translation symmetry in the y direction explicit, and leaving translation in the remaining perpendicular directions implicit, we Fourier transform $\mathcal{H}$ to realize a Bloch Hamiltonian $\mathcal{H}(k_y)$ that acts on the degrees of freedom of $(d-1)$-dimensional slices (layers or rows) with size $V_S^{\perp}$ of the lattice. With periodic boundary conditions, and taking L_y to be an even integer without loss of generality, k_y is an element of the discrete Brillouin zone (BZ):

$$\mathrm{BZ}_S = \frac{2\pi}{L_y}\left\{-\left(\frac{L_y}{2}-1\right), \ldots, 0, \ldots, \left(\frac{L_y}{2}-1\right), \frac{L_y}{2}\right\}. \tag{74}$$

In the case of N occupied bands, the eigenstates of $\mathcal{H}(k_y)$ are given by:

$$\mathcal{H}(k_y)\left|u_\alpha(k_y)\right\rangle = E_\alpha(k_y)\left|u_\alpha(k_y)\right\rangle, \tag{75}$$

where $\alpha = 1 \ldots N$. Re-expressing the eigenstates of $\mathcal{H}(k_y)$ using second-quantization:

$$\left|u_\alpha(k_y)\right\rangle = c^\dagger_\alpha(k_y)\left|0\right\rangle, \tag{76}$$

where the orthonormality of the occupied eigenstates $\left|u_\alpha(k_y)\right\rangle$ implies that:

$$\{c_\alpha(k_y), c^\dagger_\beta(k_y)\} = \delta_{\alpha\beta}. \tag{77}$$

In this construction, the ground state of S takes the form:

$$\left|\mathrm{GS}_S\right\rangle = \prod_{k_y}^{\mathrm{BZ}_S}\prod_{\alpha=1}^{N} c^\dagger_\alpha(k_y)\left|0\right\rangle. \tag{78}$$

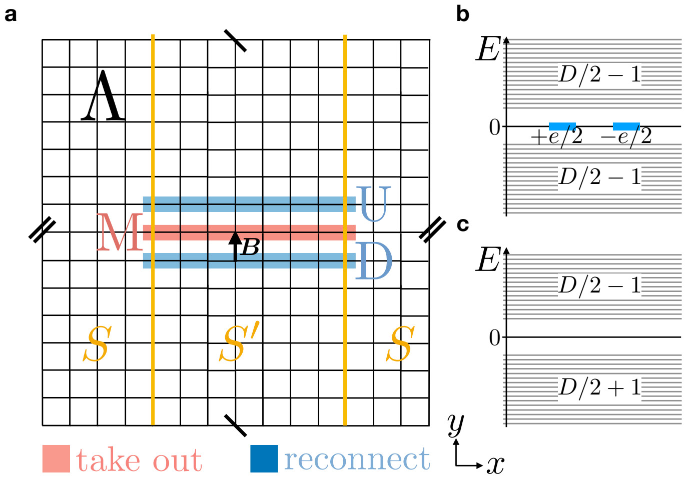

Supplementary Figure 5. **Schematic edge dislocation implementation.** ***a*** To create a pair of edge dislocations with Burgers vectors $\boldsymbol{B} = \hat{y}$ in a 2D crystal defined on a lattice Λ, we remove the sites belonging to an x-directed line M, and reconnect the sites above M – belonging to the line U – with those below M – belonging to the line D, as detailed in the text surrounding SEq. (144). ***b*** In the spectrum of a 2D $\mathcal{I}$-symmetric system calculated with open boundary conditions, a pair of dislocations can carry nontrivial topology that is indicated via dislocation bound states pinned to zero energy by a chiral (unitary particle-hole) symmetry Π, which is absent in real materials. In the topologically nontrivial phase, the conductance and valence bands are both missing one state with respect to the same spectrum calculated with periodic boundary conditions. ***c***. When Π is relaxed, however, the midgap states can be shifted out of the bulk gap. Nevertheless, the nontrivial bulk topology is still present in the system with the pair of dislocations, and is instead more generally indicated via a filling anomaly [33, 70, 141–143, 176]. Specifically, at half filling, there is a ground state degeneracy, arising from the choice of which midgap state should be occupied. Then, when one electron is added or removed, the system becomes gapped, but there is a mismatch in the number of states above and below the gap (depicted in ***b, c*** as centered at $E = 0$).

We note that in SEq. (78), the degrees of freedom along the perpendicular directions in $V_S^{\perp}$ are covered by the band index α, so that N may be very large.

An edge dislocation with $\boldsymbol{B} = \hat{y}$, located at a given y coordinate, realizes an interface – oriented parallel to the y direction – of S with a system S' that has size $V'_S = (L_y - 1)V_S^{\perp}$, but which otherwise has the same symmetry and topology as S. Here, we assume lattice relaxation away from the edge dislocation, so that both S and S' have translation symmetry in y direction, and also in all remaining directions away from the dislocation. While dislocations in real materials generically do not fully satisfy this requirement, the topological dislocation bound states that we uncover and analyze in this work are insensitive to the scale of translation-restoring lattice relaxation, provided that the bulk and interface gaps are large, such that the 0D defect states are well localized. Under the preservation of y-direction translation symmetry, S' is described by the Bloch Hamiltonian $\mathcal{H}(k'_y)$, where k'_y is instead an element of the discrete BZ:

$$\mathrm{BZ}_{S'} = \frac{2\pi}{L_y - 1}\left\{-\left(\frac{L_y}{2} - 1\right), \ldots, 0, \ldots, \left(\frac{L_y}{2} - 1\right)\right\}. \tag{79}$$

The ground state of S' ($|\mathrm{GS}_{S'}\rangle$) is formed from the occupied subspaces of $H(k'_y)$ with k'_y being an element of SEq. (79), such that $|\mathrm{GS}_{S'}\rangle$ is given by:

$$|\mathrm{GS}_{S'}\rangle = \prod_{k'_y}^{\mathrm{BZ}_{S'}} \prod_{\alpha=1}^{N} c^{\dagger}_{\alpha}(k'_y)\,|0\rangle\,. \tag{80}$$

Importantly, in SEqs. (79) and (80), the $k'_y = \pi$ subspace of the continuum BZ is never sampled, and thus does not contribute, even though $\mathrm{BZ}_{S'}$ approaches BZ_S as L_y goes to infinity. To restate, as long as L_y is discretely valued, only BZ_S contains $k_y = \pi$. Therefore, a $\boldsymbol{B} = \hat{y}$ edge dislocation forms an interface between two systems S and S', of which only one system has a ground state in which $k_y = \pi$ is occupied.

Comparing SEqs. (74) and (79) establishes that there is a mismatch between $|\mathrm{GS}_S\rangle$ and $|\mathrm{GS}_{S'}\rangle$ that is given by the $(d-1)$-D (possibly topological) contribution of $\mathcal{H}(\pi)$ to the ground state of S:

$$|\mathrm{GS}_S\rangle \approx \prod_{\alpha=1}^{N} c_\alpha^\dagger(\pi)\,|\mathrm{GS}_{S'}\rangle\,, \tag{81}$$

where by $\approx$ we mean that the states on either side of the equation are adiabatically related to each other. Namely, the $\approx$ symbol indicates that the states on the two sides of SEq. (81) only differ by the precise BZ location of the low-symmetry k_y momenta (neither $k_y = 0$ nor $k_y = \pi$) contained in the ground states of S and S'. BZ manifolds at such choices of k_y break $\mathcal{I}$ symmetry, and therefore do not contribute any $\mathcal{I}$-protected topological indices to the summed topology. The $(d-1)$-D contribution of $\mathcal{H}(\pi)$ can be trivial or (crystalline or polarization) topological; in the latter case, the edge dislocation acts as a boundary that can host topologically protected states by the bulk-boundary (domain-wall) correspondence between S and S'.

To be precise, the ground state of S is adiabatically related to a state given by the (appropriately antisymmetrized) tensor product of the ground state of $\mathcal{H}(k_y = \pi)$ with that of S'. We relate this to the bulk-boundary (domain-wall) correspondence between S and S' by noting that, under the tensor product, which is physically implemented by stacking (*i.e.* superposition), free-fermion topological phases form an additive group [255], which allows us to "subtract" ($\ominus$) $|\mathrm{GS}_{S'}\rangle$ from both sides of the dislocation in SFig. 5 $\boldsymbol{a}$. We then conclude that the ground state of the subsystem of the dislocation is, via this subtraction, adiabatically related to the ground state of $\mathcal{H}(k_y = \pi)$, which is given by:

$$|\mathrm{GS}_S\rangle \ominus |\mathrm{GS}_{S'}\rangle \approx \prod_{\alpha=1}^{N} c_\alpha^\dagger(\pi)\,|0\rangle\,, \tag{82}$$

with respect to the vacuum $|0\rangle$.

The condition for anomalous bound states to be present is that all (unitary and antiunitary) symmetries that protect the topology of $\mathcal{H}(\pi)$ remain enforced by the defect geometry. Importantly, we do not require individual edge dislocations to preserve these symmetries on their own, but we do require the set of dislocations to be arranged in a manner in which the $(d-1)$-D surface connecting them does preserve the global symmetries enforcing the topology of $\mathcal{H}(k_y)$.

We also emphasize that there exist several distinct notions of topological nontriviality. In the language of Topological Quantum Chemistry (TQC) [152–155, 243], the occupied bands of topological (crystalline) insulators (TIs and TCIs) cannot be represented in terms of maximally localized, symmetric Wannier functions [256]. However, the results in this section also apply if the ground state of $\mathcal{H}(k_y = \pi)$ [SEq. (82)] carries the topology of an OAL, whose occupied bands *can* be represented in terms of maximally localized Wannier functions, but not on the positions of the underlying atoms [152]. Specifically, if the ground state of $\mathcal{H}(k_y)$ in SEq. (81) is an OAL, then a dislocation still acts as a boundary for it, and can still host protected (0D) states due to excess charge (or spin) [30, 70, 95, 176, 215–218]. As shown in recent works [33, 70, 100, 176], fragile TIs (FTIs) also carry the same boundary spin and charge as OALs, and thus our analysis applies to FTIs as well. For simplicity, in this section, we group strong TIs, TCIs, OALs, and FTIs together under the general label "topological," unless stated otherwise.

In order to generalize to arbitrary Burgers vectors, we phrase the above in coordinate-independent terms. Making the full translation symmetry of $\mathcal{H}$ explicit, we consider its Fourier transform $\mathcal{H}(\boldsymbol{k})$, where $\boldsymbol{k}$ is an element of the d-dimensional Brillouin zone. Hence, an edge dislocation with Burgers vector $\boldsymbol{B}$ forms an interface between two systems S and S' that differ by the ground state of a $(d-1)$-dimensional momentum-space subsystem with the Bloch Hamiltonian:

$$\mathcal{H}(\boldsymbol{k}_\perp), \quad \boldsymbol{k}_\perp \in \{\boldsymbol{k}\,|\,\boldsymbol{B}\cdot\boldsymbol{k} = \pi\}. \tag{83}$$

2. *π-Flux States in d-D Crystals*

In this section, we will derive the topological relations governing the appearance of bound states arising from the insertion of a set of π-flux tubes into an insulating system with arbitrary dimensionality (d-D). We first focus on the physically relevant cases of two and three dimensions before briefly generalizing to insulators with arbitrarily large dimensionality d.

A pair of magnetic fluxes (flux tubes) with strength $\pm\phi$ can be introduced in a 2D (3D) system S, containing $L_x L_y$ ($L_x L_y L_z$) lattice sites, by multiplying all of the hoppings across a line (plane) M of sites in the crystal lattice by a factor of $e^{\mathrm{i}\phi}$ in one direction and $e^{-\mathrm{i}\phi}$ in the other. This is known as the Peierls substitution [257], and here specifically

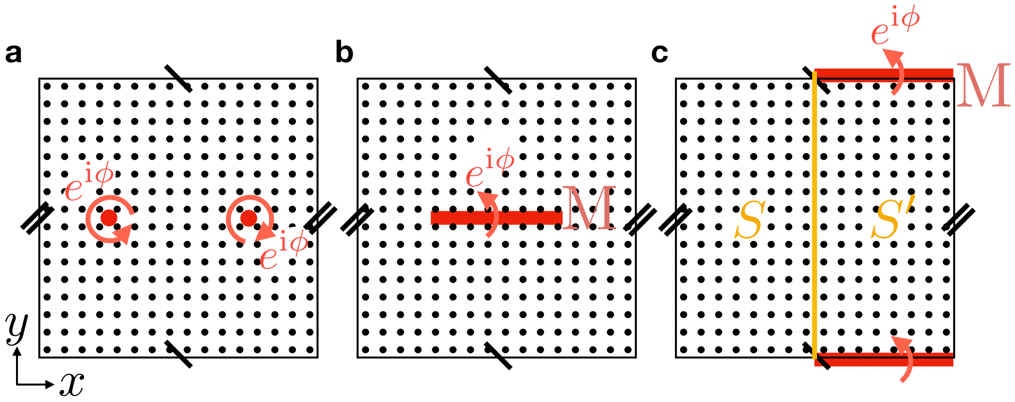

Supplementary Figure 6. **Schematic magnetic flux implementation.** ***a***-***c*** A pair of magnetic $\pm\phi$-fluxes (flux tubes in 3D) in the (x, y)-plane, shown in ***a***, can be implemented by multiplying all hoppings across a line (plane) with length L_M in the x direction connecting the two flux cores (tubes) by $e^{i\phi}$, as shown in ***b***. This is known as the Peierls substitution [257], and corresponds to a gauge choice for the electromagnetic vector potential. In a system with otherwise periodic boundary conditions, the Peierls substitution in ***b*** is equivalent to implementing twisted boundary conditions in a part of the system, as shown in ***c***. The flux cores (tubes) therefore form an interface between the system with standard periodic boundary conditions and the system with twisted boundary conditions (shown in the y direction in ***c***).

represents a convenient gauge choice: the only physical (gauge-invariant) requirement is that the hoppings encircling a flux core (tube) in real space accumulate a phase of $e^{\pm i\phi}$. We orient the line (plane) M containing the $e^{i\phi}$ phase shift (red line in SFig. 6 ***b***) along the x direction in 2D (along the x and z directions in 3D), and choose its linear extent to be half of the linear size (here L_M) of our full system (see also SFig. 6 ***a***-***c***). While the proofs in this section and in SN 2 A 3 employ this particular straight-line (plane) electromagnetic gauge, ultimately, the physical observables obtained in both sections (bound charge, spin, or chiral or helical modes) appear at gauge-invariant locations with topologically quantized values (or multiplicities).

As shown in SFig. 6 ***c***, in the thermodynamic limit, the flux core (tube) realizes a $(d-2)$-D interface between the original, d-D unmodified system S (with periodic boundary conditions in the y direction), and a system S' with twisted boundary conditions in the y direction (in the straight-line gauge in SFig. 6). This follows from noting that, in S' realized with the gauge in SFig. 6, there are a total of L_M bonds in the positive y direction that cross M and carry a rotated hopping phase of $e^{i\phi}$, and that there are L_M bonds in the negative y direction with a hopping phase rotated by $e^{-i\phi}$.

We will now individually analyze S and S' in order to deduce the (gauge-invariant) electronic structure of the boundary between S and S' by using the bulk-boundary (domain-wall) correspondence between S and S'. To begin, let S be described by a gapped Bloch Hamiltonian $\mathcal{H}(k_y)$, where we suppress other momentum labels for notational simplicity. Let L_y be even without loss of generality. As in our previous analysis in SN 2 B 1, with periodic boundary conditions, k_y is here an element of the discrete BZ:

$$\mathrm{BZ}_S = \frac{2\pi}{L_y}\left\{-\left(\frac{L_y}{2}-1\right),\ldots,0,\ldots,\left(\frac{L_y}{2}-1\right),\frac{L_y}{2}\right\}, \tag{84}$$

where for simplicity, we have chosen units in which the lattice spacings $a_{x,y,z} = 1$. In the case of N occupied bands, the occupied eigenstates of $\mathcal{H}(k_y)$ are given by:

$$\mathcal{H}(k_y)\left|u_\alpha(k_y)\right\rangle = E_\alpha(k_y)\left|u_\alpha(k_y)\right\rangle, \tag{85}$$

where $\alpha = 1\ldots N$. Reexpressing the eigenstates of $\mathcal{H}(k_y)$ using second-quantization:

$$\left|u_\alpha(k_y)\right\rangle = c^\dagger_\alpha(k_y)\left|0\right\rangle, \tag{86}$$

where the orthonormality of the occupied eigenstates $|u_\alpha(k_y)\rangle$ implies that:

$$\{c_\alpha(k_y), c^\dagger_\beta(k_y)\} = \delta_{\alpha\beta}. \tag{87}$$

In this construction, the ground state of S again takes the form:

$$\left|\mathrm{GS}_S\right\rangle = \prod_{k_y}^{\mathrm{BZ}_S}\prod_{\alpha=1}^{N} c^\dagger_\alpha(k_y)\left|0\right\rangle. \tag{88}$$

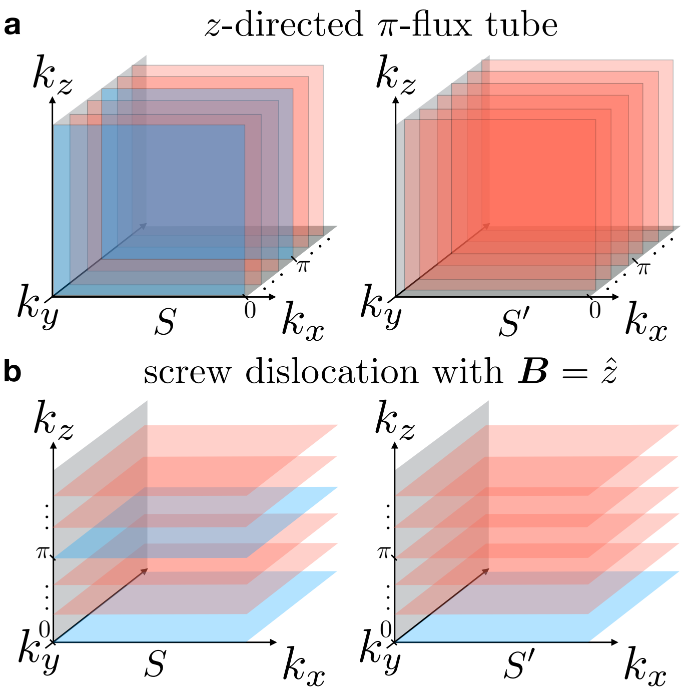

Supplementary Figure 7. **Flux and dislocation systems in discretized momentum space.** 3D Brillouin zones for the systems S and S' as respectively defined in SN 2 B 2 and 2 B 3 for the cases of ***a*** a z-directed π-flux tube and ***b*** a screw dislocation with Burgers vector $\boldsymbol{B} = \hat{z}$. ***a*** A z-directed flux tube lies at the boundary between a pristine system S and, in the gauge in SFig. 5, a system S' in which all momenta k_y are shifted by π/L_y [SEq. (89)]. Assuming L_y to be even and S and S' to be $\mathcal{I}$-symmetric, S contains the (possibly topologically nontrivial) BZ planes at $k_y = 0, \pi$ (indicated in blue), in addition to planes at intermediate momenta (indicated in red), which are generically equivalent to 2D insulators without edge or corner states [discounting for now the case of 3D weak Chern (quantum anomalous Hall) insulators]. In contrast, the discrete BZ of S' [SEq. (90)] only contains the intermediate values $k_y \neq 0, \pi$. Therefore, an interface between S and S' whose boundary is a π-flux tube will exhibit (given the preservation of bulk symmetries) the (gauge-invariant) boundary modes of the combined (superposed [255]) topologies of the Hamiltonians of the $k_y = 0, \pi$ planes of S [SEq. (92)]. ***b*** A screw dislocation with Burgers vector $\hat{z}$ forms an interface between a pristine system S and a system S' in which the momenta k_y are shifted by k_z-dependent amounts k_z/L_y [SEq. (99)]. Assuming $L_{y,z}$ to be even, S contains the (possibly topologically nontrivial) BZ planes at $k_z = 0, \pi$ (indicated in blue), in addition to planes at intermediate momenta (indicated in red), which are, like those in ***a***, generically equivalent to 2D insulators without edge or corner states. In contrast to that of S, the discrete BZ of S' only contains the $\mathcal{I}$-symmetric plane $k_z = 0$, and *does not contain* $k_z = \pi$ [SEq. (104)]. Therefore, an interface between S and S' whose boundary is a screw dislocation with an odd-integer Burgers vector will exhibit (given the preservation of bulk symmetries) the (gauge-invariant) boundary modes of the $k_z = \pi$ plane of the pristine insulator S [SEq. (105)].

Conversely, in S', the twisted boundary conditions that arise from the flux insertion shift all momenta k_y, such that:

$$\mathrm{BZ}_{S'} = \frac{\phi}{L_y} + \mathrm{BZ}_S. \tag{89}$$

Specializing to the ($\mathcal{T}$-invariant) case of $\phi = \pi$ highlighted in this work, SEqs. (84) and (89) combine to realize a finite BZ in which all k_y have become shifted by π/L_y:

$$\mathrm{BZ}_{S'} = \frac{2\pi}{L_y}\left\{-\left(\frac{L_y - 1}{2}\right), \ldots, -\frac{1}{2}, \frac{1}{2}, \ldots, \left(\frac{L_y - 1}{2}\right)\right\}. \tag{90}$$

Crucially, the finite set $\mathrm{BZ}_{S'}$ includes neither $k_y = 0$ nor $k_y = \pi$ (SFig. 7 ***a***). Using SEq. (90), the corresponding ground state of S' is then given by:

$$|\mathrm{GS}_{S'}\rangle = \prod_{k_y}^{\mathrm{BZ}_{S'}} \prod_{\alpha=1}^{N} c^\dagger_\alpha(k_y)\,|0\rangle\,. \tag{91}$$

As previously in SN 2 B 1, comparing the ground states of S and S', we find that a flux core (or tube) can host topologically protected boundary states if $|\mathrm{GS}_S\rangle$ and $|\mathrm{GS}_{S'}\rangle$ "differ" ($\ominus$) by a topologically nontrivial phase [as defined in the text surrounding SEq. (82)].

In this work, we are focused on insulators with spatial inversion symmetry ($\mathcal{I}$). The operation of $\mathcal{I}$ takes $k_y \to -k_y$, such that in both 2D and 3D insulators, there are only two $\mathcal{I}$-invariant values $k_y = 0, \pi$. We may also re-express 2D (3D) insulators as (possibly trivial) k_y-indexed pumping cycles of 1D (2D) insulators in which only the values of $k_y = 0, \pi$ correspond to 1D (2D) insulators with $\mathcal{I}$ (and optionally $\mathcal{T}$) symmetry [7, 8, 13, 30, 32, 33, 70, 249–251, 258]. Hence, if the weak Chern numbers of a 3D $\mathcal{I}$-symmetric insulator are zero [*i.e.* the bulk is not a quantum anomalous Hall insulator (QAH) insulator [42, 75, 155, 174]], then the only possible topologically nontrivial contributions to the difference $|\mathrm{GS}_S\rangle \ominus |\mathrm{GS}_{S'}\rangle$ can come from $\mathcal{H}(k_y = 0, \pi)$ [SEqs. (88) and (91)]. Furthermore, because there are no 1D topological phases in the absence of crystal symmetry [215–218, 258–260], then, in an $\mathcal{I}$- (and optionally $\mathcal{T}$-) symmetric 2D insulator, $|\mathrm{GS}_S\rangle \ominus |\mathrm{GS}_{S'}\rangle$ also only contains contributions from $\mathcal{H}(k_y = 0, \pi)$. Specifically, in both 2D and 3D, the real-space interface between S and S' in SFig. 6 ***c*** carries the summed (superposed) $(d-1)$-D topologies of $\mathcal{H}(0)$ and $\mathcal{H}(\pi)$. While the location of the interface is gauge-dependent, the location of its boundaries, the flux cores (and tubes), are gauge-independent. Therefore, exploiting that topological phases form an additive group [255], we formally express the ground state of the interface between S and S' as:

$$|\mathrm{GS}_S\rangle \ominus |\mathrm{GS}_{S'}\rangle \approx \left[\prod_{\alpha=1}^{N} c^\dagger_\alpha(0) \otimes \prod_{\alpha=1}^{N} c^\dagger_\alpha(\pi)\right] |0\rangle\,, \tag{92}$$

with respect to the vacuum $|0\rangle$. To summarize, in $\mathcal{I}$- (and optionally $\mathcal{T}$-) symmetric 2D and 3D insulators [in which the 3D insulators have vanishing weak Chern numbers], the flux tubes at the interface between S and S' will only exhibit (gauge-invariant) topological (anomalous) boundary modes if $\mathcal{H}(0) \otimes \mathcal{H}(\pi)$ is topologically equivalent to a phase with anomalous boundary modes (*i.e*, a TI, TCI, or filling-anomalous OAL or FTI).

Unlike previously in SN 2 B 1, SEq. (92) implies the possibility that $|\mathrm{GS}_S\rangle \ominus |\mathrm{GS}_{S'}\rangle$ is topologically trivial even though $\mathcal{H}(0, \pi)$ are individually topologically nontrivial. Specifically, if the topology of $\mathcal{H}(0, \pi)$ is diagnosed by a set of indices $\mathbb{Z}_{m_1} \otimes \mathbb{Z}_{m_2} \otimes \mathbb{Z}_{m_3} \otimes \ldots$ where $m_i \in \mathbb{Z}^+$, then, if both $\mathcal{H}(0)$ and $\mathcal{H}(\pi)$ have topological indices that sum to zero [*e.g.* $\mathcal{H}(0)$ and $\mathcal{H}(\pi)$ both have nontrival $\mathbb{Z}_2$ 2D TI indices [3, 5, 6]], then $\mathcal{H}(0) \otimes \mathcal{H}(\pi)$ is necessarily topologically trivial. Hence, SEq. (92) implies that $|\mathrm{GS}_S\rangle \ominus |\mathrm{GS}_{S'}\rangle$ is only topologically nontrivial if $\mathcal{H}(0)$ and $\mathcal{H}(\pi)$ do not have canceling topological indices, which, if the $\mathbb{Z}$-valued Chern numbers are zero, can only occur in a strong topological (insulating) phase in which k_y indexes a nontrivial pumping cycle [7, 8, 13, 30, 32, 33, 249–251].

Finally, while in this section we have restricted to flux tubes in 2D and 3D insulators, it is straightforward to generalize our arguments to threaded π-flux tubes in insulators with arbitrarily large dimensionality d. Specifically, to generalize the Peierls substitution employed in this section (SFig. 5) to higher dimensions, we replace the 1D line or 2D plane M with modified hoppings in SFig. 6 ***c*** with a $(d-1)$-D *hyperplane* with constant y-coordinate. Even if the boundary between S and S' has a dimensionality larger than 2, the analysis employed in this section to derive and analyze SEq. (92) still applies without further modification [though finer analysis is required if $\mathcal{H}(0, \pi)$ exhibit $\mathbb{Z}$- or $\mathbb{Z}_n$-valued $(n > 2)$ topological indices, analogous to the requirement in 3D that the QAH weak Chern numbers vanish, see the text surrounding SEq. (92)].

3. *Screw Dislocations in 3D Crystals*

In this section, we will derive the topological relations governing the appearance of bound states arising from the insertion of a set of screw dislocations into an insulating system. Because the lattice displacements in screw dislocations are more complicated to visualize than those in edge dislocations [127], we will restrict consideration in this section to the more familiar, experimentally relevant case of screw dislocations in 3D insulating crystals. Unlike the edge dislocations analyzed in SN 2 B 1, a screw dislocation corresponds to a dislocation line of displacements that is parallel to its Burgers vector (see also SN 2 A 2 and SRef. 127). We consider a pair of $\boldsymbol{B} = \hat{z}$ screw dislocations, one left-handed and the other right-handed [see SEq. (42) and the surrounding text]. To implement a right- (left-) handed screw dislocation with Burgers vector $\boldsymbol{B} = \hat{z}$, we multiply the lattice hoppings across a given line emanating from the dislocation in the (x, y)-plane by a phase of $e^{\mathrm{i}k_z}$ $(e^{-\mathrm{i}k_z})$, which originates from the net dislocation translation in the positive (negative) z direction [127]. This construction, shown in SFig. 8, is equivalent to the insertion of a pair of flux tubes (SFig. 6) under the replacement $\phi \to k_z$, and is hence reminiscent of the Peierls substitution for π-flux tubes [257] previously employed in SN 2 B 2. Specifically, with periodic boundary conditions in the z direction, a pair of screw dislocations is functionally equivalent to modifying the Bloch Hamiltonian $\mathcal{H}(\boldsymbol{k})$ with a momentum-dependent flux $\phi = k_z$. Therefore, we can model a $\boldsymbol{B} = \hat{z}$ screw dislocation in a 3D insulator as inhabiting the (linear) boundary of a 2D interface between a system S with periodic boundary conditions in all directions and a system S'

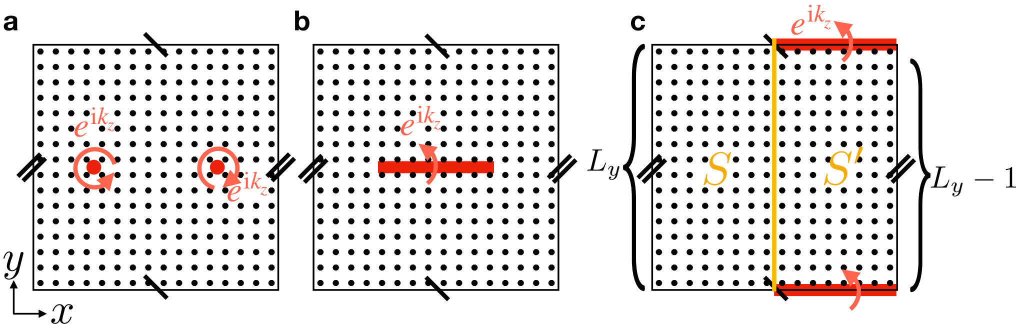

Supplementary Figure 8. **Schematic screw dislocation implementation.** ***a-c*** A pair of $\boldsymbol{B} = \hat{z}$ screw dislocations, shown in ***a***, can be implemented by multiplying all hoppings across a plane with length L_M in the x direction connecting the two screws by e^{ik_z}, as shown in ***b*** (keeping periodic boundary conditions in z direction, such that k_z is a well-defined momentum quantum number). This is equivalent to implementing k_z-dependent twisted boundary conditions in a part of the system, as shown in ***c***. The screw dislocations therefore form an interface between the system with standard periodic boundary conditions and the system with k_z-dependent twisted boundary conditions (shown in the y direction in ***c***, see also SFig. 9).

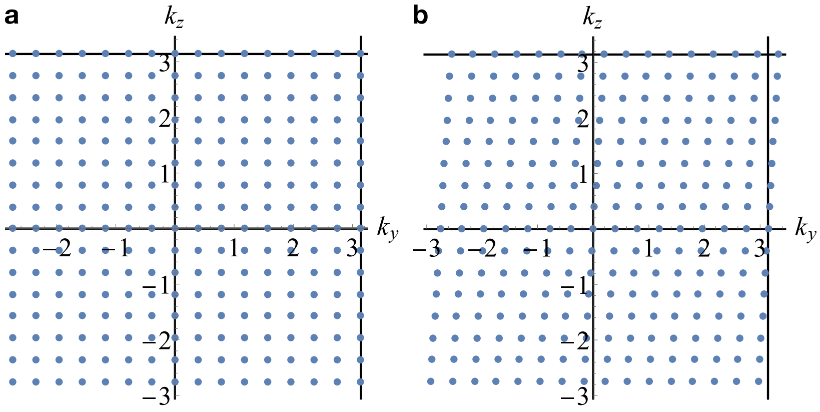

Supplementary Figure 9. **Brillouin-zone mismatch due to $\boldsymbol{B} = \hat{z}$ screw insertion.** ***a*** The (k_y, k_z) BZ of system S in SN 2 B 3 for the choice $L_y = L_z = 16$ in SEqs. (93) and (94). The remaining momentum k_x is measured perpendicular to the (k_y, k_z)-plane, and is not shown here, as it remains unaffected by our screw dislocation implementation. Both the $k_z = 0$ and the $k_z = \pi$ planes contain four high-symmetry ($\mathcal{I}$-invariant) momenta (two each for the BZ lines containing the $\mathcal{I}$-symmetric momenta $k_x = 0, \pi$). ***b*** The (k_y, k_z) BZ of system S' in SN 2 B 3 for the choice $L_y = L_z = 16$ in SEqs. (94) and (99). Crucially, only the $k_z = 0$ plane contains four high-symmetry ($\mathcal{I}$-invariant) momenta, while the $k_z = \pi$ plane does not contain any $\mathcal{I}$-invariant k points. As a consequence, the screw dislocation, which lies at the boundary between S and S', binds topological states governed by the domain-wall correspondence between S and S' *in only the* $k_z = \pi$ *BZ plane.*

with twisted boundary conditions (TBC) in the y direction for which the twist phase factor is momentum-dependent (e^{ik_z}, see SFig. 8).

As previously in SN 2 B 2, we will now individually analyze S and S' in order to deduce the (gauge-invariant) electronic structure of the interface between S and S' by using the bulk-boundary (domain-wall) correspondence between S and S'. To begin, let S be described by a gapped Bloch Hamiltonian $\mathcal{H}(k_y, k_z)$, where we suppress the momentum k_x for notational simplicity. Let L_y and L_z be even without loss of generality. With periodic boundary conditions, k_y is an element of the discrete BZ:

$$\mathrm{BZ}_{S,k_y} = \frac{2\pi}{L_y}\left\{-\left(\frac{L_y}{2}-1\right), \ldots, 0, \ldots, \left(\frac{L_y}{2}-1\right), \frac{L_y}{2}\right\}, \tag{93}$$

where for simplicity, we have chosen units in which the lattice spacings $a_{x,y,z} = 1$, and where the k_z superscript in SEq. (93) implies a (here trivial) dependence of the discretization of k_y on k_z. Unlike previously in SN 2 B 1 and 2 B 2,

k_z is here also an element of a discrete BZ:

$$\mathrm{BZ}_{S,k_z} = \frac{2\pi}{L_z}\left\{-\left(\frac{L_z}{2}-1\right),\ldots,0,\ldots,\left(\frac{L_z}{2}-1\right),\frac{L_z}{2}\right\}. \tag{94}$$

In the case of N occupied bands, the occupied eigenstates of $\mathcal{H}(k_y,k_z)$ are given by:

$$\mathcal{H}(k_y,k_z)\left|u_\alpha(k_y,k_z)\right\rangle = E_\alpha(k_y,k_z)\left|u_\alpha(k_y,k_z)\right\rangle, \tag{95}$$

where $\alpha = 1\ldots N$. Reexpressing the eigenstates of $\mathcal{H}(k_y,k_z)$ using second-quantization:

$$\left|u_\alpha(k_y,k_z)\right\rangle = c^\dagger_\alpha(k_y,k_z)\left|0\right\rangle, \tag{96}$$

where the orthonormality of the occupied eigenstates $\left|u_\alpha(k_y,k_z)\right\rangle$ implies that:

$$\{c_\alpha(k_y,k_z), c^\dagger_\beta(k_y,k_z)\} = \delta_{\alpha\beta}. \tag{97}$$

In this construction, the ground state of S takes the form (see SFig. 9 ***a***):

$$\left|\mathrm{GS}_S\right\rangle = \prod_{k_z}^{\mathrm{BZ}_{S,k_z}}\prod_{k_y}^{\mathrm{BZ}_{S,k_y}}\prod_{\alpha=1}^{N} c^\dagger_\alpha(k_y,k_z)\left|0\right\rangle. \tag{98}$$

with respect to the vacuum $\left|0\right\rangle$, where all products are appropriately anti-symmetrized.

Conversely, in S', the twisted boundary conditions that arise from the screw dislocations (as illustrated in SFig. 8) shift all momenta k_y by a k_z-dependent amount, such that k_y in S' is an element of the discrete BZ:

$$\mathrm{BZ}^{k_z}_{S',k_y} = \frac{k_z}{L_y} + \mathrm{BZ}_{S,k_y}. \tag{99}$$

where BZ_{S,k_y} is given in SEq. (93). SEq. (99) implies that in system S' at fixed k_z, k_y is sampled from the k_z-dependent set $\mathrm{BZ}^{k_z}_{S',k_y}$, so that the tuple of momenta (k_y,k_z) is drawn from

$$\bigcup_{k_z}^{\mathrm{BZ}_{S',k_z}}\bigcup_{k_y}^{\mathrm{BZ}^{k_z}_{S',k_y}}(k_y,k_z). \tag{100}$$

However, even though the screw dislocations in this section are z-directed (SFig. 7 ***b***), they *do not* affect the discretization of k_z, such that:

$$\mathrm{BZ}_{S',k_z} = \mathrm{BZ}_{S,k_z}, \tag{101}$$

where BZ_{S,k_z} is given in SEq. (94). The corresponding ground state of S' is then given by (see SFig. 9 ***b***):

$$\left|\mathrm{GS}'_S\right\rangle = \prod_{k_z}^{\mathrm{BZ}_{S',k_z}}\prod_{k_y}^{\mathrm{BZ}^{k_z}_{S',k_y}}\prod_{\alpha=1}^{N} c^\dagger_\alpha(k_y,k_z)\left|0\right\rangle. \tag{102}$$

Crucially, if we restrict to 3D insulators with only $\mathcal{I}$ (and optionally $\mathcal{T}$) symmetry, SEqs. (99), (101), and (102) can be reformulated to realize a simple result. Namely, first focusing on the $k_z = \pi$ plane in S', SEq. (99) implies that the lines $k_y = 0,\pi$ are absent in the discrete BZ. Therefore, the action of $\mathcal{I}$ (and $\mathcal{T}$) symmetry no longer takes the $k_z = \pi$ plane back to itself, such that in S', the $k_z = \pi$ plane can no longer exhibit topology enforced by $\mathcal{I}$ or $\mathcal{T}$. Hence, when restricted to $\mathcal{I}$-symmetric insulators, the ground state of S' can be adiabatically deformed from SEq. (102) to:

$$\left|\mathrm{GS}'_S\right\rangle = \prod_{\tilde{k}_z}^{\tilde{\mathrm{BZ}}_{S',\tilde{k}_z}}\prod_{\alpha=1}^{N} c^\dagger_\alpha(\tilde{k}_z)\left|0\right\rangle, \tag{103}$$

where $\tilde{k}_z$ is now an element of a discrete BZ:

$$\tilde{\mathrm{BZ}}_{S',\tilde{k}_z} = \frac{2\pi}{L_z}\left\{-\left(\frac{L_z}{2}-\frac{1}{2}\right),\ldots,0,\ldots,\left(\frac{L_z}{2}-\frac{1}{2}\right)\right\}. \tag{104}$$

that *does not* contain $\tilde{k}_z = \pi$. Specifically, because the topology of a 3D $\mathcal{I}$- (and optionally $\mathcal{T}$-) symmetric insulator (that lacks additional rotation and reflection symmetries) is entirely determined by the topologies of the 2D Hamiltonians of the $k_{x,y,z} = 0, \pi$ planes [excluding 3D quantum anomalous Hall insulators, see the discussion preceding SEq. (92)], then the ground state of S' can only contain topologically nontrivial contributions from the $\mathcal{I}$-symmetric Hamiltonian of the $k_z = 0$ ($\tilde{k}_z = 0$) plane.

As previously in SN 2 B 1 and 2 B 2, we next compare the topology of S and S'. Using the definition of "subtraction" ($\ominus$) established in the text surrounding SEq. (82), we find that SEq. (102) implies that a screw dislocation can host topologically protected boundary states if $|\text{GS}_S\rangle$ and $|\text{GS}_{S'}\rangle$ "differ" ($\ominus$) by a topologically nontrivial phase. This can be summarized by stating that the gauge-invariant screw dislocations represent the boundary of a 2D insulator whose ground state is given by:

$$|\text{GS}_S\rangle \ominus |\text{GS}_{S'}\rangle = \prod_{\alpha=1}^{N} c^\dagger_\alpha(k_z = \pi)\,|0\rangle\,. \tag{105}$$

The screw dislocations therefore bind the 1D or 0D boundary modes of the Hamiltonian of the $k_z = \pi$ plane of S, provided that the dislocation geometry preserves $\mathcal{I}$ symmetry. Generalizing to arbitrarily oriented screw dislocations, we recover SEq. (83), in agreement with the results of SN 2 A 2.

3. WEAK FRAGILE AND OBSTRUCTED-ATOMIC-LIMIT INDICES FOR 2D DISLOCATION AND 3D HEND STATES

A. The 2D Weak SSH Invariant M^{SSH}_ν

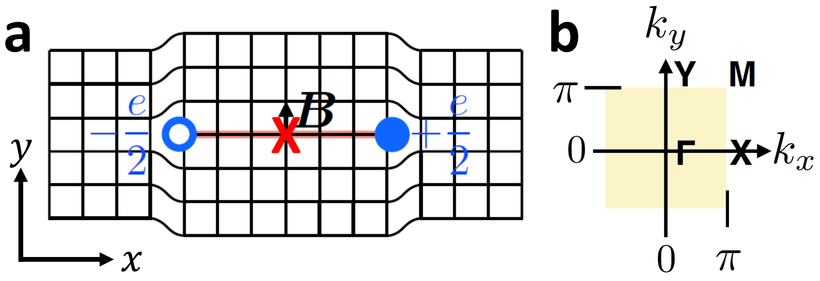

Supplementary Figure 10. **0D dislocation states in 2D crystals.** ***a*** A pair of dislocations with Burgers vectors $\boldsymbol{B} = \hat{y}$ in an $\mathcal{I}$-symmetric 2D insulator without spinful $\mathcal{T}$ symmetry. ***b*** The 2D Brillouin zone (BZ) of the insulator in ***a***. Under the preservation of $\mathcal{I}$ (red $\times$), the dislocations in ***a*** bind filling-anomalous (SRefs. 33, 70, 100, and 176 and SN 2 B 1) charges $\pm e/2$ if the $k_y = \pi$ BZ line exhibits a nontrivial polarization [6, 215–218, 246, 258, 261] n_{YM} when measured with respect to the x-coordinates of the valence atomic orbitals [SEq. (110)]. More generally, an $\mathcal{I}$-symmetric dislocation pair with Burgers vector $\boldsymbol{B}$ binds anomalous charge if $\boldsymbol{B} \cdot \boldsymbol{M}^{\text{SSH}}_\nu \bmod 2\pi = \pi$ [SEq. (106)]. If $\mathcal{T}$ symmetry is additionally present, then the dislocations will instead bind Kramers pairs of spin-charge-separated solitons, and specifically carry the same spin-charge relations as the solitons in polyacetylene (see SN 2 A and SRefs. 215–218). Using SEq. (110), we will specifically identify the presence of dislocation solitons in PbTe monolayers in SN 6 A.

In this section, we will derive the 2D weak polarization invariant $\boldsymbol{M}^{\text{SSH}}_\nu$. As discussed in the main text and shown in SN 2 B 1, an $\mathcal{I}$-symmetric 2D insulator with rectangular lattice vectors can bind 0D electronic states on pairs of dislocations that are related by a bulk $\mathcal{I}$ center (red $\times$ in SFig. 10 ***a***). Specifically, a pair of dislocations with defect Burgers vectors $\boldsymbol{B} = \hat{\imath}$ maps the momentum-space (polarization) topology of the $k_i = \pi$ line of the 2D BZ (SFig. 10 ***b***) to the real-space line spanning the dislocations (red line in SFig. 10 ***a***). If the real-space crystal with two dislocations preserves the momentum-space symmetries of the $k_i = \pi$ line of the 2D BZ modulo reciprocal lattice translations (here $\mathcal{I}$), then the polarization topology of the Hamiltonian of $k_i = \pi$ implies the presence of end (dislocation) charges in real space. That is, if the Hamiltonian of the $k_i = \pi$ line carries a nontrivial polarization (or *time-reversal* polarization [6, 258], if spinful $\mathcal{T}$ symmetry is also present) as measured relative to the unobstructed atomic limit of the valence atomic orbitals [152], then the dislocations will bind 0D states (or bound spin and charge in the more general case of relaxed particle-hole symmetry). The momentum-space (weak) polarization topology can be summarized for an $\mathcal{I}$- (and optionally $\mathcal{T}$-) symmetric 2D system as:

$$\boldsymbol{M}^{\text{SSH}}_\nu = \pi(n_{XM}, n_{YM}), \tag{106}$$

where n_{XM}/n_{YM} is the Su-Schrieffer-Heeger (SSH) polarization invariant [25, 215–218] (or time-reversal polarization [6, 258], if states are additionally doubled under $\mathcal{T}$ symmetry) of the BZ edge line $k_{x/y} = \pi$ (SFig. 10 ***b***).

We next define the polarization invariants n_{XM}/n_{YM}. We will specifically focus on n_{XM}, with the understanding that n_{YM} can analogously be derived by exchanging $x \leftrightarrow y$ in the following text. It has been shown in numerous previous works [8, 10, 246] that the polarization (and time-reversal polarization) of a 1D Hamiltonian $\mathcal{H}(k_i)$ is directly related to the Berry phase γ (or *partial* Berry phase if $\mathcal{T}$ is additionally present [6, 258]). In 1D crystals, the presence of rod group $\mathcal{I}$ symmetry (*i.e.* an inversion symmetry for which $\mathcal{I}^2 = +1$ independent of the presence of spinful electrons) [70, 262, 263] allows the immediate calculation of γ:

$$\gamma = \frac{\pi}{2}\left[1 - \xi(0)\xi(\pi)\right], \tag{107}$$

where $\xi(k_D)$ is given by the product of the parity eigenvalues of the occupied bands at the 1D TRIM point $k_D = 0, \pi$ (or the parity eigenvalues per Kramers pair if states are doubled by spinful $\mathcal{T}$ [8]). Hence, for a pristine 2D insulator, the 1D Hamiltonian along XM $\mathcal{H}_{XM}(k_y)$ has a Berry phase:

$$\gamma_{XM} = \frac{\pi}{2}\left[1 - \xi(X)\xi(M)\right]. \tag{108}$$

Because all 1D Hamiltonians are Wannierizable [152, 246, 250, 251], then we can immediately relate γ to the locations of (hybrid) Wannier centers in the 2D insulator. Specifically, in the hybrid Wannier [250, 251] basis of y and k_x, γ_{XM} mod $2\pi = \pi$ indicates that, for $k_x = \pi$, the number of hybrid Wannier centers (or Kramers pairs of Wannier centers under an additional $\mathcal{T}$ symmetry) at $y = 1/2$ (*i.e.* SSH Wyckoff position $1b$ [152]) and the number at $y = 0$ (SSH Wyckoff position $1a$) differ by an *odd* integer.

To complete our calculation of n_{XM}, we must also obtain the Berry phase γ^A_{XM} of the valence atomic orbitals in the y and $k_x = \pi$ hybrid Wannier basis, which we will shortly define below. It is crucial to note that γ^A_{XM} cannot be obtained from any momentum-space calculation, but must instead be determined *a priori* through knowledge of the implementation of the tight-binding Hamiltonian. Specifically, as described by Topological Quantum Chemistry (TQC) [135, 137, 152–155, 243], position-space atomic orbitals induce EBRs, which are then subduced onto k-points to form momentum-space bands. However, because the overlap between atomic orbitals can drive band inversions, the actual set of occupied bands may not necessarily exhibit the same symmetry eigenvalues and Berry phases as the "uninverted" atomic insulator with the same atomic orbitals. Therefore, in order to determine γ^A_{XM}, one must have knowledge of which bands are inverted relative to the unobstructed atomic limit of the valence atomic orbitals. Though this requirement may appear unrealistically stringent, in practice, most topological insulating materials with clean Fermi surfaces are narrow-gap semiconductors for which it is chemically straightforward to determine which bands from which atomic orbitals have become inverted [131, 132, 196–198, 264, 265]. For example, in SN 6 A, we will demonstrate that PbTe monolayers differ from unobstructed atomic limits by band inversion at just two (symmetry-equivalent) TRIM points. Returning to the example in this section, we determine γ^A_{XM} by projecting the positions of the valence atomic orbitals onto the y-axis, obtaining a set of y-coordinates y_a for each valence orbital a. From this, the Berry phase of the atomic orbitals is given by:

$$\gamma^A_{XM} = \left(2\pi \sum_a y_a\right) \bmod 2\pi, \tag{109}$$

where the sum is restricted to atoms within a single unit cell. Finally, we obtain n_{XM} through SEqs. (108) and (109):

$$\pi n_{XM} = \left(\gamma_{XM} - \gamma^A_{XM}\right) \bmod 2\pi. \tag{110}$$

This definition follows in perfect analogy from the definition of 1D polarization in terms of electronic and atomic Berry phases [266]. Substituting SEq. (110) into SEq. (106) and determining n_{YM} through the exchange $x \leftrightarrow y$ in SEqs. (108) through (110) and the surrounding text, we complete the definition of the weak polarization invariant $\boldsymbol{M}^{\mathrm{SSH}}_{\nu}$.

B. The 3D Weak Fragile Invariant $\boldsymbol{M}^{\mathrm{F}}_{\nu}$

In this section, we will derive the new 3D weak invariant $\boldsymbol{M}^{\mathrm{F}}_{\nu}$ for $\mathcal{I}$- (and optionally spinful $\mathcal{T}$-) symmetric insulators to identify which of the BZ boundary planes host 2D Hamiltonians with anomalous corner charges. We have previously shown in SN 2 that these corner charges will manifest as dislocation HEND states if the respective BZ plane satisfies $\boldsymbol{k} \cdot \boldsymbol{B}$ mod $2\pi = \pi$, where $\boldsymbol{B}$ is the Burgers vector of the dislocation. Because the simplest models of $\mathcal{I}$-symmetric

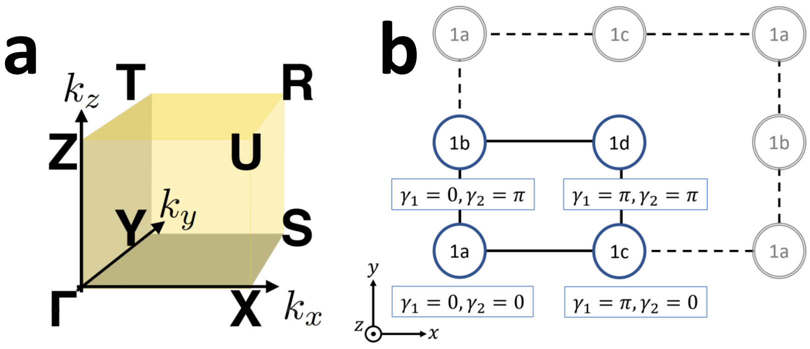

Supplementary Figure 11. **Brillouin zone for orthorhombic 3D crystals and Berry phases in 2D.** ***a*** The 3D BZ of a crystal with orthorhombic lattice vectors [223, 238, 239]. ***b*** The 2D Wyckoff positions [238, 239] of a layer group [25, 70, 223, 227, 228, 237, 262] with only rectangular lattice translation $T_{x,y}$ and $\mathcal{I}$ symmetries (and optionally an additional spinful $\mathcal{T}$ symmetry). As shown in SRef. 33, a single Wannier orbital placed at one of the four $\mathcal{I}$ centers (maximal Wyckoff positions [152]) exhibits the x-directed Berry phases γ_1 and y-directed nested Berry phases γ_2 shown in ***b***. If $\mathcal{T}$ symmetry is additionally present, then all Wannier orbitals necessarily appear in Kramers pairs that exhibit a total $\gamma_{1,2} \bmod 2\pi = 0$ at all of the maximal Wyckoff positions. Nevertheless, as we will show in SN 3 B 3, by expanding the concept of *time-reversal polarization* [6, 258] to nested Wilson loops, we can define a partial *nested* γ_2 that distinguishes the locations of Kramers pairs of Wannier orbitals.

2D insulators with fractionally-charged corner states have fragile topological bands (specifically four-band models of $\mathcal{I}$-symmetric FTIs, see SRefs. 33 and 100), then we will in this work associate $\boldsymbol{M}_\nu^{\mathrm{F}}$ with fragile topology. However and crucially, because FTIs can be trivialized into OALs through the addition of appropriately chosen trivial bands [33, 70, 100, 107–110, 134–149], and because there also exist simple models of 2D $\mathcal{I}$-symmetric OALs with anomalous corner charges [176], then we note for completeness that a nontrivial $\boldsymbol{M}_\nu^{\mathrm{F}}$ does not necessarily imply that the valence manifold taken as a whole exhibits fragile topology. Like the established index $\boldsymbol{M}_\nu$ for weak TIs [7–9, 211], $\boldsymbol{M}_\nu^{\mathrm{F}}$ has three components:

$$\boldsymbol{M}_\nu^{\mathrm{F}} = \pi\left[\nu_x^{\mathrm{F}}\hat{x} + \nu_y^{\mathrm{F}}\hat{y} + \nu_z^{\mathrm{F}}\hat{z}\right]. \tag{111}$$

In this section, we will explicitly derive ν_z^{F}, with the understanding that $\nu_{x,y}^{\mathrm{F}}$ can similarly be obtained through the cyclic exchange $x \to y \to z \to x$.

To begin, we will first consider in SN 3 B 1 a 3D insulator with orthorhombic lattice vectors and $\mathcal{I}$ symmetry. Unlike previously in SN 3 A, we will start by explicitly requiring that spinful $\mathcal{T}$ symmetry is broken. Later, in SN 3 B 2, we will extend our arguments to $\mathcal{I}$- and $\mathcal{T}$-symmetric insulators. Finally, in SN 3 B 3, we will use nested Wilson loops [30, 31, 33, 70, 95, 100, 138, 149] to develop a new formulation of nested time-reversal polarization [6, 258] to diagnose 2D $\mathcal{T}$-symmetric OALs that display anomalous corner modes despite exhibiting trivial nested Berry phases (when taken modulo 2π).

1. $\mathcal{T}$-Broken EBR Formulation of $\boldsymbol{M}_\nu^{\mathrm{F}}$

In this section, we will first establish a definition of ν_z^{F} for $\mathcal{I}$-symmetric, $\mathcal{T}$-broken 3D insulators, which, through SEq. (111) and the cyclic exchange $x \to y \to z \to x$, is sufficient to also define the weak corner-mode invariant $\boldsymbol{M}_\nu^{\mathrm{F}}$. As shown in SRefs. 33, 100, and 176, an $\mathcal{I}$-symmetric, $\mathcal{T}$-broken 2D insulator will exhibit (filling) anomalous corner charges if its valence bands B are formed from an odd total number of FTIs and OALs with anomalous corner modes. First, we will use the results of SRefs. 70 and 176 to show that a single occupied band can be converted from an unobstructed atomic limit without corner modes to an OAL with $\mathcal{I}$-anomalous corner modes through band inversions at two distinct TRIM points. Then, using the results of SRefs. 33 and 100, we will show that there are eight possible FTIs with two occupied bands and $\mathcal{I}$-anomalous corner modes, which are each driven by double band inversion [33, 100, 144] about the same TRIM point. Taken together, these OALs and FTIs, along with (stable) Chern insulators, can be used to define ν_z^{F} for a set of valence bands that differs from an unobstructed atomic limit through a known or controllable sequence of band inversions. We note that the conclusions derived here no longer generically apply when reflection and high-fold rotation symmetries are also enforced, because higher symmetries can protect a more diverse set of stable and fragile topological phases than $\mathcal{I}$ by itself [14, 15, 18, 30, 32, 33, 70, 95, 98–100, 141–143, 145–151, 176, 229–234, 267]. However, the arguments presented in this section can be adapted to also predict

electronic defect states with higher symmetry requirements, albeit with some effort to carefully treat the position-space action of more complicated (potentially magnetic [155]) crystal symmetries. We leave this generalization for future works.

To determine ν_z^{F}, we begin by restricting consideration to the $k_z = \pi$ plane of a 3D BZ (SFig. 11 ***a***). The 2D Hamiltonian of the $k_z = \pi$ plane $\mathcal{H}_{k_z=\pi}(k_x, k_y)$ contains four TRIM points at which bands are generically singly degenerate and can be labeled according to their parity eigenvalues ± 1. First and foremost, in order for $\mathcal{H}_{k_z=\pi}(k_x, k_y)$ to exhibit only corner modes, its occupied bands B must not carry a net Chern number C_B. For 2D insulators with only $\mathcal{I}$ symmetry, C_B mod 2 can be determined through the parity eigenvalues of the occupied bands [267]. Specifically, for $\mathcal{H}_{k_z=\pi}(k_x, k_y)$:

$$C_B \bmod 2 = \frac{1}{2}\left[1 - \xi(Z)\xi(U)\xi(T)\xi(R)\right], \quad (112)$$

where $\xi(\boldsymbol{k}_D)$ is the product of the parity eigenvalues of the occupied bands at the TRIM point $\boldsymbol{k}_D$.

EBRs of Magnetic Layer Group $p\bar{1}$										
Orbitals	Γ	X	Y	M		Orbitals	Γ	X	Y	M
$(s)_{1a}$	$+$	$+$	$+$	$+$		$(p)_{1a}$	$-$	$-$	$-$	$-$
$(s)_{1b}$	$+$	$+$	$-$	$-$		$(p)_{1b}$	$-$	$-$	$+$	$+$
$(s)_{1c}$	$+$	$-$	$+$	$-$		$(p)_{1c}$	$-$	$+$	$-$	$+$
$(s)_{1d}$	$+$	$-$	$-$	$+$		$(p)_{1d}$	$-$	$+$	$+$	$-$

Supplementary Table 1. Elementary band representations in a rectangular 2D system with inversion symmetry. Using BANDREP [152–154, 243] on the Bilbao Crystallographic Server (BCS), we obtain the elementary band representations (EBRs) [135, 137, 152–155, 241–243] of magnetic layer group [25, 70, 227, 228, 237, 262] $p\bar{1}$ (*i.e.*, the 2D symmetry group generated by rectangular lattice translations $T_{x,y}$ and 3D inversion $\mathcal{I}$). We employ a shorthand notation in which $(\rho)_q$ indicates the EBR induced from $\rho = s, p$ orbitals placed at the maximal Wyckoff position ($\mathcal{I}$ center) q in SFig. 11 ***b***. At each TRIM point in the 2D BZ of $p\bar{1}$ (SFig. 10 ***b***), a one-dimensional irreducible representation with a positive (negative) parity eigenvalue is subduced that is equivalent to the irreducible representation $\bar{A}_g$ ($\bar{A}_u$) of magnetic point group $\bar{1}$ [235–239], which we respectively denote with the shorthand $+$ $(-)$.

Valence Bands	Γ	X	Y	M
B	$+$	$+$	$+$	$+$
B'	$-$	$+$	$+$	$+$
B''	$-$	$-$	$+$	$+$
B'''	$-$	$-$	$-$	$+$
B''''	$-$	$-$	$-$	$-$

Supplementary Table 2. The parity ($\mathcal{I}$) eigenvalues of the occupied bands B–B'''' discussed in the text following SEq. (113).

If SEq. (112) indicates that C_B mod $2 = 0$, then B necessarily exhibits the same set of symmetry eigenvalues as a sum and difference of EBRs (*i.e.*, bands induced from Wannier orbitals at the maximal Wyckoff positions [152]). In this case, we refer to B as *irreducible-representation-equivalent* ($\overset{I}{\equiv}$ in equations, irrep-equivalent in text) [70, 135, 137, 141–143] to a linear combination of EBRs. In this section, the 2D Hamiltonian $\mathcal{H}_{k_z=\pi}(k_x, k_y)$ is invariant under magnetic layer group [25, 70, 227, 228, 237, 262] $p\bar{1}$ (*i.e.*, the 2D group generated by rectangular lattice translations $T_{x,y}$ and 3D inversion $\mathcal{I}$, for which $\mathcal{I}^2 = +1$ whether or not SOC is relevant). The complete set of EBRs of $p\bar{1}$ can be induced from s and p orbitals placed at the four maximal Wyckoff positions, *i.e.*, the four $\mathcal{I}$ centers of the 2D unit cell (SFig. 11 ***b***). We obtain the irreducible representations of the EBRs at each high-symmetry point of the 2D BZ of $p\bar{1}$ (SFig. 10 ***b***) by using the BANDREP tool on the Bilbao Crystallographic Server [152–155, 243] (BCS) for space group (SG) 2 $P\bar{1}1'$ taken without $\mathcal{T}$ symmetry, which is isomorphic to magnetic layer group $p\bar{1}$ modulo lattice translations [223, 237]. For convenience, the resulting EBRs are reproduced in Supplementary Table 1. Because $\mathcal{H}_{k_z=\pi}(k_x, k_y)$ is (quasi-) 2D, then we will convert its TRIM points into the 2D notation in SFig. 10, both to facilitate the generalization of this calculation for the later determination of $\nu_{x,y}^{\mathrm{F}}$, and to draw connection with previous works [33, 70, 100, 176, 227]. For ν_z^{F}, the conversion from 3D, zone-edge TRIM points (SFig. 11 ***a***) to 2D, layer group TRIM points (SFig. 10 ***b*** and Supplementary Table 1) is given by:

$$Z \to \Gamma,\ U \to X,\ T \to Y,\ R \to M. \quad (113)$$

As a first step towards formulating ν_z^{F}, we will consider the simplest case where B consists of a single occupied band. Specifically, we begin with a band structure for which the valence and conduction bands are respectively induced from spinful s and p orbitals placed at the same Wyckoff position. In this case, the band structure at each bulk TRIM point exhibits a gap between states with opposite parity eigenvalues, and B characterizes an unobstructed atomic limit [the first row in Supplementary Table 2 shows B induced from the $1a$ position of magnetic layer group $p\bar{1}$ (Supplementary Table 1)]. If we then invert bands at one of the TRIM points, a gap will necessarily open, because $\mathcal{I}$ symmetry cannot by itself protect crossings anywhere in a 2D BZ [19, 63, 227] (though in a 3D BZ, Weyl points are allowed to form at generic crystal momenta [61]). The band inversion results in a new insulator with an occupied band B' (the second row in Supplementary Table 2 shows B' for a band inversion at Γ). By the parity criterion in SEq. (112), B' necessarily exhibits a Chern number $|C_{B'}| = 1$. Crucially, if we again invert bands, but this time at a different TRIM point than previously, we realize a valence manifold B'' (the third row in Supplementary Table 2 shows B'', taking the second band inversion to occur at X) where symmetry-indicated Chern number of B'' is *trivial*:

$$C_{B''} \text{ mod } 2 = 0. \tag{114}$$

Furthermore, comparing B'' to Supplementary Table 1, we observe that B'' is also irrep-equivalent to an EBR, but crucially *not* the same EBR as the original valence band B. As rigorously shown in SRefs. 33, 70, 152, and 176, if $C_{B''} = 0$, which must be determined through an explicit Wilson-loop calculation, then, because B'' and the corresponding conduction band represent the shift of only two Wannier orbitals driven by one or more band inversions, B'' is necessarily an OAL. Furthermore, as shown in SRefs. 33 and 176, all OALs in $p\bar{1}$ with one occupied band necessarily exhibit filling-anomalous corner modes.

We continue by further inverting bands at a third TRIM point, yielding an occupied band B''' (the fourth row in Supplementary Table 2 shows B''', taking the third band inversion to occur at Y). Once, again, as with B', SEq. (112) implies that $C_{B'''}$ mod $2 = 1$. Finally, if we invert bands at the last TRIM point, yielding an occupied band B'''' (the fifth row in Supplementary Table 2 shows B'''', taking the final band inversion to occur at M), then again $C_{B''''}$ mod $2 = 0$ and B'''' is irrep-equivalent to an EBR. However, all of the parity eigenvalues of B'''' are simply given by those of B multiplied by -1. By comparing B'''' to Supplementary Table 1, we discover that, while B'''' is irrep-equivalent to a different EBR than B, B'''' is still irrep-equivalent to an EBR induced from the same Wyckoff position as B. In fact, this process of inverting bands at all four TRIM points, through our original construction, has merely exchanged the valence and conduction bands, which were indeed induced from s and p orbitals at the same Wyckoff position ($1a$ in Supplementary Table 2). Therefore, B'''' does not exhibit anomalous corner modes, because it is not an OAL. We have thus shown that if a generic valence manifold B'' consists of a single band and differs from an unobstructed atomic limit by two band inversions at different TRIM points between bands with opposite parity eigenvalues, then B'' will necessarily be irrep-equivalent to an OAL with anomalous corner modes.

Bands	Γ	X	Y	M
$\tilde{B}$	$+,+$	$+,+$	$+,+$	$+,+$
$\tilde{B}'$	$-,-$	$+,+$	$+,+$	$+,+$
$\tilde{\tilde{B}}'$	$+,+$	$-,-$	$-,-$	$-,-$
$\tilde{B}''$	$-,-$	$-,-$	$+,+$	$+,+$

Supplementary Table 3. The parity ($\mathcal{I}$) eigenvalues of the bands $\tilde{B}$–$\tilde{B}''$ discussed in the text surrounding SEq. (115).

Next, we consider the more complicated case in which the valence manifold $\tilde{B}$ consists of two occupied bands [the first row in Supplementary Table 3 shows $\tilde{B}$ induced from two s orbitals at the $1a$ position of magnetic layer group $p\bar{1}$ (see Supplementary Table 1)]. For simplicity, we consider $\tilde{B}$ to be induced from two spinful s orbitals at the same Wyckoff position (in the example shown in Supplementary Table 3, $\tilde{B}'$ is induced from the $1a$ position), and we consider the conduction bands to be induced from two spinful p orbitals at the same Wyckoff position as the s orbitals. Hence, the two valence bands exhibit the same parity eigenvalues, and the two conduction bands both exhibit the same parity eigenvalues, opposite to those of the valence bands. In this case, all band inversions at half filling necessarily occur between bands with opposite parity eigenvalues. We next consider creating a set of occupied bands $\tilde{B}'$ through *double band inversion* [100, 102, 144]– a process by which two valence bands are exchanged with two conduction bands at the same TRIM point (the second row in Supplementary Table 3 shows $\tilde{B}'$, taking the double band inversion to occur at Γ). Taking the valence (conduction) bands to be induced from pairs of spinful s (p) orbitals at the $1a$ position and the double band inversion to occur about the Γ point (SFig. 10 ***b***):

$$\tilde{B}' \stackrel{\mathcal{I}}{\equiv} (p)_{1b} \oplus (p)_{1c} \oplus (p)_{1d} \ominus (p)_{1a}. \tag{115}$$

The $\ominus$ in SEq. (115) indicates that $\tilde{B}'$ exhibits fragile topology [70, 135, 137, 141–143]. As extensively shown in SRefs. 33, 100, and 176, $\tilde{B}'$ specifically characterizes an $\mathcal{I}$-symmetric FTI with filling-anomalous corner modes.

Next we will generalize SEq. (115) and generate all of the $\mathcal{I}$-symmetric FTIs with two occupied bands and anomalous corner modes. First, we recognize that the previous double band inversion has created a set of two conduction bands $\tilde{\tilde{B}}'$ that are *also* fragile [33, 100] (the third row in Supplementary Table 3 shows $\tilde{\tilde{B}}'$, taking the double band inversion to occur at Γ). Here, because all of the parity eigenvalues of $\tilde{\tilde{B}}'$ are the opposite of those of $\tilde{B}'$, we determine that $\tilde{\tilde{B}}'$ is also fragile and exhibits anomalous corner modes by setting $p \to s$ in SEq. (115) (Supplementary Table 1). Next, because all of the maximal Wyckoff positions of $p\bar{1}$ have the same site-symmetry group $(\bar{1})$ [152], and because there are no other symmetries, then, in an infinite crystal, any two Wyckoff positions can be exchanged by a change of origin and coordinate definitions without loss of generality. Specifically, this set of exchanges of origin and coordinate definitions in an infinite crystal should have no effect on the corner spectrum of the same crystal as long as the corner spectrum is calculated in a finite-sized geometry that still preserves $\mathcal{I}$ [33, 100]. Hence, the corner spectrum must be invariant with respect to the exchange of any two Wyckoff positions in SEq. (115). In practice, exchanging $1b$, $1c$, or $1d$ with $1a$ in SEq. (115) simply generates the occupied bands of an FTI driven by double band inversion about a TRIM point other than Γ. Therefore, to generate the eight possible $\mathcal{I}$-symmetric FTIs with two occupied bands and filling-anomalous corner modes, we can exchange $p \leftrightarrow s$ and $1a \leftrightarrow 1b$, $1c$, or $1d$ in SEq. (115).

Next, we will exclude insulators generated from double band inversion at the same TRIM point between pairs of bands with the same symmetry eigenvalues. For example, if both the valence and conduction bands initially exhibit $\{+,-\}$ parity eigenvalues, then the valence and conduction bands will in this case *also* exhibit $\{+,-\}$ parity eigenvalues after double band inversion. Designating the initial (final) valence bands as B (B'), double band inversion between sets of bands with $\{+,-\}$ eigenvalues, if bands do not simply avoid crossing, necessarily results in a situation for which $B \overset{I}{\equiv} B'$. Therefore, double band inversion between bands with the same set of parity eigenvalues necessarily does not drive a symmetry-indicated phase transition. We leave the possibility of non-symmetry-indicated band-inversion transitions for future works [156].

Finally, we consider the process of further double band inverting from one of the four FTIs generated by the cyclic exchange of orbitals and Wyckoff positions in SEq. (115). We take the second double band inversion to occur at a different TRIM point than the previous transition that drove $\tilde{B}$ to $\tilde{B}'$, and designate the occupied bands of the insulator with four total band inversions (occurring in pairs at two different TRIM points) $\tilde{B}''$ (the fourth row in Supplementary Table 3 shows $\tilde{B}''$, taking the second double band inversion to occur at X). Because the $k \cdot p$ Hamiltonian about the TRIM point of the second double band inversion (SRefs. 70 and 100 and SN 2 A 1) is, by construction, unitarily related to the $k \cdot p$ Hamiltonian about the TRIM point of the first double band inversion, then $\tilde{B}''$ necessarily exhibits $2L^{\mathrm{FTI}}_{z,n} = 2 + 4n$ (SEq. 37) *more* corner modes than $\tilde{B}'$. Therefore, $\tilde{B}''$ must exhibit a *non-anomalous* number of corner modes $2L^{NI}_z$, where:

$$L^{NI}_z = 2n',\ n' \in \{\mathbb{Z}^+, 0\}. \tag{116}$$

Hence, beginning with an unobstructed atomic limit, only odd numbers of double band inversions between sets of bands with opposite parity eigenvalues at the same TRIM point can result in insulators with $\mathcal{I}$-anomalous corner modes.

Combining the discussions in this section, we have shown for unobstructed atomic limits with one or two occupied bands and trivial corner spectra that $2L^{\mathrm{FTI}}_{z,n} = 2 + 4n$ (SEq. 37) band inversions between bands with opposite parity eigenvalues necessarily results in an insulator with anomalous corner modes. Therefore, for a 2D insulator in $p\bar{1}$, we can define a $\mathbb{Z}_4$-valued quantity n_4 that counts the number of parity-eigenvalue-exchanging band inversions required to transition between the occupied bands and a known unobstructed atomic limit without corner modes, corresponding to uninverted bands, modulo 4. When n_4 is odd, the occupied bands necessarily exhibit an odd Chern number [SEq. (112)]. Finally, when $n_4 = 2$, the 2D system is either a non-symmetry-indicated Chern insulator [267] with an even Chern number, or an insulator with gapped edges and anomalous corner states.

The $\mathbb{Z}_4$ nature of n_4 also provides some insight on the recent identification of $\mathbb{Z}_4$-valued symmetry-based indicators for centrosymmetric magnetic TIs and semimetals [155, 174, 175]. To see this, consider a 3D insulator that is invariant under $\mathcal{I}$ and orthorhombic lattice translations $T_{x,y,z}$ (magnetic SG [155, 174, 175, 223, 237, 268] 2.4 $P\bar{1}$). The Hamiltonians of both the $k_z = 0, \pi$ planes are invariant under magnetic layer group $p\bar{1}$. We fix $n_4 = 0$ for the Hamiltonian $\mathcal{H}_{k_z=\pi}(k_x, k_y)$ [$n_4(\pi)$] and allow n_4 of $\mathcal{H}_{k_z=0}(k_x, k_y)$ [$n_4(0)$] to vary. When $n_4(0) \neq n_4(\pi)$, the 3D bulk can be reexpressed as a k_z-indexed periodic tuning cycle between two topologically distinct 2D insulators. For odd $n_4(0)$, the Chern numbers of the occupied bands at $k_z = 0$ and $k_z = \pi$ differ by an odd number [SEq. (112)], and therefore the 3D system is necessarily a Weyl semimetal [269]. When $n_4(0) = 2$, the bulk represents a gapless pumping cycle between an insulator with anomalous corner charges and one with no filling anomaly, and is thus a magnetic axion insulator (AXI) characterized by the axion (theta) angle $\theta = \pi$ [33, 107–110]. Crucially, in the previous AXI, if we exchange $k_z = 0, \pi$, then $n_4(\pi) = 2$, which directly implies that $\nu^{\mathrm{F}}_z = 1$.

Hence, ν_z^{F} (as well as $\nu_{x,y}^{\mathrm{F}}$) can simply be calculated in a 3D $\mathcal{I}$-symmetric, $\mathcal{T}$-broken insulator by counting the number of parity-eigenvalue-exchanging band inversions modulo 4 in the $k_z = \pi$ ($k_{x,y} = \pi$) plane, relative to an unobstructed atomic limit. This is equivalent to calculating n_4 in the $k_i = \pi$ plane [$n_4(k_i = \pi)$]. If $n_4(k_i = \pi)$ is odd, then ν_i^{F} is undefined, and if $n_4(k_i = \pi)$ is even, then:

$$\nu_i^{\mathrm{F}} = \frac{n_4(k_i = \pi)}{2} \bmod 2. \tag{117}$$

We have thus completed our definition of $\boldsymbol{M}_\nu^{\mathrm{F}}$ for centrosymmetric, $\mathcal{T}$-broken 3D insulators [SEq. (111)]. While the discovery of magnetic topological materials is a fruitful field of ongoing study [147, 148, 155, 174, 175, 268, 270–274], we will only focus in this work on applying the $\mathcal{T}$-broken formulation of $\boldsymbol{M}_\nu^{\mathrm{F}}$ to simple tight-binding models – which are provided in SN 4, 5 – as opposed to real-material magnetic topological (crystalline) insulators. However, in SN 3 B 2, we will shortly formulate a $\mathcal{T}$-symmetric extension of $\boldsymbol{M}_\nu^{\mathrm{F}}$, which can be used to predict spin-charge separated HEND states in nonmagnetic 3D insulators.

Lastly, because Wilson loop indices for filling anomalies in $\mathcal{I}$-symmetric, $\mathcal{T}$-broken 2D insulators were previously derived in SRef. 33, we will not reintroduce them in this work. We instead simply note that the nested Berry phase γ_2, as formulated in SRefs. 33 and 100, along with the Chern number of the occupied bands, provides an alternative formulation of n_4. In SN 3 B 3, we will build upon the results of SRefs. 33 and 100 to develop analogous nested Wilson indicators for $\mathcal{I}$- and spinful $\mathcal{T}$-symmetric 2D insulators with anomalous corner states.

2. $\mathcal{T}$-Symmetric EBR Formulation of $\boldsymbol{M}_\nu^{\mathrm{F}}$

In this section, we will adapt the definition of ν_z^{F} previously developed in SN 3 B 1 to $\mathcal{I}$- and $\mathcal{T}$-symmetric 3D insulators, which, through SEq. (111) and the cyclic exchange $x \to y \to z \to x$, is sufficient to also define the $\mathcal{T}$-symmetric weak corner-mode invariant $\boldsymbol{M}_\nu^{\mathrm{F}}$. Because parity eigenvalues are real, then the addition of spinful $\mathcal{T}$ symmetry to the previous centrosymmetric magnetic insulators in SN 3 B 1 simply doubles all of states at all crystal momenta through the combined symmetry [227] $\mathcal{I} \times \mathcal{T}$ (if we maintain the previous simplifying assumption that high-fold rotation symmetries are absent).

To begin, rather than considering a 3D insulator in magnetic SG 2.4 $P\bar{1}$ [155, 174, 175, 223, 237, 268], we consider one in its $\mathcal{T}$-symmetric supergroup SG 2 $P\bar{1}1'$ [223, 237]. Taking the lattice translations $T_{x,y,z}$ to be orthogonal without loss of generality, the Hamiltonians of the $k_i = 0, \pi$ planes of the 3D BZ (SFig. 11 ***a***) are invariant under the $\mathcal{T}$-symmetric layer group $p\bar{1}1'$ [25, 227], whose generating elements are simply rectangular lattice translation, $\mathcal{I}$, and $\mathcal{T}$. Layer group $p\bar{1}1'$, like its magnetic subgroup $p\bar{1}$, also has four maximal Wyckoff positions that coincide with the centers of $\mathcal{I}$ symmetry (SFig. 11 ***b***). Therefore, each of the four maximal Wyckoff positions has the $\mathcal{T}$-symmetric site-symmetry group $\bar{1}1'$ [235, 243], which has two, two-dimensional irreducible corepresentations:

$$\bar{\rho}^+ \equiv \bar{A}_g\bar{A}_g,\ \ \bar{\rho}^- \equiv \bar{A}_u\bar{A}_u, \tag{118}$$

that characterize Kramers pairs of states with positive and negative parity eigenvalues, respectively.

Valence Bands	Γ	X	Y	M
B	+	+	+	+
B'	−	+	+	+
B''	−	−	+	+

Supplementary Table 4. The parity ($\mathcal{I}$) eigenvalues of the occupied bands B–B'' discussed in the text following SEq. (119). Each + and − symbol represents a Kramers pair of states with the same parity eigenvalues.

Next, we will revisit all of the band-inversion arguments previously presented in SN 3 B 1. Because all states in momentum space and localized states in real space are twofold degenerate due to spinful $\mathcal{I} \times \mathcal{T}$ symmetry, then we will in this section consider single band inversion to occur between *Kramers pairs* of states, and double band inversion to occur between pairs of Kramers pairs (eight total) states, representing a doubling of the previous state counting arguments in SN 3 B 1. We then recognize that, because $\mathcal{I} \times \mathcal{T}$ symmetry cannot protect semimetallic crossings in a 2D or 3D crystal for which $(\mathcal{I} \times \mathcal{T})^2 = -1$ [19, 63, 227, 275], then all inversions between between bands with opposite parity eigenvalues still necessarily result in insulators, as they did for the 2D magnetic insulators in SN 3 B 1. Beginning with an unobstructed atomic limit, we next recognize that an odd number of band inversions with $\mathcal{I}$ and spinful $\mathcal{T}$ symmetry necessarily yields the occupied bands of a 2D TI, rather than a symmetry-indicated

Chern insulator with an odd Chern number [see the text surrounding SEq. (112) and SRef. 267], as it did previously in SN 3 B 1. Specifically, in the $\mathcal{I}$- and $\mathcal{T}$-symmetric 3D insulator discussed in this section, the 2D Hamiltonian of the $k_z = \pi$ plane $\mathcal{H}_{k_z=\pi}(k_x, k_y)$ exhibits a topology that can be partially diagnosed through the Fu-Kane index [7, 8]:

$$\nu_x = \frac{1}{2}\left[1 - \xi(Z)\xi(U)\xi(T)\xi(R)\right], \tag{119}$$

where $\xi(\boldsymbol{k}_D)$ is the product of the parity eigenvalues per Kramers pair of the occupied bands at the TRIM point $\boldsymbol{k}_D$, and where $\nu_x = 0$ ($\nu_x = 1$) indicates a trivial insulator (2D TI). Therefore, if we begin with a 2D unobstructed atomic limit with the occupied bands B (the first row in Supplementary Table 4 shows B induced from the $1a$ position of the $\mathcal{T}$-symmetric layer group $p\bar{1}1'$), a single band inversion between Kramers pairs of states with opposite parity eigenvalues results in an insulator whose occupied bands B' necessarily characterize a 2D TI (the second row in Supplementary Table 4 shows B', taking the band inversion to occur at Γ).

From B', we then consider a second band inversion, either at the same TRIM point or a different one, resulting in a set of occupied bands B'' (the third row in Supplementary Table 4 shows B'', taking the second band inversion to occur at X). Through SEq. (119), we recognize that B'', absent additional mirror or rotation symmetries [14, 15, 18, 30, 32, 33, 70, 95, 98–100, 141–143, 145–151, 176, 229–234, 267], is necessarily irrep-equivalent to a sum and difference of EBRs. For $p\bar{1}1'$, we can use all of the previous EBRs of $p\bar{1}$ (Supplementary Table 1) by recognizing that both the initial spinful atomic orbitals and the subduced momentum-space degeneracies at the TRIM points have doubled without changing any of the parity eigenvalues [152–155, 243]. We also recognize from the discussion in SRef. 100 of weak-SOC (spinless), $\mathcal{I}$-symmetric FTIs with anomalous corner modes that the superposition of two time-reversed 2D insulators with $\mathcal{I}$-anomalous corner modes remains filling-anomalous under the preservation of $\mathcal{T}$. Rather, the only qualitative differences between the $\mathcal{T}$-symmetric and $\mathcal{T}$-broken FTIs are a doubling of the state counting and a revised interpretation of the corner soliton spin and charge. Specifically, in the $\mathcal{I}$- and $\mathcal{T}$-symmetric case, the corner modes manifest in Kramers pairs that exhibit the characteristic spin-charge separation of the solitons of the spinful SSH chain, rather than the fractional $e/2$ charges of the singly-degenerate solitons of the spinless (or $\mathcal{T}$-broken) SSH chain [33, 70, 212–218]. Hence, B'', which resulted from two parity-eigenvalue-exchanging band inversions in an unobstructed atomic limit, necessarily characterizes a (possibly fragile) 2D insulator with anomalous corner modes.

Now that we have established that two band inversions necessarily drive an $\mathcal{I}$- and $\mathcal{T}$-symmetric unobstructed atomic limit into a 2D insulator with anomalous corner modes, all of the previous generalizations from SN 3 B 1 to arbitrarily large numbers of band inversions also apply, without further modification. Combining all of the analysis in this section and the previous discussion in SN 3 B 1, for a 2D insulator in $p\bar{1}1'$, we can again define a $\mathbb{Z}_4$-valued quantity n_4 that counts the number of parity-eigenvalue-exchanging band inversions required to transition between the occupied bands and a known unobstructed atomic limit, corresponding to uninverted bands, modulo 4. When n_4 is odd, the occupied bands necessarily characterize a 2D TI [SEq. (119)]. Finally, when $n_4 = 2$, the 2D system is an insulator with gapped edges and anomalous *Kramers pairs* of spin-charge-separated corner states.

The $\mathbb{Z}_4$ nature of n_4 in the $\mathcal{T}$-symmetric case also provides insight on the recent identification of a strong $\mathbb{Z}_4$ parity index for $\mathcal{I}$- and $\mathcal{T}$-symmetric 3D TIs and HOTIs [32, 100, 103–105]. To see this, consider a 3D insulator that is invariant under $\mathcal{I}$, $\mathcal{T}$, and orthorhombic lattice translations $T_{x,y,z}$ (SG 2 $P\bar{1}1'$). The Hamiltonians of both the $k_z = 0, \pi$ planes are invariant under the $\mathcal{I}$- and $\mathcal{T}$-symmetric layer group $p\bar{1}1'$. We again fix $n_4 = 0$ for the Hamiltonian $\mathcal{H}_{k_z=\pi}(k_x, k_y)$ [$n_4(\pi)$] and allow n_4 of $\mathcal{H}_{k_z=0}(k_x, k_y)$ [$n_4(0)$] to vary. When $n_4(0) \neq n_4(\pi)$, the 3D bulk can be reexpressed as a k_z-indexed periodic tuning cycle between two topologically distinct 2D insulators, analogous to the magnetic insulators previously discussed in the text surrounding SEq. (117). For odd $n_4(0)$, the 2D TI indices of the occupied bands at $k_z = 0$ and $k_z = \pi$ are distinct [SEq. (119)]. Unlike previously in SN 3 B 1, this does not imply a Weyl semimetal, because the combined symmetry $\mathcal{I} \times \mathcal{T}$ prohibits the stabilization of conventional Weyl fermions (or any other form of chiral fermion) at generic crystal momenta [275]. Instead, the 3D system represents a $\mathcal{T}$-symmetric pumping cycle between a 2D TI and a trivial insulator, which, in turn, characterizes a 3D TI [7, 8, 13]. Finally, when $n_4(0) = 2$, the bulk represents a gapped, helical pumping cycle between an insulator with a filling anomaly and a trivial insulator without corner modes, and is thus an $\mathcal{I}$- and $\mathcal{T}$-symmetric HOTI [33, 100]. Crucially, for the aforementioned HOTI, if we exchange $k_z = 0, \pi$, then $n_4(\pi) = 2$, which directly implies that $\nu_z^{\mathrm{F}} = 1$.

Therefore, ν_z^{F} (as well as $\nu_{x,y}^{\mathrm{F}}$) can again simply be calculated in an $\mathcal{I}$- and $\mathcal{T}$-symmetric 3D insulator by counting the number of parity-eigenvalue-exchanging band inversions modulo 4 in the $k_z = \pi$ ($k_{x,y} = \pi$) plane, relative to an unobstructed atomic limit. This is equivalent to calculating n_4 in the $k_i = \pi$ plane [$n_4(k_i = \pi)$]. If $n_4(k_i = \pi)$ is odd, then ν_i^{F} is undefined, but the conventional weak index ν_i is nontrivial [7–9, 211]. And if $n_4(k_i = \pi)$ is even, then, if $n_4(k_i = \pi) = 2L_{z,n}^{\mathrm{FTI}} \bmod 4 = 2$ [SEq. (37)], ν_i^{F} is necessarily nontrivial. This can be summarized as:

$$\nu_i = n_4(k_i = \pi) \bmod 2, \tag{120}$$

and:

$$\nu_i^{\mathrm{F}} = \begin{cases} \frac{n_4(k_i=\pi)}{2} \bmod 2, & \nu_i = 0 \\ \text{undefined}, & \nu_i = 1. \end{cases} \tag{121}$$

We have thus completed our definition of $\boldsymbol{M}_\nu^{\mathrm{F}}$ for $\mathcal{I}$- and $\mathcal{T}$-symmetric 3D insulators [SEq. (111)]. As noted previously in SN 3 A, because corner-mode phases in 2D reflect polarization (or fragile) topology [30, 33, 70, 95, 100, 176], then, unlike strong topological phases, corner-mode phases cannot be diagnosed through their electronic structure alone, but instead must be identified by a comparison between obstructed and unobstructed atomic limits (or FTIs). However, we note that most TIs, TCIs, and HOTIs with clean (bulk-insulating) Fermi surfaces are narrow-gap semiconductors for which it is chemically straightforward to identify which bands from which atomic orbitals have become inverted [131, 132, 196–198, 264, 265]. For example, in SN 6 B, we will demonstrate that 3D SnTe differs from an unobstructed atomic limit through double band inversions at two symmetry-related TRIM points, leading to a nontrivial $\boldsymbol{M}_\nu^{\mathrm{F}}$ vector, and, hence, a nontrivial HEND-state dislocation response.

3. $\mathcal{T}$-Symmetric Nested Wilson Loop Formulation of Partial Nested Berry Phase

In this section, we will use nested Wilson loops [30, 31, 33, 70, 95, 100, 138, 149] to rigorously relate the $\mathcal{T}$-symmetric corner-mode phases from SN 3 B 2 to a previously developed topological (polarization) index: the nested Berry phase γ_2. We will show that, while $\gamma_2 = 0$ for both the filling-anomalous and trivial phases in SN 3 B 2, a new *partial* nested Berry phase $\tilde{\gamma}_2$ can be introduced to distinguish the two insulators. During the preparation of this work, an equivalent Wilson-loop formulation of partial nested Berry phase was also introduced in SRef. 206; however the authors of that work did not form a connection between the bulk invariant and corner modes (filling anomalies), which we will establish in this section.

In SRefs. 33 and 100, the presence of anomalous corner modes in $\mathcal{I}$-symmetric, $\mathcal{T}$-broken 2D insulators was related to a nontrivial bulk (polarization) index: γ_2. Specifically, in those works, beginning with atomic orbitals with trivial Berry phases γ_1 and nested Berry phases γ_2 [*i.e.* orbitals placed at the $1a$ position in SFig. 11 ***b*** $(x, y = 0)$], it was determined that an $\mathcal{I}$-symmetric 2D insulator with two or more occupied bands and either odd, fragile Wilson winding *or* gapped Wilson loops and $\gamma_2 = \pi$ necessarily exhibits filling-anomalous corner charges.

In SRef. 100, however, an $\mathcal{I}$- and $\mathcal{T}$-symmetric 2D FTI with anomalous *Kramers pairs* of corner modes [232] was also introduced. Specifically, in Appendix A of SRef. 100, the authors introduced a 2D FTI with $\mathcal{I}$, spinful $\mathcal{T}$, and SU(2) spin symmetry that represented the weak-SOC limit of the $\mathcal{I}$- and $\mathcal{T}$-symmetric FTI that is pumped in a generic $\mathcal{I}$- and $\mathcal{T}$-symmetric HOTI [33] (equivalent to the $\mathcal{I}$- and spinful $\mathcal{T}$-symmetric FTIs previously discussed in SN 3 B 2). For the $\mathcal{I}$-, $\mathcal{T}$-, and SU(2)-symmetric 2D FTI in SRef. 100, the authors demonstrated that spin-degenerate pairs of corner states could be inferred, after the FTI was trivialized by adding trivial valence bands, by observing a nested Berry phase of $\gamma_2 = \pi$ *per spin.* Though, taken over both spin sectors, $\gamma_2 \bmod 2\pi$ was necessarily zero for both the filling-anomalous and trivial (unobstructed-atomic-limit) phases, the two phases could still be distinguished in the presence of additional SU(2) or s^z symmetries. Related corner-mode phases with net-zero γ_2 were also encountered in SRefs. 99 and 276.

However, and crucially, breaking SU(2) symmetry while preserving spinful $\mathcal{T}$ symmetry *does not* lift the filling anomaly in the model introduced in SRef. 100, though it does remove the ability to split the nested Wilson loop into spin sectors. This raises the pertinent question of whether, in the absence of SU(2) or s^z symmetries, there still exists a nested Wilson indicator of filling-anomalous corner states. In the following text, we will definitively answer this question in the positive by formulating a new *partial* nested Berry phase $\tilde{\gamma}_2$ that represents the higher-order generalization of the *time-reversal polarization* introduced in SRefs. 6 and 258 to characterize 2D TIs as helical pumping cycles of the spinful SSH chain.

To begin, consider a 2D insulator with $\mathcal{I}$-, spinful $\mathcal{T}$-, and weakly broken s^z symmetry with four valence bands and four conduction bands. We take the (valence) conduction bands to be induced from two Kramers pairs of spinful s (p) orbitals at the $1a$ Wyckoff position of layer group $p\bar{1}1'$ (SFig. 11 ***b***) [25, 70, 223, 227, 228, 237, 262]. Using the symmetry labels in Supplementary Table 1, we determine that all four of the conduction (valence) bands have positive (negative) parity eigenvalues at all four TRIM points (SFig. 10 ***b***). By construction, the occupied bands represent an unobstructed atomic limit [152]. Next, we double band invert at the Γ point [100, 102, 144], after which all four of the occupied bands at Γ now exhibit negative parity eigenvalues (boxed parity eigenvalues in SFig. 12 ***a***). As shown in SRefs. 33 and 100, once the insulating gap is reopened, the occupied bands necessarily exhibit fragile topology; specifically, the occupied bands are irrep-equivalent to ($\mathcal{T}$-reversed pairs) of the linear combination (sum and difference) of EBRs in SEq. (115). We then place an additional Kramers pair of occupied p orbitals at the $1a$ position energetically far below the four fragile valence bands, resulting in a new valence manifold with six occupied

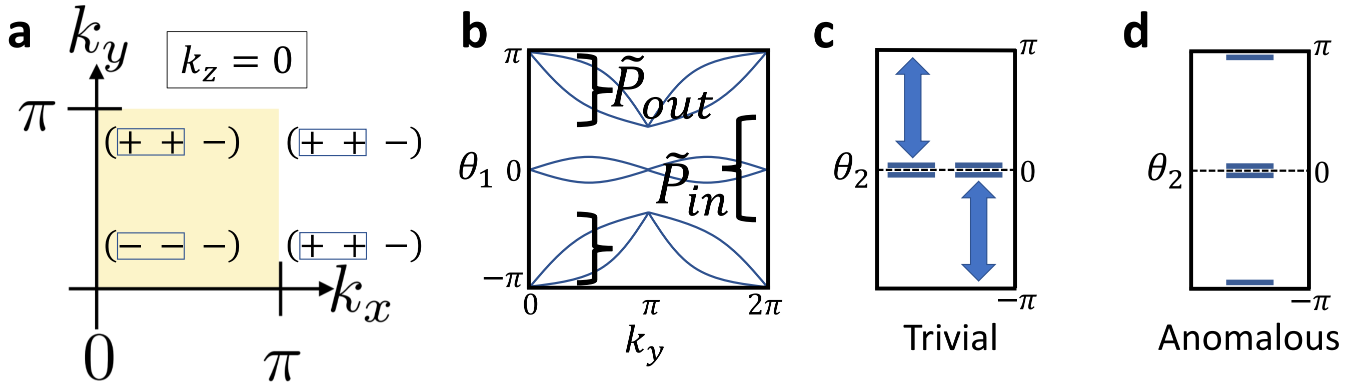

Supplementary Figure 12. **Topological nested Wilson loops in 2D planes with time-reversal and inversion symmetry.** ***a*** The parity ($\mathcal{I}$) eigenvalues per Kramers pair of the six bulk bands in the $k_z = 0$ plane of a 3D system. The boxed parity eigenvalues in ***a*** indicate that four of the bulk bands correspond in the $k_z = 0$ plane to the $\mathcal{I}$- and $\mathcal{T}$-symmetric 2D FTI with anomalous corner states introduced in SRef. 100. The remaining two bands, whose parity eigenvalues lie outside of the boxes in ***a***, are induced from a Kramers pair of spinful p orbitals at the $1a$ Wyckoff position $(x, y, z = 0)$. ***b*** A schematic depiction of the x-directed Wilson loop spectrum $W_1(k_y, k_z)$ [SEq. (122)] of an $\mathcal{I}$- and spinful $\mathcal{T}$-symmetric 3D system computed over six bulk bands with the parity eigenvalues in ***a*** in the $k_z = 0$ plane, drawn as a function of k_y and evaluated at $k_z = 0$. $W_1(k_y, k_z)$ is Wilson particle-hole symmetric at each value of $k_{y,z}$ as a consequence of bulk $\mathcal{I}\times\mathcal{T}$ symmetry [SRef. 100 and SEq. (124)], and is also Wilson particle-hole symmetric at $\mathcal{I}$-related values of $k_{y,z}$ as a consequence of bulk $\mathcal{I}$ symmetry [SRef. 33 and SEq. (125)]. Bulk $\mathcal{T}$ symmetry acts as a spinful Wilson $\mathcal{T}$ symmetry that enforces Kramers' theorem at the $\mathcal{T}$-invariant values of $k_{y,z}$ [SEq. (126)]. ***c, d*** The two possible families of nested Wilson spectra computed symmetrically [33, 100] over four Wilson bands of a 2D insulator with $\mathcal{I}$ and spinful $\mathcal{T}$ symmetry [such as the $\tilde{P}_{out}$ Wilson bands at $k_z = 0$ in ***b***]. The four nested Wilson bands in ***c, d*** exhibit nested Wilson particle-hole symmetry as a consequence of bulk $\mathcal{I}$ symmetry [SRef. 33 and SEq. (130)], as well as Kramers degeneracies at all nested Wilson energies as a consequence of bulk spinful $\mathcal{T}$ symmetry [SEq. (126)]. As shown in SEq. (137) and in the surrounding text, the nested Wilson spectrum in ***c*** can be deformed into the spectrum of a trivial insulator, because it has an even (here zero) number of Kramers pairs at $\theta_2 = \pm\pi$. Conversely, the nested Wilson spectrum in ***d*** *cannot* be deformed into the trivial spectrum in ***c*** while preserving $\mathcal{I}$ and $\mathcal{T}$ symmetries. In an $\mathcal{I}$- and spinful $\mathcal{T}$-symmetric 2D insulator whose bulk bands are induced from atoms at the $1a$ position, the nested Wilson spectrum in ***d*** also indicates the presence of a corner filling anomaly. Crucially, our analysis of the nested Wilson spectra in ***c*** and ***d*** does not depend on s^z spin symmetry labels, which are generically absent in spinful $\mathcal{T}$-symmetric insulators due to spin-orbit coupling [3, 5, 6, 258]. As shown in this section, building upon the results of SRefs. 33, 100, and 246, for an $\mathcal{I}$- and spinful $\mathcal{T}$-symmetric 2D insulator with six occupied bands that carry the parity eigenvalues in ***a***, the x-directed Wilson loop spectrum computed over the occupied bands will exhibit the Wilson band connectivity schematically depicted in ***b***, and the y-directed nested Wilson loop spectrum computed over the four $\tilde{P}_{out}$ Wilson bands in ***b*** will coincide with the anomalous (topologically nontrivial) nested Wilson loop spectrum depicted in ***d***.

bands that is irrep-equivalent to a linear combination of EBRs with only positive coefficients (SFig. 12 ***a***). As shown in SRefs. 33 and 100, the six occupied bands no longer exhibit fragile topology, allowing the calculation of (nested) Wilson loops.

For the future generalization of our (nested) Wilson calculations to 3D systems, we next take the 2D trivialized FTI with six occupied bands in the previous paragraph to be described by the Hamiltonian at $k_z = 0$ of an $\mathcal{I}$- and $\mathcal{T}$-symmetric 3D system with orthogonal lattice vectors. Taking the six occupied bands of the 3D system, we will first calculate the discretized x-directed Wilson loop (holonomy) matrix [23–25, 137, 244–246]:

$$W_1(k_y, k_z) = V(2\pi\hat{x})\Pi(k_{x0}, k_y, k_z), \tag{122}$$

where $V(2\pi\hat{x})$ is a sewing matrix that enforces the basepoint (k_{x0}) independence [23] of SEq. (122) and $\Pi(k_{x0}, k_y, k_z)$ is the product of projectors onto the occupied bands at each $\mathbf{k}$ point along the line $(k_{x0}+2\pi, k_y, k_z) \to (k_{x0}, k_y, k_z)$. More formal definitions of $W_1(k_y, k_z)$ are provided in the appendices of SRefs. 33 and 100. The eigenvalues of $W_1(k_y, k_z)$ are gauge-independent phases $\exp[i\theta_1(k_y, k_z)]$ [24, 25, 245, 246], allowing us to define a Hermitian *Wilson Hamiltonian*:

$$H_{W_1}(k_y, k_z) = -i\ln[W_1(k_y, k_z)], \tag{123}$$

whose eigenvalues take the form of real angles $\theta_1(k_y, k_z)$ and form smooth and continuous *Wilson bands* [24, 25, 246]. We refer to the values of $\theta_1(k_y, k_z)$ as *Wilson energies.*

The action of $\mathcal{I}\times\mathcal{T}$ and $\mathcal{I}$ on $H_{W_1}(k_y, k_z)$ were respectively determined in SRefs. 100 and 33, allowing us to also infer the action of spinful $\mathcal{T}$. First, in SRef. 100, it was determined that spinful $\mathcal{I}\times\mathcal{T}$ acts on the Wilson Hamiltonian

as an antiunitary particle-hole symmetry $\tilde{\Xi}$ that preserves the signs of $k_{y,z}$:

$$\tilde{\Xi}H_{W_1}(k_y,k_z)\tilde{\Xi}^{-1} = -\tilde{U}H^*_{W_1}(k_y,k_z)\tilde{U}^\dagger, \tag{124}$$

where $\tilde{U}\tilde{U}^* = -\mathbb{1}$. SEq. (124) implies that, for every Wilson eigenstate at $k_{y,z}$ with eigenvalue $\theta_1(k_y,k_z)$, there is another eigenstate, also at $k_{y,z}$, with eigenvalue $-\theta_1(k_y,k_z)$. Next, it was determined in SRef. 33 (in a reproduction of the analyses in SRefs. 25 and 246) that $\mathcal{I}$ acts on the Wilson Hamiltonian as a unitary particle-hole symmetry $\tilde{\chi}$ that flips the signs of $k_{y,z}$:

$$\tilde{\chi}H_{W_1}(k_y,k_z)\tilde{\chi}^{-1} = -H_{W_1}(-k_y,-k_z), \tag{125}$$

implying that, for every Wilson eigenstate at $k_{y,z}$ with eigenvalue $\theta_1(k_y,k_z)$, there is another eigenstate at $-k_{y,z}$ with eigenvalue $-\theta_1(k_y,k_z)$. Taken together, SEqs. (124) and (125) imply that spinful $\mathcal{T}$ symmetry acts on the Wilson Hamiltonian as an antiunitary time-reversal symmetry $\tilde{\Theta}$ that flips the signs of $k_{y,z}$:

$$\tilde{\Theta}H_{W_1}(k_y,k_z)\tilde{\Theta}^{-1} = \tilde{U}H^*_{W_1}(-k_y,-k_z)\tilde{U}^\dagger, \tag{126}$$

implying that, for every Wilson eigenstate at $k_{y,z}$ with eigenvalue $\theta_1(k_y,k_z)$, there is another eigenstate at $-k_{y,z}$ with the same eigenvalue $\theta_1(k_y,k_z)$, and that states are at least twofold degenerate [23–25] by Kramers' theorem at the $\mathcal{T}$-invariant values of $k_{y,z}$. In SFig. 12 ***b***, we schematically depict the x-directed Wilson spectrum in the $k_z=0$ plane of an $\mathcal{I}$- and $\mathcal{T}$-symmetric 3D system. Specifically, at $k_z=0$ in SFig. 12 ***b***, the Wilson spectrum is the same as that of the six occupied bands of the 2D trivialized FTI discussed in this section, whose occupied parity eigenvalues (per Kramers pair) are shown in SFig. 12 ***a***. As drawn in SFig. 12 ***b***, $W_1(k_y,k_z)$ satisfies the constraints imposed by $\mathcal{I}$ and spinful $\mathcal{T}$ symmetry [SEqs. (124) through (126)].

Next, we consider the discretized y-directed nested Wilson loop [30, 33, 95, 100] computed over spectrally isolated Kramers pairs of Wilson bands:

$$W_2(k_z) = \tilde{V}(2\pi\hat{y})\tilde{\Pi}(k_{x0},k_{y0},k_z), \tag{127}$$

where $\tilde{V}(2\pi\hat{y})$ is again a sewing matrix that enforces basepoint (k_{y0}) independence and $\tilde{\Pi}(k_{x0},k_{y0},k_z)$ is the product of projectors onto a set of *Wilson* bands along the line $(k_{x0},k_{y0}+2\pi,k_z)\rightarrow(k_{x0},k_{y0},k_z)$. More formal expressions for SEqs. (122) and (127) are also provided in the appendices of SRefs. 33 and 100. We will first determine the action of symmetries on $W_2(k_z)$ before specifically analyzing the nested Wilson spectrum $W_2(0)$ of the $\mathcal{I}$- and $\mathcal{T}$-symmetric trivialized FTI highlighted in this section and previously in SN 3 B 2. The eigenvalues of $W_2(k_y,k_z)$ are gauge-independent phases $\exp[i\theta_2(k_y,k_z)]$, allowing us to define a Hermitian *nested Wilson Hamiltonian*:

$$H_{W_2}(k_z) = -i\ln[W_2(k_z)], \tag{128}$$

whose eigenvalues take the form of real angles $\theta_2(k_z)$ and form smooth and continuous *nested Wilson bands* [30, 31, 33, 100]. We refer to the values of $\theta_2(k_z)$ as *nested Wilson energies.* At each value of k_z, the sum of the nested Wilson energies modulo 2π is equal to the nested Berry phase:

$$\gamma_2(k_z) = \sum_{n=1}^{\tilde{n}_{\text{occ}}}\theta_2^n(k_z)\text{ mod }2\pi. \tag{129}$$

It was discovered in SRef. 100 that, if $W_2(k_z)$ is calculated using a $\tilde{\Xi}$-symmetric grouping of Wilson bands (*i.e.*, using the nested Wilson projectors $\tilde{P}_{in,out}$ in SFig. 12 ***b***), then $\mathcal{I}\times\mathcal{T}$ also imposes constraints on the *nested* Wilson spectrum. In SRef. 33, the constraints imposed by $\mathcal{I}$ on $W_2(k_z)$ (when calculated on a $\tilde{\chi}$-symmetric grouping of Wilson bands) were also determined. Taken together, these constraints imply the action of $\tilde{\Theta}$, and thus spinful $\mathcal{T}$, on $W_2(k_z)$. Specifically, first, it was determined in SRef. 100 that $\tilde{\Xi}$ (and therefore $\mathcal{I}\times\mathcal{T}$) acts on the nested Wilson Hamiltonian as an antiunitary particle-hole symmetry $\tilde{\tilde{\Xi}}$ that preserves the sign of k_z:

$$\tilde{\tilde{\Xi}}H_{W_2}(k_z)\tilde{\tilde{\Xi}}^{-1} = -\tilde{\tilde{U}}H^*_{W_2}(k_z)\tilde{\tilde{U}}^\dagger, \tag{130}$$

where $\tilde{\tilde{U}}\tilde{\tilde{U}}^* = -\mathbb{1}$. SEq. (130) implies that, for every nested Wilson eigenstate at k_z with eigenvalue $\theta_2(k_z)$, there is another eigenstate, also at k_z, with eigenvalue $-\theta_2(k_z)$. Next, it was determined in SRef. 33 that $\tilde{\chi}$ (and therefore $\mathcal{I}$) acts on the nested Wilson Hamiltonian as a unitary particle-hole symmetry $\tilde{\tilde{\chi}}$ that flips the sign of k_z:

$$\tilde{\tilde{\chi}}H_{W_2}(k_z)\tilde{\tilde{\chi}}^{-1} = -H_{W_2}(-k_z), \tag{131}$$

implying that, for every nested Wilson eigenstate at k_z with eigenvalue $\theta_2(k_z)$, there is another eigenstate at $-k_z$ with eigenvalue $-\theta_2(k_z)$. Taken together, SEqs. (130) and (131) imply that $\tilde{\Theta}$ (and therefore spinful $\mathcal{T}$) acts on the nested Wilson Hamiltonian as an antiunitary time-reversal symmetry $\tilde{\tilde{\Theta}}$ that flips the sign of k_z:

$$\tilde{\tilde{\Theta}} H_{W_2}(k_z)\tilde{\tilde{\Theta}}^{-1} = \tilde{\tilde{U}} H^{*}_{W_2}(-k_z)\tilde{\tilde{U}}^{\dagger}, \tag{132}$$

implying that, for every nested Wilson eigenstate at k_z with eigenvalue $\theta_2(k_z)$, there is another eigenstate at $-k_z$ with the same eigenvalue $\theta_2(k_z)$, and that states are at least twofold degenerate by Kramers' theorem at the $\mathcal{T}$-invariant values $k_z = 0, \pi$.

In SFig. 12 ***c*, *d***, we show the two possible families of $\mathcal{I}$- and spinful $\mathcal{T}$-symmetric nested Wilson spectra $W_2(0)$ symmetrically computed over four spinful Wilson bands (returning from $\tilde{\tilde{\chi}}$ and $\tilde{\tilde{\Theta}}$, respectively, to the symbols for bulk symmetries for notational simplicity, and focusing specifically on the $\mathcal{I}$- and $\mathcal{T}$-symmetric $k_z = 0$ plane). First, in SFig. 12 ***c***, we show a nested Wilson spectrum with two Kramers pairs of states at $\theta_2 = 0$. Satisfying nested Wilson time-reversal and particle-hole symmetries [SEqs. (130) through (132)], we can "push" the two nested Wilson eigenstates in SFig. 12 ***c*** pairwise from $\theta_2 = 0$ to $\theta_2 = \pi$. In contrast, in SFig. 12 ***d***, we show a nested Wilson spectrum with one Kramers pair at $\theta_2 = 0$ and one Kramers pair at $\theta_2 = \pi$. In the nested Wilson spectrum in SFig. 12 ***d*** – which corresponds to the four spinful $\tilde{P}_{out}$ Wilson bands in SFig. 12 ***b*** – the constraints of $\mathcal{I}$ and $\mathcal{T}$ symmetry conversely *pin* the Kramers pairs in nested Wilson energy, and we cannot float the nested Wilson eigenstates away from their respectively values of θ_2. Furthermore, as shown in SRef. 33, because of the constraints imposed by $\mathcal{I}$ symmetry on $W_1(k_y, k_z)$ [SEq. (125)], then Wilson gap closures unaccompanied by energy gap closures occur in pairs that do not change $\gamma_2(0)$ (or $\gamma_2(\pi)$, if we expand our focus beyond the $k_z = 0$ plane). Crucially, in the presence of an additional SU(2) or spinful $\mathcal{T}$ symmetry, which both enforce a twofold degeneracy in the eigenvalues of $W_2(0)$ [SEq. (132)], then SEq. (125) implies that Wilson gap closures unaccompanied by energy gap closures *also* do not change $\gamma_2^{\uparrow,\downarrow}(0)$, *i.e.*, the nested Berry phase, *per spin sector*. As we will argue below, given orbitals originating from atoms at the $1a$ position (SFig. 11 ***b***), the nested Wilson spectrum in SFig. 12 ***c*** (SFig. 12 ***d***) corresponds to a trivial (filling-anomalous) 2D insulator without (with) Kramers pairs of corner modes that can only be removed by closing a bulk energy gap or breaking a symmetry. Specifically, we will see that, even though both nested Wilson spectra in SFig. 12 ***c*, *d*** exhibit $\gamma_2(0) = 0$ [SEq. (129)], the two nested Wilson spectra can still be meaningfully distinguished by the presence of an even or odd number of Kramers pairs of nested Wilson eigenstates at $\theta_2 = \pm\pi$. Lastly, for completeness, we note that the results of SRefs. 33 and 100 imply that the y-directed nested Wilson spectrum of the two spinful $\tilde{P}_{in}$ Wilson bands in SFig. 12 ***b*** would consist of a single Kramers pair of nested Wilson eigenstates pinned to $\theta_2 = \pi$, and would therefore also contain the same number of Kramers pairs of nested Wilson eigenstates at $\theta_2 = \pi$ (taken modulo 2) as the four $\tilde{P}_{out}$ Wilson bands.

To demonstrate the robustness of the number of Kramers pairs at $\theta_2 = \pm\pi$ in the nested Wilson spectrum, we will first consider the limit in which SU(2) spin rotation symmetry, and thus s^z symmetry, is restored to the $\mathcal{I}$- and $\mathcal{T}$-symmetric 2D FTI discussed throughout this section. In this case, the six valence bands remain separated from the conduction bands, and the six Wilson bands in SFig. 12 ***b*** collapse into doubly degenerate pairs while preserving the Wilson gaps between the $\tilde{P}_{in/out}$ pairs of Wilson bands. We can therefore still calculate the four-band nested Wilson spectrum of the $\tilde{P}_{\text{out}}$ Wilson bands. As determined numerically in SRef. 100, in the SU(2)-symmetric limit, the trivialized FTI discussed in this section exhibits the nested Wilson spectrum shown in SFig. 12 ***d***. Specifically, $W_2(0) = W_2$ displays one pair of $s_z = \uparrow, \downarrow$ states at $\theta_2 = 0$ and another pair at $\theta_2 = \pi$. Hence, within each spin sector:

$$\gamma_2^{\uparrow,\downarrow} = \pi, \tag{133}$$

where we have suppressed the explicit k_z dependence $\gamma_2^{\uparrow,\downarrow} = \gamma_2^{\uparrow,\downarrow}(0)$. We further recognize that, because $\mathcal{I}$ symmetry requires that nested Wilson eigenstates within each s^z sector come in nested Wilson particle-hole-symmetric pairs [SEq. (131)], then the sum in SEq. (129) simplifies:

$$\gamma_2^{\uparrow,\downarrow} = \pi \left(n_\pi^{\uparrow,\downarrow} \bmod 2\right), \tag{134}$$

where $n_\pi^{\uparrow,\downarrow}$ indicates the number of nested Wilson eigenvalues with $\theta_2 = \pm\pi$ within each spin sector $s^z = \uparrow, \downarrow$. For the 2D trivialized FTI discussed in this section, SEq. (134) specifically indicates a filling anomaly when:

$$n_\pi^{\uparrow,\downarrow} = L_{z,n}^{\text{FTI}} = 1 + 2n,\ \ n \in \{\mathbb{Z}^+, 0\}, \tag{135}$$

where $L_{z,n}^{\text{FTI}}$ also describes the number of spin-degenerate pairs of anomalous corner modes [SEq. (37)]. Because SU(2) symmetry requires that the eigenstates of W_2 appear in spin-degenerate pairs, then the total number of nested Wilson eigenstates at $\theta_2 = \pm\pi$ is given by:

$$n_\pi = n_\pi^{\uparrow} + n_\pi^{\downarrow} = 2n_\pi^{\uparrow}, \tag{136}$$

and indicates a filling anomaly if:

$$n_\pi \text{ mod } 4 = 2L_{z,n}^{\text{FTI}} \text{ mod } 4 = 2. \tag{137}$$

We next allow for weak, $\mathcal{T}$-symmetric SOC, which relaxes SU(2) and s^z symmetry. This splits the Wilson band degeneracies at generic crystal momenta, but does not close any additional bulk gaps (though it may close and reopen Wilson gaps). Hence, the nested Wilson projector $\tilde{P}_{out}$ in SFig. 12 ***b***, and thus W_2, remain well-defined throughout the introduction of SOC. Crucially, as shown in SEq. (132), because spinful $\mathcal{T}$ symmetry imposes *the same* constraint on W_2 that SU(2) symmetry did previously, namely that all nested Wilson eigenstates are doubly degenerate, then n_π mod 4 *cannot* change under the $\mathcal{T}$-symmetric relaxation of SU(2) symmetry. Specifically, while n_π itself can be changed by a Wilson gap closure unaccompanied by an energy gap closure [33], $\mathcal{I}$- and $\mathcal{T}$ symmetries require that Wilson gap closings at generic Wilson energies and crystal momenta k_y in the $k_z = 0$ plane come in sets of four (*i.e.* at $\pm\theta_1$ and $\pm k_y$), such that n_π mod 4 cannot change without a bulk energy gap closure. Additionally, as shown in SRefs. 33 and 100, a filling anomaly in an $\mathcal{I}$-, $\mathcal{T}$-, and SU(2)-symmetric insulator *remains preserved* under the $\mathcal{T}$-symmetric relaxation of SU(2) spin symmetry. Therefore, because n_π mod 4 can only be changed through a bulk gap closure that removes the filling anomaly or by lowering the bulk symmetry, SEq. (137) *remains* an indicator of nontrivial (partial) polarization topology, *even in the absence of s^z symmetry.*

Finally, using n_π, we define a *partial* or *time-reversal* nested polarization:

$$\tilde{\gamma}_2 = \pi\left[\left(\frac{n_\pi}{2}\right) \text{ mod } 2\right], \tag{138}$$

that indicates the presence of Kramers pairs of corner states in an $\mathcal{I}$- and $\mathcal{T}$-symmetric 2D insulator (given orbitals originating from atoms at the $1a$ position in SFig. 11 ***b***). SEq. (138) therefore represents the higher-order analog of time-reversal polarization, which was introduced in SRefs. 6, 8, and 258 to predict Kramers pairs of end states in $\mathcal{I}$- and $\mathcal{T}$-symmetric 1D insulators (spinful SSH chains).

While it was shown in SRefs. 33, 100, and 176 and SN 3 B 2 that n_π mod 4 [SEq. (137)] can be determined in an $\mathcal{I}$- and $\mathcal{T}$-symmetric 2D insulator using the parity eigenvalues of the occupied bands, the analysis in this section *also* applies with only minor modification to spinful C_{2z}- (twofold-rotation-) and $\mathcal{T}$-symmetric 2D insulators [33, 107–110], whose topology cannot be inferred from symmetry eigenvalues [100, 103, 104]. Therefore, our formulation of $\tilde{\gamma}_2$ [SEq. (138)] provides an essential bridge towards the identification of *non-symmetry-indicated* higher-order topology in $\mathcal{T}$-symmetric 3D insulators, which has to date only been predicted in the noncentrosymmetric structural phases of XTe_2 (X=Mo,W) [100].

4. NUMERICAL CALCULATION DETAILS: EDGE AND SCREW DISLOCATIONS

In this section, we will present numerical calculations for representative 2D and 3D insulators that confirm the dislocation responses discussed in this work. First, in SN 4 A, we will detail the dislocation response of 2D (SN 4 A 1) and 3D (SN 4 A 2) insulators with broken $\mathcal{T}$ symmetry. Then, in SN 4 B, we will detail analogous calculations demonstrating the dislocation response of $\mathcal{T}$-symmetric 2D (SN 4 B 1) and 3D (SN 4 B 2) insulators.

A. Dislocation Response without Time-Reversal Symmetry

1. 2D Point Dislocations in the Absence of $\mathcal{T}$ Symmetry

In this section, we will demonstrate the dislocation response of 2D insulators with broken $\mathcal{T}$ symmetry. We begin by comparing the minimal tight-binding model for an inversion- ($\mathcal{I}$-) symmetric Chern insulator with Chern number $|C| = 1$ to that of a stack (array) of Su-Schrieffer-Heeger (SSH) chains [216]. Consider the Bloch Hamiltonian:

$$\begin{aligned}\mathcal{H}(\boldsymbol{k}) &= \sin k_x\, \sigma^x + \xi \sin k_y\, \sigma^y \\ &\quad + \left[\alpha \cos k_x + \xi\left(1 + \beta \cos k_y\right)\right]\sigma^z,\end{aligned} \tag{139}$$

defined on a square lattice, where σ^i, $i = 0, x, y, z$ are Pauli matrices indexing spinless valence s and conduction p orbitals on each site, and $\xi = 0, 1$ and $\alpha, \beta = \pm 1$ are parameters that can be tuned to realize different topological phases. Inversion symmetry ($\mathcal{I}$) is represented by:

$$\mathcal{I}: \ \mathcal{H}(\boldsymbol{k}) \rightarrow \sigma^z \mathcal{H}(-\boldsymbol{k}) \sigma^z. \tag{140}$$

At half filling there is a single occupied band with the Bloch eigenstates $|u(\boldsymbol{k})\rangle$. At the four time-reversal-invariant momenta (TRIM points) $\bar{\boldsymbol{k}}$ of the 2D BZ (SFig. 13 ***a***) – here, because $\mathcal{T}$ symmetry is absent [223, 237], instead defined by $\mathcal{I}\bar{\boldsymbol{k}} = \bar{\boldsymbol{k}}$ mod $\mathbf{b}$, where $\mathbf{b}$ is a reciprocal lattice vector – we can define the parity eigenvalue $\lambda_{\bar{\boldsymbol{k}}}$ of the occupied band as:

$$\mathcal{I}\left|u(\bar{\boldsymbol{k}})\right\rangle = \lambda_{\bar{\boldsymbol{k}}}\left|u(\bar{\boldsymbol{k}})\right\rangle, \tag{141}$$

where $\mathcal{I}^2 = 1$ implies that $\lambda_{\bar{\boldsymbol{k}}} = \pm 1$. For $\alpha = \beta = 0$ and $\xi = 0, 1$, SEq. (139) is topologically trivial, and its occupied subspace is composed of a single band with the parity eigenvalues $\lambda_{\bar{\boldsymbol{k}}} = 1$ at all TRIM points.

For $\xi = 1$, $\mathcal{H}(\boldsymbol{k})$ describes a symmetry-indicated $|C| = 1$ Chern insulator [7, 8, 10, 246, 267, 277] that differs from a trivial (unobstructed) atomic limit by a single sign-change of the parity eigenvalue of the occupied band at $\bar{\boldsymbol{k}} = (1+\alpha, 1+\beta)\pi/2$. To determine the topology of $\mathcal{H}(\boldsymbol{k})$, we exploit that the Chern number modulo 2 of a $\mathcal{T}$-broken 2D insulator is indicated by the product of the parity eigenvalues of the occupied bands [7, 8, 10, 246, 267, 277]. Here, and throughout this work, we consider the process of closing a gap and inverting bands to be a *band inversion*, and restrict focus in this and the following section (SN 5) to band inversions that exchange the parity eigenvalues of the valence and conduction bands.

When $\xi = 0$, $\mathcal{H}(\boldsymbol{k})$ is equivalent to an array of identical x-directed SSH chains [278] indexed by k_y whose polarization (Berry phase) is indicated by the parity eigenvalues at $k_x = 0, \pi$ [246]. More generally, in this work, we define a stack or array as a *weak insulating* phase whose Bloch Hamiltonian has the same symmetry eigenvalues along a specified momentum-space direction k_i (reciprocal to a real-space direction i), and therefore the same $(d-1)$-D topological indices in BZ surfaces indexed by k_i. For example, a 3D weak TI is equivalent to a stack of 2D TIs [7–9, 211].

Having established the properties of the Hamiltonian of the pristine system [SEq. (139)], we will now probe the dislocation response. We introduce a pair of 0D dislocations with Burgers vector $\boldsymbol{B} = \hat{y}$. Through SEq. (83), we deduce that each dislocation realizes an interface between two insulators S and S' that "differ" by the Hamiltonian of the $k_y = \pi$ plane of SEq. (139):

$$\mathcal{H}(k_x, \pi) = \sin k_x\, \sigma^x + [\alpha \cos k_x + \xi(1-\beta)]\sigma^z. \tag{142}$$

Setting, $\alpha = -1$, $\mathcal{H}(k_x, \pi)$ describes an $\mathcal{I}$-symmetric SSH chain with a trivial (nontrivial) polarization for $\xi = 1$, $\beta = -1$ ($\xi = 1$, $\beta = +1$ or $\xi = 0$) when measured with respect to the valence s orbitals (SN 3 A).

To obtain the numerical results presented in SFig. 2 of the main text (reproduced in SFig. 13), we begin with a square lattice Λ of size $|\Lambda| = L^2$ with $L = 16$ and periodic boundary conditions (PBC). We then remove a line of 8 sites to create a pair of dislocations with Burgers vector $\boldsymbol{B} = \hat{y}$. We note that two is the minimal number of dislocations compatible with (untwisted) PBC: a single point dislocation introduces a fault line in the lattice that must terminate either at a boundary or at another dislocation.

Next, we analyze the system with two dislocations using the terminology established in SN 2 B 1. We begin by denoting the pristine (dislocation-free) real-space Hamiltonian corresponding to the model in SEq. (139) as H. H contains only nearest-neighbor hoppings, and has $(2L^2 \times 2L^2) = (512 \times 512)$ elements. Let $H_{a,b}$ be the components of the $(2|A| \times 2|B|)$-dimensional submatrix that couples lattice sites taken from the sets A and B. $H_{a,b}$ is the matrix obtained by removing all of the rows from the $(2L^2 \times 2L^2)$ matrix H that do not couple to lattice sites within the set A. Similarly, we remove all of the columns in $H_{a,b}$ that do not couple to the set B. We next introduce the shorthand notation:

$$\sum_{a,b} H_{a,b}|a\rangle\langle b| = \sum_{\boldsymbol{r}\in A}\sum_{\boldsymbol{r'}\in B}\sum_{\mu,\nu\in\{1,2\}} H^{\mu,\nu}_{\boldsymbol{r},\boldsymbol{r'}}|\boldsymbol{r},\mu\rangle\langle\boldsymbol{r'},\nu|, \tag{143}$$

where $|\boldsymbol{r},\mu\rangle$ denotes the basis state at position $\boldsymbol{r} \in \Lambda$ with an orbital indexed by μ [the Bloch Hamiltonian in SEq. (139) is a (2×2) matrix in the basis of spinless s and p orbitals].

We will next detail how, in a tight-binding model with nearest-neighbor hoppings and orthogonal lattice vectors, we can numerically implement a pair of point dislocations by first removing a line of sites from a pristine lattice, and then subsequently reconnecting the two lines above and below the missing line. We use the site labeling U, M, and D established in SFig. 5 ***a***, and specialize to $\boldsymbol{B} = \hat{y}$ dislocations. Defining $O = U \cup M \cup D$ and $\bar{O} = \Lambda\backslash O$, we form the expression:

$$\begin{aligned} H = &\sum_{\bar{o},\bar{o}'} H_{\bar{o},\bar{o}'}|\bar{o}\rangle\langle\bar{o}'| + \sum_{o,\bar{o}} H_{o,\bar{o}}|o\rangle\langle\bar{o}| \\ &+ \sum_{\bar{o},o} H_{\bar{o},o}|\bar{o}\rangle\langle o| + \sum_{o,o'} H_{o,o'}|o\rangle\langle o'|. \end{aligned} \tag{144}$$

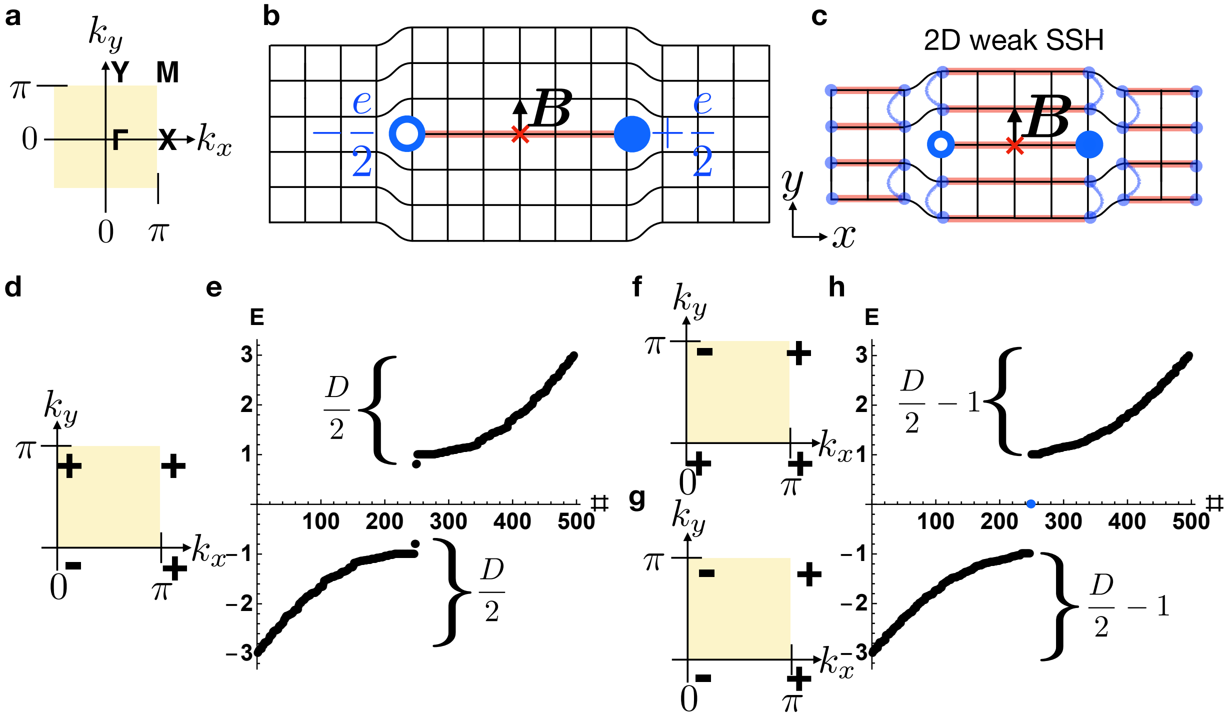

Supplementary Figure 13. **0D dislocation bound states in 2D insulators with inversion symmetry.** ***a*** The bulk BZ of a 2D rectangular magnetic crystal with only inversion ($\mathcal{I}$) symmetry. ***b*** A pair of 0D dislocations with Burgers vector $\boldsymbol{B} = \hat{y}$ that are related by a global $\mathcal{I}$ center (red $\times$) and bind anomalous fractional charges. ***c*** A pair of fractionally charged 0D dislocation states can be obtained by coupling an array of 1D x-directed SSH chains along the y direction in a manner in which one SSH chain remains decoupled from the others; two midgap states, equivalent to the end states of a nontrivial SSH chain, remain bound to the cores of the dislocations introduced by the interchain coupling. ***d*** – ***h*** Bulk parity eigenvalues and energy spectra with periodic boundary conditions (PBC) for a pair of dislocations with $\boldsymbol{B} = \hat{y}$ inserted into 2D insulators whose occupied bands carry the parity eigenvalues in ***d***, ***f***, and ***g***, respectively. ***d***, ***e*** characterize a $|C| = 1$ Chern insulator with band inversion at Γ [$\boldsymbol{M}_{\nu}^{\text{SSH}} = (0, 0)$ as defined in SN 3 A], ***f***, ***h*** characterize a $|C| = 1$ Chern insulator with band inversion at Y [$\boldsymbol{M}_{\nu}^{\text{SSH}} = (0, \pi)$], and ***g***, ***h*** characterize a weak y-directed array of SSH chains [215, 216] [$\boldsymbol{M}_{\nu}^{\text{SSH}} = (0, \pi)$]. Anomalous 0D defect states with charge $\pm e/2$ are present in cases ***f*** and ***g***, but not in ***d***, and are equivalent to the end states of an $\mathcal{I}$-symmetric, $\mathcal{T}$-broken SSH chain [215, 216] [SEq. (142), red line in ***b***], and thus persist under the relaxation of particle-hole symmetry in the form of a filling anomaly [33, 70, 212–214]. As derived in SEq. (83), the dislocation response in ***d*** – ***h*** is nontrivial when $\boldsymbol{B} \cdot \boldsymbol{M}_{\nu}^{\text{SSH}} \bmod 2\pi = \pi$.

Noting that H only contains nearest-neighbor hoppings, we then expand SEq. (144):

$$\begin{aligned}\sum_{o,o'} H_{o,o'} |o\rangle \langle o'| = &\sum_{u,u'} H_{u,u'} |u\rangle \langle u'| \\ &+ \sum_{m,m'} H_{m,m'} |m\rangle \langle m'| + \sum_{d,d'} H_{d,d'} |d\rangle \langle d'| \\ &+ \sum_{u,m} H_{u,m} |u\rangle \langle m| + \sum_{m,u} H_{m,u} |m\rangle \langle u| \\ &+ \sum_{m,d} H_{m,d} |m\rangle \langle d| + \sum_{d,m} H_{d,m} |d\rangle \langle m|.\end{aligned} \tag{145}$$

To introduce a pair of dislocations, we form the Hamiltonian $\tilde{H}$, which is defined on $\tilde{\Lambda} = \Lambda \backslash M$ – *i.e.*, the lattice obtained by taking out the set of sites M. Defining $\tilde{O} = U \cup D$ and $\bar{\tilde{O}} = \tilde{\Lambda} \backslash \tilde{O}$, $\tilde{H}$ can be expanded as:

$$\begin{aligned}\tilde{H} = &\sum_{\bar{\tilde{o}},\bar{\tilde{o}}'} H_{\bar{\tilde{o}},\bar{\tilde{o}}'} |\bar{\tilde{o}}\rangle \langle \bar{\tilde{o}}'| + \sum_{\tilde{o},\bar{\tilde{o}}} H_{\tilde{o},\bar{\tilde{o}}} |\tilde{o}\rangle \langle \bar{\tilde{o}}| \\ &+ \sum_{\bar{\tilde{o}},\tilde{o}} H_{\bar{\tilde{o}},\tilde{o}} |\bar{\tilde{o}}\rangle \langle \tilde{o}| + \sum_{\tilde{o},\tilde{o}'} H_{\tilde{o},\tilde{o}'} |\tilde{o}\rangle \langle \tilde{o}'|,\end{aligned} \tag{146}$$

in which we can further expand:

$$\begin{aligned}\sum_{\tilde{o},\tilde{o}'} H_{\tilde{o},\tilde{o}'} |\tilde{o}\rangle \langle \tilde{o}'| = &\sum_{u,u'} H_{u,u'} |u\rangle \langle u'| + \sum_{d,d'} H_{d,d'} |d\rangle \langle d'| \\ &+ \sum_{u,d} \tilde{H}_{u,d} |u\rangle \langle d| + \sum_{d,u} \tilde{H}_{d,u} |d\rangle \langle u|.\end{aligned} \tag{147}$$

Finally, we conclude that:

$$\tilde{H}_{u,d} = H_{u,m}, \quad \tilde{H}_{d,u} = H_{d,m}, \tag{148}$$

such that the matrix elements of $\tilde{H}$ between sites in U and sites in D are given by those of H between U and M.

After following the prescription in SEqs. (144) through (148), we obtain a Hamiltonian $\tilde{H}$ that contains two $\boldsymbol{B} = \hat{y}$ point dislocations on the lattice $\tilde{\Lambda}$ and $[2(L^2 - 8) \times 2(L^2 - 8)] = (496 \times 496)$ elements. We will now discuss the electronic structure of $\tilde{H}$ in detail for characteristic values of ξ and β.

a. $\xi = 1$, $\beta = -1$: $|C| = 1$ *Chern insulator with band inversion at* Γ. This case corresponds to an inversion-symmetry-indicated $|C| = 1$ Chern insulator [7, 8, 10, 246, 267, 277] driven by a single band inversion at $\boldsymbol{k} = (0, 0)$ in SEq. (139). In this case, the bulk characterizes a strong topological phase. Because the bands at X, Y, and M are uninverted relative to an atomic insulator with orbitals at the $1a$ position, the weak SSH invariant $\boldsymbol{M}_{\nu}^{\text{SSH}} = (0, 0)$ (SN 3 A). This implies that $\boldsymbol{B} \cdot \boldsymbol{M}_{\nu}^{\text{SSH}} \bmod 2\pi = 0$, and therefore, that the dislocations do not bind anomalous charges ($q \bmod e = 0$). In SFig. 13 $\boldsymbol{d}, \boldsymbol{e}$, we respectively show the parity eigenvalues of the occupied bands and the dislocation spectrum, which does not exhibit a filling anomaly or midgap dislocation bound states.

b. $\xi = 1$, $\beta = +1$: $|C| = 1$ *Chern insulator with band inversion at* Y. This case describes a $|C| = 1$ Chern insulator [7, 8, 10, 246, 267, 277] driven by band inversion at the Y point (SFig. 13 $\boldsymbol{a}$). Relative to the initial $1a$ atomic insulator, the band inversion at Y has changed not only the strong index (2D Chern number), but also the weak SSH indices [the parity eigenvalues of the occupied bands are shown in SFig. 13 $\boldsymbol{f}$]. Specifically, the bulk exhibits a weak SSH invariant $\boldsymbol{M}_{\nu}^{\text{SSH}} = (0, \pi)$ as defined in SN 3 A. This implies that $\boldsymbol{B} \cdot \boldsymbol{M}_{\nu}^{\text{SSH}} \bmod 2\pi = \pi$, indicating that the dislocation response is nontrivial. Correspondingly, in the dislocation spectrum (SFig. 13 $\boldsymbol{h}$), we observe one midgap-localized zero mode per dislocation. The zero modes are protected by the chiral (*i.e.* unitary particle-hole) symmetry Π, which is defined through the action:

$$\Pi: \ \mathcal{H}(k_x, \pi) \rightarrow \sigma^y \mathcal{H}(k_x, \pi) \sigma^y, \tag{149}$$

such that Π is a symmetry of $\mathcal{H}(k_x, \pi)$ if $\Pi \mathcal{H}(k_x, \pi) \Pi^{-1} = \sigma^y \mathcal{H}(k_x, \pi) \sigma^y = -\mathcal{H}(k_x, \pi)$. Crucially, if we were to relax Π symmetry, then the midgap states could be pushed out of the gap. However, if we preserve $\mathcal{I}$ symmetry while breaking Π, then $\pm e/2$ end charges would still remain bound to the dislocations [279], in a generalization of the conclusions of Goldstone and Wilczek [212–214].

Equivalently, in the absence of chiral symmetry, we can also identify the nontrivial topology by counting the number of states that are occupied up to a given Fermi level in the gap. Let $\tilde{H}$ be a $(D \times D)$ matrix [in our numerics, $D = 496$, as explained in the text following SEq. (148)]. Comparing SFigs. 13 $\boldsymbol{e}$ and $\boldsymbol{h}$, we observe that the two spectra differ by the presence of two midgap states and the absence of one state from each of the valence and conduction manifolds. When chiral symmetry is broken, the energy of the midgap states can be shifted, but only in a manner that preserves $\mathcal{I}$ symmetry, leaving the two states degenerate in the thermodynamic limit. This implies that any gapped Fermi level that encloses either $(D/2 - 1)$ or $(D/2 + 1)$ occupied states indicates a nontrivial topology, whereas a Fermi level enclosing $D/2$ occupied states indicates trivial bulk topology (this is also detailed in SFigs. 5 $\boldsymbol{b}, \boldsymbol{c}$). More generally, the presence of anomalous 0D states in arbitrary dimensions can be diagnosed in a similar manner by constructing a 0D system with a boundary that preserves a global point group symmetry and observing an imbalance in the number of states above and below the gap that cannot be resolved without breaking a symmetry or closing the gap (*i.e.* a filling anomaly) [32, 33, 70, 141–143, 176].

c. $\xi=0$*: Weak Array of x-Directed SSH Chains.* In this case, $\mathcal{H}(\boldsymbol{k})$ is independent of β, and SEq. (139) describes a y-directed array of identical x-directed SSH chains [278]. Specifically, the Hamiltonian [SEq. (139) with $\xi=0$] is in this case completely independent of k_y. For each SSH chain, the nontrivial polarization is indicated by the difference in the parity eigenvalues of the occupied band at $k_x=0,\pi$ [6, 215–218, 246, 258, 261]. This case is an example of weak topology, because the Hamiltonian $\mathcal{H}(\boldsymbol{k})$ can be deformed into a real-space array of decoupled 1D chains without breaking a symmetry or closing the bulk gap. Relative to the initial $1a$ atomic insulator, $\mathcal{H}(\boldsymbol{k})$ features band inversions at Γ and Y (the parity eigenvalues are shown in SFig. 13 ***g***), such that the bulk exhibits a trivial symmetry-indicated Chern number C mod $2=0$ and nontrivial weak SSH indices, and is irrep-equivalent to an OAL from $1a$ $[(x,y)=(0,0)]$ to $1c$ $[((x,y)=(1/2,0)]$ (SFig. 11 ***b***). Specifically, the bulk exhibits a weak SSH invariant $\boldsymbol{M}_{\nu}^{\text{SSH}}=(0,\pi)$ as defined in SN 3 A. This implies that, even though the bulk is an OAL (SN 3 A), the dislocation response is nontrivial. Correspondingly, in the dislocation spectrum (SFig. 13 ***h***), we observe a filling anomaly.

For the present case of an array of SSH chains, we can also understand the existence of dislocation midgap states intuitively without invoking the more general theory used to derive SEq. (142). As depicted in SFig. 13 ***c***, the dislocations introduce an uncoupled SSH chain into the system whose end states become the dislocation bound states. As long as the "leftover" SSH chain contains an inversion center, its end states also induce a system filling anomaly, consistent with the numerical results shown in SFig. 13 ***h***.

2. 3D Screw Dislocations in the Absence of $\mathcal{T}$ Symmetry

In this section, we will demonstrate the screw dislocation response of 3D insulators with broken $\mathcal{T}$ symmetry. We begin by comparing the minimal tight-binding model for an axion insulator (AXI) [30–33, 96, 97, 100, 101, 106–110, 172, 173, 175] to that of a weak stack of 2D fragile topological insulators (FTIs) [33, 100], where we retain $\mathcal{I}$ symmetry in all cases. Consider the Bloch Hamiltonian:

$$\begin{aligned}\mathcal{H}(\boldsymbol{k}) = &\sin k_x\,\tau^z\sigma^x+\sin k_y\,\tau^z\sigma^y+\xi\sin k_z\,\tau^z\sigma^z\\ &+[\alpha\cos k_x+\beta\cos k_y+\xi\,(2+\gamma\cos k_z)]\,\tau^x\sigma^0\\ &+\xi\,\delta\,(\tau^x\sigma^x+\tau^x\sigma^y+\tau^x\sigma^z),\end{aligned}\tag{150}$$

defined on a square lattice. In SEq. (150), τ^i and σ^i, $i=0,x,y,z$ are respectively two sets of Pauli matrices indexing sublattice and orbital degrees of freedom, $\xi=0,1$ and $\alpha,\beta,\gamma=\pm1$ are parameters that can be tuned to realize different topological phases, and δ is a small parameter that gaps out the bulk (we will use $\delta=1/4$ in all calculations). We note that when $\delta=0$, SEq. (150) is instead the Hamiltonian of two uncoupled 3D Weyl semimetals [42, 155]. Throughout this work, we abbreviate the Kronecker product of Pauli matrices as $\tau^i\otimes\sigma^j\equiv\tau^i\sigma^j$.

In SEq. (150), $\mathcal{I}$ symmetry is represented by:

$$\mathcal{I}:\ \mathcal{H}(\boldsymbol{k})\rightarrow\tau^x\sigma^0\mathcal{H}(-\boldsymbol{k})\tau^x\sigma^0.\tag{151}$$

At the eight time-reversal invariant momenta (TRIM points) $\bar{\boldsymbol{k}}$ of the 3D BZ (SFig. 14 ***a***) – here, because $\mathcal{T}$ symmetry is absent [223, 237], instead defined by $\mathcal{I}\bar{\boldsymbol{k}}=\bar{\boldsymbol{k}}$ mod $\mathbf{b}$, where $\mathbf{b}$ is a reciprocal lattice vector – we can again compute the parity eigenvalues $\lambda^n_{\bar{\boldsymbol{k}}}$, $n=1,2$ of the two occupied bands, where $\lambda^n_{\bar{\boldsymbol{k}}}=\pm1$.

For $\alpha=\beta=\gamma=0$ and $\xi=0,1$, SEq. (150) is topologically trivial, and the occupied subspace is composed of two bands with equal parity eigenvalues $\lambda_{\bar{\boldsymbol{k}}}=1$ at all TRIM points. For $\xi=1$, $\mathcal{H}(\boldsymbol{k})$ describes an inversion-symmetry-indicated axion insulator (AXI) [33, 97, 106–110, 155, 172–175] that differs from a trivial (unobstructed) atomic limit by a sign-change of the parity eigenvalues of the two occupied bands (*i.e.* double band inversion) at $\bar{\boldsymbol{k}}=(1+\alpha,1+\beta,1+\gamma)\pi/2$. For $\xi=0$, $\mathcal{H}(\boldsymbol{k})$ describes a weak z-directed stack of identical inversion-symmetry-indicated [33, 97, 106–110, 155, 172–175] 2D FTIs with $\pm e/2$ corner charges.

Having established the properties of the Hamiltonian of the pristine system [SEq. (150)], we will now probe the dislocation response. Previously, in SN 4 A 1, we discussed 0D states bound to point dislocations in 2D crystals. In 3D, there are point, screw, and mixed [127] (as well as partial [79]) dislocations. In this work, we focus on defects with integer Burgers vectors, for which the presence of topological bound states only depends on the direction and length of the Burgers vector, as opposed to the details of the dislocation (see SN 2). Hence, in this section, we will restrict focus to 3D screw dislocations, with the understanding that our conclusions also apply to edge and mixed dislocations with integer Burgers vectors. In particular in this work, for simplicity, we have not performed numerical analyses of systems with mixed dislocations, because mixed dislocations have position-dependent sense vectors, whereas the sense vectors of screw and edge dislocations are always parallel and perpendicular to the Burgers vector, respectively [127].

We introduce a pair of 1D screw dislocations with Burgers vector $\boldsymbol{B}=\hat{z}$ and opposite chiralities [SEq. (42)] into the system described in the text surrounding SEq. (150). Through SEq. (83), we deduce that each screw realizes an

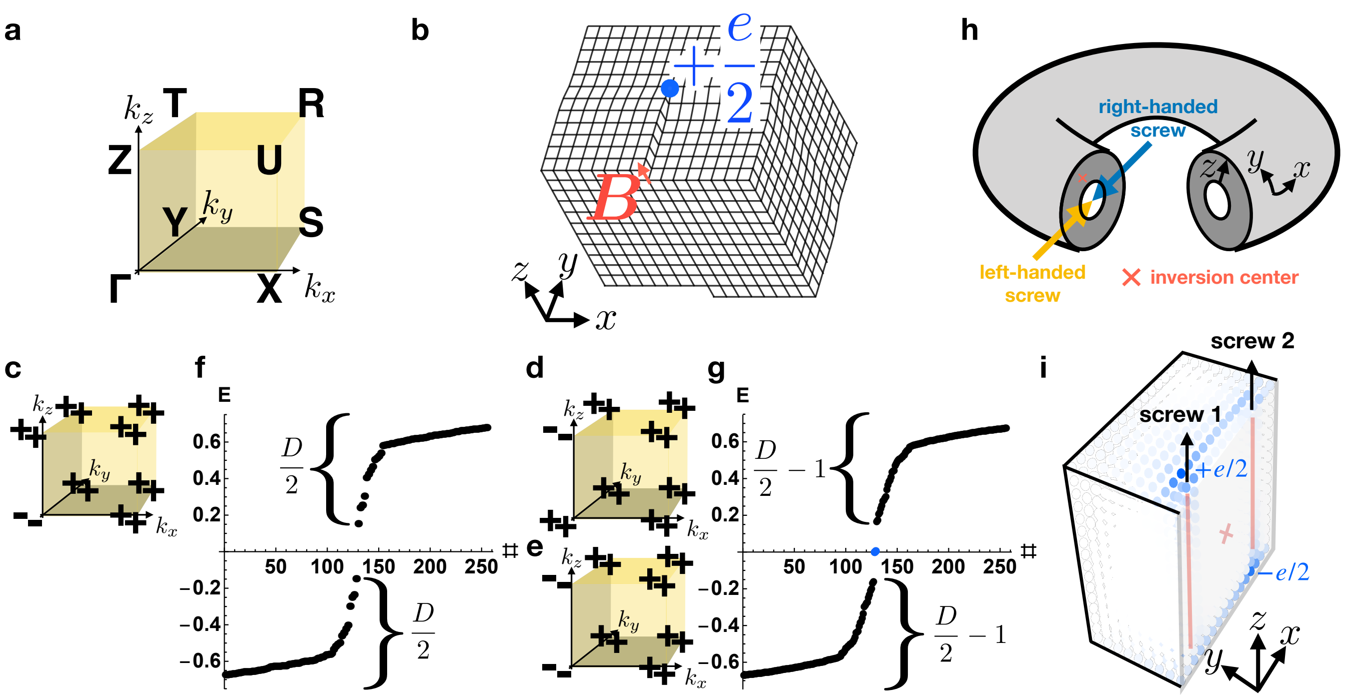

Supplementary Figure 14. **0D screw dislocation bound states in 3D insulators with inversion symmetry.** ***a*** The bulk BZ of a 3D orthorhombic crystal with only inversion ($\mathcal{I}$) symmetry. ***b*** A screw dislocation in an $\mathcal{I}$-symmetric crystal with Burgers vector $\boldsymbol{B}=\hat{z}$ in a geometry with open boundary conditions in all directions. ***c-g*** Bulk parity eigenvalues and hollow-doughnut-boundary-condition (HDBC) (***h***) energy spectra for the defect in ***b*** when the crystal with the BZ in ***a*** is equivalent to ***c***, ***f*** an $\mathcal{I}$-symmetric axion insulator (AXI) [30–33, 96, 97, 100, 101, 106–110, 172, 173, 175] with double band inversion at Γ [$\boldsymbol{M}_\nu^{\mathrm{F}}=(0,0,0)$ as defined in SN 3 B], ***d***, ***g*** an AXI with double band inversion at Z [$\boldsymbol{M}_\nu^{\mathrm{F}}=(0,0,\pi)$], and ***e***, ***g*** a weak z-directed stack of $\mathcal{I}$-symmetric 2D FTIs with $\pm e/2$ corner charges [33, 100] [$\boldsymbol{M}_\nu^{\mathrm{F}}=(0,0,\pi)$]. The HDBC geometry is closely related to the "Corbino doughnut" geometry employed in SRef. 8 to characterize 3D TIs; however, in this work, we insert screw dislocations (and later flux tubes) in a different arrangement than in SRef. 8. In the absence of dislocations, the HDBC geometry does not have any hinges. Hence, the system with HDBC, in the absence of dislocations, exhibits the same energy spectrum whether the bulk is topologically equivalent to a trivial insulator or an AXI. In ***f***, ***g***, only the 256 lowest-lying states are shown. Anomalous 0D (HEND) states with charge $\pm e/2$ are present in ***d*** and ***e***, but are absent in ***c***. The HEND states in ***d*** and ***e*** are equivalent to the corner states of an $\mathcal{I}$-symmetric 2D FTI, and thus persist under the relaxation of particle-hole symmetry [33, 70, 100]. ***i*** Real-space-resolved absolute square of the midgap eigenstates in ***g*** for the model in SEq. (150) on a $16\times16\times16$ lattice. $\mathcal{I}$-related bound states appear on two of the four screw dislocation ends. The inversion center in ***i*** is marked with a red X. Consistent with the analytic calculations in the text preceding SEq. (83), the dislocation response in ***d*** – ***h*** is nontrivial when $\boldsymbol{B}\cdot\boldsymbol{M}_\nu^{\mathrm{F}}$ mod $2\pi=\pi$.

interface between two insulators S and S' that "differ" by the Hamiltonian of the $k_z=\pi$ plane of SEq. (150):

$$\begin{aligned}\mathcal{H}(k_x,k_y,\pi) =& \sin k_x\,\tau^z\sigma^x+\sin k_y\,\tau^z\sigma^y\\ &+[\alpha\cos k_x+\beta\cos k_y+\xi(2-\gamma)]\tau^x\sigma^0\\ &+\xi\,\delta\,(\tau^x\sigma^x+\tau^x\sigma^y+\tau^x\sigma^z).\end{aligned}\tag{152}$$

Setting $\alpha=\beta=-1$, $\delta=1/4$, SEq. (152) describes an $\mathcal{I}$-symmetric 2D FTI [33, 100] (trivial insulator) when $\xi=1$, $\gamma=+1$ ($\xi=1$, $\gamma=-1$).

To obtain the numerical results presented in SFig. 4 of the main text (reproduced in SFig. 14), we begin with a square lattice Λ of size $|\Lambda|=L^2$ with $L=16$ and PBC to model the (x,y)-plane, while keeping k_z as a quantum number that labels states by their momentum in the z direction. We then modify the hoppings across a plane of 8 sites in Λ with a fixed y coordinate to create a pair of screw dislocations with Burgers vector $\boldsymbol{B}=\hat{z}$. We note that two is the minimal number of screw dislocations compatible with untwisted PBC.

Next, we analyze the system with two dislocations using the terminology established in SN 2 B 3. We begin by denoting the pristine real-space Hamiltonian corresponding to the Fourier transform of the model in SEq. (150)

within the (x, y)-plane as $H(k_z)$. $H(k_z)$ contains only nearest-neighbor hoppings, and has $(4L^2 \times 4L^2) = (1024 \times 1024)$ elements for each value of k_z. As previously in SEq. (143), $H_{a,b}$ denotes the matrix obtained by removing all of the rows from H that do not couple to lattice sites within the (freely specifiable) set A, and removing all of the columns in H that do not couple to the set B, such that the only remaining couplings are between A and B. We next introduce the shorthand notation:

$$\begin{aligned}&\sum_{a,b} H_{a,b}(k_z)|a\rangle\langle b|\\&=\sum_{\boldsymbol{r}\in A}\sum_{\boldsymbol{r'}\in B}\sum_{\mu,\nu=1\ldots 4} H^{\mu,\nu}_{\boldsymbol{r},\boldsymbol{r'}}(k_z)|\boldsymbol{r},\mu\rangle\langle \boldsymbol{r'},\nu|,\end{aligned} \tag{153}$$

where $|\boldsymbol{r},\mu\rangle$ denotes the basis state at position $\boldsymbol{r}\in\Lambda$ with an orbital indexed by μ, where the Bloch Hamiltonian in SEq. (150) is a (4×4) matrix in a basis with four orbitals. Additionally, in SEq. (153), $H^{\mu,\nu}_{\boldsymbol{r},\boldsymbol{r'}}$ are the real-space matrix elements that follow from Fourier transforming SEq. (150).

Using the site labeling U and M established in SFig. 5 $\boldsymbol{a}$, where here M is the 8-site plane across which hoppings are modified, we will now detail how we numerically implement a pair of $\boldsymbol{B}=\hat{z}$ screw dislocations. Defining $O = U\cup M$ and $\bar{O}=\Lambda\backslash O$, we form the expression:

$$\begin{aligned}H(k_z) =& \sum_{\bar{o},\bar{o}'} H_{\bar{o},\bar{o}'}(k_z)|\bar{o}\rangle\langle\bar{o}'| + \sum_{o,\bar{o}} H_{o,\bar{o}}(k_z)|o\rangle\langle\bar{o}|\\&+\sum_{\bar{o},o} H_{\bar{o},o}(k_z)|\bar{o}\rangle\langle o| + \sum_{o,o'} H_{o,o'}(k_z)|o\rangle\langle o'|.\end{aligned} \tag{154}$$

We then expand SEq. (154):

$$\begin{aligned}&\sum_{o,o'} H_{o,o'}(k_z)|o\rangle\langle o'|\\&=\sum_{u,u'} H_{u,u'}(k_z)|u\rangle\langle u'| + \sum_{m,m'} H_{m,m'}(k_z)|m\rangle\langle m'|\\&\quad+\sum_{u,m} H_{u,m}(k_z)|u\rangle\langle m| + \sum_{m,u} H_{m,u}(k_z)|m\rangle\langle u|.\end{aligned} \tag{155}$$

To introduce a pair of screw dislocations, we form the Hamiltonian $\tilde{H}(k_z)$, which is defined on Λ and labeled by k_z. $\tilde{H}(k_z)$ can be expanded as:

$$\begin{aligned}\tilde{H}(k_z) =& \sum_{\bar{o},\bar{o}'} H_{\bar{o},\bar{o}'}(k_z)|\bar{o}\rangle\langle\bar{o}'| + \sum_{o,\bar{o}} H_{o,\bar{o}}(k_z)|o\rangle\langle\bar{o}|\\&+\sum_{\bar{o},o} H_{\bar{o},o}(k_z)|\bar{o}\rangle\langle o| + \sum_{o,o'} \tilde{H}_{o,o'}(k_z)|o\rangle\langle o'|,\end{aligned} \tag{156}$$

in which we can further expand:

$$\begin{aligned}&\sum_{o,o'} \tilde{H}_{o,o'}(k_z)|o\rangle\langle o'|\\&=\sum_{u,u'} H_{u,u'}(k_z)|u\rangle\langle u'| + \sum_{m,m'} H_{m,m'}(k_z)|m\rangle\langle m'|\\&\quad+\sum_{u,m} e^{-\mathrm{i}k_z} H_{u,m}(k_z)|u\rangle\langle m| + \sum_{m,u} e^{\mathrm{i}k_z} H_{m,u}(k_z)|m\rangle\langle u|,\end{aligned} \tag{157}$$

where the exponential factors $e^{\pm ik_z}$ implement the screw dislocations (we have set the lattice spacing to $a_{x,y,z}=1$). To summarize, SEqs. (154) through (157) describe how, in a tight-binding model with nearest-neighbor hoppings and orthogonal lattice vectors, we numerically implement a pair of screw dislocations by multiplying all hoppings across the plane M in a pristine lattice by the momentum-dependent phase factor $e^{\mathrm{i}k_z}$ in one direction, and $e^{-\mathrm{i}k_z}$ in the other direction, to obtain a Hermitian Hamiltonian. After following this prescription, we obtain a Hamiltonian $\tilde{H}(k_z)$ that contains two $\boldsymbol{B}=\hat{z}$ screw dislocations with opposite sense vectors [defined in the text surrounding SEq. (42)]. We will now discuss the electronic structure of $\tilde{H}(k_z)$ in detail for characteristic values of ξ and γ.

a. $\xi = 1$, $\gamma = -1$: Axion insulator with double band inversion at Γ This case corresponds to an inversion-symmetry-indicated AXI driven by a pair of band inversions at $\boldsymbol{k} = (0,0,0)$ in SEq. (150). In this case, the bulk characterizes a strong topological phase (specifically, an AXI). Because the bands at X, Y, M, Z, U, R, and T are uninverted relative to an atomic insulator with orbitals at the $1a$ position, then the weak fragile invariant $\boldsymbol{M}_\nu^{\mathrm{F}} = (0,0,0)$ (SN 3 B). This implies that $\boldsymbol{B} \cdot \boldsymbol{M}_\nu^{\mathrm{F}} \bmod 2\pi = 0$, and therefore, that the dislocations do not bind anomalous charges ($q \bmod e = 0$). In SFig. 14 ***c***, ***f***, we respectively show the parity eigenvalues of the occupied bands and the dislocation spectrum, which does not exhibit a filling anomaly or midgap dislocation bound states.

b. $\xi = 1$, $\gamma = +1$: Axion insulator with double band inversion at Z This case describes an AXI driven by double band inversion at the Z point (SFig. 14 ***a***). Relative to the initial trivial atomic insulator, the double band inversion at Z has changed not only the strong AXI index, but also the weak fragile indices [the parity eigenvalues of the occupied bands are shown in SFig. 14 ***d***]. Specifically, the bulk exhibits a weak fragile invariant $\boldsymbol{M}_\nu^{\mathrm{F}} = (0,0,\pi)$ as defined in SN 3 B. This implies that $\boldsymbol{B} \cdot \boldsymbol{M}_\nu^{\mathrm{F}} \bmod 2\pi = \pi$, indicating that the dislocation response is nontrivial. Correspondingly, in the dislocation spectrum (SFig. 14 ***g***), we observe one midgap-localized zero mode per dislocation. The zero modes are protected at zero energy by the chiral (*i.e.* unitary particle-hole) symmetry Π, which is defined through the action:

$$\Pi: \ \mathcal{H}(k_x, k_y, \pi) \rightarrow \tau^y \sigma^0 \mathcal{H}(k_x, k_y, \pi) \tau^y \sigma^0, \tag{158}$$

such that Π is a symmetry of $\mathcal{H}(k_x, k_y, \pi)$ if $\Pi \mathcal{H}(k_x, k_y, \pi) \Pi^{-1} = \tau^y \sigma^0 \mathcal{H}(k_x, k_y, \pi) \tau^y \sigma^0 = -\mathcal{H}(k_x, k_y, \pi)$. Crucially, if we were to relax Π symmetry, then the midgap states could be pushed out of the gap. However, if we preserve $\mathcal{I}$ symmetry while breaking Π, then $\pm e/2$ end charges would still remain bound to the dislocations [279], in a generalization of the conclusions of Goldstone and Wilczek [212–214]. Equivalently, in the absence of chiral symmetry, we can also identify the nontrivial topology by counting the number of states that are occupied up to a given Fermi level in the gap [33, 70, 141–143, 176], as we did previously in SN 4 A 1 b. Let the 3D real-space Hamiltonian corresponding to the system with two screw dislocations be a $(D \times D)$ matrix. Comparing SFigs. 14 ***f*** and ***g***, we observe that the two spectra differ by the presence of two midgap states and the absence of one state from each of the valence and conduction manifolds. When chiral symmetry is broken, the energy of the midgap states can be shifted in a manner that preserves $\mathcal{I}$ symmetry, leaving the two states degenerate in the thermodynamic limit. This implies that any gapped Fermi level that encloses either $(D/2-1)$ or $(D/2+1)$ occupied states indicates a nontrivial topology, whereas a Fermi level enclosing $D/2$ occupied states indicates trivial bulk topology. More generally, the presence of anomalous 0D states in arbitrary dimensions can be diagnosed in a similar manner by constructing a 0D system with a pair of generalized dislocations that preserve a global point group symmetry and observing an imbalance in the number of states above and below the gap that cannot be resolved without breaking a symmetry or closing the gap (*i.e.*, a filling anomaly) [32, 33, 70, 141–143, 176].

c. $\xi = 0$: Weak FTI stack In this case, $\mathcal{H}(\boldsymbol{k})$ is independent of γ, and SEq. (150) describes a z-directed stack of identical 2D FTI models. Specifically, the Hamiltonian [SEq. (150) with $\xi = 0$] is in this case completely independent of k_z. This is an example of weak topology, because the Hamiltonian $\mathcal{H}(\boldsymbol{k})$ can be deformed into a real-space, z-directed stack of decoupled 2D FTIs without breaking a symmetry or closing the bulk gap. Relative to the initial trivial atomic insulator, $\mathcal{H}(\boldsymbol{k})$ features band inversions at Γ and Z (the parity eigenvalues are shown in SFig. 14 ***e***), such that the bulk exhibits a nontrivial weak fragile invariant $\boldsymbol{M}_\nu^{\mathrm{F}} = (0,0,\pi)$ as defined in SN 3 A. This implies that even though the bulk is an FTI (which is Wannierizable under the addition of trivial bands, see SN 3 B), the dislocation response is still nontrivial. Correspondingly, in the dislocation spectrum (SFig. 14 ***g***), we observe a filling anomaly.

For the present case of an array of FTI planes, we can also understand the existence of dislocation midgap states intuitively without invoking the more general theory used to derive SEq. (152). Specifically, if we were to insert a loop of edge dislocations with Burgers vector $\boldsymbol{B} = \hat{z}$ (instead of screw dislocations), then we would effectively introduce an uncoupled FTI into the system whose corner states become dislocation end states. As long as the "leftover" FTI contains an inversion center, then its corner states also induce a system filling anomaly, consistent with the numerical results shown in SFig. 14 ***g***.

B. Dislocation Response with Time-Reversal Symmetry

1. 2D Point Dislocations in the Presence of $\mathcal{T}$ Symmetry

In this section, we will demonstrate the dislocation response of 2D insulators with $\mathcal{T}$ symmetry. We begin by comparing the minimal tight-binding model of an inversion- ($\mathcal{I}$-) symmetric 2D topological insulator (TI) to that of a stack (array) of $\mathcal{I}$- and $\mathcal{T}$-symmetric Su-Schrieffer-Heeger (SSH) chains [215–218, 258]. Consider the Bloch

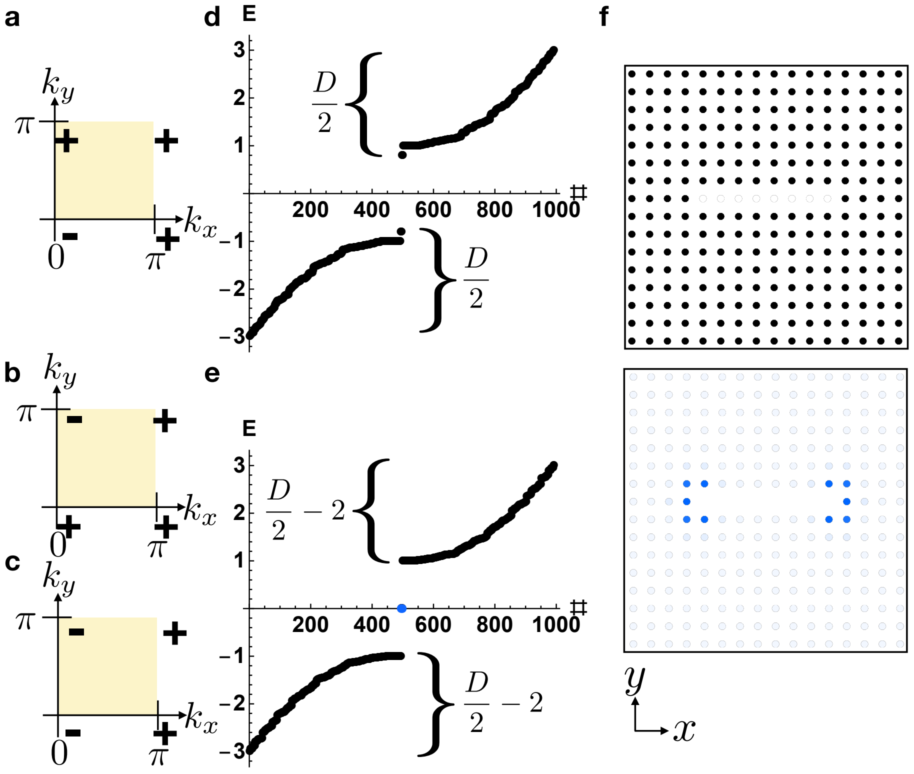

Supplementary Figure 15. **0D point dislocation bound states in 2D insulators with inversion and time-reversal symmetry.** ***a*-*e*** Bulk parity [inversion ($\mathcal{I}$)] eigenvalues and PBC energy spectra for the defect in SFig. 13 ***b*** when the bulk is equivalent to ***a***, ***d*** a 2D TI with a Kramers pair of band inversions at Γ [$\boldsymbol{M}_\nu^{\text{SSH}}=(0,0)$ as defined in SN 3 A], ***b***, ***e*** a 2D TI with a Kramers pair of band inversions at Y [$\boldsymbol{M}_\nu^{\text{SSH}}=(0,\pi)$], ***c***, ***e*** a weak y-directed array of time-reversal- ($\mathcal{T}$-) symmetric SSH chains [$\boldsymbol{M}_\nu^{\text{SSH}}=(0,\pi)$]. Kramers pairs of parity eigenvalues are denoted by a single $\pm$ symbol. Anomalous 0D defect states with spin-charge separation (SN 2 A) are present in cases ***b*** and ***c***, but not in ***a***, and are equivalent to the end states of an $\mathcal{I}$- and $\mathcal{T}$-symmetric SSH chain [215–218, 258], and thus persist under the relaxation of particle-hole symmetry in the form of a filling anomaly [33, 70, 141–143, 176, 212–214]. ***f*** Real-space geometry with a pair of point dislocations and absolute square of the wavefunction of the midgap states in ***e*** (indicated in blue in the lower panel) on a 16×16 lattice. As derived in SEq. (83), the dislocation response in ***a*** – ***e*** is nontrivial when $\boldsymbol{B}\cdot\boldsymbol{M}_\nu^{\text{SSH}}$ mod $2\pi=\pi$.

Hamiltonian:

$$\begin{aligned}\mathcal{H}(\boldsymbol{k}) =& \sin k_x\,\tau^0\sigma^x+\xi\sin k_y\,\tau^0\sigma^y\\ &+\left[\alpha\cos k_x+\xi\left(1+\beta\cos k_y\right)\right]\tau^z\sigma^z,\end{aligned} \tag{159}$$

defined on a square lattice, where $\alpha,\beta=\pm1$ are parameters that can be tuned to realize different topological phases. Time-reversal and inversion symmetry are represented by:

$$\begin{aligned}\mathcal{T}:&\ \mathcal{H}(\boldsymbol{k})\rightarrow\tau^x\sigma^y\mathcal{H}^*(-\boldsymbol{k})\tau^x\sigma^y,\\ \mathcal{I}:&\ \mathcal{H}(\boldsymbol{k})\rightarrow\tau^z\sigma^z\mathcal{H}(-\boldsymbol{k})\tau^z\sigma^z.\end{aligned} \tag{160}$$

At half filling, there are two occupied bands with the Bloch eigenstates $|u^n(\boldsymbol{k})\rangle$, $n=1,2$. At the four time-reversal-invariant momenta (TRIM points) of the 2D BZ shown in SFig. 13 ***a*** (*i.e.* the k points for which $\mathcal{T}\bar{\boldsymbol{k}}=\bar{\boldsymbol{k}}$ mod $\mathbf{b}$, where $\mathbf{b}$ is a reciprocal lattice vector), we can define the parity eigenvalue $\lambda_{\bar{\boldsymbol{k}}}$ of the occupied Kramers pair as:

$$\mathcal{I}\left|u^1(\bar{\boldsymbol{k}})\right\rangle=\lambda_{\bar{\boldsymbol{k}}}\left|u^1(\bar{\boldsymbol{k}})\right\rangle, \tag{161}$$

where $\mathcal{I}^2=1$ implies that $\lambda_{\bar{\boldsymbol{k}}}=\pm1$. At each TRIM point, states appear in Kramers pairs with the same parity ($\mathcal{I}$) eigenvalues, because the eigenvalues of $\mathcal{I}$ are real ($\lambda_{\bar{\boldsymbol{k}}}=\pm1$), and because the matrix representatives of $\mathcal{I}$ and $\mathcal{T}$ commute at the TRIM points [SEq. (160)].

For $\alpha=\beta=0$ and $\xi=0,1$, SEq. (159) is topologically trivial, and its occupied subspace is composed of two bands with the same parity eigenvalues $\lambda_{\bar{\boldsymbol{k}}}=1$ at all TRIM points. For $\xi=1$, $\mathcal{H}(\boldsymbol{k})$ describes a symmetry-indicated

2D TI that differs from a trivial (unobstructed) atomic limit by a single sign-change of the parity eigenvalue of the occupied Kramers pair of bands at $\bar{\boldsymbol{k}} = (1+\alpha, 1+\beta)\pi/2$. Specifically, the $\mathbb{Z}_2$ TI (Fu-Kane) invariant is indicated by the product of the parity eigenvalues of the occupied Kramers pairs [8, 10]. For $\xi = 0$, $\mathcal{H}(\boldsymbol{k})$ becomes equivalent to an array of identical x-directed $\mathcal{T}$-symmetric SSH chains indexed by k_y whose time-reversal polarization is indicated by the parity eigenvalues at $k_x = 0, \pi$ [6, 215–218, 246, 258, 261].

Having established the properties of the Hamiltonian of the pristine system [SEq. (159)], we will now probe the dislocation response. We introduce a pair of 0D dislocations with Burgers vector $\boldsymbol{B} = \hat{y}$. Through SEq. (83), we deduce that each dislocation realizes an interface between two insulators S and S' that "differ" by the Hamiltonian of the $k_y = \pi$ plane of SEq. (159):

$$\mathcal{H}(k_x, \pi) = \sin k_x \, \tau^0 \sigma^x + [\alpha \cos k_x + \xi(1-\beta)]\tau^z \sigma^z. \tag{162}$$

Setting $\alpha = -1$, $\mathcal{H}(k_x, \pi)$ describes an $\mathcal{I}$- and $\mathcal{T}$-symmetric SSH chain with a trivial (nontrivial) time-reversal polarization for $\xi = 1$, $\beta = -1$ ($\xi = 1$, $\beta = +1$).

To obtain the numerical results presented in SFig. 15, we use the same prescription as employed in SN 4 A 1 to obtain a Hamiltonian $\tilde{H}$ that differs from the pristine 2D Hamiltonian $\mathcal{H}(\boldsymbol{k})$ by the presence of a pair of point dislocations. With $L = 16$, $\tilde{H}$ has the dimensions $[4(L^2-8) \times 4(L^2-8)] = (992 \times 992)$. We will now discuss the electronic structure of $\tilde{H}$ in detail for characteristic values of ξ and β.

a. $\xi = 1, \beta = -1$: Topological insulator with band inversion at Γ This case corresponds to an inversion-symmetry-indicated 2D TI driven by band inversion at $\boldsymbol{k} = (0,0)$ in SEq. (159). In this case, the bulk characterizes a strong topological phase. Because the bands at X, Y, and M are uninverted relative to an atomic insulator with orbitals at the $1a$ position, the time-reversal generalization of the weak SSH invariant $\boldsymbol{M}_{\nu}^{\text{SSH}} = (0,0)$ (SN 3 A). This implies that $\boldsymbol{B} \cdot \boldsymbol{M}_{\nu}^{\text{SSH}} \bmod 2\pi = 0$. In SFig. 15 ***a***, ***d***, we respectively show the parity eigenvalues of the occupied bands and the dislocation spectrum, which does not exhibit a filling anomaly or midgap dislocation bound states.

b. $\xi = 1$, $\beta = +1$: Topological insulator with band inversion at Y This case corresponds to an inversion-symmetry-indicated 2D TI driven by band inversion at the Y point (SFig. 13 ***a***). Relative to the initial $1a$ atomic insulator, the band inversion at Y has changed not only the strong index (the $\mathbb{Z}_2$ TI invariant), but also the weak SSH indices [the parity eigenvalues of the occupied bands are shown in SFig. 15 ***b***]. Specifically, the bulk exhibits a weak SSH invariant $\boldsymbol{M}_{\nu}^{\text{SSH}} = (0, \pi)$ as defined in SN 3 A. This implies that $\boldsymbol{B} \cdot \boldsymbol{M}_{\nu}^{\text{SSH}} \bmod 2\pi = \pi$, indicating that the dislocation response is nontrivial. Correspondingly, in the dislocation spectrum (SFig. 15 ***e***), we observe one midgap-localized Kramers pair of zero modes per dislocation. The zero modes are protected by $\mathcal{T}$ symmetry and the chiral (*i.e.*, unitary particle-hole) symmetry Π, which is defined through the action:

$$\Pi : \; \mathcal{H}(k_x, \pi) \to \tau^0 \sigma^y \mathcal{H}(k_x, \pi) \tau^0 \sigma^y, \tag{163}$$

such that Π is a symmetry of $\mathcal{H}(k_x, \pi)$ if $\Pi \mathcal{H}(k_x, \pi) \Pi^{-1} = \tau^0 \sigma^y \mathcal{H}(k_x, \pi) \tau^0 \sigma^y = -\mathcal{H}(k_x, \pi)$. Crucially, if we were to relax Π symmetry, then the midgap states could be pushed out of the gap. However, if we preserve $\mathcal{I}$ and $\mathcal{T}$ symmetries while breaking Π, then chargeless spin would still remain bound to the dislocations, in a generalization of the conclusions of Goldstone and Wilczek [212–214] and SRefs. 164–166, 169, 170, and 279 (SN 2 A).

c. $\xi = 0$: $\mathcal{T}$-symmetric weak SSH array In this case, $\mathcal{H}(\boldsymbol{k})$ is independent of β, and SEq. (159) describes a y-directed array of identical x-directed $\mathcal{T}$-symmetric SSH chains [278]. Specifically, the Hamiltonian [SEq. (159) with $\xi = 0$] is in this case completely independent of k_y. For each SSH chain, the nontrivial time-reversal polarization is indicated by the difference in the parity eigenvalues of the occupied bands at $k_x = 0, \pi$ [6, 215–218, 246, 258, 261]. This case is an example of weak topology, because the Hamiltonian $\mathcal{H}(\boldsymbol{k})$ can be deformed into a real-space array of decoupled 1D chains without breaking a symmetry or closing the bulk gap. Relative to the initial $1a$ atomic insulator, $\mathcal{H}(\boldsymbol{k})$ features band inversions at Γ and Y (the parity eigenvalues are shown in SFig. 15 ***c***), such that the bulk exhibits a trivial symmetry-indicated $\mathbb{Z}_2$ TI invariant and nontrivial weak SSH indices, and is irrep-equivalent to an OAL from $1a$ $[(x,y) = (0,0)]$ to $1c$ $[((x,y) = (1/2, 0)]$ (SFig. 11 ***b***). Specifically, the bulk exhibits a weak SSH invariant $\boldsymbol{M}_{\nu}^{\text{SSH}} = (0, \pi)$ as defined in SN 3 A. This implies that, even though the bulk is an OAL (SN 3 A), the dislocation response is nontrivial. Correspondingly, in the dislocation spectrum (SFig. 15 ***e***), we observe a filling anomaly.

For the present case of an array of SSH chains, we can also understand the existence of dislocation midgap states intuitively without invoking the more general theory used to derive SEq. (162). The dislocations introduce an uncoupled SSH chain into the system whose end states become the dislocation bound states. As long as the "leftover" SSH chain is $\mathcal{T}$-symmetric and contains an inversion center, its end states also induce a system filling anomaly, consistent with the numerical results shown in SFig. 15 ***e***.

2. 3D Screw Dislocations in the Presence of $\mathcal{T}$ Symmetry

In this section, we will demonstrate the screw dislocation response of 3D insulators with $\mathcal{T}$ symmetry. We will here focus on higher-order TIs and weak FTIs, which, as we will numerically demonstrate, exhibit 0D dislocation bound states. However, we have also verified that our numerical models exhibit helical modes bound to screw dislocations inserted into insulators with nontrivial weak TI indices, in agreement with the results of SRefs. 71, 72, 75–77, and 80. Specifically, we observe that when the vector of weak TI indices in a $\mathcal{T}$-invariant 3D insulator $\boldsymbol{M}_\nu$ satisfies $\boldsymbol{B}\cdot\boldsymbol{M}_\nu \text{ mod } 2\pi = \pi$, the plane spanning the screw dislocations in SEq. (83) corresponds to 2D TI with 1D helical edge states that coincide with the bulk screw dislocations and boundary step edges. For the remainder of this section and work, we will restrict focus to $\mathcal{T}$-invariant 3D insulators for which line-like dislocations bind 0D (HEND) states (*i.e.* non-axionic HOTIs and weak FTIs/OALs).

While we will focus in this section on the topological response of 3D insulators to screw dislocations, we note that we can also consider $\mathcal{I}$-symmetric arrangements of edge dislocations. As shown in SN 2 A 1, when the HEND-state weak index vector $\boldsymbol{M}_\nu^{\text{F}}$ introduced in this work is nontrivial, an edge dislocation with a Burgers vector $\boldsymbol{B}$ will also bind an anomalous number of 0D states if $\boldsymbol{B}\cdot\boldsymbol{M}_\nu^{\text{F}} \text{ mod } 2\pi = \pi$. Later, in SN 6 B, we will use edge dislocations to demonstrate the nontrivial HEND-state defect response of 3D SnTe crystals (SFig. 24 ***a***).

We begin by comparing the minimal tight-binding model for a non-axionic higher-order topological insulator (HOTI) [30–33, 96, 97, 100, 101, 106–110, 172, 173, 175] to that of a stack of 2D $\mathcal{T}$-symmetric fragile topological insulators ($\mathcal{T}$-symmetric FTIs) [33, 100], where we retain $\mathcal{I}$ and $\mathcal{T}$ symmetries for all models. Consider the Bloch Hamiltonian:

$$\begin{aligned}\mathcal{H}(\boldsymbol{k}) = &\sin k_x\, \rho^z\tau^z\sigma^x + \sin k_y\, \rho^z\tau^z\sigma^y + \xi\sin k_z\, \rho^z\tau^z\sigma^z \\ &+ \left[\alpha\cos k_x + \beta\cos k_y + \xi\,(2+\gamma\cos k_z)\right]\rho^z\tau^x\sigma^0 \\ &+ \xi\,\delta\,(\rho^y\tau^z\sigma^x + \rho^y\tau^z\sigma^y + \rho^y\tau^z\sigma^z),\end{aligned} \tag{164}$$

defined on a square lattice, where ρ^i, τ^i and σ^i, $i = 0, x, y, z$ are three sets of Pauli matrices indexing sublattice and orbital degrees of freedom, respectively, and $\xi = 0, 1$ and $\alpha, \beta, \gamma = \pm 1$ are parameters that can be tuned to realize different topological phases, while δ is a small parameter that gaps out the helical states along dislocations (we will use $\delta = 1/4$ in all calculations, for $\delta = 0$ the Hamiltonian is a model of two uncoupled 3D TIs [104]). We abbreviate the Kronecker product by $\rho^i\otimes\tau^j\otimes\sigma^k \equiv \rho^i\tau^j\sigma^k$. Time-reversal ($\mathcal{T}$) and inversion ($\mathcal{I}$) symmetries are respectively represented by:

$$\begin{aligned}\mathcal{T}:\ \mathcal{H}(\boldsymbol{k}) &\to \rho^0\tau^0\sigma^y\mathcal{H}^*(-\boldsymbol{k})\rho^0\tau^0\sigma^y, \\ \mathcal{I}:\ \mathcal{H}(\boldsymbol{k}) &\to \rho^z\tau^x\sigma^0\mathcal{H}(-\boldsymbol{k})\rho^z\tau^x\sigma^0.\end{aligned} \tag{165}$$

At the eight TRIM points of the 3D BZ shown in SFig. 14 ***a***, we can again compute the parity eigenvalues $\lambda^n_{\bar{\boldsymbol{k}}}$, $n = 1, 2$ of the two occupied Kramers pairs of bands, where $\lambda^n_{\bar{\boldsymbol{k}}} = \pm 1$. For $\alpha = \beta = \gamma = 0$ and $\xi = 0, 1$, SEq. (164) is topologically trivial and its occupied subspace is composed of four bands with the parity eigenvalues $\lambda_{\bar{\boldsymbol{k}}} = 1$ at all TRIM points. For $\xi = 1$, $\mathcal{H}(\boldsymbol{k})$ describes a symmetry-indicated HOTI [100, 103–105] driven by a sign-change of the parity eigenvalues of the two occupied Kramers pairs of bands at $\bar{\boldsymbol{k}} = (1+\alpha, 1+\beta, 1+\gamma)\pi/2$. For $\xi = 0$, $\mathcal{H}(\boldsymbol{k})$ becomes equivalent to a z-directed stack of identical $\mathcal{I}$-symmetry-indicated [33, 97, 106–110, 155, 172–175] $\mathcal{T}$-symmetric FTIs indexed by k_z.

Having established the properties of the Hamiltonian of the pristine system [SEq. (164)], we will now probe its dislocation response. We introduce a pair of 1D screw dislocations with Burgers vector $\boldsymbol{B} = \hat{z}$ and opposite chiralities [SEq. (42)]. Through SEq. (83), we deduce that each dislocation realizes an interface between two insulators S and S' that "differ" by the Hamiltonian of the $k_z = \pi$ plane of SEq. (164):

$$\begin{aligned}\mathcal{H}(k_x, k_y, \pi) = &\sin k_x\, \rho^0\tau^z\sigma^x + \sin k_y\, \rho^0\tau^z\sigma^y \\ &+ \left[\alpha\cos k_x + \beta\cos k_y + \xi(2-\gamma)\right]\rho^z\tau^x\sigma^0 \\ &+ \xi\,\delta\,(\rho^z\tau^x\sigma^x + \rho^z\tau^x\sigma^y + \rho^z\tau^x\sigma^z).\end{aligned} \tag{166}$$

Setting $\alpha = \beta = -1$, $\mathcal{H}(k_x, k_y, \pi)$ describes a 2D trivial insulator ($\mathcal{T}$-symmetric FTI [33, 100]) for $\xi = 1$, $\gamma = -1$ ($\xi = 1$, $\gamma = +1$, $\delta = 1/4$). We will now discuss the electronic structure of $\tilde{H}(k_z)$ in detail for characteristic values of ξ and γ (SFig. 16). To obtain the numerical results presented in SFig. 16, we use the same prescription as employed in SN 4 A 2 [specifically SEqs. (154)-(157)] to obtain a Hamiltonian $\tilde{H}(k_z)$ that differs from a pristine crystal by a pair of screw dislocations.

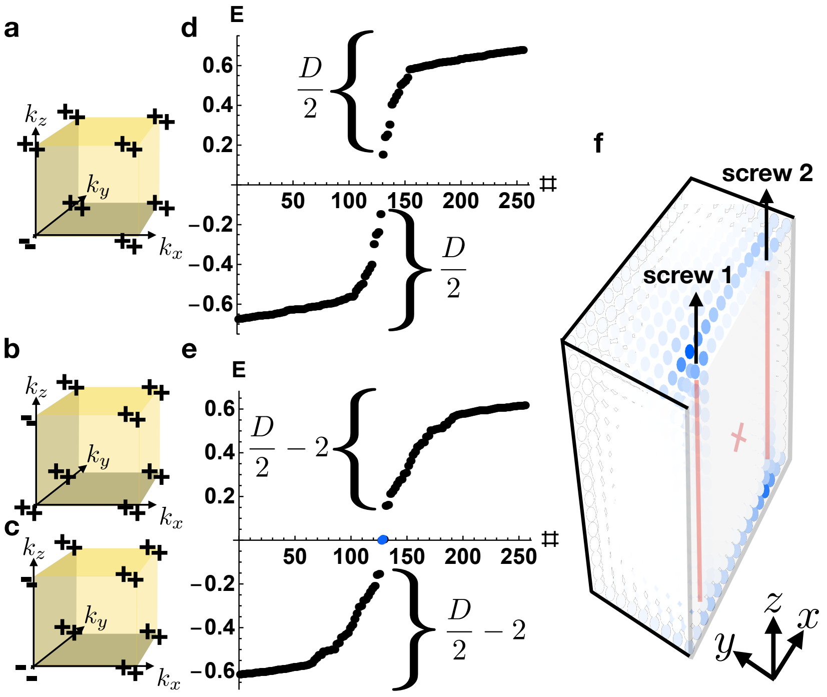

Supplementary Figure 16. **0D screw dislocation bound states in 3D insulators with time-reversal and inversion symmetry.** ***a*-*e*** Bulk parity [inversion ($\mathcal{I}$)] eigenvalues and hollow-doughnut-boundary-condition (HDBC) energy spectra for a pair of screw dislocations (SFig. 14 ***b***) inserted into ***a***, ***d*** an $\mathcal{I}$- and time-reversal- ($\mathcal{T}$-) symmetric HOTI with double band inversion at Γ [$\boldsymbol{M}_{\nu}^{\mathrm{F}} = (0,0,0)$ as defined in SN 3 B], ***b***, ***e*** a HOTI with double band inversion at Z [$\boldsymbol{M}_{\nu}^{\mathrm{F}} = (0,0,\pi)$], and ***c***, ***e*** a weak stack of $\mathcal{I}$- and $\mathcal{T}$-symmetric 2D FTIs [$\boldsymbol{M}_{\nu}^{\mathrm{F}} = (0,0,\pi)$]. In ***a***, ***b***, ***c***, Kramers pairs of parity eigenvalues are denoted with a single $\pm$ symbol. Anomalous 0D HEND states with spin-charge separation (SN 2 A) are present on the dislocation ends in ***b*** and ***c***, but not in ***a***. The 0D (HEND) states in ***b*** and ***c*** are equivalent to the corner states of an $\mathcal{I}$- and $\mathcal{T}$-symmetric 2D FTI [33, 100], and thus persist under the relaxation of particle-hole symmetry [33, 70, 100]. In ***d***, ***e***, we only show the 256 lowest-lying states. In the presence of an additional chiral symmetry, the two midgap Kramers pairs of dislocation end states in ***b*** and ***c*** are pinned to zero energy. In the absence of chiral symmetry, a nontrivial dislocation response can still be diagnosed by observing filling anomaly [33, 70, 141–143, 176]. ***f*** Real-space-resolved absolute square of the midgap eigenstates shown in ***e*** on a $16 \times 16 \times 16$ lattice (cut in half along the transparent gray plane to expose the bound states). $\mathcal{I}$-related Kramers pairs of bound states appear on two of the four screw dislocation ends. The inversion center in ***f*** is marked with a red X. As derived in SEq. (83), the dislocation response in ***a*** – ***c*** is nontrivial when $\boldsymbol{B} \cdot \boldsymbol{M}_{\nu}^{\mathrm{F}} \bmod 2\pi = \pi$.

a. $\xi = 1$, $\gamma = -1$: Higher-order topological insulator with double band inversion at Γ This case corresponds to an inversion-symmetry-indicated HOTI driven by two Kramers pairs of band inversions at $\boldsymbol{k} = (0,0,0)$ in SEq. (164). In this case, the bulk characterizes a strong topological phase. Because the bands at X, Y, M, Z, U, R, and T are uninverted relative to an atomic insulator with orbitals at the $1a$ position, the weak fragile invariant $\boldsymbol{M}_{\nu}^{\mathrm{F}} = (0,0,0)$ (SN 3 B). This implies that $\boldsymbol{B} \cdot \boldsymbol{M}_{\nu}^{\mathrm{F}} \bmod 2\pi = 0$, and therefore, that the dislocations do not bind anomalous Kramers pairs of spin-charge-separated HEND states. In SFig. 16 ***a***, ***d***, we respectively show the parity eigenvalues of the occupied bands and the dislocation spectrum, which does not exhibit a filling anomaly or midgap dislocation bound states.

b. $\xi = 1$, $\gamma = +1$: Higher-order topological insulator with double band inversion at Z This case describes a HOTI driven by double band inversion at the Z point (SFig. 14 ***a***). Relative to a $1a$ trivial (unobstructed) atomic limit, the double band inversion at Z has changed not only the strong $\mathbb{Z}_4$ HOTI index [100, 103–105], but also the weak fragile indices [the parity eigenvalues of the occupied bands are shown in SFig. 16 ***b***]. Specifically, the bulk exhibits a nontrivial weak fragile index $\boldsymbol{M}_{\nu}^{\mathrm{F}} = (0,0,\pi)$ as defined in SN 3 B. This implies that $\boldsymbol{B} \cdot \boldsymbol{M}_{\nu}^{\mathrm{F}} \bmod 2\pi = \pi$, indicating that the dislocation response is nontrivial. Correspondingly, in the dislocation spectrum (SFig. 16 ***e***), we observe one midgap-localized Kramers pair of modes per dislocation. The zero modes are protected by the chiral (*i.e.* unitary particle-hole) symmetry Π, which is defined through the action:

$$\Pi : \ \mathcal{H}(k_x, k_y, \pi) \rightarrow \rho^0 \tau^y \sigma^0 \mathcal{H}(k_x, k_y, \pi) \rho^0 \tau^y \sigma^0, \tag{167}$$

such that Π is a symmetry of $\mathcal{H}(k_x,k_y,\pi)$ if $\Pi\mathcal{H}(k_x,k_y,\pi)\Pi^{-1}=\rho^0\tau^y\sigma^0\mathcal{H}(k_x,k_y,\pi)\rho^0\tau^y\sigma^0=-\mathcal{H}(k_x,k_y,\pi)$. Crucially, if we were to relax Π symmetry, the midgap states could be pushed out of the gap. Equivalently, in the absence of chiral symmetry, we can also identify the nontrivial topology by counting the number of states that are occupied up to a given Fermi level in the gap [33, 70, 141–143, 176], just as we did in SN 4 B 1 b. Let the 3D real-space Hamiltonian corresponding to the system with two screw dislocations be a $(D\times D)$ matrix. Comparing SFigs. 16 ***d*** and ***e***, we observe that the spectra differ by the presence of two Kramers pairs of midgap states and the absence of two states from each of the valence and conduction manifolds. When chiral symmetry is broken while preserving $\mathcal{I}$ and $\mathcal{T}$ symmetries, then the energy of the midgap states can be shifted, but only in a manner that preserves $\mathcal{I}$ and $\mathcal{T}$, leaving the four states degenerate in the thermodynamic limit. This implies that any gapped Fermi level that encloses either $(D/2-2)$ or $(D/2+2)$ occupied states indicates a nontrivial dislocation response, whereas a Fermi level enclosing $D/2$ occupied states indicates a trivial screw dislocation response.

c. $\xi=0$*: Weak* $\mathcal{T}$*-symmetric FTI stack* In this case, $\mathcal{H}(\boldsymbol{k})$ is independent of γ, and SEq. (164) describes a z-directed stack of identical 2D $\mathcal{T}$-symmetric FTIs. Specifically, the Hamiltonian [SEq. (164) with $\xi=0$] is in this case completely independent of k_z. This is an example of weak topology, because the Hamiltonian $\mathcal{H}(\boldsymbol{k})$ can be deformed into a real-space stack of decoupled $\mathcal{I}$- and $\mathcal{T}$-symmetric 2D FTIs without breaking a symmetry or closing the bulk gap. Relative to the initial trivial atomic insulator, $\mathcal{H}(\boldsymbol{k})$ features double band inversions at Γ and Z (the parity eigenvalues are shown in SFig. 16 ***c***), such that the bulk exhibits a nontrivial weak fragile invariant $\boldsymbol{M}^{\mathrm{F}}_{\nu}=(0,0,\pi)$ as defined in SN 3 A. This implies that, even though the bulk is Wannierizable (after the addition of trivial bands, see SN 3 B), the dislocation response is nontrivial. Correspondingly, in the dislocation spectrum (SFig. 16 ***e***), we observe a filling anomaly.

For the present case of an array of FTI planes, we can also understand the existence of dislocation midgap states intuitively without invoking the more general theory used to derive SEq. (166). Specifically, if we were to instead insert a loop of edge dislocations with Burgers vector $\boldsymbol{B}=\hat{z}$, then we would effectively introduce an uncoupled $\mathcal{I}$- and $\mathcal{T}$-symmetric FTI into the system whose corner states become dislocation end states. As long as the "leftover" FTI plane contains an inversion center, then its corner states will also induce a system filling anomaly, consistent with the numerical results shown in SFig. 16 ***e***.

5. NUMERICAL CALCULATION DETAILS: FLUXES AND FLUX TUBES

A. Flux Response without Time-Reversal Symmetry

1. 2D Fluxes in the Absence of $\mathcal{T}$ Symmetry

In this section, we will demonstrate the π-flux response of $\mathcal{I}$-symmetric 2D insulators with broken $\mathcal{T}$ symmetry [72, 75, 76, 164–166, 169, 170, 183]. We begin by considering a pristine 2D insulator described by the Bloch Hamiltonian $\mathcal{H}(\boldsymbol{k})$ in SEq. (139) of SN 4 A 1. We then numerically introduce a pair of π-fluxes related by a bulk $\mathcal{I}$ center as discussed in SN 2 B 2.

To obtain the numerical results presented in SFig. 17, we begin with a square lattice Λ of size $|\Lambda|=L^2$ with $L=16$ and PBC to model the (x,y)-plane. We then modify the y-directed hoppings crossing a line of length 8 at fixed (but necessarily fractional) y coordinate to create a pair of π-fluxes, where two is the minimal number of fluxes compatible with (untwisted) PBC. We define H and $H_{A,B}|A\rangle\langle B|$ using the same notation employed in SN 4 A 1. Using the site labeling U and M established in SFig. 5 ***a***, we will now detail how we numerically implement a pair of π-fluxes.

Defining $S=U\cup M$ and $\bar{S}=\Lambda\backslash S$, we form the expression:

$$H=H_{\Lambda,\Lambda}|\Lambda\rangle\langle\Lambda| = H_{\bar{S},\bar{S}}|\bar{S}\rangle\langle\bar{S}|+H_{S,\bar{S}}|S\rangle\langle\bar{S}| + H_{\bar{S},S}|\bar{S}\rangle\langle S|+H_{S,S}|S\rangle\langle S|. \tag{168}$$

We then expand SEq. (168):

$$H_{S,S}|S\rangle\langle S| = H_{U,U}|U\rangle\langle U|+H_{M,M}|M\rangle\langle M| + H_{U,M}|U\rangle\langle M|+H_{M,U}|M\rangle\langle U|. \tag{169}$$

To introduce a pair of π-fluxes, we form the Hamiltonian $\tilde{H}$, which is defined on Λ, and can be expanded as:

$$\tilde{H}=\tilde{H}_{\Lambda,\Lambda}|\Lambda\rangle\langle\Lambda| = H_{\bar{S},\bar{S}}|\bar{S}\rangle\langle\bar{S}|+H_{S,\bar{S}}|S\rangle\langle\bar{S}| + H_{\bar{S},S}|\bar{S}\rangle\langle S|+\tilde{H}_{S,S}|S\rangle\langle S|, \tag{170}$$

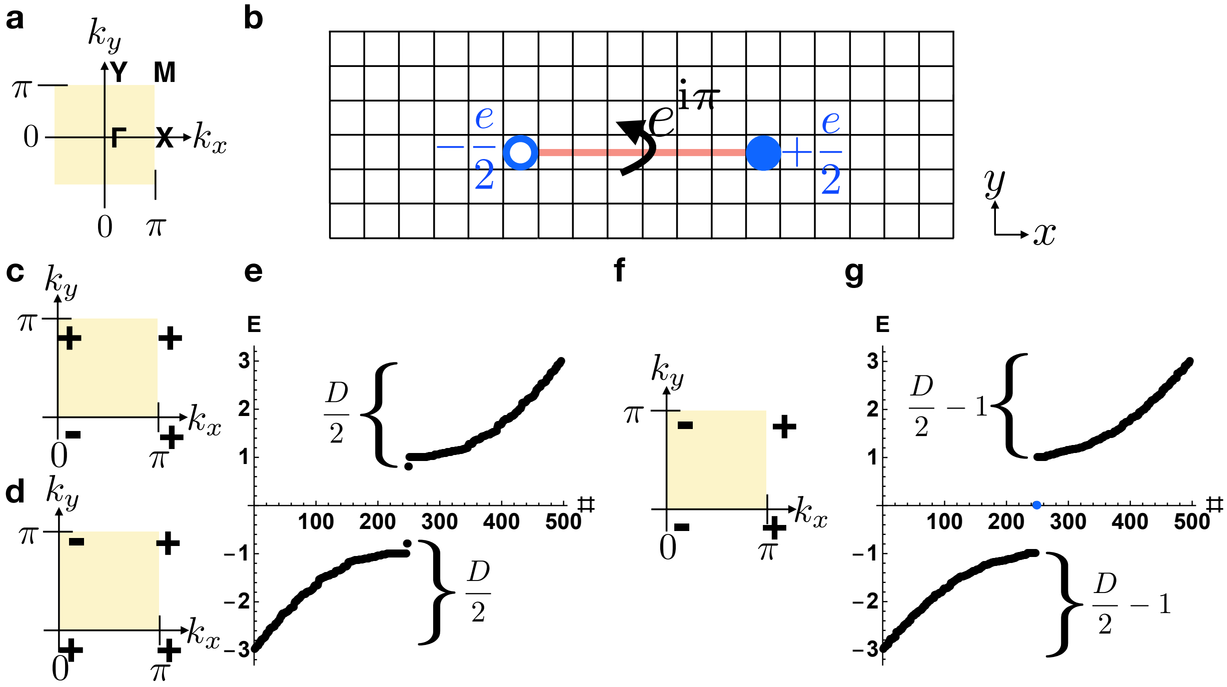

Supplementary Figure 17. **0D π-flux bound states in 2D insulators with inversion symmetry.** ***a*** The bulk BZ of a 2D rectangular magnetic crystal with only inversion ($\mathcal{I}$) symmetry. ***b*** We implement a pair of 0D π-fluxes related by $\mathcal{I}$ by multiplying all of the hoppings along the red line of sites by $e^{\mathrm{i}\pi}$ in the positive y direction, and by multiplying all of the hoppings along the same line by $e^{-\mathrm{i}\pi}$ in the negative y direction. ***c*** – ***g*** Bulk parity ($\mathcal{I}$) eigenvalues and PBC energy spectra for the flux in ***b*** when the crystal with the BZ in ***a*** is equivalent to ***c***, ***g*** a $|C| = 1$ Chern insulator with band inversion at Γ, ***d***, ***g*** a $|C| = 1$ Chern insulator with band inversion at Y, and ***f***, ***e*** a weak y-directed array of SSH chains [215, 216]. Anomalous 0D flux states with charge $\pm e/2$ are present in cases ***c*** and ***d***, but not in ***f***, and are equivalent to the end states of an $\mathcal{I}$-symmetric, $\mathcal{T}$-broken SSH chain [215, 216] [SEq. (142), red line in ***b***], and thus persist under the relaxation of particle-hole symmetry [33, 70, 212–214].

in which we can further expand:

$$\tilde{H}_{S,S}|S\rangle\langle S| = H_{U,U}|U\rangle\langle U| + H_{M,M}|M\rangle\langle M| + e^{-\mathrm{i}\pi}H_{U,M}|U\rangle\langle M| + e^{\mathrm{i}\pi}H_{M,U}|M\rangle\langle U|, \tag{171}$$

where the exponential factors $e^{\pm \mathrm{i}\pi}$ implement the π-fluxes. To summarize, SEqs. 168 through 171 describe how, in a tight-binding model with nearest-neighbor hoppings and orthogonal lattice vectors, we numerically implement a pair of π-fluxes by multiplying all of the hoppings across a line between sites in a pristine lattice by the phase factors $e^{\pm \mathrm{i}\pi}$.

We will now discuss the electronic structure of $\tilde{H}$ in detail for characteristic values of ξ and β, setting $\alpha = -1$ for all of the cases discussed in this section.

a. $\xi = 1$, $\beta = -1$: Chern insulator with band inversion at Γ In this case, we consider the system response to a pair of π-fluxes spatially separated in the x direction. As shown in SN 2 B 2, the π-flux response is given in this case by the summed topologies of the $k_y = 0, \pi$ lines in the 2D BZ of $\mathcal{H}(\boldsymbol{k})$ (which can be summarized as the bulk Chern number C mod 2). In the pristine insulating bulk, the $k_y = 0$ ($k_y = \pi$) line characterizes a nontrivial (trivial) SSH chain [SEq. (142)], and the bulk correspondingly exhibits an inversion-symmetry-indicated Chern number $|C| = 1$ [13]. We observe a single midgap state at each π-flux core, which can be pinned to zero energy by the chiral symmetry in SEq. (149) or diagnosed via a filling anomaly [33, 70, 141–143, 176]. The bulk parity eigenvalues and the flux spectrum are respectively shown in SFig. 17 ***c***, ***g***. The dislocation response of a $|C| = 1$ Chern insulator driven by band inversion at Γ was previously discussed in detail in SN 4 A 1 a – further details of our numerical implementation are provided in that section.

b. $\xi = 1$, $\beta = +1$: Chern insulator with band inversion at Y As in the previous case of a $|C| = 1$ Chern insulator driven by band inversion at Γ, because the summed topology of the $k_y = 0, \pi$ lines in the 2D BZ of $\mathcal{H}(\boldsymbol{k})$ is again nontrivial (and, equivalently, because the bulk Chern number is odd), then we observe a nontrivial flux response. This underlines the fact that, in contrast to the dislocation response, the π-flux response of 2D and 3D insulators only depends on the strong topological index (SN 2 A 3 and 2 B 2), and in particular does not depend on where in the BZ

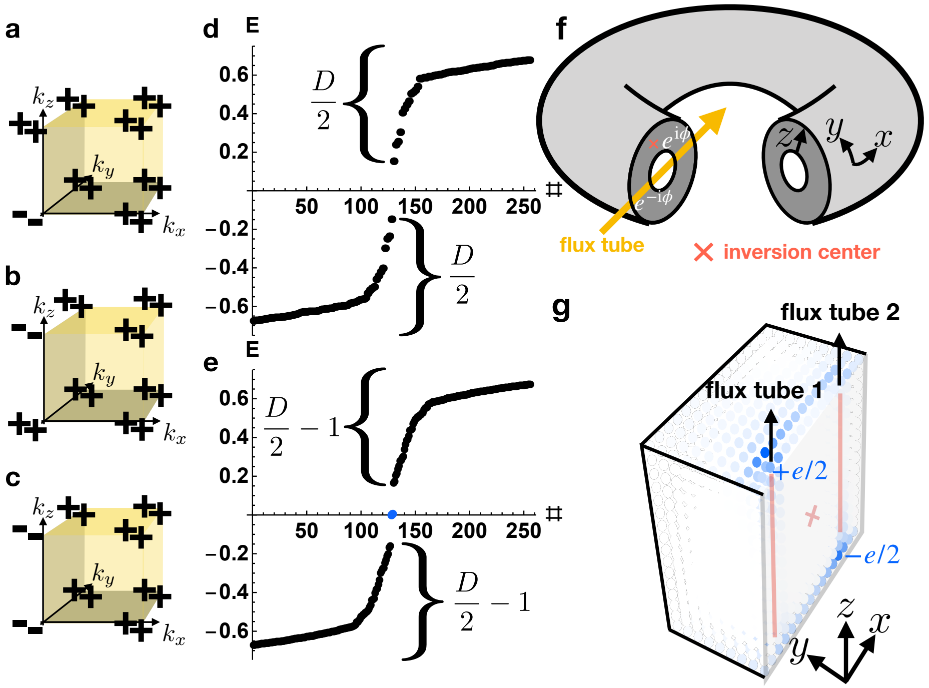

Supplementary Figure 18. **0D π-flux bound states in 3D insulators with inversion symmetry.** ***a*** – ***e*** Bulk parity eigenvalues and HDBC energy spectra for the flux in SFig. 17 ***b***, extended along the z direction, when the crystal with the BZ in SFig. 14 ***a*** is equivalent to ***a***, ***e*** an $\mathcal{I}$-symmetric axion insulator (AXI) [30–33, 96, 97, 100, 101, 106–110, 172, 173, 175] with double band inversion at Γ, ***b***, ***e*** an AXI with double band inversion at Z, ***c***, ***d*** a weak stack of $\mathcal{I}$-symmetric 2D FTIs with $\pm e/2$ corner charges [33, 100]. Only the 256 lowest-lying states are shown in ***d***, ***e***. Anomalous 0D (HEND) states with charge $\pm e/2$ are present on $\mathcal{I}$-related flux tube ends in ***a*** and ***b***, but not in ***c***. The HEND states in ***a*** and ***b*** are equivalent to the corner states of an $\mathcal{I}$-symmetric, $\mathcal{T}$-broken 2D FTI [33, 100], and thus persist under the relaxation of particle-hole symmetry [33, 70, 100]. This is consistent with the conclusions of SN 2 A 3 and 2 B 2, in which it was determined that only topological phases with nontrivial strong indices exhibit a nontrivial π-flux response. ***f*** Hollow doughnut boundary conditions (HDBC) used to obtain the flux tube end states, which correspond to periodic boundary conditions in the x, y directions and open boundary conditions in z direction. Crucially, the HDBC geometry does not have any hinges, such that, absent π-flux tubes, trivial insulators and AXIs exhibit the same gapped energy spectra when terminated in an HDBC geometry. ***g*** Absolute square of the wavefunction of the midgap states in ***e*** on a $16 \times 16 \times 16$ lattice (cut in half along the transparent gray plane to expose the bound states, which appear on two of the four flux tube ends). The inversion center in ***g*** is marked with a red X. In ***g***, the bound state wavefunctions exhibit some residual localization on all four flux tube ends. However, there is no symmetry that relates the two flux tube ends on each surface (as opposed to flux tube ends on opposing tubes and surfaces, which are conversely related by bulk $\mathcal{I}$). Hence, in the presence of symmetry-allowed terms that break all artificial mirror reflection symmetries [100, 149], we expect that as the system size is increased, the two anomalous midgap states will more strongly localize on only two of the four flux tube ends.

bands are inverted. The bulk parity eigenvalues and the flux spectrum are respectively shown in SFig. 17 ***d***, ***g***. The dislocation response of a $|C| = 1$ Chern insulator driven by band inversion at Y was previously discussed in detail in SN 4 A 1 b – further details of our numerical implementation are provided in that section.

c. $\xi = 0$: Weak SSH array The bulk parity eigenvalues and the flux spectrum are shown in SFig. 17 ***f***, ***e***. Because the bulk carries a trivial Chern number, then the π-flux response is trivial. The dislocation response of a weak array of $\mathcal{I}$-symmetric, $\mathcal{T}$-broken SSH chains was previously discussed in detail in SN 4 A 1 c – further details of our numerical implementation are provided in that section.

2. 3D Flux Tubes in the Absence of $\mathcal{T}$ Symmetry

In this section, we will demonstrate the π-flux response of $\mathcal{I}$-symmetric 3D insulators with broken $\mathcal{T}$ symmetry [72, 75, 76, 164–166, 169, 170, 183]. We begin by considering a pristine 3D insulator described by the Bloch Hamiltonian

$\mathcal{H}(\boldsymbol{k})$ in SEq. (150) of SN 4 A 2. We then numerically introduce a pair of π-flux tubes related by a bulk $\mathcal{I}$ center as discussed in SN 2 B 2. Each tube corresponds to a π-flux in each real-space plane indexed by z, or equivalently, at each momentum k_z. As in SN 4 A 2, we set $\alpha = \beta = -1$ in SEq. (150). In our numerics, the flux tubes are implemented by following the procedure employed for the 2D insulators in SN 5 A 1 in each z-indexed, real-space plane of the 3D insulators examined in this section.

We will now discuss the electronic structure of the Hamiltonian with two π-flux tubes $\tilde{H}(k_z)$ in detail for representative values of ξ and γ. The numerical results are summarized in SFig. 18.

a. $\xi = 1$, $\gamma = -1$: Axion insulator with double band inversion at Γ In this case, we consider the system response to a pair of z-directed π-flux tubes that are spatially separated in the x direction. As shown in SN 2 B 2, the π-flux response is given in this case by the summed topologies of the $k_y = 0, \pi$ planes in the 3D BZ of $\mathcal{H}(\boldsymbol{k})$ (which can be summarized by the value of the strong AXI index [33]). In the pristine insulating bulk, the $k_y = 0$ ($k_y = \pi$) plane characterizes an $\mathcal{I}$-symmetric 2D FTI (trivial insulator), and the bulk correspondingly exhibits an inversion-symmetry-indicated nontrivial AXI index [30–33, 96, 97, 100, 101, 106–110, 155, 172–175]. We observe a single midgap state on two of four flux tube ends, which can be pinned to zero energy by the chiral symmetry in SEq. (158) or diagnosed via a filling anomaly in the absence of chiral symmetry [33, 70, 141–143, 176]. The bulk parity eigenvalues and the flux spectrum are respectively shown in SFig. 18 ***a***, ***e***. The dislocation response of an AXI with double band inversion at Γ was previously discussed in detail in SN 4 A 2 a – further details of our numerical implementation are provided in that section.

b. $\xi = 1$, $\gamma = +1$: Axion insulator with double band inversion at Z As in the previous case of an AXI driven by double band inversion at Γ, because the summed topology of the $k_y = 0, \pi$ planes in the 3D BZ of $\mathcal{H}(\boldsymbol{k})$ is again nontrivial (and, equivalently, because the bulk strong AXI index is nontrivial), then we observe a nontrivial flux response. This further underlines the fact that, in contrast to the dislocation response, the π-flux response of 2D and 3D insulators only depends on the strong topological index (SN 2 A 3 and 2 B 2), and in particular does not depend on where bands are inverted. The bulk parity eigenvalues and the flux spectrum are respectively shown in SFig. 18 ***b***, ***e***. The dislocation response of an AXI with double band inversion at Z was previously discussed in detail in SN 4 A 2 b – further details of our numerical implementation are provided in that section.

c. $\xi = 0$: Weak FTI stack The bulk parity eigenvalues and the flux spectrum are respectively shown in SFig. 18 ***c***, ***d***. Because the bulk carries a trivial strong (AXI) index, then the π-flux response is trivial. The dislocation response of a weak stack of $\mathcal{I}$-symmetric, $\mathcal{T}$-broken 2D FTIs was previously discussed in detail in SN 4 A 2 c – further details of our numerical implementation are provided in that section.

B. Flux Response with Time-Reversal Symmetry

1. 2D Fluxes in the Presence of $\mathcal{T}$ Symmetry

In this section, we will demonstrate the π-flux response of $\mathcal{I}$- and $\mathcal{T}$-symmetric 2D insulators [72, 75, 76, 164–166, 169, 170, 183]. We begin by considering a pristine 2D insulator described by the Bloch Hamiltonian $\mathcal{H}(\boldsymbol{k})$ in SEq. (159) of SN 4 B 1. We then numerically introduce a pair of π-fluxes related by a bulk $\mathcal{I}$ center as discussed in SN 2 B 2 (SFig. 19). To obtain the numerical results presented in SFig. 19, we use the same prescription as employed in SN 5 A 1 to create a pair of π-fluxes. We will now discuss the electronic structure of the resulting model $\tilde{H}$ in detail for $\alpha = -1$ and for representative values of ξ and β.

a. $\xi = 1$, $\beta = -1$: 2D topological insulator with band inversion at Γ In this case, we consider the system response to a pair of π-fluxes spatially separated in the x direction. As shown in SN 2 B 2, the π-flux response is given in this case by the summed topologies of the $k_y = 0, \pi$ lines in the 2D BZ of $\mathcal{H}(\boldsymbol{k})$ (which can be summarized as the bulk $\mathbb{Z}_2$ invariant [3, 5, 6]). In the pristine insulating bulk, the $k_y = 0$ ($k_y = \pi$) line characterizes a nontrivial (trivial) $\mathcal{T}$-symmetric SSH chain [SEq. (162)], and the bulk correspondingly exhibits an inversion-symmetry-indicated nontrivial 2D TI $\mathbb{Z}_2$ Fu-Kane index [7, 8]. We observe a Kramers pair of midgap states at each π-flux core, which can be pinned to zero energy by the chiral symmetry in SEq. (163) or diagnosed via a filling anomaly [33, 70, 141–143, 176]. The bulk parity eigenvalues and the flux spectrum are respectively shown in SFig. 19 ***a***, ***e***. The dislocation response of a 2D TI with band inversion at Γ was previously discussed in detail in SN 4 B 1 a – further details of our numerical implementation are provided in that section.

b. $\xi = 1$, $\beta = +1$: 2D topological insulator with band inversion at Y As in the previous case of a 2D TI driven by band inversion at Γ, because the summed topology of the $k_y = 0, \pi$ lines in the 2D BZ of $\mathcal{H}(\boldsymbol{k})$ is again nontrivial (and, equivalently, because the bulk 2D TI index is nontrivial), then we observe a nontrivial flux response. This further underlines the fact that, in contrast to the dislocation response, the π-flux response of 2D and 3D insulators only depends on the strong topological index (SN 2 A 3 and 2 B 2), and in particular does not depend on where bands are inverted. The bulk parity eigenvalues and the flux spectrum are respectively shown in SFig. 19 ***b***, ***e***. The dislocation

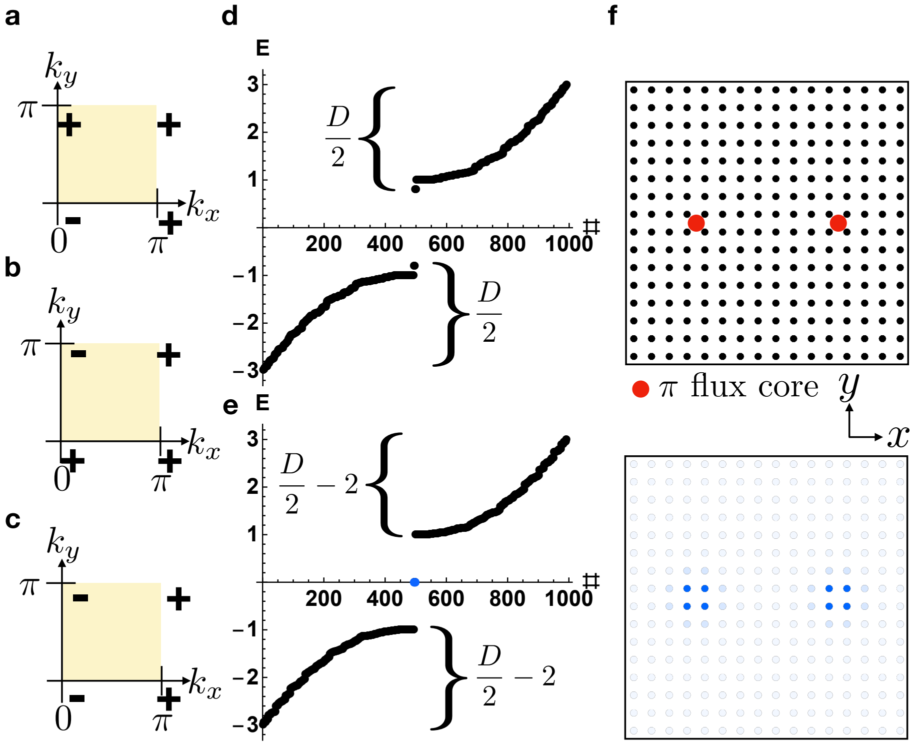

Supplementary Figure 19. **0D π-flux bound states in 2D insulators with time-reversal and inversion symmetry.** ***a*** – ***e*** Bulk parity eigenvalues and PBC energy spectra for the flux in SFig. 17 ***b*** when the crystal with the BZ in SFig. 17 ***a*** is equivalent to ***a***, ***e*** a 2D TI driven by band inversion at Γ, ***b***, ***e*** a 2D TI driven by band inversion at Y, and ***c***, ***d*** a weak y-directed array of $\mathcal{I}$- and $\mathcal{T}$-symmetric SSH chains [215–218, 258, 278]. In ***a*** – ***c***, we denote Kramers pairs of parity eigenvalues with a single ± sign. Anomalous 0D flux states with spin-charge separation [164–166, 169, 170] are present in cases ***a*** and ***b***, but not in ***c***, and are equivalent to the end states of an $\mathcal{I}$- and $\mathcal{T}$-symmetric SSH chain [215–218, 258]. With chiral symmetry, the two midgap Kramers pairs that are present in ***a*** and ***b*** are pinned to zero energy. In the absence of chiral symmetry, a nontrivial π-flux response can still be diagnosed by observing a filling anomaly [33, 70, 141–143, 176]. ***f*** Real-space geometry with a pair of π-flux cores and absolute square of the wavefunction of the midgap states in ***e*** on a 16×16 lattice.

response of a 2D TI with band inversion at Y was previously discussed in detail in SN 4 B 1 b – further details of our numerical implementation are provided in that section.

c. $\xi = 0$: Weak $\mathcal{T}$-symmetric SSH array The bulk parity eigenvalues and the flux spectrum are respectively shown in SFig. 19 ***c***, ***d***. Because the bulk carries a trivial 2D TI index, then the π-flux response is trivial. The dislocation response of a weak array of $\mathcal{I}$- and $\mathcal{T}$-symmetric SSH chains was previously discussed in detail in SN 4 B 1 c – further details of our numerical implementation are provided in that section.

2. 3D Flux Tubes in the Presence of $\mathcal{T}$ Symmetry

In this section, we will demonstrate the π-flux response of 3D insulators with $\mathcal{I}$ and $\mathcal{T}$ symmetry [72, 75, 76, 164–166, 169, 170, 183]. We begin by considering a pristine 3D insulator described by the Bloch Hamiltonian $\mathcal{H}(\boldsymbol{k})$ in SEq. (164) of SN 4 B 2. We then numerically introduce a pair of π-flux tubes related by a bulk $\mathcal{I}$ center as detailed in SN 5 A 2. As in SN 4 B 2, we set $\alpha = \beta = -1$ in SEq. (164). We will now discuss the electronic structure of the Hamiltonian with two π-flux tubes $\tilde{H}(k_z)$ in detail for representative values of ξ and γ. The numerical results are summarized in SFig. 20.

a. $\xi = 1$, $\gamma = -1$: Higher-order topological insulator with double band inversion at Γ In this case, we consider the system response to a pair of z-directed π-flux tubes that are spatially separated in the x direction. As shown in SN 2 B 2, the π-flux response is given in this case by the summed topologies of the $k_y = 0, \pi$ planes in the 3D BZ of $\mathcal{H}(\boldsymbol{k})$ (which can be summarized by the value of the strong $\mathbb{Z}_4$ HOTI index [33, 100]). In the pristine insulating

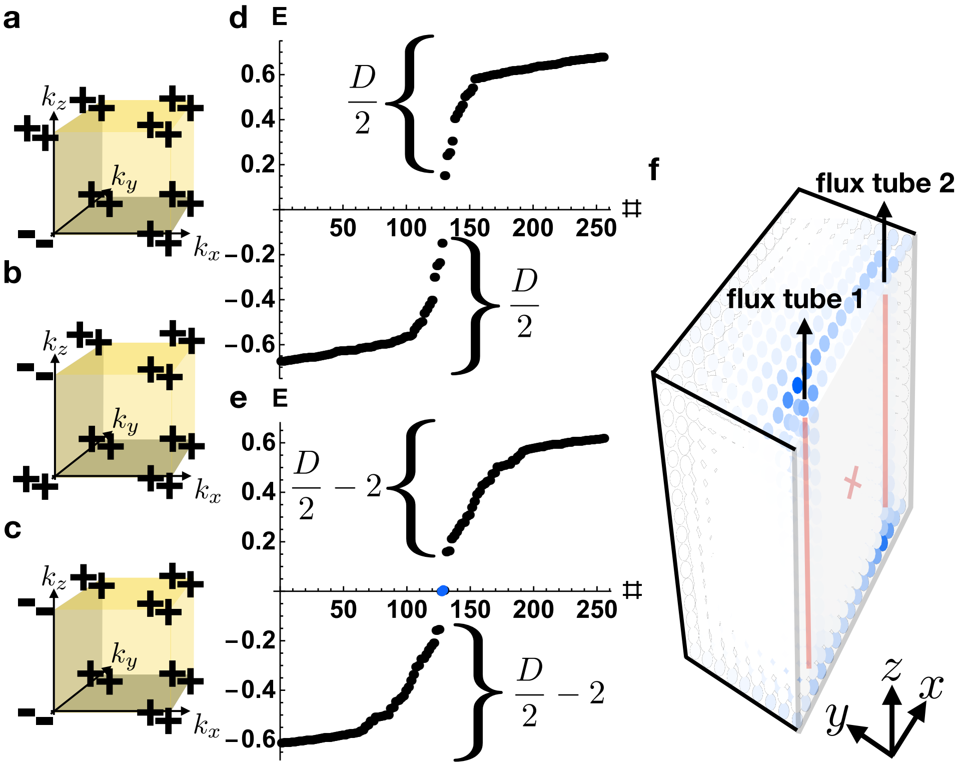

Supplementary Figure 20. **0D π-flux bound states in 3D insulators with time-reversal and inversion symmetry.** $\boldsymbol{a}-\boldsymbol{e}$ Bulk parity eigenvalues and HDBC energy spectra for the flux in SFig. 17 $\boldsymbol{b}$, extended along the z direction, when the crystal with the BZ shown in SFig. 14 $\boldsymbol{a}$ is equivalent to $\boldsymbol{a},\boldsymbol{e}$ an $\mathcal{I}$- and $\mathcal{T}$-symmetric HOTI driven by double band inversion at Γ, $\boldsymbol{b},\boldsymbol{e}$ a HOTI with double band inversion at Z, and $\boldsymbol{c},\boldsymbol{d}$ a weak stack of $\mathcal{I}$- and $\mathcal{T}$-symmetric 2D FTIs [100] with corner midgap Kramers pairs. In $\boldsymbol{a}-\boldsymbol{c}$, we denote Kramers pairs of parity eigenvalues with a single $\pm$ sign. Only the 256 lowest-lying states are shown in $\boldsymbol{d},\boldsymbol{e}$. Anomalous 0D (HEND) states with spin-charge separation [164–166, 169, 170] are present on $\mathcal{I}$-related flux tube ends in $\boldsymbol{a}$ and $\boldsymbol{b}$, but not in $\boldsymbol{c}$. The HEND states in $\boldsymbol{a}$ and $\boldsymbol{b}$ are equivalent to the corner states of an $\mathcal{I}$- and $\mathcal{T}$-symmetric 2D FTI [33, 100], and thus persist under the relaxation of particle-hole symmetry [33, 70, 100]. This is consistent with the conclusions of SN 2 A 3 and 2 B 2, in which it was determined that only topological phases with nontrivial strong indices exhibit a nontrivial π-flux response. With chiral symmetry, the two midgap Kramers pairs that are present in cases $\boldsymbol{a}$ and $\boldsymbol{b}$ are pinned to zero energy. In the absence of chiral symmetry, the π-flux-tube response can still be diagnosed as topologically nontrivial by observing a filling anomaly [33, 70, 141–143, 176]. $\boldsymbol{f}$ Absolute square of the midgap states in $\boldsymbol{e}$ on a $16\times16\times16$ lattice (cut in half along the transparent gray plane to expose the bound states, which appear on two of the four flux tube ends). The inversion center in $\boldsymbol{f}$ is marked with a red X. In $\boldsymbol{f}$, the bound state wavefunctions exhibit some residual localization on all four flux tube ends. However, there is no symmetry that relates the two flux tube ends on each surface (as opposed to flux tube ends on opposing tubes and surfaces, which are conversely related by bulk $\mathcal{I}$). Hence, in the presence of symmetry-allowed terms that break all artificial mirror reflection symmetries [100, 149], we expect that as the system size is increased, the two anomalous Kramers pairs of midgap states will more strongly localize on only two of the four flux tube ends.

bulk, the $k_y=0$ ($k_y=\pi$) plane characterizes an $\mathcal{I}$- and $\mathcal{T}$-symmetric 2D FTI (trivial insulator) [SEq. (166)], and the bulk correspondingly exhibits an inversion-symmetry-indicated nontrivial $\mathbb{Z}_4$ HOTI index [100, 103–105]. We observe a Kramers pair of midgap states on two of four flux tube ends, which can be pinned to zero energy by the chiral symmetry in SEq. (167) or diagnosed via a filling anomaly in the absence of chiral symmetry [33, 70, 141–143, 176]. The bulk parity eigenvalues and the flux spectrum are respectively shown in SFig. 20 $\boldsymbol{a},\boldsymbol{e}$. The dislocation response of an $\mathcal{I}$- and $\mathcal{T}$-symmetric HOTI with double band inversion at Γ was previously discussed in detail in SN 4 B 2 a – further details of our numerical implementation are provided in that section.

b. $\xi=1$, $\gamma=+1$: *HOTI with double band inversion at* Z As in the previous case of a HOTI driven by double band inversion at Γ, because the summed topology of the $k_y=0,\pi$ planes in the 3D BZ of $\mathcal{H}(\boldsymbol{k})$ is again nontrivial (and, equivalently, because the bulk is a symmetry-indicated HOTI [33, 100]), then we observe a nontrivial flux response. This further underlines the fact that, in contrast to the dislocation response, the π-flux response of 2D and

3D insulators only depends on the strong topological index (SN 2 A 3 and 2 B 2), and in particular does not depend on where bands are inverted. The bulk parity eigenvalues and the flux spectrum are respectively shown in SFig. 20 ***b***, ***e***. The dislocation response of an $\mathcal{I}$- and $\mathcal{T}$-symmetric HOTI with double band inversion at Z was previously discussed in detail in SN 4 B 2 b – further details of our numerical implementation are provided in that section.

c. $\xi = 0$: Weak $\mathcal{T}$-symmetric FTI stack The bulk parity eigenvalues and the flux spectrum are respectively shown in SFig. 20 ***c***, ***d***. Because the bulk carries trivial strong TI and HOTI indices, then the π-flux response is trivial. The dislocation response of a weak stack of $\mathcal{I}$- and $\mathcal{T}$-symmetric 2D FTIs was previously discussed in detail in SN 4 B 2 c – further details of our numerical implementation are provided in that section.

6. FIRST-PRINCIPLES CALCULATION DETAILS

A. 2D PbTe Monolayers

In this section, we will detail our first-principles and tight-binding calculations confirming a nontrivial dislocation response in 2D lead telluride (PbTe) monolayers. PbTe monolayers (SFig. 21 ***a***) have theoretically been predicted to be 2D TCIs [128–130] with mirror Chern number [15] $C_{M_z} = 2$. Previous works [128–130] have specifically determined that the nontrivial crystalline topology of PbTe monolayers is driven by band inversion at two $\boldsymbol{k}$ points along the 2D BZ boundary (SFig. 21 ***b***), suggesting the possibility of a nontrivial point dislocation response (see SN 3 A). In this section, we will use first-principles calculations to reproduce the electronic structure and topology of PbTe monolayers, which we will then use to determine the electronic response to point dislocations.

We begin by calculating the electronic structure of a PbTe monolayer. To obtain the crystal structure of a single, pristine monolayer, we start with a 3D crystal of rock-salt-structure PbTe [SG 225 $Fm\bar{3}m1'$, Inorganic Crystal Structure Database (ICSD) [280] No. 194220, further details available at https://topologicalquantumchemistry.com/#/detail/194220 [131, 132, 152, 238, 239]], increase the lattice spacing in the z (c-axis) direction to isolate a single plane of Pb and Te atoms (SFig. 21 ***a***), and then restrict the system symmetry to layer group (LG) [25, 70, 223, 227, 228, 237, 262] $p4/mmm1'$. Notably, 3D PbTe crystals, depending on the choice of simulation parameters and initial structure (indexed by ICSD number), have been shown to be "rotation-anomaly" TCIs with helical hinge states [26, 31, 32, 131–133]. We next perform fully relativistic density functional theory (DFT) calculations of the electronic structure using the Vienna Ab initio Simulation Package (VASP) [281, 282] employing the projector-augmented wave (PAW) method [283, 284] and the Perdew, Burke, and Ernzerhof generalized-gradient approximation (GGA-PBE) [285] for the exchange-correlation functional. In our first-principles calculations, we have used the

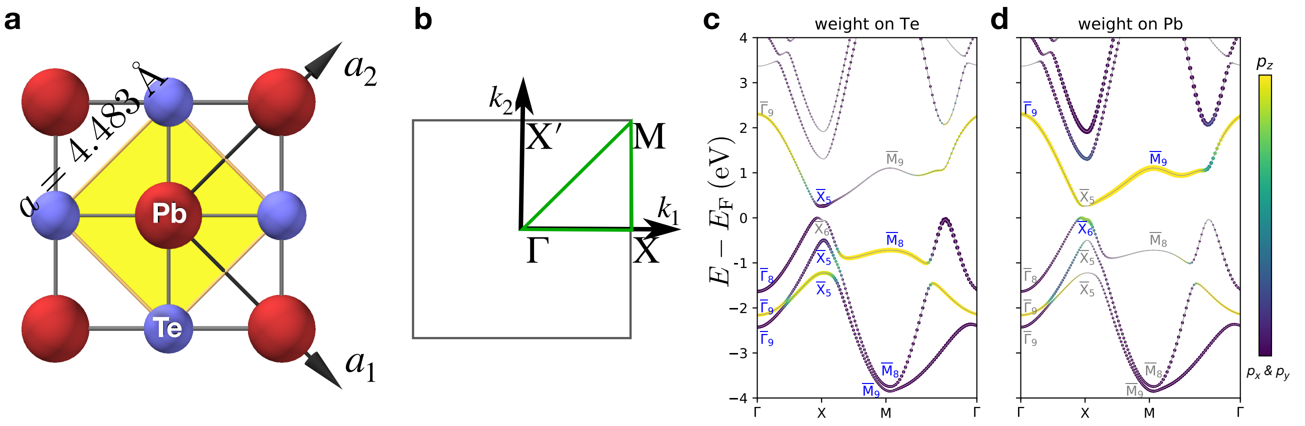

Supplementary Figure 21. **Bulk electronic structure of monolayer PbTe.** ***a*** The crystal structure of monolayer PbTe in layer group (LG) $p4/mmm1'$ [25, 70, 223, 227, 228, 237, 262]. The primitive (unit) cell is highlighted in yellow. ***b*** The BZ of LG $p4/mmm1'$. ***c***, ***d*** The band structure of monolayer PbTe calculated from first principles; bands at the TRIM points are labeled with the irreducible small corepresentations of $p4/mmm1'$ using the convention established in the REPRESENTATIONS DSG tool on the BCS [152, 243] for SG 123 $P4/mmm1'$, the index-2 space supergroup of LG $p4/mmm1'$ generated by adding lattice translations in the z direction. Bands at each k point are doubly degenerate due to the presence of $\mathcal{I} \times \mathcal{T}$ symmetry [227]. The relative projected spectral weight of the Te (Pb) p_z and $p_{x,y}$ orbitals nearest the Fermi energy is respectively indicated with yellow and blue, as shown in the color bar to the right of panel ***d***. The band structures and spectral weights in ***c*** and ***d*** indicate that PbTe monolayers differ from an unobstructed atomic limit by a band inversion between Bloch states with oppositely-signed Kramers pairs of parity eigenvalues [SEq. (177)] at the X [$\boldsymbol{k}_X = \boldsymbol{b}_1/2$] and X' [$\boldsymbol{k}_{X'} = C_{4z}\boldsymbol{k}_X$ mod $\boldsymbol{b}_1$ mod $\boldsymbol{b}_2 = \boldsymbol{b}_2/2$] points.

primitive unit cell shown in SFig. 21 ***a***, which contains one Pb atom at $(x, y) = (0, 0)$ and one Te atom at $(1/2, 0)$. The lattice vectors of the primitive cell (see SFig. 21 ***a***) are given by:

$$\boldsymbol{a}_1 = (1/2, -1/2),\ \boldsymbol{a}_2 = (1/2, 1/2), \tag{172}$$

and the reciprocal lattice vectors are given by:

$$\boldsymbol{b}_1 = 2\pi(1, -1),\ \boldsymbol{b}_2 = 2\pi(1, 1). \tag{173}$$

Lastly, we have allowed the in-plane lattice spacing $a_1 = a_2 = a$ to relax from its experimental value to an equilibrium length of $a = 4.483$ Å.

In SFig. 21 ***c, d***, we plot the electronic structure of a PbTe monolayer. The bands at each k point are doubly degenerate due to the presence of spinful $\mathcal{I} \times \mathcal{T}$ symmetry [227]. We find that the 12 bands closest to the Fermi energy (E_F) are induced from the $5p$ orbitals of Te and the $6p$ orbitals of Pb. To determine the location in momentum space and associated irreducible small corepresentations (coreps) of the bulk band inversion, we construct maximally-localized, symmetric Wannier functions (MLWFs) [256, 286] using Wannier90 [287, 288]. Specifically, to capture the six valence bands and two conduction bands closest to E_F, we construct MLWFs using Kramers pairs of $5p_{x,y,z}$ orbitals from Te and Kramers pairs of $6p_z$ orbitals from Pb. In SFig. 21 ***c*** (***d***) we show the electronic structure of a PbTe monolayer with the projected weight of the states at each k point onto the twelve total $5p$ Te and $6p$ Pb orbitals. We observe that the bands closest to E_F are well captured by the eight orbitals (Kramers pairs of $5p_{x,y,z}$ Te orbitals and $6p_z$ Pb orbitals) used to construct the MLWFs, and that the bulk differs from an obstructed atomic limit by band inversion at the X point between states from the $6p_z$ orbitals of Pb and the $5p_{x,y}$ orbitals of Te.

To determine the topology of the PbTe monolayer, we use the IrRep program [289] to deduce the small coreps of the six highest valence and the two lowest conduction bands (*i.e.* the bands previously selected to construct MLWFs), which are shown in SFig. 21 ***c, d*** and labeled employing the convention of the REPRESENTATIONS DSG tool on the BCS [152, 243] for the $k_z = 0$ plane of SG 123 $P4/mmm1'$, the index-2 supergroup of LG $p4/mmm1'$ generated by adding lattice translations in the z direction. Layer group $p4/mmm1'$, because of the presence of bulk C_{4z} and M_z symmetries, supports a symmetry-indicated mirror Chern number [267]:

$$\begin{aligned} C_{M_z} \bmod 4 =& \frac{3}{2}n\left(\bar{\Gamma}_6\right) - \frac{3}{2}n\left(\bar{\Gamma}_8\right) - \frac{1}{2}n\left(\bar{\Gamma}_7\right) + \frac{1}{2}n\left(\bar{\Gamma}_9\right) \\ &+ \frac{3}{2}n\left(\bar{M}_6\right) - \frac{3}{2}n\left(\bar{M}_8\right) - \frac{1}{2}n\left(\bar{M}_7\right) + \frac{1}{2}n\left(\bar{M}_9\right) \\ &+ n\left(\bar{X}_5\right) - n\left(\bar{X}_6\right) \bmod 4. \end{aligned} \tag{174}$$

The six highest valence bands in energy in SFig. 21 ***c, d*** are separated from the other valence bands by a large energy gap, and exhibit the small corep multiplicities:

$$\begin{aligned} & n\left(\bar{\Gamma}_6\right) = n\left(\bar{\Gamma}_7\right) = 0,\ n\left(\bar{\Gamma}_8\right) = 1,\ n\left(\bar{\Gamma}_9\right) = 2, \\ & n\left(\bar{M}_6\right) = n\left(\bar{M}_7\right) = 0,\ n\left(\bar{M}_8\right) = 2,\ n\left(\bar{M}_9\right) = 1, \\ & n\left(\bar{X}_5\right) = 2,\ n\left(\bar{X}_6\right) = 1. \end{aligned} \tag{175}$$

Substituting SEq. (175) into SEq. (174), we determine that PbTe monolayers exhibit:

$$C_{M_z} \bmod 4 = 2, \tag{176}$$

in agreement with the results of SRefs. 128–130.

Next, to determine the dislocation response of PbTe monolayers, we calculate the weak (partial) SSH invariant vector $\boldsymbol{M}_\nu^{\text{SSH}}$, which is defined in the text surrounding SEq. (106). $\boldsymbol{M}_\nu^{\text{SSH}}$ can be obtained by counting the number of parity-eigenvalue-exchanging band inversions by which a set of bands differs from an unobstructed (trivial) atomic limit with a trivial dislocation response. As shown in SFig. 21 ***c, d***, PbTe monolayers differ from an unobstructed atomic limit through band inversion at the X point [$\boldsymbol{k}_X = \boldsymbol{b}_1/2 = (\pi, -\pi)$] between bands labeled by the small coreps $\bar{X}_{5,6}$ of the little group at X. The small coreps $\bar{X}_{5,6}$ correspond to doubly-degenerate pairs of states with the same parity ($\mathcal{I}$) eigenvalues within each pair, such that:

$$\chi_{\bar{X}_5}(\mathcal{I}) = 2,\ \chi_{\bar{X}_6}(\mathcal{I}) = -2, \tag{177}$$

where $\chi_\rho(h)$ is the character of the unitary symmetry h in the corep ρ, and is equal to the sum of the eigenvalues of h in ρ. Because the X and symmetry-equivalent X' [$\boldsymbol{k}_{X'} = C_{4z}\boldsymbol{k}_X \bmod \boldsymbol{b}_1 \bmod \boldsymbol{b}_2 = \boldsymbol{b}_2/2 = (\pi, \pi)$] points lie along

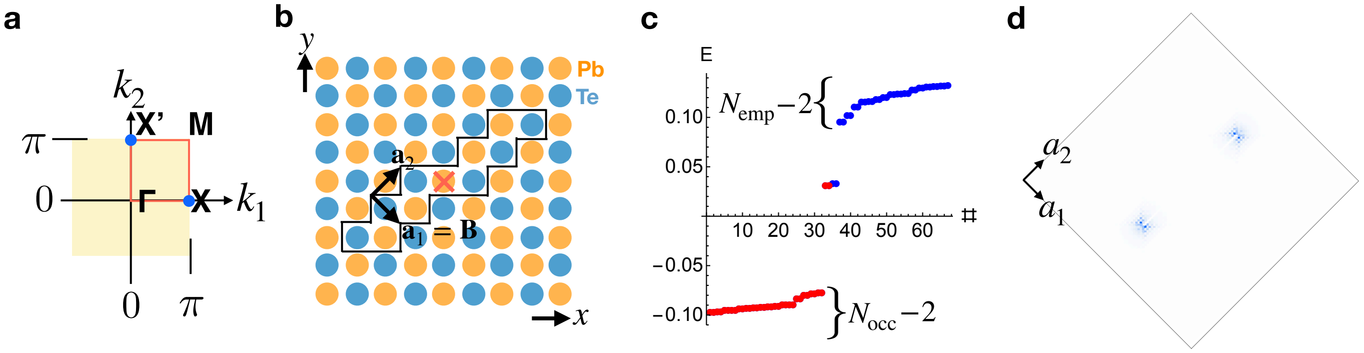

Supplementary Figure 22. **Dislocation response of a PbTe monolayer.** ***a*** Brillouin zone (BZ) of a pristine monolayer of PbTe, which respects layer group (LG) $p4/mmm1'$ [25, 70, 223, 227, 228, 237, 262]. Focusing on the six highest valence bands and the two lowest conduction bands in SFig. 21 ***c*, *d***, the highest (lowest) doubly degenerate pair of valence (conduction) bands is inverted at the X and symmetry-equivalent X' points (blue dots in ***a***). The band inversion exchanges Kramers pairs of states with opposite parity eigenvalues, driving the monolayer to exhibit nontrivial weak (partial) SSH indices $\boldsymbol{M}_{\nu}^{\rm SSH} = (\boldsymbol{b}_1 + \boldsymbol{b}_2)/2$ [see SN 3 A and the text surrounding SEq. (178)]. ***b*** Schematic of the real-space implementation of an $\mathcal{I}$-related pair of $\boldsymbol{B} = \boldsymbol{a}_1$ point dislocations in a tight-binding model of a PbTe monolayer obtained from first-principles calculations, where the $\mathcal{I}$ center is marked with a red $\times$ symbol. The black line in ***b*** encloses atoms with a fixed average value of the a_1 coordinate that were removed to create the pair of dislocations. ***c*** Energy spectrum of a tight-binding model of PbTe with the $\mathcal{I}$-related pair of $\boldsymbol{B} = \boldsymbol{a}_1$ point dislocations shown in ***b*** and periodic boundary conditions. The filling $N_{\rm occ}$ is set by assigning six occupied electrons to each Te atom in the system, corresponding to the six occupied Te p orbitals used to construct our Wannier-based tight-binding model [see the text following SEq. (173)]. ***d*** The real-space localization of the four midgap states. One Kramers pair of states is localized on each dislocation core, and, when half-filled, corresponds to a chargeless, spin-1/2 quasiparticle (*i.e.* a spinon) that is equivalent to the end state of a spinful SSH chain (SN 2 A). The white dislocation line in ***d*** does not carry any spectral weight because it represents a line of removed sites (*i.e.*, the atoms surrounded by black lines in panel ***b***). Because the dislocation geometry in ***b*** preserves $\mathcal{I}$ and spinful $\mathcal{T}$ symmetries, then the spectrum in ***c*** cannot be deformed into that of a trivial insulator with dislocations, and is thus *filling-anomalous* [33, 70, 100, 176]. The presence of anomalous chargeless spin bound to dislocations is consistent with SEq. (83) and with the discussion in SN 3 A, in which it was determined that 2D insulators with nontrivial weak (partial) SSH indices $\boldsymbol{M}_{\nu}^{\rm SSH}$ exhibit a filling-anomalous energy spectrum in the presence of point dislocations that satisfy $\boldsymbol{B}\cdot\boldsymbol{M}_{\nu}^{\rm SSH} \bmod 2\pi = \pi$.

the BZ-edge XM and $X'M$ lines, then, following the discussion in SN 3 A, we conclude that PbTe monolayers exhibit a nontrivial weak partial (time-reversal) SSH invariant vector:

$$\boldsymbol{M}_{\nu}^{\rm SSH} = \frac{1}{2}(\boldsymbol{b}_1 + \boldsymbol{b}_2) = (2\pi, 0). \tag{178}$$

We emphasize that, despite $\nu_x^{\rm SSH} \bmod 2\pi = \nu_y^{\rm SSH} \bmod 2\pi = 0$ in SEq. (178), $\boldsymbol{M}_{\nu}^{\rm SSH}$ is still nontrivial, because $(2\pi, 0)$ and $(0, 2\pi)$ are *not* reciprocal lattice vectors [SEq. (173)] in the rotated coordinates used in this section (which we have chosen because, as will be discussed in SN 6 B, the Bravais lattice of 3D PbTe is face-centered cubic, see SFig. 21 ***a*, *b***).

To confirm the nontrivial dislocation response of a PbTe monolayer, we will now insert a pair of 0D dislocations into the previous eight-band tight-binding model obtained from MLWFs through WANNIER90 [287, 288] [detailed in the text preceding SEq. (174)]. In practice, when mapping a DFT calculation to a tight-binding model, one must choose a cutoff distance for hopping interactions. Surprisingly, even though the band inversion in PbTe monolayers is relatively strong (the negative band gap at the X and X' points is roughly ~ 260 meV) [128–130], we find that the strong and weak partial-polarization topology of a PbTe monolayer is only reproduced in a tight-binding model that is truncated to a minimum range of *sixth-nearest-neighbor* hopping.

We next place the tight-binding model on periodic boundary conditions (PBC). Because the tight-binding model has six valence states and two conduction states per unit cell, then the PBC energy spectrum exhibits a valence (conduction) manifold with $6N/8$ ($2N/8$) states (*i.e.*, the spectrum is gapped at an electronic filling fraction $\nu/N = 3/4$). To probe the dislocation response, we insert a pair of 0D dislocations with Burgers vector $\boldsymbol{B} = \boldsymbol{a}_1$, which satisfies $\boldsymbol{B}\cdot\boldsymbol{M}_{\nu}^{\rm SSH} \bmod 2\pi = \pi$ [SEq. (178)]. We take the filling fraction $\nu/N = 3/4$ to be derived from three occupied Kramers pairs of electrons per Te atom, corresponding to the six occupied Te p orbitals used to construct our Wannier-based tight-binding model [see the text following SEq. (173)]. We then denote the corresponding number of filled states in the defect geometry as $N_{\rm occ}$. In terms of the tight-binding model used to demonstrate the dislocation response, prior to inserting the dislocations, we have employed a PBC geometry with 71×71 unit cells. We next implement the dislocations by removing the atoms surrounding a line M (defined in SN 4 A 1) of a linear extent of 40 cells and

reconnecting the sites across M (schematically shown in SFig. 22 ***b*** for a smaller system size). Hence, in our numerical implementation, $N_{\text{occ}} = 29994$.

In SFig. 22 ***c***, we plot the PBC spectrum of the $\mathcal{I}$- and spinful $\mathcal{T}$-symmetric Hamiltonian $\tilde{H}$ with two $\boldsymbol{B} = \boldsymbol{a}_1$ dislocations. The PBC spectrum in SFig. 22 ***c*** exhibits a filling anomaly [33, 70, 100, 176] at N_{occ} filled states, with the valence and conduction manifolds each specifically missing one Kramers pair of states. The four anomalous states appear as midgap Kramers pairs in the PBC defect spectrum at filling $\nu = N_{\text{occ}}$ in SFig. 22 ***c***, and are localized on the dislocation cores (SFig. 22 ***d***). When the system is three-quarters-filled, we expect the dislocation bound states to each be half filled in the limit that the dislocation separation is thermodynamically large. Analogous to the domain-wall (end) states of a spinful SSH chain [215–218, 258], each Kramers pair of dislocation bound states in SFig. 22 ***c, d*** corresponds to a chargeless, spin-1/2 quasiparticle (*i.e.* a spinon) when half filled (see SN 2 A).

To conclude, in this section, we have demonstrated that PbTe monolayers exhibit an anomalous dislocation response that is consistent with SEq. (83) and with the discussion in SN 3 A, in which it was determined that 2D insulators with nontrivial weak (partial) SSH indices $\boldsymbol{M}_{\nu}^{\text{SSH}}$ [SEq. (178)] exhibit an anomalous energy spectrum in the presence of point dislocations that satisfy $\boldsymbol{B} \cdot \boldsymbol{M}_{\nu}^{\text{SSH}} \bmod 2\pi = \pi$.

B. 3D SnTe

Three-dimensional tin telluride (SnTe) has been predicted to be a rotation-anomaly TCI with helical hinge states [26, 31, 32]. In this section, we will demonstrate that 3D SnTe crystals exhibit a nontrivial HEND-state dislocation response. First, in SN 6 B 1, we will use first-principles calculations to reproduce the electronic structure and diagnose the topology of 3D SnTe – as well as its topologically trivial counterpart 3D PbTe. We will then specifically demonstrate in SN 6 B 2 that 3D SnTe (PbTe) hosts a nontrivial (trivial) $\mathcal{I}$-symmetry-indicated response to 1D dislocations.

1. *First-Principles Calculation of the Nontrivial Dislocation Response Vector in SnTe*

In this section, we will use first principles to calculate the weak fragile index $\boldsymbol{M}_{\nu}^{\text{F}}$ [defined in SEqs. (111), (120), and in the surrounding text in SN 3 B 2] for 3D PbTe and SnTe. We begin by performing fully-relativistic DFT calculations of the electronic structure of 3D SnTe and PbTe using VASP [281, 282] employing the PAW method [283, 284] and GGA-PBE [285] for the exchange-correlation functional [131, 290]. The lattice parameters of the rock-salt structure [SG 225 $Fm\bar{3}m1'$] were fixed to their experimental values [291] $a = 6.32$ Å for SnTe and $a = 6.46$ Å for PbTe.

Below, we will specifically compute the dislocation response for the shortest possible dislocation Burgers vectors – *i.e.* dislocations for which the Burgers vector $\boldsymbol{B}$ is equal to one of the primitive, face-centered-cubic lattice vectors of SnTe or PbTe. We are particularly interested in the case in which $\boldsymbol{B}$ is equal to a primitive lattice vector, as the Frank energy criterion [292] implies that dislocations become increasingly energetically unfavorable with increasing $|\boldsymbol{B}|$. Hence, for geometric simplicity and because 3D SnTe and PbTe are cubic, we will, without loss of generality, form a tetragonal supercell in which the $\boldsymbol{a}_1$ and $\boldsymbol{a}_2$ primitive lattice vectors are also lattice vectors in the face-centered cubic cell, but in which $\boldsymbol{a}_3$ is $\sqrt{2}$ times the length of a face-centered-cubic primitive lattice vector [see SFig. 23 ***a***]. The tetragonal cell specifically contains two Sn/Pb atoms at $(x, y, z) = (0, 0, 0)$ and $(1/2, 1/2, 1/2)$ and two Te atoms at $(0, 0, 1/2)$ and $(1/2, 1/2, 0)$, and respects the symmetries of SG 123 $P4/mmm1'$. As shown in SFig. 23 ***a***, the lattice vectors of the tetragonal cell are given by:

$$\boldsymbol{a}_1 = (1/2, -1/2, 0),\ \boldsymbol{a}_2 = (1/2, 1/2, 0),\ \boldsymbol{a}_3 = (0, 0, 1), \tag{179}$$

in units in which the lattice spacing $a = 1$, and the reciprocal lattice vectors are given by:

$$\boldsymbol{b}_1 = 2\pi(1, -1, 0),\ \boldsymbol{b}_2 = 2\pi(1, 1, 0),\ \boldsymbol{b}_3 = 2\pi(0, 0, 1). \tag{180}$$

In our first-principles calculations, we only incorporate-valence shell states – hence, our calculations only include the $5p$ orbitals of Te and $5p$ ($6p$) orbitals of Sn (Pb), as well as twelve total empty conduction bands from higher-shell (empty) valence orbitals. Therefore, at each TRIM point in SFig. 23 ***c, d***, the lower twelve (upper twelve) bands are occupied (unoccupied) [the bands in SFig. 23 ***c, d*** are fourfold degenerate due to the combined effects of spinful $\mathcal{I} \times \mathcal{T}$ symmetry and supercell BZ folding].

As shown in SN 2 and 3, $\boldsymbol{M}_{\nu}^{\text{F}}$ can be obtained by counting the number of parity-eigenvalue-exchanging band inversions by which a set of bands differs from an unobstructed atomic limit with a trivial dislocation response. We first establish that 3D PbTe is an unobstructed atomic limit [18] in which three Kramers pairs of states are located on each of the four Te atoms in the tetragonal supercell – this can be seen from the absence of band inversions in

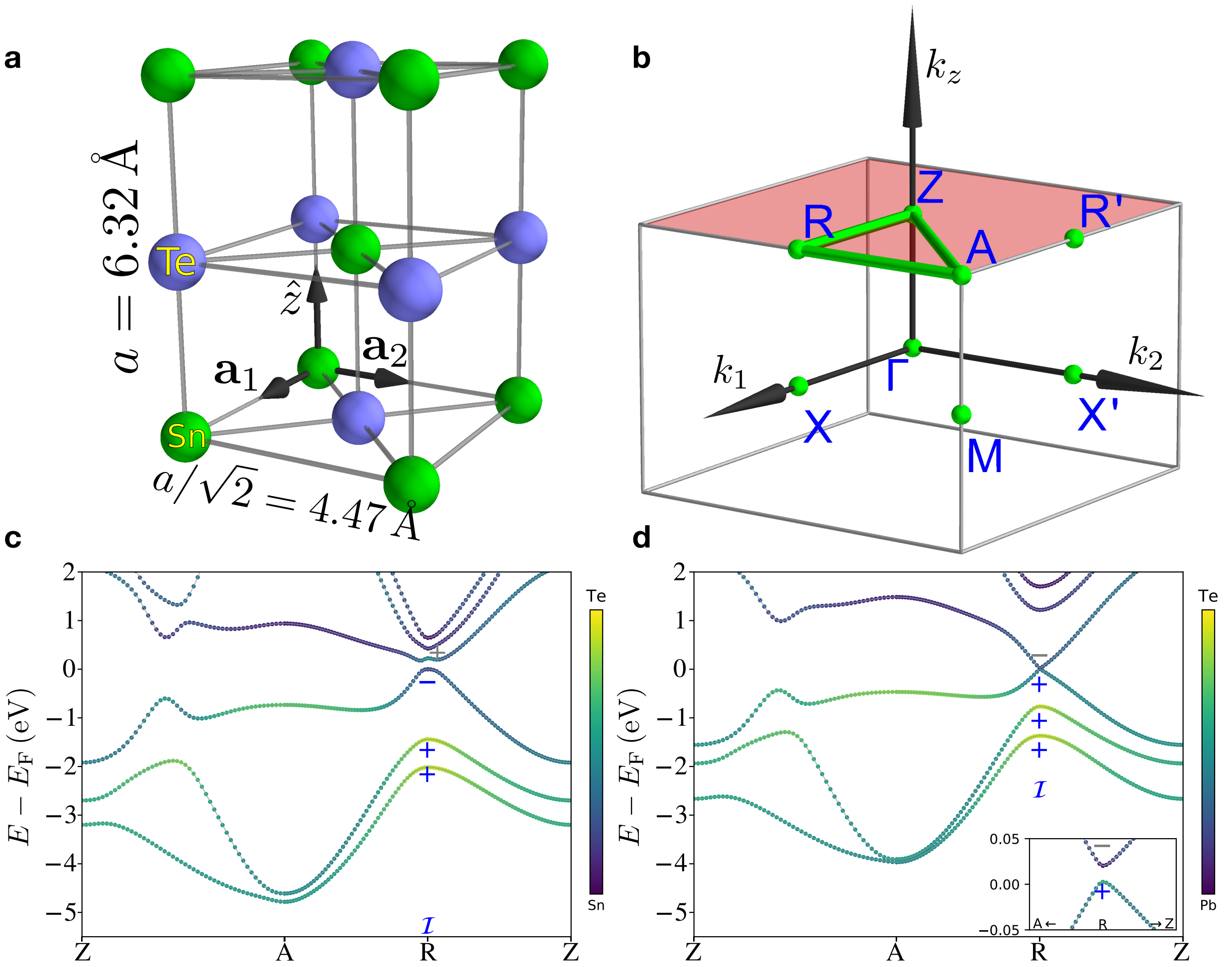

Supplementary Figure 23. **Bulk electronic structures of 3D SnTe and PbTe.** ***a*** Crystal structure of 3D SnTe in a tetragonal supercell. The supercell has twice as many atoms (four in total) as the primitive (unit) cell, and respects the symmetries of SG 123 $P4/mmm1'$. In 3D PbTe, the crystal structure is the same as in ***a***, with the Sn atoms replacing the Pb atoms. ***b*** The BZ of SnTe and PbTe in the tetragonal supercell in ***a***. ***c*, *d*** The electronic structures of ***c*** SnTe and ***d*** PbTe calculated from first-principles and plotted along the path indicated in ***b*** with a green line. The bands in ***c*** and ***d*** exhibit an artificial fourfold degeneracy at all $\boldsymbol{k}$ points, due to the combined effects of spinful $\mathcal{I} \times \mathcal{T}$ symmetry and supercell BZ folding. The $+$ and $-$ signs in ***c*, *d*** denote the parity eigenvalues per Kramers pair of the Bloch states at the TRIM point R $[\boldsymbol{k}_R = \boldsymbol{b}_1/2]$. The band structures in ***c*, *d*** indicate that 3D SnTe differs from 3D PbTe by a double band inversion at the R point and at the symmetry-related point R' $[\boldsymbol{k}_{R'} = C_{4z}\boldsymbol{k}_R \bmod \boldsymbol{b}_1 \bmod \boldsymbol{b}_2 = \boldsymbol{b}_2/2]$ between two pairs of Kramers pairs of states with opposite parity eigenvalues [four valence bands and four conduction bands become inverted at R and at R']. Because PbTe in ***d*** is an unobstructed atomic limit, then the double band inversion in SnTe leads to a nontrivial weak (partial) fragile index vector $\boldsymbol{M}^{\mathrm{F}}_{\nu} = (\boldsymbol{b}_1 + \boldsymbol{b}_2)/2$ [see SN 3 B and the text surrounding SEq. (186)].

SFig. 23 ***c***, and has been shown in previous works [18]. The band structures in SFig. 23 ***c*, *d*** indicate that 3D SnTe differs from 3D PbTe by double band inversions at the R point $[\boldsymbol{k}_R = \boldsymbol{b}_1]$ and at the symmetry-related point R' $[\boldsymbol{k}_{R'} = C_{4z}\boldsymbol{k}_R \bmod \boldsymbol{b}_1 \bmod \boldsymbol{b}_2 = \boldsymbol{b}_2/2]$ between two pairs of Kramers pairs of states with opposite parity eigenvalues [four valence states become inverted with four conduction states at R and at R'].

Before calculating the dislocation response index vector $\boldsymbol{M}^{\mathrm{F}}_{\nu}$, we will next calculate the stable topological indices of 3D SnTe (as we have established that 3D PbTe is topologically trivial when its electronic structure is calculated as detailed above). First, SRefs. 32, 103, and 105 previously established the existence of a strong $\mathbb{Z}_8$-valued topological index z_8 in SG 123 $P4/mmm1'$. Below, we reproduce the specific formula for z_8 provided in Supplementary Table 2 in SRef. 103:

$$z_8 = \frac{1}{2}\left[3n^+_{\frac{3}{2}} - 3n^-_{\frac{3}{2}} - n^+_{\frac{1}{2}} + n^-_{\frac{1}{2}}\right] \bmod 8. \tag{181}$$

Applying the IrRep program [289] to the output of our first-principles calculations to obtain the small coreps that correspond to the occupied Bloch states of 3D SnTe, we find that, using the definitions for the small corep multiplicities $n^{\pm}_{1/2,3/2}$ established in SRef. 103 [*c.f.* Supplementary Table 1 in SRef. 103]:

$$n^{+}_{\frac{1}{2}} = 11, \quad n^{-}_{\frac{1}{2}} = 15, \quad n^{+}_{\frac{3}{2}} = 9, \quad n^{-}_{\frac{3}{2}} = 13. \tag{182}$$

SEqs. (181) and (182) together indicate that:

$$z_8 = 4, \tag{183}$$

confirming that SnTe realizes an $\mathcal{I}$- and C_{4z}-indicated fourfold-rotation-anomaly TCI.

In SG 123 $P4/mmm1'$ there are also weak mirror Chern numbers and $\mathbb{Z}_2$ 2D TI indices [32, 103, 105]. First, through the Fu-Kane weak-index parity indices [8], the small corep multiplicities in SEq. (182) imply that the weak $\mathbb{Z}_2$ indices in the $\boldsymbol{b}_{1,2}/2$ planes are trivial. Next, we calculate the $\mathbb{Z}_4$-valued index $z_{4m,\pi}$ established in SRefs. 32, 103, 105, and 267:

$$\begin{aligned} z_{4m,\pi} =& \frac{3}{2}n\left(E^{Z}_{\frac{3}{2}g}\right) - \frac{3}{2}n\left(E^{Z}_{\frac{3}{2}u}\right) - \frac{1}{2}n\left(E^{Z}_{\frac{1}{2}g}\right) + \frac{1}{2}n\left(E^{Z}_{\frac{1}{2}u}\right) \\ &+ \frac{3}{2}n\left(E^{A}_{\frac{3}{2}g}\right) - \frac{3}{2}n\left(E^{A}_{\frac{3}{2}u}\right) - \frac{1}{2}n\left(E^{A}_{\frac{1}{2}g}\right) + \frac{1}{2}n\left(E^{A}_{\frac{1}{2}u}\right) \\ &+ n\left(E^{R}_{\frac{1}{2}g}\right) - n\left(E^{R}_{\frac{1}{2}u}\right) \bmod 4, \end{aligned} \tag{184}$$

where $z_{4m,\pi}$ indicates the mirror Chern number in the $k_z = \pi$ plane C_{M_z} mod 4. Restricting the small corep multiplicities obtained in our first-principles calculations to the $k_z = \pi$ plane, we determine that

$$\begin{aligned} &n\left(E^{Z}_{\frac{3}{2}g}\right) = 1, \quad n\left(E^{Z}_{\frac{3}{2}u}\right) = 1, \quad n\left(E^{Z}_{\frac{1}{2}g}\right) = 2, \quad n\left(E^{Z}_{\frac{1}{2}u}\right) = 2, \\ &n\left(E^{A}_{\frac{3}{2}g}\right) = 1, \quad n\left(E^{A}_{\frac{3}{2}u}\right) = 2, \quad n\left(E^{A}_{\frac{1}{2}g}\right) = 2, \quad n\left(E^{A}_{\frac{1}{2}u}\right) = 1, \\ &n\left(E^{R}_{\frac{1}{2}g}\right) = 4, \quad n\left(E^{R}_{\frac{1}{2}u}\right) = 2, \end{aligned} \tag{185}$$

which implies that $z_{4m,\pi} = 0$, such that $C_{M_z}(k_z = \pi)$ mod $4 = 0$. In agreement with previous works [18, 31, 103], we have additionally numerically confirmed through Wilson-loop calculations that $C_{M_z}(k_z = \pi) = 0$ in the tetragonal supercell of SnTe.

In this work, we have focused on SnTe specifically because the R and R' points lie on the BZ boundary, such that the previously-established band inversions in SnTe [18] imply a nontrivial dislocation response (see SN 2). To determine the dislocation response of SnTe, we first establish that $\boldsymbol{M}^{\mathrm{F}}_{\nu} = \boldsymbol{0}$ in PbTe, because PbTe is an unobstructed atomic limit. Hence, because SnTe differs from PbTe by double band inversions at the R and R' points in the tetragonal cell in SFig. 23 **a** between two pairs of Kramers pairs of states with opposite parity eigenvalues [four valence states become inverted with four conduction states at R and at R'], then the HEND-state response of SnTe is *nontrivial*:

$$\boldsymbol{M}^{\mathrm{F}}_{\nu} = (\boldsymbol{b}_1 + \boldsymbol{b}_2)/2 = (2\pi, 0, 0). \tag{186}$$

We emphasize that, despite ν^{F}_x mod $2\pi = \nu^{\mathrm{F}}_y$ mod $2\pi = 0$ in SEq. (186), $\boldsymbol{M}^{\mathrm{F}}_{\nu}$ is still nontrivial, because $(2\pi, 0, 0)$ and $(0, 2\pi, 0)$ are *not* reciprocal lattice vectors in the tetragonal supercell of SnTe [SEq. (180)].

2. *Tight-Binding Calculation of Topological HEND States in 3D SnTe*

In this section, we will use tight-binding calculations to demonstrate a nontrivial $\mathcal{I}$-symmetry-indicated HEND-state response in 3D SnTe. Specifically, in the text surrounding SEq. (186), we previously demonstrated that the bulk of 3D SnTe carries a nontrivial HEND-state dislocation response vector $\boldsymbol{M}^{\mathrm{F}}_{\nu} = (\boldsymbol{b}_1 + \boldsymbol{b}_2)/2$, where $\boldsymbol{b}_{1,2}$ are defined in SEq. (180). Below, we will demonstrate that, as predicted by SEq. (40) in SN 2 A 1, an $\mathcal{I}$-symmetric configuration of edge dislocations in SnTe with Burgers vector $\boldsymbol{B}$ satisfying $\boldsymbol{B} \cdot \boldsymbol{M}^{\mathrm{F}}_{\nu}$ mod $2\pi = \pi$ will bind anomalous 0D states at $\mathcal{I}$-related locations along the set of dislocations. In fact, the shortest dislocations with integer Burgers vectors $\boldsymbol{B} = \boldsymbol{a}_{1,2}$ satisfy the requirement that $\boldsymbol{B} \cdot \boldsymbol{M}^{\mathrm{F}}_{\nu}$ mod $2\pi = \pi$. Furthermore, because the Frank energy criterion [292] indicates that dislocations with larger values of $|\boldsymbol{B}|$ are energetically unfavorable, then dislocations with the smallest possible integer Burgers vectors $\boldsymbol{B} = \boldsymbol{a}_{1,2}$ may be energetically favorable and present in SnTe samples. We note that

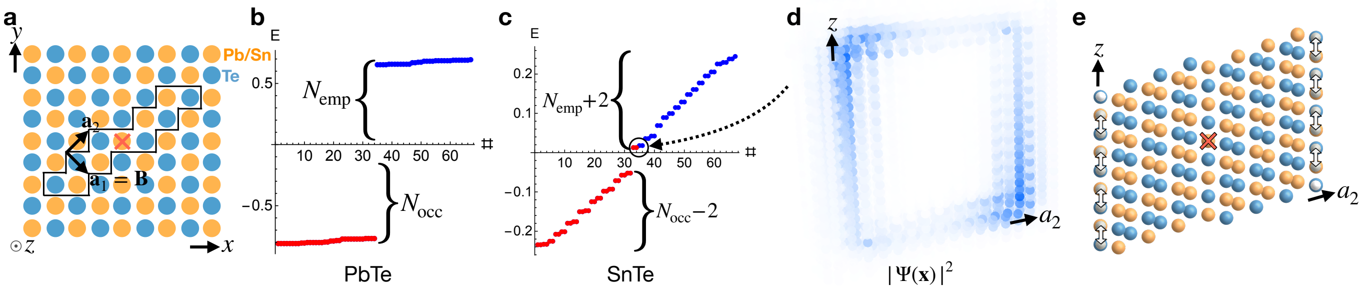

Supplementary Figure 24. **Defect spectra of 3D PbTe and SnTe for edge dislocations with Burgers vector $\boldsymbol{B} = \boldsymbol{a}_1$.** $\boldsymbol{a}$ Defect geometry for an $\mathcal{I}$-related pair of edge dislocations, where the $\mathcal{I}$ center is marked with a red × symbol. The black line in $\boldsymbol{a}$ encloses the cross section of the sites surrounding a plane with fixed $\boldsymbol{a}_1$ that were removed to create the internal dislocation pair [our numerical calculations were performed employing a PBC grid of size 11 (along $\boldsymbol{a}_1$) × 17 (along $\boldsymbol{a}_2$) × 17 (along $\boldsymbol{a}_3 = \hat{z}$) unit cells and a 23 (in the $\boldsymbol{a}_1, \boldsymbol{a}_2$ plane) × 25 (along $\hat{z}$) site defect plane (where a single unit cell is taken to contain four sites, see SFig. 23 $\boldsymbol{a}$)]. The calculations of the dislocation spectrum were performed using the tight-binding model for SnTe introduced in SRef. 18. $\boldsymbol{b}, \boldsymbol{c}$ The PBC dislocation spectrum of $\boldsymbol{b}$ 3D PbTe and $\boldsymbol{c}$ 3D SnTe using the edge dislocation geometry in $\boldsymbol{a}$. As previously for 2D monolayer PbTe (see SN 6 A), the electronic filling of $\nu = N_{\text{occ}}$ states is set by the unobstructed atomic limit of six electrons per Te atom, in addition to the tightly-bound core-shell electrons (which coincides with the bulk trivial topology of PbTe, see SN 6 A). The defect spectrum for PbTe in $\boldsymbol{b}$ exhibits a large, trivial gap. Conversely, the dislocation spectrum in $\boldsymbol{c}$ for SnTe exhibits midgap states. Specifically, because the dislocation geometry in $\boldsymbol{a}$ preserves $\mathcal{I}$ and spinful $\mathcal{T}$ symmetries, then the spectrum in $\boldsymbol{c}$ for SnTe cannot be deformed into that of a trivial insulator with dislocations, and is thus filling-anomalous [33, 70, 100, 176]. $\boldsymbol{d}$ The real-space profile of the four (circled) states in $\boldsymbol{c}$ that contribute to the filling anomaly of the SnTe spectrum. In $\boldsymbol{d}$, two total Kramers pairs of states are localized on $\mathcal{I}$-related dislocation corners (one Kramers pair of states is bound to every other corner, for a total of four, filling-anomalous HEND states). When the HEND states in $\boldsymbol{d}$ are half-filled, each Kramers pair corresponds to a chargeless, spin-1/2 quasiparticle (*i.e.* a spinon) that is equivalent to the corner state of an $\mathcal{I}$- and $\mathcal{T}$-symmetric 2D FTI (see SN 4 B 2 and SRefs. 33 and 100). $\boldsymbol{e}$ The SnTe defect plane (black cross-section) from $\boldsymbol{a}$ schematically depicted as a stack of PbTe monolayer defect lines (SFig. 22 $\boldsymbol{b}, \boldsymbol{d}$). In $\boldsymbol{e}$, each defect line has two 0D dislocations on its end, which each bind first-order 0D topological dislocation states. In an $\mathcal{I}$-symmetric stack of dislocation lines, the 0D dislocations evolve into 1D edge dislocations, and the 0D states pairwise annihilate, leaving two, filling-anomalous HEND states. We choose PbTe for the monolayers – rather than SnTe – because the interlayer coupling in realistic 3D PbTe drives additional band inversions, whereas a tetragonal supercell of 3D SnTe has the same x, y components of the $\boldsymbol{M}_\nu^{\text{F}}$ vector as a decoupled stack of PbTe monolayers (see SN 6 A). Hence, in the same sense that a helical (non-axionic) HOTI is equivalent to an $\mathcal{I}$-symmetric stack of 2D TIs (with an odd total number of layers) [31, 100, 102, 103, 155, 211], HEND dislocation states can be considered the result of stacking and symmetrically coupling (gray arrows in $\boldsymbol{e}$) an odd number of 2D monolayers that each contain first-order dislocation bound states.

screw dislocations with $\boldsymbol{B} = \boldsymbol{a}_{1,2}$, unlike edge dislocations, must reach a crystal surface of SnTe, and cannot form internal loops with a spatially constant Burgers vector (see SN 2 A 1 and 2 A 2). Additionally, because SnTe is a (rotation-anomaly) TCI with gapless surface states [18, 31, 103], then the HEND states bound to screw dislocations in SnTe may be obscured due to their coexistence with gapless surface states.

We will now explicitly confirm the nontrivial defect response of 3D SnTe predicted by our first-principles calculations in SN 6 B 1. To model an edge dislocation in SnTe, we use the tight-binding model from SRef. 18, with the parameters listed in SRef. 293. We first enlarge the model unit cell to obtain the tetragonal supercell shown in SFig. 23 $\boldsymbol{a}$. We then determine the locations of the $\mathcal{I}$ centers in the supercell from the mirror symmetry representations given in SRef. 293 – in real space, the Sn and Te atoms in the model in SRef. 18 occupy inversion centers that coincide with lines of C_{4z} symmetry in the tetragonal supercell (SFig. 23 $\boldsymbol{a}$).

We next implement an internal edge dislocation with $\boldsymbol{B} = \boldsymbol{a}_1$ as detailed in SN 4 A 1. First, because 3D PbTe is isostructural to 3D SnTe, then the tetragonal supercell in SFig. 23 $\boldsymbol{a}$ can be considered a bilayer of the 2D PbTe crystal structure in SFig. 21 $\boldsymbol{a}$. Hence, a finite 2D plane bounded on two sides by $\boldsymbol{B} = \boldsymbol{a}_1$, 1D dislocations in 3D SnTe can be constructed by stacking alternating bilayers of the 1D line bounded by two $\boldsymbol{B} = \boldsymbol{a}_1$, 0D dislocations in a monolayer of SnTe with the same crystal structure as PbTe in SFig. 22 $\boldsymbol{b}$. Importantly, in order to use filling anomalies to diagnose the nontrivial HEND-state dislocation response, we must implement the defect plane in an $\mathcal{I}$-symmetric manner, which we accomplish with the alternating pattern of site removal depicted in SFig. 24 $\boldsymbol{a}$.

To provide a reference for our numerical analysis of the defect response in 3D SnTe, we have also implemented a $\boldsymbol{B} = \boldsymbol{a}_1$ pair of edge dislocations in a tight-binding model of 3D PbTe. To construct the tight-binding model, we have increased the on-site energy difference between the two inequivalent atoms in the primitive unit cell [specifically,

in the notation of SRef. 293, we have changed the parameter m from 1.65 to 3 in Eq. (16) in SRef. 293]. Increasing the on-site energies reverses the pair of double band inversions at R and R' (see SN 6 B 1), and reproduces the first-principles-derived parity eigenvalues and electronic structure of PbTe (SFig. 23 ***b***). The on-site potential can also be understood as a chemical potential that localizes all of the electrons on the Te atoms of PbTe, in agreement with our diagnosis in SN 6 B 1 of 3D PbTe as an unobstructed (trivial) atomic limit. Because PbTe is isostructural to SnTe, then the real-space defect geometry for our tight-binding model of PbTe is identical to the defect geometry previously employed in SnTe (depicted in SFig. 24 ***a***).

In SFig. 24 ***b*** (SFig. 24 ***c***), we plot the PBC defect spectrum for PbTe (SnTe) using the tight-binding models and $\boldsymbol{B} = \boldsymbol{a}_1$ internal edge dislocations described earlier in this section. For both SnTe and PbTe, we consider the valence electrons to originate from the Te p orbitals, as described in SN 6 B 1 – hence, N_{occ} in the defect spectra in SFig. 24 ***b, c*** is equal to six times the number of Te atoms in the PBC defect geometry (*i.e.*, excluding the Te atoms removed to implement the dislocations). The dislocation spectrum of PbTe exhibits a large gap and is trivial, whereas the defect spectrum of SnTe is conversely filling-anomalous, specifically exhibiting four midgap states (two Kramers pairs corresponding to the circled states in SFig. 24 ***c***). As shown in SFig. 24 ***d***, the four filling-anomalous defect states in SnTe are localized on two $\mathcal{I}$-related dislocation corners, and are thus HEND states. When the system has an electronic filling of $\nu = N_{\text{occ}}$, the dislocation bound states will each be half filled in the limit that the dislocation separation is thermodynamically large. Analogous to the corner states of $\mathcal{I}$- and $\mathcal{T}$-symmetric 2D FTIs, each Kramers pair of dislocation bound states corresponds to a chargeless, spin-1/2 quasiparticle (*i.e.* a spinon) when half filled (see SN 2 A). To conclude, we have demonstrated a nontrivial HEND-state dislocation response in 3D SnTe that is consistent with SEq. (83) and with the discussion in SN 3 B.

---

[1] Hasan, M. Z. & Kane, C. L. Colloquium: Topological insulators. *Rev. Mod. Phys.* **82**, 3045–3067 (2010). URL https://link.aps.org/doi/10.1103/RevModPhys.82.3045.

[2] Wieder, B. J. *et al.* Topological materials discovery from crystal symmetry. *Nature Reviews Materials* (2021). URL https://doi.org/10.1038/s41578-021-00380-2.

[3] Bernevig, B. A., Hughes, T. L. & Zhang, S.-C. Quantum Spin Hall Effect and Topological Phase Transition in HgTe Quantum Wells. *Science* **314**, 1757–1761 (2006). URL http://science.sciencemag.org/content/314/5806/1757.

[4] Bernevig, B. A. & Zhang, S.-C. Quantum Spin Hall Effect. *Phys. Rev. Lett.* **96**, 106802 (2006). URL https://link.aps.org/doi/10.1103/PhysRevLett.96.106802.

[5] Kane, C. L. & Mele, E. J. Quantum Spin Hall Effect in Graphene. *Phys. Rev. Lett.* **95**, 226801 (2005). URL https://link.aps.org/doi/10.1103/PhysRevLett.95.226801.

[6] Kane, C. L. & Mele, E. J. Z_2 Topological Order and the Quantum Spin Hall Effect. *Phys. Rev. Lett.* **95**, 146802 (2005). URL https://link.aps.org/doi/10.1103/PhysRevLett.95.146802.

[7] Fu, L., Kane, C. L. & Mele, E. J. Topological insulators in three dimensions. *Phys. Rev. Lett.* **98**, 106803 (2007). URL https://link.aps.org/doi/10.1103/PhysRevLett.98.106803.

[8] Fu, L. & Kane, C. L. Topological insulators with inversion symmetry. *Phys. Rev. B* **76**, 045302 (2007). URL https://link.aps.org/doi/10.1103/PhysRevB.76.045302.

[9] Moore, J. E. & Balents, L. Topological invariants of time-reversal-invariant band structures. *Phys. Rev. B* **75**, 121306 (2007). URL https://link.aps.org/doi/10.1103/PhysRevB.75.121306.

[10] Hughes, T. L., Prodan, E. & Bernevig, B. A. Inversion-symmetric topological insulators. *Phys. Rev. B* **83**, 245132 (2011). URL https://link.aps.org/doi/10.1103/PhysRevB.83.245132.

[11] Hsieh, D. *et al.* A topological Dirac insulator in a quantum spin Hall phase. *Nature* **452**, 970–974 (2008). URL https://doi.org/10.1038/nature06843.

[12] König, M. *et al.* Quantum Spin Hall Insulator State in HgTe Quantum Wells. *Science* **318**, 766–770 (2007). URL http://science.sciencemag.org/content/318/5851/766.

[13] Qi, X.-L., Hughes, T. L. & Zhang, S.-C. Topological field theory of time-reversal invariant insulators. *Phys. Rev. B* **78**, 195424 (2008). URL https://link.aps.org/doi/10.1103/PhysRevB.78.195424.

[14] Fu, L. Topological crystalline insulators. *Phys. Rev. Lett.* **106**, 106802 (2011). URL https://link.aps.org/doi/10.1103/PhysRevLett.106.106802.

[15] Teo, J. C. Y., Fu, L. & Kane, C. L. Surface states and topological invariants in three-dimensional topological insulators: Application to $Bi_{1-x}Sb_x$. *Phys. Rev. B* **78**, 045426 (2008). URL https://link.aps.org/doi/10.1103/PhysRevB.78.045426.

[16] Huang, S.-J., Song, H., Huang, Y.-P. & Hermele, M. Building crystalline topological phases from lower-dimensional states. *Phys. Rev. B* **96**, 205106 (2017). URL https://link.aps.org/doi/10.1103/PhysRevB.96.205106.

[17] Song, Z., Huang, S.-J., Qi, Y., Fang, C. & Hermele, M. Topological states from topological crystals. *Science Advances* **5**, eaax2007 (2019). URL https://www.science.org/doi/abs/10.1126/sciadv.aax2007.

[18] Hsieh, T. H. *et al.* Topological crystalline insulators in the SnTe material class. *Nature Communications* **3**, 982 (2012). URL https://doi.org/10.1038/ncomms1969.

[19] Fang, C. & Fu, L. New classes of three-dimensional topological crystalline insulators: Nonsymmorphic and magnetic. *Phys. Rev. B* **91**, 161105 (2015). URL https://link.aps.org/doi/10.1103/PhysRevB.91.161105.

[20] Watanabe, H. & Fu, L. Topological crystalline magnets: Symmetry-protected topological phases of fermions. *Phys. Rev. B* **95**, 081107 (2017). URL https://link.aps.org/doi/10.1103/PhysRevB.95.081107.

[21] Slager, R.-J., Mesaros, A., Juričić, V. & Zaanen, J. The space group classification of topological band-insulators. *Nature Physics* **9**, 98–102 (2013). URL https://doi.org/10.1038/nphys2513.

[22] Kruthoff, J., de Boer, J., van Wezel, J., Kane, C. L. & Slager, R.-J. Topological classification of crystalline insulators through band structure combinatorics. *Phys. Rev. X* **7**, 041069 (2017). URL https://link.aps.org/doi/10.1103/PhysRevX.7.041069.

[23] Wang, Z., Alexandradinata, A., Cava, R. J. & Bernevig, B. A. Hourglass fermions. *Nature* **532**, 189–194 (2016). URL https://doi.org/10.1038/nature17410.

[24] Alexandradinata, A., Wang, Z. & Bernevig, B. A. Topological insulators from group cohomology. *Phys. Rev. X* **6**, 021008 (2016). URL https://link.aps.org/doi/10.1103/PhysRevX.6.021008.

[25] Wieder, B. J. *et al.* Wallpaper fermions and the nonsymmorphic Dirac insulator. *Science* **361**, 246–251 (2018). URL http://science.sciencemag.org/content/361/6399/246.

[26] Fang, C. & Fu, L. New classes of topological crystalline insulators having surface rotation anomaly. *Science Advances* **5**, eaat2374 (2019). URL https://www.science.org/doi/abs/10.1126/sciadv.aat2374.

[27] Hsu, C.-H. *et al.* Purely rotational symmetry-protected topological crystalline insulator α-Bi_4Br_4. *2D Materials* (2019). URL http://iopscience.iop.org/10.1088/2053-1583/ab1607.

[28] Yoon, C., Liu, C.-C., Min, H. & Zhang, F. Quasi-One-Dimensional Higher-Order Topological Insulators. *arXiv e-prints* arXiv:2005.14710 (2020). 2005.14710.

[29] Zhang, T. *et al.* Topological crystalline insulators with C_2 rotation anomaly. *Phys. Rev. Research* **1**, 012001 (2019). URL https://link.aps.org/doi/10.1103/PhysRevResearch.1.012001.

[30] Benalcazar, W. A., Bernevig, B. A. & Hughes, T. L. Electric multipole moments, topological multipole moment pumping, and chiral hinge states in crystalline insulators. *Phys. Rev. B* **96**, 245115 (2017). URL https://link.aps.org/doi/10.1103/PhysRevB.96.245115.

[31] Schindler, F. *et al.* Higher-order topological insulators. *Science Advances* **4**, eaat0346 (2018). URL https://www.science.org/doi/abs/10.1126/sciadv.aat0346.

[32] Song, Z., Fang, Z. & Fang, C. $(d-2)$-Dimensional Edge States of Rotation Symmetry Protected Topological States. *Phys. Rev. Lett.* **119**, 246402 (2017). URL https://link.aps.org/doi/10.1103/PhysRevLett.119.246402.

[33] Wieder, B. J. & Bernevig, B. A. The Axion Insulator as a Pump of Fragile Topology. *ArXiv e-prints* (2018). 1810.02373.

[34] van Miert, G. & Ortix, C. Higher-order topological insulators protected by inversion and rotoinversion symmetries. *Phys. Rev. B* **98**, 081110 (2018). URL https://link.aps.org/doi/10.1103/PhysRevB.98.081110.

[35] Langbehn, J., Peng, Y., Trifunovic, L., von Oppen, F. & Brouwer, P. W. Reflection-symmetric second-order topological insulators and superconductors. *Phys. Rev. Lett.* **119**, 246401 (2017). URL https://link.aps.org/doi/10.1103/PhysRevLett.119.246401.

[36] Trifunovic, L. & Brouwer, P. W. Higher-order bulk-boundary correspondence for topological crystalline phases. *Phys. Rev. X* **9**, 011012 (2019). URL https://link.aps.org/doi/10.1103/PhysRevX.9.011012.

[37] Tyner, A. C. & Goswami, P. Symmetry indicators vs. bulk winding numbers of topologically non-trivial bands. *arXiv e-prints* arXiv:2109.06871 (2021). 2109.06871.

[38] Velury, S., Bradlyn, B. & Hughes, T. L. Topological crystalline phases in a disordered inversion-symmetric chain. *Phys. Rev. B* **103**, 024205 (2021). URL https://link.aps.org/doi/10.1103/PhysRevB.103.024205.

[39] Komori, S. & Kondo, K. A proposal of strong and weak phases in second-order topological insulators. *Journal of Physics Communications* **4**, 125005 (2020). URL https://doi.org/10.1088/2399-6528/abd0d4.

[40] Ghorashi, S. A. A., Hu, X., Hughes, T. L. & Rossi, E. Second-order Dirac superconductors and magnetic field induced Majorana hinge modes. *Phys. Rev. B* **100**, 020509 (2019). URL https://link.aps.org/doi/10.1103/PhysRevB.100.020509.

[41] You, Y., Devakul, T., Burnell, F. J. & Neupert, T. Higher-order symmetry-protected topological states for interacting bosons and fermions. *Phys. Rev. B* **98**, 235102 (2018). URL https://link.aps.org/doi/10.1103/PhysRevB.98.235102.

[42] Wieder, B. J., Lin, K.-S. & Bradlyn, B. Axionic band topology in inversion-symmetric Weyl-charge-density waves. *Phys. Rev. Research* **2**, 042010 (2020). URL https://link.aps.org/doi/10.1103/PhysRevResearch.2.042010.

[43] Yu, J., Wieder, B. J. & Liu, C.-X. Dynamical piezomagnetic effect in time-reversal-invariant Weyl semimetals with axionic charge density waves. *Phys. Rev. B* **104**, 174406 (2021). URL https://link.aps.org/doi/10.1103/PhysRevB.104.174406.

[44] Lin, K.-S. & Bradlyn, B. Simulating higher-order topological insulators in density wave insulators. *Phys. Rev. B* **103**, 245107 (2021). URL https://link.aps.org/doi/10.1103/PhysRevB.103.245107.

[45] Shi, W. *et al.* A charge-density-wave topological semimetal. *Nature Physics* **17**, 381–387 (2021). URL https://doi.org/10.1038/s41567-020-01104-z.

[46] Gooth, J. *et al.* Axionic charge-density wave in the Weyl semimetal $(TaSe_4)_2I$. *Nature* **575**, 315–319 (2019). URL https://doi.org/10.1038/s41586-019-1630-4.

[47] Peterson, C. W., Li, T., Jiang, W., Hughes, T. L. & Bahl, G. Trapped fractional charges at bulk defects in topological insulators. *Nature* **589**, 376–380 (2021). URL https://doi.org/10.1038/s41586-020-03117-3.

[48] Liu, Y. *et al.* Bulk–disclination correspondence in topological crystalline insulators. *Nature* **589**, 381–385 (2021). URL https://doi.org/10.1038/s41586-020-03125-3.

[49] Castro Neto, A. H., Guinea, F., Peres, N. M. R., Novoselov, K. S. & Geim, A. K. The electronic properties of graphene. *Rev. Mod. Phys.* **81**, 109–162 (2009). URL https://link.aps.org/doi/10.1103/RevModPhys.81.109.

[50] DiVincenzo, D. P. & Mele, E. J. Self-consistent effective-mass theory for intralayer screening in graphite intercalation compounds. *Phys. Rev. B* **29**, 1685–1694 (1984). URL http://link.aps.org/doi/10.1103/PhysRevB.29.1685.

[51] van Miert, G., Ortix, C. & Smith, C. M. Topological origin of edge states in two-dimensional inversion-symmetric insulators and semimetals. *2D Materials* **4**, 015023 (2016). URL https://doi.org/10.1088/2053-1583/4/1/015023.

[52] Armitage, N. P., Mele, E. J. & Vishwanath, A. Weyl and Dirac semimetals in three-dimensional solids. *Rev. Mod. Phys.* **90**, 015001 (2018). URL https://link.aps.org/doi/10.1103/RevModPhys.90.015001.

[53] Yan, B. & Felser, C. Topological Materials: Weyl Semimetals. *Annual Review of Condensed Matter Physics* **8**, 337–354 (2017). URL https://doi.org/10.1146/annurev-conmatphys-031016-025458.

[54] Liu, Z. K. *et al.* Discovery of a Three-Dimensional Topological Dirac Semimetal, Na_3Bi. *Science* **343**, 864–867 (2014). URL http://science.sciencemag.org/content/343/6173/864.

[55] Chiu, C.-K. & Schnyder, A. P. Classification of crystalline topological semimetals with an application to Na_3Bi. *Journal of Physics: Conference Series* **603**, 012002 (2015). URL http://stacks.iop.org/1742-6596/603/i=1/a=012002.

[56] Yang, B.-J. & Nagaosa, N. Classification of stable three-dimensional Dirac semimetals with nontrivial topology. *Nature Communications* **5**, 4898 (2014). URL https://doi.org/10.1038/ncomms5898.

[57] Wang, Z., Weng, H., Wu, Q., Dai, X. & Fang, Z. Three-dimensional Dirac semimetal and quantum transport in Cd_3As_2. *Phys. Rev. B* **88**, 125427 (2013). URL http://link.aps.org/doi/10.1103/PhysRevB.88.125427.

[58] Xu, S.-Y. *et al.* Observation of Fermi arc surface states in a topological metal. *Science* **347**, 294–298 (2015). URL https://science.sciencemag.org/content/347/6219/294.

[59] Borisenko, S. *et al.* Experimental Realization of a Three-Dimensional Dirac Semimetal. *Phys. Rev. Lett.* **113**, 027603 (2014). URL https://link.aps.org/doi/10.1103/PhysRevLett.113.027603.

[60] Young, S. M. *et al.* Dirac Semimetal in Three Dimensions. *Phys. Rev. Lett.* **108**, 140405 (2012). URL https://link.aps.org/doi/10.1103/PhysRevLett.108.140405.

[61] Wan, X., Turner, A. M., Vishwanath, A. & Savrasov, S. Y. Topological semimetal and Fermi-arc surface states in the electronic structure of pyrochlore iridates. *Phys. Rev. B* **83**, 205101 (2011). URL https://link.aps.org/doi/10.1103/PhysRevB.83.205101.

[62] Weng, H., Fang, C., Fang, Z., Bernevig, B. A. & Dai, X. Weyl Semimetal Phase in Noncentrosymmetric Transition-Metal Monophosphides. *Phys. Rev. X* **5**, 011029 (2015). URL https://link.aps.org/doi/10.1103/PhysRevX.5.011029.

[63] Kim, Y., Wieder, B. J., Kane, C. L. & Rappe, A. M. Dirac Line Nodes in Inversion-Symmetric Crystals. *Phys. Rev. Lett.* **115**, 036806 (2015). URL https://link.aps.org/doi/10.1103/PhysRevLett.115.036806.

[64] Fang, C., Chen, Y., Kee, H.-Y. & Fu, L. Topological nodal line semimetals with and without spin-orbital coupling. *Phys. Rev. B* **92**, 081201 (2015). URL https://link.aps.org/doi/10.1103/PhysRevB.92.081201.

[65] Wieder, B. J., Kim, Y., Rappe, A. M. & Kane, C. L. Double Dirac Semimetals in Three Dimensions. *Phys. Rev. Lett.* **116**, 186402 (2016). URL http://link.aps.org/doi/10.1103/PhysRevLett.116.186402.

[66] Chang, G. *et al.* Topological quantum properties of chiral crystals. *Nature Materials* **17**, 978–985 (2018). URL https://doi.org/10.1038/s41563-018-0169-3.

[67] Bradlyn, B. *et al.* Beyond Dirac and Weyl fermions: Unconventional quasiparticles in conventional crystals. *Science* **353**, aaf5037 (2016). URL https://www.science.org/doi/abs/10.1126/science.aaf5037.

[68] Chang, G. *et al.* Unconventional Chiral Fermions and Large Topological Fermi Arcs in RhSi. *Phys. Rev. Lett.* **119**, 206401 (2017). URL https://link.aps.org/doi/10.1103/PhysRevLett.119.206401.

[69] Tang, P., Zhou, Q. & Zhang, S.-C. Multiple Types of Topological Fermions in Transition Metal Silicides. *Phys. Rev. Lett.* **119**, 206402 (2017). URL https://link.aps.org/doi/10.1103/PhysRevLett.119.206402.

[70] Wieder, B. J. *et al.* Strong and fragile topological Dirac semimetals with higher-order Fermi arcs. *Nature Communications* **11**, 627 (2020). URL https://doi.org/10.1038/s41467-020-14443-5.

[71] Ran, Y., Zhang, Y. & Vishwanath, A. One-dimensional topologically protected modes in topological insulators with lattice dislocations. *Nature Physics* **5**, 298–303 (2009). URL https://doi.org/10.1038/nphys1220.

[72] Teo, J. C. Y. & Kane, C. L. Topological defects and gapless modes in insulators and superconductors. *Phys. Rev. B* **82**, 115120 (2010). URL https://link.aps.org/doi/10.1103/PhysRevB.82.115120.

[73] Sessi, P. *et al.* Robust spin-polarized midgap states at step edges of topological crystalline insulators. *Science* **354**, 1269–1273 (2016). URL http://science.sciencemag.org/content/354/6317/1269.

[74] Barkeshli, M. & Qi, X.-L. Topological Nematic States and Non-Abelian Lattice Dislocations. *Phys. Rev. X* **2**, 031013 (2012). URL https://link.aps.org/doi/10.1103/PhysRevX.2.031013.

[75] Bulmash, D., Hosur, P., Zhang, S.-C. & Qi, X.-L. Unified Topological Response Theory For Gapped and Gapless Free Fermions. *Phys. Rev. X* **5**, 021018 (2015). URL https://link.aps.org/doi/10.1103/PhysRevX.5.021018.

[76] Juričić, V., Mesaros, A., Slager, R.-J. & Zaanen, J. Universal probes of two-dimensional topological insulators: Dislocation and π flux. *Phys. Rev. Lett.* **108**, 106403 (2012). URL https://link.aps.org/doi/10.1103/PhysRevLett.108.106403.

[77] Imura, K.-I., Takane, Y. & Tanaka, A. Weak topological insulator with protected gapless helical states. *Phys. Rev. B* **84**, 035443 (2011). URL https://link.aps.org/doi/10.1103/PhysRevB.84.035443.

[78] Xu, Y. *et al.* Connection topology of step edge state bands at the surface of a three dimensional topological insulator. *New Journal of Physics* **20**, 073014 (2018). URL http://stacks.iop.org/1367-2630/20/i=7/a=073014.

[79] Queiroz, R., Fulga, I. C., Avraham, N., Beidenkopf, H. & Cano, J. Partial lattice defects in higher-order topological insulators. *Phys. Rev. Lett.* **123**, 266802 (2019). URL https://link.aps.org/doi/10.1103/PhysRevLett.123.266802.

[80] Slager, R.-J., Mesaros, A., Juričić, V. & Zaanen, J. Interplay between electronic topology and crystal symmetry: Dislocation-line modes in topological band insulators. *Phys. Rev. B* **90**, 241403 (2014). URL https://link.aps.org/doi/10.1103/PhysRevB.90.241403.

[81] You, Y., Cho, G. Y. & Hughes, T. L. Response properties of axion insulators and Weyl semimetals driven by screw dislocations and dynamical axion strings. *Phys. Rev. B* **94**, 085102 (2016). URL https://link.aps.org/doi/10.1103/PhysRevB.94.085102.

[82] Takane, Y. Regularized Framework of a Weyl Equation for Describing a Weyl Semimetal: Application to the Case with a Screw Dislocation. *Journal of the Physical Society of Japan* **86**, 123708 (2017). URL https://doi.org/10.7566/JPSJ.86.123708.

[83] Takane, Y. Chiral Surface States on the Step Edge in a Weyl Semimetal. *Journal of the Physical Society of Japan* **86**, 104709 (2017). URL https://doi.org/10.7566/JPSJ.86.104709.

[84] Chernodub, M. N. & Zubkov, M. A. Chiral anomaly in Dirac semimetals due to dislocations. *Phys. Rev. B* **95**, 115410 (2017). URL https://link.aps.org/doi/10.1103/PhysRevB.95.115410.

[85] Phillips, M. & Mele, E. J. Zero modes on zero-angle grain boundaries in graphene. *Phys. Rev. B* **91**, 125404 (2015). URL https://link.aps.org/doi/10.1103/PhysRevB.91.125404.

[86] Phillips, M. & Mele, E. J. Charge and spin transport on graphene grain boundaries in a quantizing magnetic field. *Phys. Rev. B* **96**, 041403 (2017). URL https://link.aps.org/doi/10.1103/PhysRevB.96.041403.

[87] Killi, M., Wei, T.-C., Affleck, I. & Paramekanti, A. Tunable Luttinger Liquid Physics in Biased Bilayer Graphene. *Phys. Rev. Lett.* **104**, 216406 (2010). URL https://link.aps.org/doi/10.1103/PhysRevLett.104.216406.

[88] Wieder, B. J., Zhang, F. & Kane, C. L. Critical behavior of four-terminal conductance of bilayer graphene domain walls. *Phys. Rev. B* **92**, 085425 (2015). URL https://link.aps.org/doi/10.1103/PhysRevB.92.085425.

[89] Iordanskii, S. V. & Koshelev, A. E. Interaction of excitations with dislocations in a crystal. *ZETF* **90**, 1399 (1986). URL http://www.jetp.ac.ru/cgi-bin/dn/e_063_04_0820.pdf.

[90] Song, Z.-D. *et al.* Delocalization transition of a disordered axion insulator. *Phys. Rev. Lett.* **127**, 016602 (2021). URL https://link.aps.org/doi/10.1103/PhysRevLett.127.016602.

[91] Wang, Q. *et al.* Vortex states in an acoustic Weyl crystal with a topological lattice defect. *Nature Communications* **12**, 3654 (2021). URL https://doi.org/10.1038/s41467-021-23963-7.

[92] Bertoldo, F., Ali, S., Manti, S. & Thygesen, K. S. Quantum point defects in 2D materials: The QPOD database. *arXiv e-prints* arXiv:2110.01961 (2021). 2110.01961.

[93] Tuegel, T. I., Chua, V. & Hughes, T. L. Embedded topological insulators. *Phys. Rev. B* **100**, 115126 (2019). URL https://link.aps.org/doi/10.1103/PhysRevB.100.115126.

[94] Velury, S. & Hughes, T. L. Embedded topological semimetals. *Phys. Rev. B* **105**, 184105 (2022). URL https://link.aps.org/doi/10.1103/PhysRevB.105.184105.

[95] Benalcazar, W. A., Bernevig, B. A. & Hughes, T. L. Quantized electric multipole insulators. *Science* **357**, 61–66 (2017). URL http://science.sciencemag.org/content/357/6346/61.

[96] Zhang, F., Kane, C. L. & Mele, E. J. Surface state magnetization and chiral edge states on topological insulators. *Phys. Rev. Lett.* **110**, 046404 (2013). URL https://link.aps.org/doi/10.1103/PhysRevLett.110.046404.

[97] Ezawa, M. Magnetic second-order topological insulators and semimetals. *Phys. Rev. B* **97**, 155305 (2018). URL https://link.aps.org/doi/10.1103/PhysRevB.97.155305.

[98] Liu, F. & Wakabayashi, K. Novel Topological Phase with a Zero Berry Curvature. *Phys. Rev. Lett.* **118**, 076803 (2017). URL https://link.aps.org/doi/10.1103/PhysRevLett.118.076803.

[99] Franca, S., van den Brink, J. & Fulga, I. C. An anomalous higher-order topological insulator. *Phys. Rev. B* **98**, 201114 (2018). URL https://link.aps.org/doi/10.1103/PhysRevB.98.201114.

[100] Wang, Z., Wieder, B. J., Li, J., Yan, B. & Bernevig, B. A. Higher-Order Topology, Monopole Nodal Lines, and the Origin of Large Fermi Arcs in Transition Metal Dichalcogenides $X\mathrm{Te}_2$ ($X = \mathrm{Mo}, \mathrm{W}$). *Phys. Rev. Lett.* **123**, 186401 (2019). URL https://link.aps.org/doi/10.1103/PhysRevLett.123.186401.

[101] Matsugatani, A. & Watanabe, H. Connecting higher-order topological insulators to lower-dimensional topological insulators. *Phys. Rev. B* **98**, 205129 (2018). URL https://link.aps.org/doi/10.1103/PhysRevB.98.205129.

[102] Schindler, F. *et al.* Higher-order topology in bismuth. *Nature Physics* **14**, 918–924 (2018). URL https://doi.org/10.1038/s41567-018-0224-7.

[103] Song, Z., Zhang, T., Fang, Z. & Fang, C. Quantitative mappings between symmetry and topology in solids. *Nature Communications* **9**, 3530 (2018). URL https://doi.org/10.1038/s41467-018-06010-w.

[104] Po, H. C., Vishwanath, A. & Watanabe, H. Symmetry-based indicators of band topology in the 230 space groups. *Nature Communications* **8**, 50 (2017). URL https://doi.org/10.1038/s41467-017-00133-2.

[105] Khalaf, E., Po, H. C., Vishwanath, A. & Watanabe, H. Symmetry indicators and anomalous surface states of topological crystalline insulators. *Phys. Rev. X* **8**, 031070 (2018). URL https://link.aps.org/doi/10.1103/PhysRevX.8.031070.

[106] Varnava, N. & Vanderbilt, D. Surfaces of axion insulators. *Phys. Rev. B* **98**, 245117 (2018). URL https://link.aps.org/doi/10.1103/PhysRevB.98.245117.

[107] Ahn, J. & Yang, B.-J. Symmetry representation approach to topological invariants in $C_{2z}T$-symmetric systems. *Phys. Rev. B* **99**, 235125 (2019). URL https://link.aps.org/doi/10.1103/PhysRevB.99.235125.

[108] Varnava, N., Souza, I. & Vanderbilt, D. Axion coupling in the hybrid Wannier representation. *Phys. Rev. B* **101**, 155130 (2020). URL https://link.aps.org/doi/10.1103/PhysRevB.101.155130.

[109] Olsen, T., Taherinejad, M., Vanderbilt, D. & Souza, I. Surface theorem for the Chern-Simons axion coupling. *Phys. Rev. B* **95**, 075137 (2017). URL https://link.aps.org/doi/10.1103/PhysRevB.95.075137.

[110] Olsen, T., Rauch, T., Vanderbilt, D. & Souza, I. Gapless hinge states from adiabatic pumping of axion coupling. *Phys. Rev. B* **102**, 035166 (2020). URL https://link.aps.org/doi/10.1103/PhysRevB.102.035166.

[111] Drozdov, I. K. *et al.* One-dimensional topological edge states of bismuth bilayers. *Nature Physics* **10**, 664–669 (2014). URL https://doi.org/10.1038/nphys3048.

[112] Murani, A. *et al.* Ballistic edge states in bismuth nanowires revealed by squid interferometry. *Nature Communications* **8**, 15941 (2017). URL https://doi.org/10.1038/ncomms15941.

[113] Bernard, A. *et al.* Evidence for topological hinge states in a bismuth nanoring Josephson junction. *arXiv e-prints* arXiv:2110.13539 (2021). 2110.13539.

[114] Liang, S. *et al.* A gap-protected zero-Hall effect state in the quantum limit of the non-symmorphic metal KHgSb. *Nature Materials* **18**, 443–447 (2019). URL https://doi.org/10.1038/s41563-019-0303-x.

[115] Huang, F.-T. *et al.* Polar and phase domain walls with conducting interfacial states in a Weyl semimetal $MoTe_2$. *Nature Communications* **10**, 4211 (2019). URL https://doi.org/10.1038/s41467-019-11949-5.

[116] Wang, W. *et al.* Evidence for an edge supercurrent in the Weyl superconductor $MoTe_2$. *Science* **368**, 534–537 (2020). URL https://science.sciencemag.org/content/368/6490/534.

[117] Choi, Y.-B. *et al.* Evidence of higher-order topology in multilayer WTe_2 from Josephson coupling through anisotropic hinge states. *Nature Materials* **19**, 974–979 (2020). URL https://doi.org/10.1038/s41563-020-0721-9.

[118] Kononov, A. *et al.* One-Dimensional Edge Transport in Few-Layer WTe_2. *Nano Letters* **20**, 4228–4233 (2020). URL https://doi.org/10.1021/acs.nanolett.0c00658.

[119] Huang, C. *et al.* Edge superconductivity in multilayer WTe_2 Josephson junction. *National Science Review* **7**, 1468–1475 (2020). URL https://doi.org/10.1093/nsr/nwaa114.

[120] Wang, Y., Lee, G.-H. & Ali, M. N. Topology and superconductivity on the edge. *Nature Physics* **17**, 542–546 (2021). URL https://doi.org/10.1038/s41567-021-01190-7.

[121] Noguchi, R. *et al.* Evidence for a higher-order topological insulator in a three-dimensional material built from van der Waals stacking of bismuth-halide chains. *Nature Materials* **20**, 473–479 (2021). URL https://doi.org/10.1038/s41563-020-00871-7.

[122] Mao, P. *et al.* Observation of the Topologically Originated Edge States in large-gap Quasi-One-Dimensional a-Bi_4Br_4. *arXiv e-prints* arXiv:2007.00223 (2020). 2007.00223.

[123] Mao, P. *et al.* Ultralong carrier lifetime of topological edge states in a-Bi4Br4. *arXiv e-prints* arXiv:2007.00264 (2020). 2007.00264.

[124] Shumiya, N. *et al.* Evidence of a room-temperature quantum spin Hall edge state in a higher-order topological insulator. *Nature Materials* (2022). URL https://doi.org/10.1038/s41563-022-01304-3.

[125] Nayak, A. K. *et al.* Resolving the topological classification of bismuth with topological defects. *Science Advances* **5**, eaax6996 (2019). URL https://www.science.org/doi/abs/10.1126/sciadv.aax6996.

[126] Kang, W., Spathelf, F., Fauqué, B., Fuseya, Y. & Behnia, K. Boundary conductance in macroscopic bismuth crystals. *Nature Communications* **13**, 189 (2022). URL https://doi.org/10.1038/s41467-021-27721-7.

[127] Mermin, N. D. The topological theory of defects in ordered media. *Rev. Mod. Phys.* **51**, 591–648 (1979). URL https://link.aps.org/doi/10.1103/RevModPhys.51.591.

[128] Jia, Y.-z. *et al.* Prediction of topological crystalline insulators and topological phase transitions in two-dimensional PbTe films. *Phys. Chem. Chem. Phys.* **19**, 29647–29652 (2017). URL http://dx.doi.org/10.1039/C7CP04679K.

[129] Niu, C. *et al.* Topological crystalline insulator and quantum anomalous Hall states in IV-VI-based monolayers and their quantum wells. *Phys. Rev. B* **91**, 201401 (2015). URL https://link.aps.org/doi/10.1103/PhysRevB.91.201401.

[130] Liu, J., Qian, X. & Fu, L. Crystal Field Effect Induced Topological Crystalline Insulators In Monolayer IV-VI Semiconductors. *Nano Letters* **15**, 2657–2661 (2015). URL https://doi.org/10.1021/acs.nanolett.5b00308.

[131] Vergniory, M. G. *et al.* A complete catalogue of high-quality topological materials. *Nature* **566**, 480–485 (2019). URL https://doi.org/10.1038/s41586-019-0954-4.

[132] Vergniory, M. G. *et al.* All topological bands of all nonmagnetic stoichiometric materials. *Science* **376**, eabg9094 (2022). URL https://www.science.org/doi/abs/10.1126/science.abg9094.

[133] Robredo, I. n., Vergniory, M. G. & Bradlyn, B. Higher-order and crystalline topology in a phenomenological tight-binding model of lead telluride. *Phys. Rev. Materials* **3**, 041202 (2019). URL https://link.aps.org/doi/10.1103/PhysRevMaterials.3.041202.

[134] Po, H. C., Watanabe, H. & Vishwanath, A. Fragile Topology and Wannier Obstructions. *Phys. Rev. Lett.* **121**, 126402 (2018). URL https://link.aps.org/doi/10.1103/PhysRevLett.121.126402.

[135] Cano, J. *et al.* Topology of disconnected elementary band representations. *Phys. Rev. Lett.* **120**, 266401 (2018). URL https://link.aps.org/doi/10.1103/PhysRevLett.120.266401.

[136] Bouhon, A., Black-Schaffer, A. M. & Slager, R.-J. Wilson loop approach to fragile topology of split elementary band representations and topological crystalline insulators with time-reversal symmetry. *Phys. Rev. B* **100**, 195135 (2019). URL https://link.aps.org/doi/10.1103/PhysRevB.100.195135.

[137] Bradlyn, B., Wang, Z., Cano, J. & Bernevig, B. A. Disconnected elementary band representations, fragile topology, and Wilson loops as topological indices: An example on the triangular lattice. *Phys. Rev. B* **99**, 045140 (2019). URL https://link.aps.org/doi/10.1103/PhysRevB.99.045140.

[138] Song, Z. *et al.* All magic angles in twisted bilayer graphene are topological. *Phys. Rev. Lett.* **123**, 036401 (2019). URL https://link.aps.org/doi/10.1103/PhysRevLett.123.036401.

[139] Zou, L., Po, H. C., Vishwanath, A. & Senthil, T. Band structure of twisted bilayer graphene: Emergent symmetries, commensurate approximants, and Wannier obstructions. *Phys. Rev. B* **98**, 085435 (2018). URL https://link.aps.org/doi/10.1103/PhysRevB.98.085435.

[140] Po, H. C., Zou, L., Senthil, T. & Vishwanath, A. Faithful tight-binding models and fragile topology of magic-angle bilayer graphene. *Phys. Rev. B* **99**, 195455 (2019). URL https://link.aps.org/doi/10.1103/PhysRevB.99.195455.

[141] Liu, S., Vishwanath, A. & Khalaf, E. Shift insulators: Rotation-protected two-dimensional topological crystalline insulators. *Phys. Rev. X* **9**, 031003 (2019). URL https://link.aps.org/doi/10.1103/PhysRevX.9.031003.

[142] Peri, V. *et al.* Experimental characterization of fragile topology in an acoustic metamaterial. *Science* **367**, 797–800 (2020). URL https://science.sciencemag.org/content/367/6479/797.

[143] Song, Z.-D., Elcoro, L. & Bernevig, B. A. Twisted bulk-boundary correspondence of fragile topology. *Science* **367**, 794–797 (2020). URL https://science.sciencemag.org/content/367/6479/794.

[144] Ahn, J., Kim, D., Kim, Y. & Yang, B.-J. Band topology and linking structure of nodal line semimetals with Z_2 monopole charges. *Phys. Rev. Lett.* **121**, 106403 (2018). URL https://link.aps.org/doi/10.1103/PhysRevLett.121.106403.

[145] Else, D. V., Po, H. C. & Watanabe, H. Fragile topological phases in interacting systems. *Phys. Rev. B* **99**, 125122 (2019). URL https://link.aps.org/doi/10.1103/PhysRevB.99.125122.

[146] Kooi, S., van Miert, G. & Ortix, C. The bulk-corner correspondence of time-reversal symmetric insulators. *npj Quantum Materials* **6**, 1 (2021). URL https://doi.org/10.1038/s41535-020-00300-7.

[147] Bouhon, A., Lange, G. F. & Slager, R.-J. Topological correspondence between magnetic space group representations and subdimensions. *Phys. Rev. B* **103**, 245127 (2021). URL https://link.aps.org/doi/10.1103/PhysRevB.103.245127.

[148] Lange, G. F., Bouhon, A. & Slager, R.-J. Subdimensional topologies, indicators, and higher order boundary effects. *Phys. Rev. B* **103**, 195145 (2021). URL https://link.aps.org/doi/10.1103/PhysRevB.103.195145.

[149] Ahn, J., Park, S. & Yang, B.-J. Failure of Nielsen-Ninomiya Theorem and Fragile Topology in Two-Dimensional Systems with Space-Time Inversion Symmetry: Application to Twisted Bilayer Graphene at Magic Angle. *Phys. Rev. X* **9**, 021013 (2019). URL https://link.aps.org/doi/10.1103/PhysRevX.9.021013.

[150] Hwang, Y., Ahn, J. & Yang, B.-J. Fragile topology protected by inversion symmetry: Diagnosis, bulk-boundary correspondence, and Wilson loop. *Phys. Rev. B* **100**, 205126 (2019). URL https://link.aps.org/doi/10.1103/PhysRevB.100.205126.

[151] Song, Z.-D., Elcoro, L., Xu, Y.-F., Regnault, N. & Bernevig, B. A. Fragile phases as affine monoids: Classification and material examples. *Phys. Rev. X* **10**, 031001 (2020). URL https://link.aps.org/doi/10.1103/PhysRevX.10.031001.

[152] Bradlyn, B. *et al.* Topological quantum chemistry. *Nature* **547**, 298–305 (2017). URL https://doi.org/10.1038/nature23268.

[153] Vergniory, M. G. *et al.* Graph theory data for topological quantum chemistry. *Phys. Rev. E* **96**, 023310 (2017). URL https://link.aps.org/doi/10.1103/PhysRevE.96.023310.

[154] Cano, J. *et al.* Building blocks of topological quantum chemistry: Elementary band representations. *Phys. Rev. B* **97**, 035139 (2018). URL https://link.aps.org/doi/10.1103/PhysRevB.97.035139.

[155] Elcoro, L. *et al.* Magnetic topological quantum chemistry. *Nature Communications* **12**, 5965 (2021). URL https://doi.org/10.1038/s41467-021-26241-8.

[156] Cano, J., Elcoro, L., Aroyo, M. I., Bernevig, B. A. & Bradlyn, B. Topology invisible to eigenvalues in obstructed atomic insulators. *Phys. Rev. B* **105**, 125115 (2022). URL https://link.aps.org/doi/10.1103/PhysRevB.105.125115.

[157] Hirayama, M., Matsuishi, S., Hosono, H. & Murakami, S. Electrides as a new platform of topological materials. *Phys. Rev. X* **8**, 031067 (2018). URL https://link.aps.org/doi/10.1103/PhysRevX.8.031067.

[158] Nie, S., Bernevig, B. A. & Wang, Z. Sixfold excitations in electrides. *Phys. Rev. Research* **3**, L012028 (2021). URL https://link.aps.org/doi/10.1103/PhysRevResearch.3.L012028.

[159] Nie, S. *et al.* Application of topological quantum chemistry in electrides. *Phys. Rev. B* **103**, 205133 (2021). URL https://link.aps.org/doi/10.1103/PhysRevB.103.205133.

[160] Xu, Y. *et al.* Filling-Enforced Obstructed Atomic Insulators. *arXiv e-prints* arXiv:2106.10276 (2021). 2106.10276.

[161] Gao, J. *et al.* Unconventional materials: the mismatch between electronic charge centers and atomic positions. *Science Bulletin* (2022). URL https://www.sciencedirect.com/science/article/pii/S2095927321008045.

[162] Xu, Y. *et al.* Three-Dimensional Real Space Invariants, Obstructed Atomic Insulators and A New Principle for Active Catalytic Sites. *arXiv e-prints* arXiv:2111.02433 (2021). 2111.02433.

[163] Li, G. *et al.* Obstructed surface states as the descriptor for predicting catalytic active sites in inorganic crystalline materials. *Advanced Materials* 2201328 (2022). URL https://onlinelibrary.wiley.com/doi/abs/10.1002/adma.202201328.

[164] Qi, X.-L. & Zhang, S.-C. Spin-Charge Separation in the Quantum Spin Hall State. *Phys. Rev. Lett.* **101**, 086802 (2008). URL https://link.aps.org/doi/10.1103/PhysRevLett.101.086802.

[165] Ran, Y., Vishwanath, A. & Lee, D.-H. Spin-charge separated solitons in a topological band insulator. *Phys. Rev. Lett.* **101**, 086801 (2008). URL https://link.aps.org/doi/10.1103/PhysRevLett.101.086801.

[166] Ringel, Z. & Stern, A. $\mathbb{Z}_2$ anomaly and boundaries of topological insulators. *Phys. Rev. B* **88**, 115307 (2013). URL https://link.aps.org/doi/10.1103/PhysRevB.88.115307.

[167] Rosenberg, G., Guo, H.-M. & Franz, M. Wormhole effect in a strong topological insulator. *Phys. Rev. B* **82**, 041104 (2010). URL https://link.aps.org/doi/10.1103/PhysRevB.82.041104.

[168] Gong, M. *et al.* Transport study of the wormhole effect in three-dimensional topological insulators. *Phys. Rev. B* **102**, 165425 (2020). URL https://link.aps.org/doi/10.1103/PhysRevB.102.165425.

[169] Ostrovsky, P. M., Gornyi, I. V. & Mirlin, A. D. Interaction-Induced Criticality in $\mathbb{Z}_2$ Topological Insulators. *Phys. Rev. Lett.* **105**, 036803 (2010). URL https://link.aps.org/doi/10.1103/PhysRevLett.105.036803.

[170] Assaad, F. F., Bercx, M. & Hohenadler, M. Topological invariant and quantum spin models from magnetic π fluxes in correlated topological insulators. *Phys. Rev. X* **3**, 011015 (2013). URL https://link.aps.org/doi/10.1103/PhysRevX.3.011015.

[171] Lin, Z.-K. *et al.* Topological Wannier cycles induced by sub-unit-cell artificial gauge flux in a sonic crystal. *Nature Materials* **21**, 430–437 (2022). URL https://doi.org/10.1038/s41563-022-01200-w.

[172] Yue, C. *et al.* Symmetry-enforced chiral hinge states and surface quantum anomalous Hall effect in the magnetic axion insulator $Bi_{2-x}Sm_xSe_3$. *Nature Physics* **15**, 577–581 (2019). URL https://doi.org/10.1038/s41567-019-0457-0.

[173] Khalaf, E. Higher-order topological insulators and superconductors protected by inversion symmetry. *Phys. Rev. B* **97**, 205136 (2018). URL https://link.aps.org/doi/10.1103/PhysRevB.97.205136.

[174] Xu, Y. *et al.* High-throughput calculations of magnetic topological materials. *Nature* **586**, 702–707 (2020). URL https://doi.org/10.1038/s41586-020-2837-0.

[175] Watanabe, H., Po, H. C. & Vishwanath, A. Structure and topology of band structures in the 1651 magnetic space groups. *Science Advances* **4**, eaat8685 (2018). URL https://www.science.org/doi/abs/10.1126/sciadv.aat8685.

[176] Benalcazar, W. A., Li, T. & Hughes, T. L. Quantization of fractional corner charge in C_n-symmetric higher-order topological crystalline insulators. *Phys. Rev. B* **99**, 245151 (2019). URL https://link.aps.org/doi/10.1103/PhysRevB.99.245151.

[177] Liu, C.-X., Qi, X.-L. & Zhang, S.-C. Half quantum spin Hall effect on the surface of weak topological insulators. *Physica E: Low-dimensional Systems and Nanostructures* **44**, 906 – 911 (2012). URL http://www.sciencedirect.com/science/article/pii/S1386947711004000.

[178] Levin, M. & Stern, A. Fractional topological insulators. *Phys. Rev. Lett.* **103**, 196803 (2009). URL https://link.aps.org/doi/10.1103/PhysRevLett.103.196803.

[179] Neupert, T., Santos, L., Ryu, S., Chamon, C. & Mudry, C. Fractional topological liquids with time-reversal symmetry and their lattice realization. *Phys. Rev. B* **84**, 165107 (2011). URL https://link.aps.org/doi/10.1103/PhysRevB.84.165107.

[180] Wen, X.-G. Colloquium: Zoo of quantum-topological phases of matter. *Rev. Mod. Phys.* **89**, 041004 (2017). URL https://link.aps.org/doi/10.1103/RevModPhys.89.041004.

[181] Fu, L. & Kane, C. L. Topology, Delocalization via Average Symmetry and the Symplectic Anderson Transition. *Phys. Rev. Lett.* **109**, 246605 (2012). URL https://link.aps.org/doi/10.1103/PhysRevLett.109.246605.

[182] Mong, R. S. K., Bardarson, J. H. & Moore, J. E. Quantum transport and two-parameter scaling at the surface of a weak topological insulator. *Phys. Rev. Lett.* **108**, 076804 (2012). URL https://link.aps.org/doi/10.1103/PhysRevLett.108.076804.

[183] Kimchi, I., Chou, Y.-Z., Nandkishore, R. M. & Radzihovsky, L. Anomalous localization at the boundary of an interacting topological insulator. *Phys. Rev. B* **101**, 035131 (2020). URL https://link.aps.org/doi/10.1103/PhysRevB.101.035131.

[184] Ostrovsky, P. M., Gornyi, I. V. & Mirlin, A. D. Quantum criticality and minimal conductivity in graphene with long-range disorder. *Phys. Rev. Lett.* **98**, 256801 (2007). URL https://link.aps.org/doi/10.1103/PhysRevLett.98.256801.

[185] Grushin, A. G. & Palumbo, G. Fermionic dualities with axial gauge fields. *Phys. Rev. B* **102**, 115146 (2020). URL https://link.aps.org/doi/10.1103/PhysRevB.102.115146.

[186] Metlitski, M. A., Kane, C. L. & Fisher, M. P. A. Symmetry-respecting topologically ordered surface phase of three-dimensional electron topological insulators. *Phys. Rev. B* **92**, 125111 (2015). URL https://link.aps.org/doi/10.1103/PhysRevB.92.125111.

[187] Stern, A. Fractional topological insulators: A pedagogical review. *Annual Review of Condensed Matter Physics* **7**, 349–368 (2016). URL https://doi.org/10.1146/annurev-conmatphys-031115-011559.

[188] Maciejko, J., Qi, X.-L., Karch, A. & Zhang, S.-C. Fractional topological insulators in three dimensions. *Phys. Rev. Lett.* **105**, 246809 (2010). URL https://link.aps.org/doi/10.1103/PhysRevLett.105.246809.

[189] Mross, D. F., Essin, A. & Alicea, J. Composite Dirac Liquids: Parent States for Symmetric Surface Topological Order. *Phys. Rev. X* **5**, 011011 (2015). URL https://link.aps.org/doi/10.1103/PhysRevX.5.011011.

[190] Wang, C. & Senthil, T. Half-filled Landau level, topological insulator surfaces, and three-dimensional quantum spin liquids. *Phys. Rev. B* **93**, 085110 (2016). URL https://link.aps.org/doi/10.1103/PhysRevB.93.085110.

[191] Zou, L., Wang, C. & Senthil, T. Symmetry enriched U(1) quantum spin liquids. *Phys. Rev. B* **97**, 195126 (2018). URL https://link.aps.org/doi/10.1103/PhysRevB.97.195126.

[192] Zou, L. Bulk characterization of topological crystalline insulators: Stability under interactions and relations to symmetry enriched $U(1)$ quantum spin liquids. *Phys. Rev. B* **97**, 045130 (2018). URL https://link.aps.org/doi/10.1103/PhysRevB.97.045130.

[193] Vishwanath, A. & Senthil, T. Physics of Three-Dimensional Bosonic Topological Insulators: Surface-Deconfined Criticality and Quantized Magnetoelectric Effect. *Phys. Rev. X* **3**, 011016 (2013). URL https://link.aps.org/doi/10.1103/PhysRevX.3.011016.

[194] Metlitski, M. A., Kane, C. L. & Fisher, M. P. A. Bosonic topological insulator in three dimensions and the statistical Witten effect. *Phys. Rev. B* **88**, 035131 (2013). URL https://link.aps.org/doi/10.1103/PhysRevB.88.035131.

[195] Fang, Y. & Cano, J. Higher-order topological insulators in antiperovskites. *Phys. Rev. B* **101**, 245110 (2020). URL https://link.aps.org/doi/10.1103/PhysRevB.101.245110.

[196] Zhang, T. *et al.* Catalogue of topological electronic materials. *Nature* **566**, 475–479 (2019). URL https://doi.org/10.1038/s41586-019-0944-6.

[197] Tang, F., Po, H. C., Vishwanath, A. & Wan, X. Efficient topological materials discovery using symmetry indicators. *Nature Physics* **15**, 470–476 (2019). URL https://doi.org/10.1038/s41567-019-0418-7.

[198] Tang, F., Po, H. C., Vishwanath, A. & Wan, X. Comprehensive search for topological materials using symmetry indicators. *Nature* **566**, 486–489 (2019). URL https://doi.org/10.1038/s41586-019-0937-5.

[199] Lee, E., Kim, R., Ahn, J. & Yang, B.-J. Two-dimensional higher-order topology in monolayer graphdiyne. *npj Quantum Materials* **5**, 1 (2020). URL https://doi.org/10.1038/s41535-019-0206-8.

[200] Nomura, T., Habe, T., Sakamoto, R. & Koshino, M. Three-dimensional graphdiyne as a topological nodal-line semimetal. *Phys. Rev. Materials* **2**, 054204 (2018). URL https://link.aps.org/doi/10.1103/PhysRevMaterials.2.054204.

[201] Lin, K.-S. *et al.* Spin-Resolved Topology and Partial Axion Angles in Three-Dimensional Insulators. *arXiv e-prints* arXiv:2207.10099 (2022). 2207.10099.

[202] Geier, M., Fulga, I. C. & Lau, A. Bulk-boundary-defect correspondence at disclinations in rotation-symmetric topological insulators and superconductors. *SciPost Phys.* **10**, 92 (2021). URL https://scipost.org/10.21468/SciPostPhys.10.4.092.

[203] Roy, B. & Juričić, V. Dislocation as a bulk probe of higher-order topological insulators. *Phys. Rev. Research* **3**, 033107 (2021). URL https://link.aps.org/doi/10.1103/PhysRevResearch.3.033107.

[204] You, Y., Bibo, J., Pollmann, F. & Hughes, T. L. Fracton Critical Point in Higher-Order Topological Phase Transition. *arXiv e-prints* arXiv:2008.01746 (2020). 2008.01746.

[205] Lee, E., Furusaki, A. & Yang, B.-J. Fractional charge bound to a vortex in two-dimensional topological crystalline insulators. *Phys. Rev. B* **101**, 241109 (2020). URL https://link.aps.org/doi/10.1103/PhysRevB.101.241109.

[206] Kooi, S. H., van Miert, G. & Ortix, C. Classification of crystalline insulators without symmetry indicators: Atomic and fragile topological phases in twofold rotation symmetric systems. *Phys. Rev. B* **100**, 115160 (2019). URL https://link.aps.org/doi/10.1103/PhysRevB.100.115160.

[207] Henke, J., Kurttutan, M., Kruthoff, J. & van Wezel, J. Topological invariants of rotationally symmetric crystals. *Phys. Rev. B* **104**, L201110 (2021). URL https://link.aps.org/doi/10.1103/PhysRevB.104.L201110.

[208] You, Y., Burnell, F. J. & Hughes, T. L. Multipolar topological field theories: Bridging higher order topological insulators and fractons. *Phys. Rev. B* **103**, 245128 (2021). URL https://link.aps.org/doi/10.1103/PhysRevB.103.245128.

[209] Majlin Skiff, R., de Juan, F., Queiroz, R., Beidenkopf, H. & Ilan, R. Confined vs. extended Dirac surface states in topological crystalline insulator nanowires. *arXiv e-prints* arXiv:2109.02023 (2021). 2109.02023.

[210] Petrides, I. & Zilberberg, O. A semiclassical treatment of spinor topological effects in driven, inhomogeneous insulators under external electromagnetic fields. *arXiv e-prints* arXiv:2203.14902 (2022). 2203.14902.

[211] Ringel, Z., Kraus, Y. E. & Stern, A. Strong side of weak topological insulators. *Phys. Rev. B* **86**, 045102 (2012). URL https://link.aps.org/doi/10.1103/PhysRevB.86.045102.

[212] Wilczek, F. Two applications of axion electrodynamics. *Phys. Rev. Lett.* **58**, 1799–1802 (1987). URL https://link.aps.org/doi/10.1103/PhysRevLett.58.1799.

[213] Goldstone, J. & Wilczek, F. Fractional quantum numbers on solitons. *Phys. Rev. Lett.* **47**, 986–989 (1981). URL https://link.aps.org/doi/10.1103/PhysRevLett.47.986.

[214] Niemi, A. & Semenoff, G. Fermion number fractionization in quantum field theory. *Physics Reports* **135**, 99 – 193 (1986). URL http://www.sciencedirect.com/science/article/pii/0370157386901675.

[215] Rice, M. J. & Mele, E. J. Elementary excitations of a linearly conjugated diatomic polymer. *Phys. Rev. Lett.* **49**, 1455–1459 (1982). URL https://link.aps.org/doi/10.1103/PhysRevLett.49.1455.

[216] Su, W. P., Schrieffer, J. R. & Heeger, A. J. Solitons in polyacetylene. *Phys. Rev. Lett.* **42**, 1698–1701 (1979). URL https://link.aps.org/doi/10.1103/PhysRevLett.42.1698.

[217] Su, W. P., Schrieffer, J. R. & Heeger, A. J. Soliton excitations in polyacetylene. *Phys. Rev. B* **22**, 2099–2111 (1980). URL https://link.aps.org/doi/10.1103/PhysRevB.22.2099.

[218] Heeger, A. J., Kivelson, S., Schrieffer, J. R. & Su, W. P. Solitons in conducting polymers. *Rev. Mod. Phys.* **60**, 781–850 (1988). URL https://link.aps.org/doi/10.1103/RevModPhys.60.781.

[219] Essin, A. M., Moore, J. E. & Vanderbilt, D. Magnetoelectric polarizability and axion electrodynamics in crystalline insulators. *Phys. Rev. Lett.* **102**, 146805 (2009). URL https://link.aps.org/doi/10.1103/PhysRevLett.102.146805.

[220] Mulligan, M. & Burnell, F. J. Topological insulators avoid the parity anomaly. *Phys. Rev. B* **88**, 085104 (2013). URL https://link.aps.org/doi/10.1103/PhysRevB.88.085104.

[221] Potter, A. C., Wang, C., Metlitski, M. A. & Vishwanath, A. Realizing topological surface states in a lower-dimensional flat band. *Phys. Rev. B* **96**, 235114 (2017). URL https://link.aps.org/doi/10.1103/PhysRevB.96.235114.

[222] Tiwari, A., Li, M.-H., Bernevig, B. A., Neupert, T. & Parameswaran, S. A. Unhinging the surfaces of higher-order topological insulators and superconductors. *Phys. Rev. Lett.* **124**, 046801 (2020). URL https://link.aps.org/doi/10.1103/PhysRevLett.124.046801.

[223] Bradley, C. & Cracknell, A. *The Mathematical Theory of Symmetry in Solids: Representation Theory for Point Groups and Space Groups* (Clarendon Press, 1972). URL https://books.google.com/books?id=OKXvAAAAMAAJ.

[224] Jackiw, R. & Rebbi, C. Solitons with fermion number $\frac{1}{2}$. *Phys. Rev. D* **13**, 3398–3409 (1976). URL https://link.aps.org/doi/10.1103/PhysRevD.13.3398.

[225] Zhang, F., Kane, C. L. & Mele, E. J. Surface states of topological insulators. *Phys. Rev. B* **86**, 081303 (2012). URL https://link.aps.org/doi/10.1103/PhysRevB.86.081303.

[226] Young, S. M. & Kane, C. L. Dirac Semimetals in Two Dimensions. *Phys. Rev. Lett.* **115**, 126803 (2015). URL http://link.aps.org/doi/10.1103/PhysRevLett.115.126803.

[227] Wieder, B. J. & Kane, C. L. Spin-orbit semimetals in the layer groups. *Phys. Rev. B* **94**, 155108 (2016). URL http://link.aps.org/doi/10.1103/PhysRevB.94.155108.

[228] Young, S. M. & Wieder, B. J. Filling-Enforced Magnetic Dirac Semimetals in Two Dimensions. *Phys. Rev. Lett.* **118**, 186401 (2017). URL https://link.aps.org/doi/10.1103/PhysRevLett.118.186401.

[229] Kunst, F. K., van Miert, G. & Bergholtz, E. J. Lattice models with exactly solvable topological hinge and corner states. *Phys. Rev. B* **97**, 241405 (2018). URL https://link.aps.org/doi/10.1103/PhysRevB.97.241405.

[230] van Miert, G. & Ortix, C. On the topological immunity of corner states in two-dimensional crystalline insulators. *npj Quantum Materials* **5**, 63 (2020). URL https://doi.org/10.1038/s41535-020-00265-7.

[231] Jung, M., Yu, Y. & Shvets, G. Exact higher-order bulk-boundary correspondence of corner-localized states. *Phys. Rev. B* **104**, 195437 (2021). URL https://link.aps.org/doi/10.1103/PhysRevB.104.195437.

[232] Schindler, F. *et al.* Fractional corner charges in spin-orbit coupled crystals. *Phys. Rev. Research* **1**, 033074 (2019). URL https://link.aps.org/doi/10.1103/PhysRevResearch.1.033074.

[233] Park, M. J., Kim, Y., Cho, G. Y. & Lee, S. Higher-order topological insulator in twisted bilayer graphene. *Phys. Rev. Lett.* **123**, 216803 (2019). URL https://link.aps.org/doi/10.1103/PhysRevLett.123.216803.

[234] Radha, S. K. & Lambrecht, W. R. L. Topological obstructed atomic limit insulators by annihilating Dirac fermions. *Phys. Rev. B* **103**, 075435 (2021). URL https://link.aps.org/doi/10.1103/PhysRevB.103.075435.

[235] Aroyo, M. I., Kirov, A., Capillas, C., Perez-Mato, J. M. & Wondratschek, H. Bilbao Crystallographic Server. II. Representations of crystallographic point groups and space groups. *Acta Crystallographica Section A* **62**, 115–128 (2006). URL https://doi.org/10.1107/S0108767305040286.

[236] Altmann, S. & Herzig, P. *Point-Group Theory Tables* (University of Vienna, 2011). URL http://phaidra.univie.ac.at/o:104731.

[237] Litvin, D. B. *Magnetic Group Tables* (International Union of Crystallography, 2013).

[238] Aroyo, M. I. *et al.* Bilbao Crystallographic Server: I. Databases and crystallographic computing programs. *Zeitschrift für Kristallographie - Crystalline Materials* **221**, 15 – 27 (2006). URL https://www.degruyter.com/view/journals/zkri/221/1/article-p15.xml.

[239] Aroyo, M. I., Kirov, A., Capillas, C., Perez-Mato, J. M. & Wondratschek, H. Bilbao Crystallographic Server. II. Representations of crystallographic point groups and space groups. *Acta Crystallographica Section A* **62**, 115–128 (2006). URL https://doi.org/10.1107/S0108767305040286.

[240] Schindler, F. & Bernevig, B. A. Noncompact atomic insulators. *Phys. Rev. B* **104**, L201114 (2021). URL https://link.aps.org/doi/10.1103/PhysRevB.104.L201114.

[241] Zak, J. Band representations and symmetry types of bands in solids. *Phys. Rev. B* **23**, 2824–2835 (1981). URL https://link.aps.org/doi/10.1103/PhysRevB.23.2824.

[242] Zak, J. Band representations of space groups. *Phys. Rev. B* **26**, 3010–3023 (1982). URL https://link.aps.org/doi/10.1103/PhysRevB.26.3010.

[243] Elcoro, L. *et al.* Double crystallographic groups and their representations on the Bilbao Crystallographic Server. *Journal of Applied Crystallography* **50**, 1457–1477 (2017). URL https://doi.org/10.1107/S1600576717011712.

[244] Yu, R., Qi, X. L., Bernevig, A., Fang, Z. & Dai, X. Equivalent expression of $\mathbb{Z}_2$ topological invariant for band insulators using the non-Abelian Berry connection. *Phys. Rev. B* **84**, 075119 (2011). URL https://link.aps.org/doi/10.1103/PhysRevB.84.075119.

[245] Fidkowski, L., Jackson, T. S. & Klich, I. Model Characterization of Gapless Edge Modes of Topological Insulators Using Intermediate Brillouin-Zone Functions. *Phys. Rev. Lett.* **107**, 036601 (2011). URL https://link.aps.org/doi/10.1103/PhysRevLett.107.036601.

[246] Alexandradinata, A., Dai, X. & Bernevig, B. A. Wilson-loop characterization of inversion-symmetric topological insulators. *Phys. Rev. B* **89**, 155114 (2014). URL https://link.aps.org/doi/10.1103/PhysRevB.89.155114.

[247] Thouless, D. J., Kohmoto, M., Nightingale, M. P. & den Nijs, M. Quantized Hall Conductance in a Two-Dimensional Periodic Potential. *Phys. Rev. Lett.* **49**, 405–408 (1982). URL https://link.aps.org/doi/10.1103/PhysRevLett.49.405.

[248] Thouless, D. J. Wannier functions for magnetic sub-bands. *Journal of Physics C: Solid State Physics* **17**, L325 (1984). URL http://stacks.iop.org/0022-3719/17/i=12/a=003.

[249] Thouless, D. J. Quantization of particle transport. *Phys. Rev. B* **27**, 6083–6087 (1983). URL https://link.aps.org/doi/10.1103/PhysRevB.27.6083.

[250] Soluyanov, A. A. & Vanderbilt, D. Wannier representation of $\mathbb{Z}_2$ topological insulators. *Phys. Rev. B* **83**, 035108 (2011). URL https://link.aps.org/doi/10.1103/PhysRevB.83.035108.

[251] Soluyanov, A. A. & Vanderbilt, D. Smooth gauge for topological insulators. *Phys. Rev. B* **85**, 115415 (2012). URL https://link.aps.org/doi/10.1103/PhysRevB.85.115415.

[252] Chen, X., Fidkowski, L. & Vishwanath, A. Symmetry enforced non-Abelian topological order at the surface of a topological insulator. *Phys. Rev. B* **89**, 165132 (2014). URL https://link.aps.org/doi/10.1103/PhysRevB.89.165132.

[253] Turner, A. M., Zhang, Y. & Vishwanath, A. Entanglement and inversion symmetry in topological insulators. *Phys. Rev. B* **82**, 241102 (2010). URL https://link.aps.org/doi/10.1103/PhysRevB.82.241102.

[254] Wu, L. *et al.* Quantized Faraday and Kerr rotation and axion electrodynamics of a 3D topological insulator. *Science* **354**, 1124–1127 (2016). URL http://science.sciencemag.org/content/354/6316/1124.

[255] Turner, A. M. & Vishwanath, A. Chapter 11 - Beyond Band Insulators: Topology of Semimetals and Interacting Phases. In Franz, M. & Molenkamp, L. (eds.) *Topological Insulators*, vol. 6 of *Contemporary Concepts of Condensed Matter Science*, 293 – 324 (Elsevier, 2013). URL http://www.sciencedirect.com/science/article/pii/B9780444633149000111.

[256] Marzari, N., Mostofi, A. A., Yates, J. R., Souza, I. & Vanderbilt, D. Maximally localized Wannier functions: Theory and applications. *Rev. Mod. Phys.* **84**, 1419–1475 (2012). URL https://link.aps.org/doi/10.1103/RevModPhys.84.1419.

[257] Peierls, R. Zur Theorie des Diamagnetismus von Leitungselektronen. *Zeitschrift für Physik* **80**, 763–791 (1933). URL https://doi.org/10.1007/BF01342591.

[258] Fu, L. & Kane, C. L. Time reversal polarization and a Z_2 adiabatic spin pump. *Phys. Rev. B* **74**, 195312 (2006). URL https://link.aps.org/doi/10.1103/PhysRevB.74.195312.

[259] Altland, A. & Zirnbauer, M. R. Nonstandard symmetry classes in mesoscopic normal-superconducting hybrid structures. *Phys. Rev. B* **55**, 1142–1161 (1997). URL https://link.aps.org/doi/10.1103/PhysRevB.55.1142.

[260] Kitaev, A. Periodic table for topological insulators and superconductors. *AIP Conference Proceedings* **1134**, 22–30 (2009). URL https://aip.scitation.org/doi/abs/10.1063/1.3149495.

[261] Zak, J. Berry's phase for energy bands in solids. *Phys. Rev. Lett.* **62**, 2747–2750 (1989). URL https://link.aps.org/doi/10.1103/PhysRevLett.62.2747.

[262] Kopsky, V. & Litvin, D. *International Tables for Crystallography, Volume E: Subperiodic Groups*. International Tables for Crystallography (Springer Netherlands, 2002). URL https://books.google.com/books?id=if8nMGopkNgC.

[263] Aroyo, M. I. *International Tables for Crystallography, Volume A: Space-Group Symmetry* (International Union of Crystallography, 2016). URL http://it.iucr.org/A/.

[264] Tang, F., Po, H. C., Vishwanath, A. & Wan, X. Topological materials discovery by large-order symmetry indicators. *Science Advances* **5**, eaau8725 (2019). URL https://www.science.org/doi/abs/10.1126/sciadv.aau8725.

[265] Choudhary, K., Garrity, K. F. & Tavazza, F. High-throughput discovery of topologically non-trivial materials using spin-orbit spillage. *Scientific Reports* **9**, 8534 (2019). URL https://doi.org/10.1038/s41598-019-45028-y.

[266] Vanderbilt, D. *Berry Phases in Electronic Structure Theory: Electric Polarization, Orbital Magnetization and Topological Insulators* (Cambridge University Press, 2018). URL https://books.google.com/books?id=485FtgEACAAJ.

[267] Fang, C., Gilbert, M. J. & Bernevig, B. A. Bulk topological invariants in noninteracting point group symmetric insulators. *Phys. Rev. B* **86**, 115112 (2012). URL https://link.aps.org/doi/10.1103/PhysRevB.86.115112.

[268] Gallego, S. V. *et al.* *MAGNDATA*: towards a database of magnetic structures. I.The commensurate case. *Journal of Applied Crystallography* **49**, 1750–1776 (2016).

[269] Nielsen, H. & Ninomiya, M. Absence of neutrinos on a lattice: (I). Proof by homotopy theory. *Nuclear Physics B* **185**, 20 – 40 (1981). URL http://www.sciencedirect.com/science/article/pii/0550321381903618.

[270] Peng, B., Jiang, Y., Fang, Z., Weng, H. & Fang, C. Topological classification and diagnosis in magnetically ordered electronic materials. *Phys. Rev. B* **105**, 235138 (2022). URL https://link.aps.org/doi/10.1103/PhysRevB.105.235138.

[271] Yang, J., Fang, C. & Liu, Z.-X. Symmetry-protected nodal points and nodal lines in magnetic materials. *Phys. Rev. B* **103**, 245141 (2021). URL https://link.aps.org/doi/10.1103/PhysRevB.103.245141.

[272] Călugăru, D. *et al.* General construction and topological classification of crystalline flat bands. *Nature Physics* **18**, 185–189 (2022). URL https://doi.org/10.1038/s41567-021-01445-3.

[273] Liu, P., Li, J., Han, J., Wan, X. & Liu, Q. Spin-group symmetry in magnetic materials with negligible spin-orbit coupling. *Phys. Rev. X* **12**, 021016 (2022). URL https://link.aps.org/doi/10.1103/PhysRevX.12.021016.

[274] Yang, J., Liu, Z.-X. & Fang, C. Symmetry Invariants of Spin Space Groups in Magnetic Materials. *arXiv e-prints* arXiv:2105.12738 (2021). 2105.12738.

[275] Vafek, O. & Vishwanath, A. Dirac Fermions in Solids: From High-T_c Cuprates and Graphene to Topological Insulators and Weyl Semimetals. *Annual Review of Condensed Matter Physics* **5**, 83–112 (2014). URL https://doi.org/10.1146/annurev-conmatphys-031113-133841.

[276] Kang, B., Shiozaki, K. & Cho, G. Y. Many-body order parameters for multipoles in solids. *Phys. Rev. B* **100**, 245134 (2019). URL https://link.aps.org/doi/10.1103/PhysRevB.100.245134.

[277] Qi, X.-L., Wu, Y.-S. & Zhang, S.-C. Topological quantization of the spin Hall effect in two-dimensional paramagnetic semiconductors. *Phys. Rev. B* **74**, 085308 (2006). URL https://link.aps.org/doi/10.1103/PhysRevB.74.085308.

[278] Jeon, S. & Kim, Y. Two-dimensional weak topological insulators in inversion-symmetric crystals. *Phys. Rev. B* **105**, L121101 (2022). URL https://link.aps.org/doi/10.1103/PhysRevB.105.L121101.

[279] van Miert, G. & Ortix, C. Excess charges as a probe of one-dimensional topological crystalline insulating phases. *Phys. Rev. B* **96**, 235130 (2017). URL https://link.aps.org/doi/10.1103/PhysRevB.96.235130.

[280] Bergerhoff, G., Hundt, R., Sievers, R. & Brown, I. D. The inorganic crystal structure data base. *Journal of Chemical Information and Computer Sciences* **23**, 66–69 (1983). URL https://pubs.acs.org/doi/abs/10.1021/ci00038a003.

[281] Kresse, G. & Hafner, J. Ab initio molecular dynamics for open-shell transition metals. *Phys. Rev. B* **48**, 13115–13118 (1993). URL https://link.aps.org/doi/10.1103/PhysRevB.48.13115.

[282] Kresse, G. & Furthmüller, J. Efficiency of ab-initio total energy calculations for metals and semiconductors using a plane-wave basis set. *Computational Materials Science* **6**, 15 – 50 (1996). URL http://www.sciencedirect.com/science/article/pii/0927025696000080.

[283] Blöchl, P. E. Projector augmented-wave method. *Phys. Rev. B* **50**, 17953–17979 (1994). URL https://link.aps.org/doi/10.1103/PhysRevB.50.17953.

[284] Kresse, G. & Joubert, D. From ultrasoft pseudopotentials to the projector augmented-wave method. *Phys. Rev. B* **59**, 1758–1775 (1999). URL https://link.aps.org/doi/10.1103/PhysRevB.59.1758.

[285] Perdew, J. P., Burke, K. & Ernzerhof, M. Generalized gradient approximation made simple. *Phys. Rev. Lett.* **77**, 3865–3868 (1996). URL https://link.aps.org/doi/10.1103/PhysRevLett.77.3865.

[286] Marzari, N. & Vanderbilt, D. Maximally localized generalized Wannier functions for composite energy bands. *Phys. Rev. B* **56**, 12847–12865 (1997). URL https://link.aps.org/doi/10.1103/PhysRevB.56.12847.

[287] Mostofi, A. A. *et al.* wannier90: A tool for obtaining maximally-localised Wannier functions. *Computer Physics Communications* **178**, 685 – 699 (2008). URL http://www.sciencedirect.com/science/article/pii/S0010465507004936.

[288] Mostofi, A. A. *et al.* An updated version of wannier90: A tool for obtaining maximally-localised Wannier functions. *Comput. Phys. Commun.* **185**, 2309 (2014).

[289] Iraola, M. *et al.* IrRep: Symmetry eigenvalues and irreducible representations of ab initio band structures. *Computer Physics Communications* **272**, 108226 (2022). URL https://www.sciencedirect.com/science/article/pii/S0010465521003386.

[290] Topological material database. https://topologicalquantumchemistry.com/.

[291] Bauer Pereira, P. *et al.* Lattice dynamics and structure of GeTe, SnTe and PbTe. *Phys. Status Solidi B* **250**, 1300–1307 (2013). URL https://onlinelibrary.wiley.com/doi/abs/10.1002/pssb.201248412.

[292] Frank, F. C. & Read, W. T. Multiplication processes for slow moving dislocations. *Phys. Rev.* **79**, 722–723 (1950). URL https://link.aps.org/doi/10.1103/PhysRev.79.722.

[293] Fulga, I. C., Avraham, N., Beidenkopf, H. & Stern, A. Coupled-layer description of topological crystalline insulators. *Phys. Rev. B* **94**, 125405 (2016). URL https://link.aps.org/doi/10.1103/PhysRevB.94.125405.
